# Supplementary material for: Alpha 2 agonists for sedation to produce better outcomes from critical illness (A2B Trial): protocol for a multicentre phase 3 pragmatic clinical and cost-effectiveness randomised trial in the UK
Source: BMJ Open. 2023 Dec 9;13(12):e078645. doi: 10.1136/bmjopen-2023-078645 (PMC10729141; doi:10.1136/bmjopen-2023-078645)
Supplement: Supplementary data [file bmjopen-2023-078645supp001.pdf]

Appendix

Contents

Appendix 1
Figure illustrating the consent process utilised for enrolling patients in the absence of patient capacity 2
Example of Consent Form – Personal Legal Representative Consent form 2
Example of consent form 3
Weight-based drug dosing algorithms used in the A2B trial 5
Clonidine Drug Regimen 5
Dexmedetomidine Drug regimen 6
Clinical management algorithms to guide dosing of intervention drugs in the A2B trial 7
CLONIDINE Flowchart 8
Dexmedetomidine Flowchart 10
Usual Care (propofol) flowchart 12
Trial Estimand (see also Statistical Analysis Plan) 14
Health Economic Evaluation 16
Overview 16
Within-trial analysis 16
Lifetime analysis 17

Figure illustrating the consent process utilised for enrolling patients in the absence of patient capacity

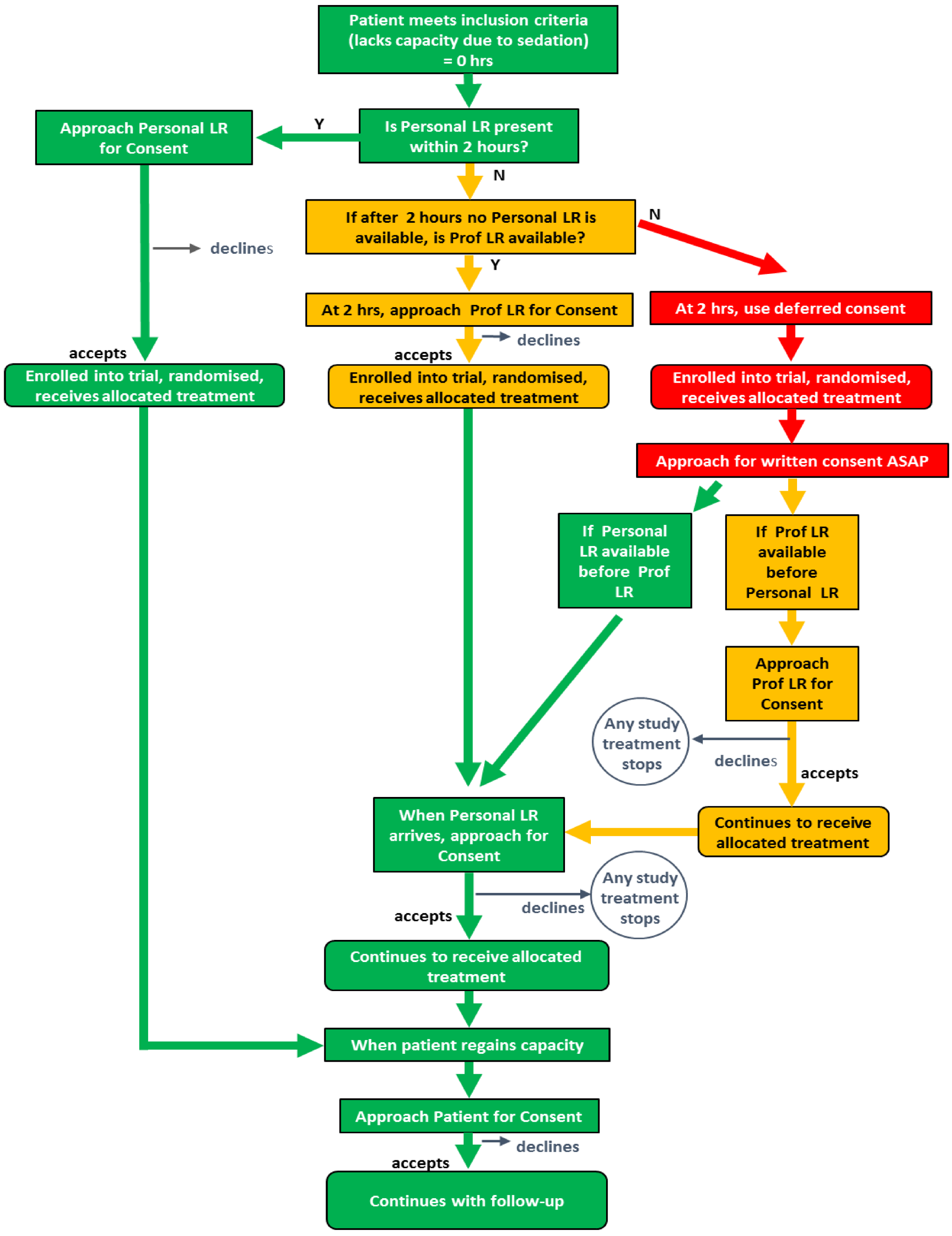

Personal LR = Personal Legal Representative   Prof LR = Professional Legal Representative

Example of Consent Form – Personal Legal Representative Consent form

(Additional consent forms used for Professional Legal Representative Consent, and for Patient Consent to remain in trial (once regained capacity)

Example of consent form

Participant ID:		Centre ID	

CONSENT FORM
England, Wales, Northern Ireland
Guardian or Nearest Relative
(Personal Legal Representative – Pre randomisation)

ALPHA-2 AGONISTS FOR SEDATION TO PRODUCE BETTER OUTCOMES FROM CRITICAL ILLNESS ('A2B TRIAL')

Please **initial** box

1. I confirm that I have read and understand the Personal LR Pre-randomisation information sheet England/Wales/Northern Ireland (18MAY2023 V2.0) for the above study. I have had the opportunity to consider the information, ask questions and have had these questions answered satisfactorily. ☐
2. I understand that my relative's participation is voluntary and that I am free to withdraw my consent at any time without giving any reason and without my relative's medical care and/or legal rights being affected. ☐
3. I give permission for the research team to access my relative's medical records for the purposes of this research study ☐
4. I understand that relevant sections of my relative's medical notes and data collected during the study may be looked at by individuals from the Sponsor (University of Edinburgh and/or NHS Lothian), from the NHS organisation or other regulatory authorities where it is relevant to their taking part in this research. I give permission for these individuals to have access to my relative's data and/or medical records. ☐
5. I give permission for my relative's personal information (including name, address, date of birth, telephone number and consent form) to be passed to the University of Edinburgh and Edinburgh Clinical Trials Unit for administration of the study and follow-up purposes. ☐
6. I give permission for my relative's hospital number to be collected and passed to the University of Edinburgh and Edinburgh Clinical Trials Unit. ☐
7. I agree that the information held and maintained by NHS Digital and other central UK NHS bodies may be used to provide information about my relative's health status. ☐
8. I agree to my relative taking part in the substudy which would involve giving two 20ml blood samples which will be used to study inflammation in the blood and for genetic DNA analysis. Yes ☐ No ☐
9. I give permission for DNA analysis, including whole genome sequencing, to be conducted on my relative's samples Yes ☐ No ☐
10. I agree that information collected about my relative can be used to support other research in the future, and may be shared anonymously with other researchers. Yes ☐ No ☐
11. I agree that my relative's blood and DNA samples can be used to support other research in the future, and may be shared anonymously with other researchers. Yes ☐ No ☐
12. I agree to provide my opinion on my relative's level of comfort and my ability to communicate with them and I give my permission for this data to be used. ☐
13. I agree to my relative taking part in the above study ☐

**Participant ID:**			**Centre ID**

I understand that my relative's data will not be shared beyond those noted on the consent form and that access will be managed via a secure system.

Please **initial** box.

☐ I confirm that I am Personal Legal Representative for ____

Relationship to participant ____

Name of Person Giving Consent	Date	Time	Signature
Name of person receiving consent	Date	Time	Signature

Weight-based drug dosing algorithms used in the A2B trial

Clonidine Drug Regimen

Weight Based Infusion Rates in <u>mls per hour</u> for Clonidine 15micrograms per ml

	Patient's weight (actual) in kilograms											
**Dose in micrograms per kilogram per hour**	**45**	**50**	**55**	**60**	**65**	**70**	**75**	**80**	**85**	**90**	**95**	**≥100**
**0.1**	0.3	0.3	0.4	0.4	0.4	0.5	0.5	0.5	0.6	0.6	0.6	0.7
**0.2**	0.6	0.7	0.7	0.8	0.9	0.9	1.0	1.1	1.1	1.2	1.3	1.3
**0.3**	0.9	1.0	1.1	1.2	1.3	1.4	1.5	1.6	1.7	1.8	1.9	2.0
**0.4**	1.2	1.3	1.5	1.6	1.7	1.9	2.0	2.1	2.3	2.4	2.5	2.7
**0.5**	1.5	1.7	1.8	2.0	2.2	2.3	2.5	2.7	2.8	3.0	3.2	3.3
**0.6**	1.8	2.0	2.2	2.4	2.6	2.8	3.0	3.2	3.4	3.6	3.8	4.0
**0.7**	2.1	2.3	2.6	2.8	3.0	3.3	3.5	3.7	4.0	4.2	4.4	4.7
**0.8**	2.4	2.7	2.9	3.2	3.5	3.7	4.0	4.3	4.5	4.8	5.1	5.3
**0.9**	2.7	3.0	3.3	3.6	3.9	4.2	4.5	4.8	5.1	5.4	5.7	6.0
**1.0**	3.0	3.3	3.7	4.0	4.3	4.7	5.0	5.3	5.7	6.0	6.3	6.7
**1.1**	3.3	3.7	4.0	4.4	4.8	5.1	5.5	5.9	6.2	6.6	7.0	7.3
**1.2**	3.6	4.0	4.4	4.8	5.2	5.6	6.0	6.4	6.8	7.2	7.6	8.0
**1.3**	3.9	4.3	4.8	5.2	5.6	6.1	6.5	6.9	7.4	7.8	8.2	8.7
**1.4**	4.2	4.7	5.1	5.6	6.1	6.5	7.0	7.5	7.9	8.4	8.9	9.3
**1.5**	4.5	5.0	5.5	6.0	6.5	7.0	7.5	8.0	8.5	9.0	9.5	10.0
**1.6**	4.8	5.3	5.9	6.4	6.9	7.5	8.0	8.5	9.1	9.6	10.1	10.7
**1.7**	5.1	5.7	6.2	6.8	7.4	7.9	8.5	9.1	9.6	10.2	10.8	11.3
**1.8**	5.4	6.0	6.6	7.2	7.8	8.4	9.0	9.6	10.2	10.8	11.4	12.0
**1.9**	5.7	6.3	7.0	7.6	8.2	8.9	9.5	10.1	10.8	11.4	12.0	12.7
**2.0**	6.0	6.7	7.3	8.0	8.7	9.3	10.0	10.7	11.3	12.0	12.7	13.3

For patients who weigh less than 45kg, please contact the trial management team for specific advice prior to randomisation.

Patients who weigh greater than 100kg will be dosed using the regimen suggested for 100kg weight. Please contact the trial management team for advice if required prior to randomisation.

Dexmedetomidine Drug regimen

Weight Based Infusion Rates in <u>mls per hour</u> for Dexmedetomidine 8 micrograms per ml

		Patient's weight (actual) in kilograms											
Dose in micrograms per kilogram per hour		**45**	**50**	**55**	**60**	**65**	**70**	**75**	**80**	**85**	**90**	**95**	**≥100**
	**0.1**	0.6	0.6	0.7	0.8	0.8	0.9	0.9	1.0	1.1	1.1	1.2	1.3
	**0.2**	1.1	1.3	1.4	1.5	1.6	1.8	1.9	2.0	2.1	2.3	2.4	2.5
	**0.3**	1.7	1.9	2.1	2.3	2.4	2.6	2.8	3.0	3.2	3.4	3.6	3.8
	**0.4**	2.3	2.5	2.8	3.0	3.3	3.5	3.8	4.0	4.3	4.5	4.8	5.0
	**0.5**	2.8	3.1	3.4	3.8	4.1	4.4	4.7	5.0	5.3	5.6	5.9	6.3
	**0.6**	3.4	3.8	4.1	4.5	4.9	5.3	5.6	6.0	6.4	6.8	7.1	7.5
	**0.7**	3.9	4.4	4.8	5.3	5.7	6.1	6.6	7.0	7.4	7.9	8.3	8.8
	**0.8**	4.5	5.0	5.5	6.0	6.5	7.0	7.5	8.0	8.5	9.0	9.5	10.0
	**0.9**	5.1	5.6	6.2	6.8	7.3	7.9	8.4	9.0	9.6	10.1	10.7	11.3
	**1.0**	5.6	6.3	6.9	7.5	8.1	8.8	9.4	10.0	10.6	11.3	11.9	12.5
	**1.1**	6.2	6.9	7.6	8.3	8.9	9.6	10.3	11.0	11.7	12.4	13.1	13.8
	**1.2**	6.8	7.5	8.3	9.0	9.8	10.5	11.3	12.0	12.8	13.5	14.3	15.0
	**1.3**	7.3	8.1	8.9	9.8	10.6	11.4	12.2	13.0	13.8	14.6	15.4	16.3
	**1.4**	7.9	8.8	9.6	10.5	11.4	12.3	13.1	14.0	14.9	15.8	16.6	17.5

For patients who weigh less than 45kg, please contact the trial management team for specific advice prior to randomisation.

Patients who weigh greater than 100kg will be dosed using the regimen suggested for 100kg weight. Please contact the trial management team for advice if required prior to randomisation.

Walsh TS, *et al. BMJ Open* 2023; 13:e078645. doi: 10.1136/bmjopen-2023-078645

Clinical management algorithms to guide dosing of intervention drugs in the A2B trial

Walsh TS, *et al. BMJ Open* 2023; 13:e078645. doi: 10.1136/bmjopen-2023-078645

CLONIDINE Flowchart

Clonidine Group Sedation Flowchart

An ICU Sedation Study

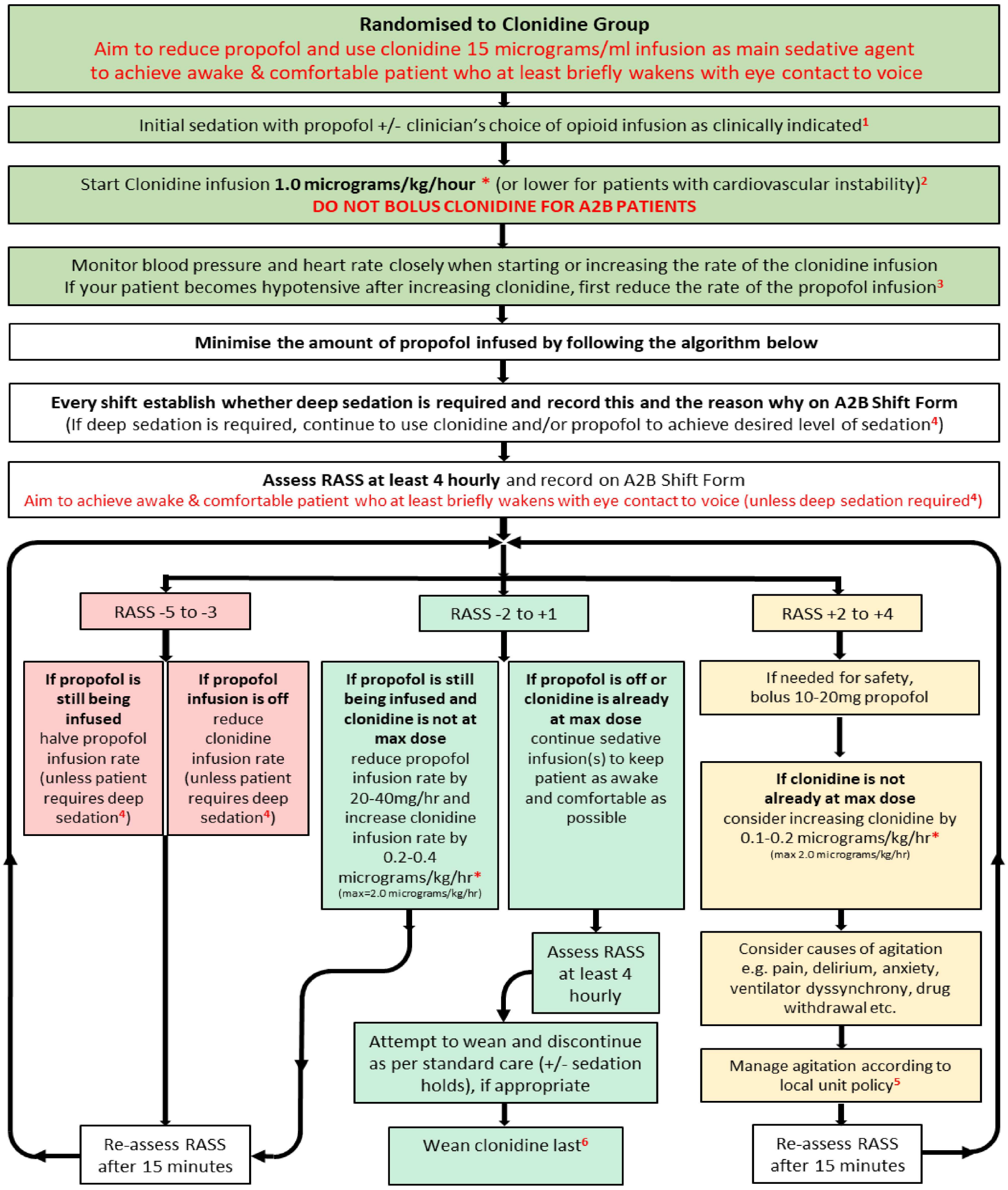

* **See A2B Clonidine infusion table for mls/hr infusion rates for patient weight**

[1] additional opioid boluses can be given as required

[2] if on more than 0.15 micrograms/kg/min noradrenaline to maintain target MAP, use lower starting dose initially

[3] PTO for advice on managing severe bradycardia or hypotension on the reverse of this page

[4] PTO for deep sedation advice on the reverse of this page

[5] dexmedetomidine should not be prescribed for the Clonidine group

[6] PTO for weaning advice on the reverse of this page

An ICU Sedation Study

Clonidine Group Sedation

A2B An ICU Sedation Study

TARGET: most awake and comfortable state considered safe (the least awake target will be "briefly wakens with eye contact to voice")

- Primary sedative agent is **CLONIDINE** (diluted with 5% glucose or 0.9% NaCl solution to a concentration of **15 micrograms per ml**)
- **Aim to reduce/stop propofol infusion.**
- All patients should receive opioid infusions for analgesia as clinically indicated at the discretion of clinical teams.
- **NB Clonidine has analgesic properties, so it may be possible to reduce opioids if pain is well controlled.**

Drugs you should not give:

- **Dexmedetomidine should <u>not</u> be used as first line sedation during the intervention period.**

How to manage agitation (RASS +2 TO +4)

- Maintain patient/staff safety by bolusing propofol if needed.
- Once agitation is under control, try to identify and manage the cause, using local unit policy for the management of pain/delirium/anxiety/withdrawal etc.
- Anticipate and avoid agitation; use opioid analgesia in advance of uncomfortable or painful interventions or procedures.

What to do if my patient develops severe bradycardia (HR<50 beats per minute)

- **If your patient's heart rate decreases to less than 50 beats per minute, clonidine should be temporarily stopped.**
- NB Clonidine's effect on heart rate can take several hours to resolve, so stopping the clonidine infusion may not immediately resolve bradycardia.
- Seek advice from medical staff who can review and prescribe, glycopyrronium, atropine, dobutamine, adrenaline or other agents, as per your ICU policy.
- Once heart rate is maintained above 50 beats per minute, clonidine should be re-commenced at a dose appropriate to the sedation target, but caution should be used when the clinical target is deep sedation.

What to do if my patient becomes hypotensive

- Hypotension should be treated as per local unit policy. Continuous fluid infusions, fluid challenges and vasopressor infusions are all permitted for patients in the A2B Trial.
- If changes to sedation are required as a result of hypotension, propofol should be decreased before clonidine, unless clinically contraindicated (e.g. if patient requires propofol for neuromuscular blockade).
- Continue clonidine infusion unless causing haemodynamic compromise, such that target MAP cannot be maintained with 0.15 micrograms/kg/min of noradrenaline. If haemodynamically compromised, consider halving the rate of the clonidine infusion, then halving again, or stopping, as needed. Clonidine can be restarted/increased once the patient is more stable, at the discretion of medical staff.
- NB Clonidine's effect on heart rate can take several hours to resolve, so stopping the clonidine infusion may not immediately resolve hypotension.

What if my patient requires deep sedation (RASS -3 TO -5) e.g. for neuromuscular blockade (muscle relaxant)?

- If medical staff have asked to keep your patient deeply sedated, please record the reason deep sedation was requested on the A2B Shift Form.
- **Patients who require neuromuscular blockade after randomisation must receive adequate sedation as per standard care, at the discretion of the treating clinician, to prevent awareness during paralysis. Clonidine alone may not provide adequate sedation to prevent awareness.**
- It is suggested that clonidine is titrated up to the maximum tolerated dose, but if additional sedative drugs are needed to achieve target deep sedation this can be achieved with propofol or benzodiazepine.
- When deep sedation is no longer requested by the caring clinician, aim to use clonidine as main sedative agent as per flowchart over page. It is suggested that any propofol or benzodiazepine sedation is decreased and stopped prior to reducing the dose of clonidine.

What if my patient needs an operative procedure/CT scan/MRI etc.?

- Increase sedation for transfer if needed, using clonidine and/or propofol.
- If required, anaesthesia should be administered as per local perioperative guidelines.
- Continue clonidine infusion unless haemodynamically compromised, in which case consider halving infusion rate and halving again or stopping, as needed.
- Following the procedure, again aim to reduce propofol and use clonidine as main sedative agent as per flowchart over page.

Weaning and discontinuing Clonidine

- **Clonidine can be abruptly discontinued, but slower weaning should be used if withdrawal reactions occur (rebound hypertension or tachycardia, agitation, sweating etc.). However, this should not delay weaning from ventilation as clonidine is relatively free from respiratory depressive effects, so can be safely continued post-extubation.**

PTO for Clonidine Group Sedation Flowchart on reverse of this page

Walsh TS, *et al. BMJ Open* 2023; 13:e078645. doi: 10.1136/bmjopen-2023-078645

Dexmedetomidine Flowchart

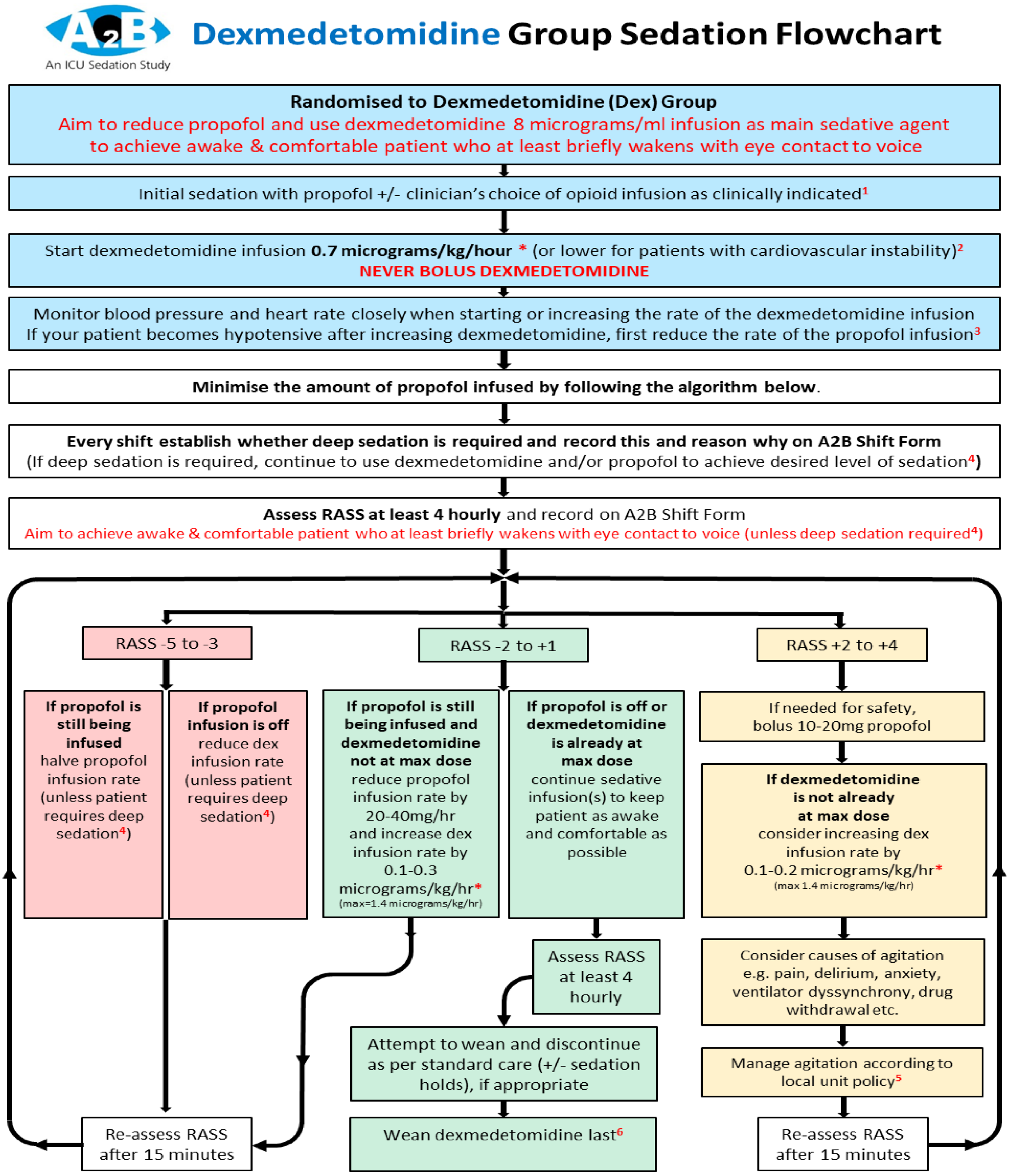

* **See A2B Dexmedetomidine infusion table for mls/hr infusion rates for patient weight**

[1] additional opioid boluses can be given as required

[2] if on more than 0.15 micrograms/kg/min noradrenaline to maintain target MAP, use lower starting dose initially

[3] PTO for advice on managing severe bradycardia or hypotension on the reverse of this page

[4] PTO for deep sedation advice on the reverse of this page

[5] clonidine should not be prescribed for the dexmedetomidine group

[6] PTO for weaning advice on the reverse of this page

Dexmedetomidine (Dex) Group Sedation

An ICU Sedation Study

TARGET: most awake and comfortable state considered safe (the least awake target will be "briefly wakens with eye contact to voice")

- Primary sedative agent is **DEXMEDETOMIDINE** (diluted with 5% glucose or 0.9% NaCl solution to a concentration of **8 micrograms per ml**)
- **Aim to reduce/stop propofol infusion.**
- All patients should receive opioid infusions for analgesia as clinically indicated at the discretion of clinical teams.
- **NB Dexmedetomidine has analgesic properties, so it may be possible to reduce opioids if pain is well controlled.**

Drugs you should not give:

- **Clonidine should <u>not</u> be used as first line sedation during the intervention period.**

How to manage agitation (RASS +2 TO +4)

- Maintain patient/staff safety by bolusing propofol if needed.
- Once agitation is under control, try to identify and manage the cause, using local unit policy for the management of pain/delirium/anxiety/withdrawal etc.
- Anticipate and avoid agitation; use opioid analgesia in advance of uncomfortable or painful interventions or procedures.

What to do if my patient develops severe bradycardia (HR<50 beats per minute)

- **If your patient's heart rate decreases to less than 50 beats per minute, dexmedetomidine should be temporarily stopped.**
- NB Dexmedetomidine's effect on heart rate can take several hours to resolve, so stopping the dexmedetomidine infusion may not immediately resolve bradycardia.
- Seek advice from medical staff who can review and prescribe, glycopyrronium, atropine, dobutamine, adrenaline or other agents, as per your ICU policy.
- Once heart rate is maintained above 50 beats per minute, dexmedetomidine should be re-commenced at a dose appropriate to the sedation target, but caution should be used when the clinical target is deep sedation.

What to do if my patient becomes hypotensive

- Hypotension should be treated as per local unit policy. Continuous fluid infusions, fluid challenges and vasopressor infusions are all permitted for patients in the A2B Trial.
- If changes to sedation are required as a result of hypotension, propofol should be decreased before dexmedetomidine, unless clinically contraindicated (e.g. if patient requires propofol for neuromuscular blockade).
- Continue dexmedetomidine infusion unless causing haemodynamic compromise, such that target MAP cannot be maintained with 0.15 micrograms/kg/min of noradrenaline. If haemodynamically compromised, consider halving the rate of the dexmedetomidine infusion, then halving again, or stopping, as needed. Dexmedetomidine can be restarted/increased once the patient is more stable, at the discretion of medical staff.
- NB Dexmedetomidine's effect on heart rate can take several hours to resolve, so stopping the dexmedetomidine infusion may not immediately resolve hypotension.

What if my patient requires deep sedation (RASS -3 TO -5) e.g. for neuromuscular blockade (muscle relaxant)?

- If medical staff have asked to keep your patient deeply sedated, please record the reason deep sedation was requested on the A2B Shift Form.
- **Patients who require neuromuscular blockade after randomisation must receive adequate sedation as per standard care at the discretion of the treating clinician, to prevent awareness during paralysis. Dexmedetomidine alone may not provide adequate sedation to prevent awareness.**
- It is suggested that dexmedetomidine is titrated up to the maximum tolerated dose, but if additional sedative drugs are needed to achieve target deep sedation this can be achieved with propofol or benzodiazepine.
- When deep sedation is no longer requested by the caring clinician, aim to use dexmedetomidine as main sedative agent as per flowchart over page. It is suggested that any propofol or benzodiazepine sedation is decreased and stopped prior to reducing the dose of dexmedetomidine.

What if my patient needs an operative procedure/CT scan/MRI etc.?

- Increase sedation for transfer if needed, using dexmedetomidine and/or propofol.
- If required, anaesthesia should be administered as per local perioperative guidelines.
- Continue dexmedetomidine infusion unless haemodynamically compromised, in which case consider halving infusion rate and halving again or stopping, as needed.
- Following the procedure, again aim to reduce propofol and use dexmedetomidine as main sedative agent as per flowchart over page.

Weaning and discontinuing Dexmedetomidine

- **Dexmedetomidine can be abruptly discontinued, but slower weaning should be used if withdrawal reactions occur (rebound hypertension or tachycardia, agitation, sweating etc.). However, this should not delay weaning from ventilation as dexmedetomidine is relatively free from respiratory depressive effects, so can be safely continued post-extubation.**

PTO for Dexmedetomidine Group Sedation Flowchart on reverse of this page

Usual Care (propofol) flowchart

An ICU Sedation Study

Usual Care Group Sedation Flowchart

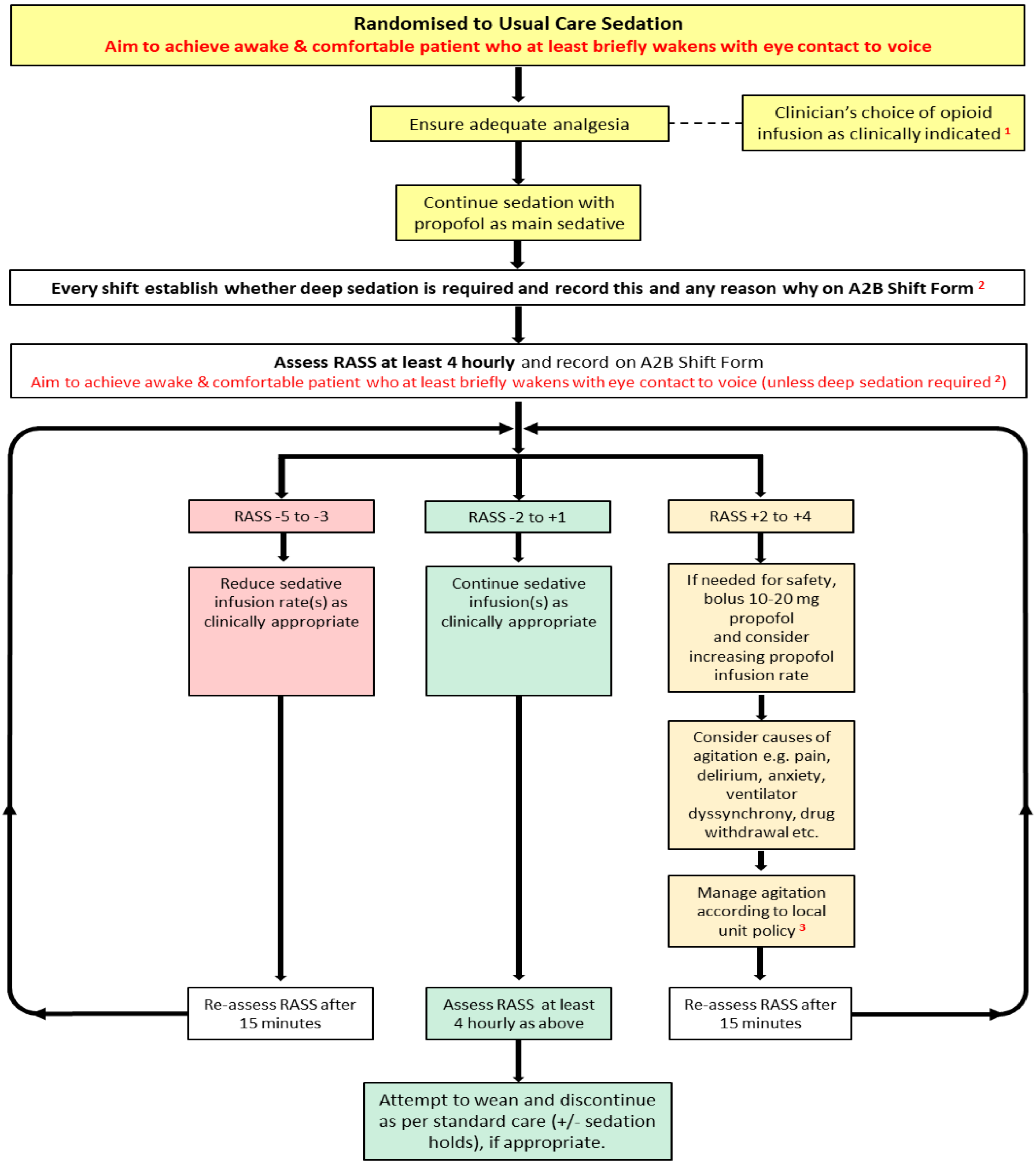

[1] additional opioid boluses can be given as required
[2] PTO for deep sedation advice on the reverse of this page
[3] See advice in "Drugs you should not give" on the reverse of this page before using Dexmedetomidine or Clonidine

An ICU Sedation Study

Usual Care Group Sedation

An ICU Sedation Study

TARGET: most awake and comfortable state considered safe (the least awake target will be "briefly wakens with eye contact to voice")

- Primary sedative agent is **PROPOFOL** (either **1% or 2%**).
- All patients should receive opioid infusions for analgesia as clinically indicated at the discretion of clinical teams

Drugs you should not give:

- **Neither clonidine nor dexmedetomidine should be used as first line sedation during the intervention period.**
- However, either clonidine or dexmedetomidine can be used at the discretion of the clinical teams for specific indications such as agitation, severe delirium or during difficult weaning.

How to manage agitation (RASS +2 TO +4)

- Maintain patient/staff safety by bolusing propofol if needed.
- Once agitation is under control, try to identify and manage the cause, using local unit policy for the management of pain/delirium/anxiety/withdrawal etc.
- Anticipate and avoid agitation; use opioid analgesia in advance of uncomfortable or painful interventions or procedures.

What to do if my patient develops severe bradycardia (HR<50/min)

- Seek advice from medical staff who can review and prescribe, glycopyrronium, atropine, dobutamine, adrenaline or other agents, as per your ICU policy.

What to do if my patient becomes hypotensive

- Hypotension should be treated as per local unit policy. Continuous fluid infusions, fluid challenges and vasopressor infusions are all permitted for patients in the A2B Trial.

What if my patient requires deep sedation (RASS -3 TO -5) e.g. for neuromuscular blockade (muscle relaxant)?

- If medical staff have asked to keep your patient deeply sedated, please record the reason deep sedation was requested on the A2B Shift Form.
- **Patients who require neuromuscular blockade after randomisation must receive adequate sedation as per standard care at the discretion of the treating clinician, to prevent awareness during paralysis.**
- If additional sedative drugs are needed to achieve target deep sedation this can be achieved with benzodiazepine.
- When deep sedation is no longer requested by the caring clinician, aim to use propofol as main sedative agent as per flowchart over page.

What if my patient needs an operative procedure?

- Increase sedation for transfer if needed.
- If required, anaesthesia should be administered as per local perioperative guidelines.
- Following the operative procedure, again aim to use propofol as main sedative agent as per flowchart over page.

What if my patient needs an operative procedure/CT scan/MRI etc.?

- Increase sedation for transfer to theatre if needed using propofol.
- If required, anaesthesia should be administered as per local perioperative guidelines.

PTO for Usual Care Group Sedation Flowchart on reverse of this page

Trial Estimand (see also Statistical Analysis Plan)

Here we define the estimand for the primary analysis of the primary outcome in the trial, in line with the draft addendum to ICH E9 (Statistical Principles for Clinical Trials): ICH E9(R1), Defining the Appropriate Estimand for a Clinical Trial/Sensitivity Analyses.

Population Adult ICU patients enrolled within 48h of MV starting in ICU and expected to require sedation with propofol and MV for at least 48h, at least 24h of which would be after randomisation. Long-term home ventilation, terminal illness, selected diagnoses, allergy to study medication, pregnancy and expected death within 24h are exclusion criteria.

Variable Time to successful extubation post-randomisation (hours).

Population-level Summary Cumulative incidence function of time to extubation; sub-distribution hazard ratio (HR)

The following Intercurrent Events have been identified which would prevent measurement of the primary outcome or change the interpretation of the measured primary outcome:

1. Death before the time point at which randomised treatment is due to start.

2. (a) Dexmedetomidine allocated in randomisation but not started

(b) Clonidine allocated in randomisation but not started

3. Additional propofol being administered when cardiovascular side effects have limited the escalation of dexmedetomidine or clonidine.

4. Additional propofol being administered when non-cardiovascular side effects have limited the escalation of dexmedetomidine or clonidine.

5. Death before successful extubation.

6. Patient withdrawal from intervention and follow-up (situation where deferred consent is not granted is a subset of such events).

7. Transfer to another ICU before successful extubation.

8. Use of dexmedetomidine as main sedative in usual care group.

9. Use of clonidine as main sedative in usual care group.

10. Use of rescue medication in the presence of agitation or delirium.

Events 1, 2(a), 2(b), 8 and 9 are expected to be rare and no specific actions will be taken: analysis of these events will be by intention to treat, except for event 1 which will be handled in the same way as event 5.

Walsh TS, *et al. BMJ Open* 2023; 13:e078645. doi: 10.1136/bmjopen-2023-078645

Events 3 and 4 will be dealt with using an intention to treat approach. Non-cardiovascular side-effects will mostly be sedation-related and therefore will be further analysed as secondary outcomes.

Event 5 will be treated as a competing risk for the primary outcome, and will therefore be analysed using a hypothetical strategy.

Event 6 will also be handled using a hypothetical strategy, in which the time to extubation will be censored at the point of withdrawal and the withdrawals will be assumed to lead to missing at random (MAR) data on the primary outcome. Complete follow up should still be possible for most participants in whom event 7 occurs; if not, the hypothetical strategy used for event 6 will also be implemented.

Event 10 is analogous to events 8 and 9 but applies to all treatment arms and medications. An intention to treat approach will be used for this event.

Full details of the methods of dealing with the above intercurrent events will be incorporated in the statistical analysis plan.

Walsh TS, *et al. BMJ Open* 2023; 13:e078645. doi: 10.1136/bmjopen-2023-078645

Health Economic Evaluation

Overview

The significant cost differences between dexmedetomidine and both usual care and clonidine make the health economic evaluation especially relevant. Of importance, the cost of dexmedetomidine has decreased substantially during the conduct of the trial, such that the cost-effectiveness of the interventions may be different in the context of pricing in the 'post trial' era. Additional drug costs associated with α2-agonists should be balanced against potential cost savings from reductions in ICU and hospital stay and altered outcomes. We will undertake a detailed analysis of the cost and cost-effectiveness of dexmedetomidine, clonidine and usual care. Our analysis will conform to accepted economic evaluation methods in the UK. The comparisons made in the economic evaluation will reflect those of the staged hypothesis tests for the primary outcome (figure 1). We will estimate costs and cost-effectiveness for the 'within-trial' period (6 months/short-run model) and also over the expected lifetime of the patient ('lifetime'/long-run model). Costs will be assessed from the perspective of the NHS and personal social services (PSS). For both the within-trial and lifetime analyses we will undertake cost-utility analyses, estimating incremental cost per quality-adjusted life year (QALY) gained.

Within-trial analysis

For the within-trial analysis we will calculate detailed cost information on index hospitalisation for every patient from ICU admission to hospital discharge and during 6 months follow-up. Patient-level resource use data will be collected on items including: sedative drugs; costs associated with managing adverse events (e.g., delirium); length of ICU stay and hospital wards; use of MV; and co-prescribed medications. Patient-level resource use data will be collected post-discharge up to 6 months using questionnaires at 3 and 6 month follow-up: hospital re-admissions; A&E and outpatient visits; GP and nurse visits in clinic and at home; medications; care home admissions; any other contacts with primary and social care (e.g., physiotherapist, occupational therapist, social worker, counsellor). Unit costs will be obtained from published sources and inflated where appropriate before being applied to the volume of resource use data.

QALYs will be calculated based on the HRQoL and mortality data collected during the trial. HRQoL will be measured using the EQ-5D-5L (www.euroqol.org), which we will collect at 30 days, 3 and 6 months (see table 1). As patients recruited to the trial will be critically ill, completion of the EQ-5D-5L at baseline will not be possible. Previous studies have assumed a common baseline value for all patients (e.g., zero). We will use this approach and two alternatives. First, we will estimate baseline utility scores for patients using proxy responses from a person who knows the patient. In this case we will use the proxy version of the EQ-5D-5L (by the patient's spouse, parent or (adult) child). The type of proxy respondent will be controlled for in subsequent analyses. Second, we will ask patients to retrospectively record their baseline EQ-5D-5L at the 30 day follow-up point. We will evaluate QALYs associated with each strategy using all three approaches; the base case approach will use the proxy responses. Patients who die will be assigned a utility value of zero at the date of death and all subsequent time periods. Patient-specific utility profiles will be constructed assuming a

straight line relation between each of the patients' EQ-5D-5L scores at each follow-up point. The QALYs experienced by each patient from baseline to 6 months will be calculated as the area underneath this profile.

Multiple imputation by chained equations will be used to deal with missing HRQoL and resource use values. Subsequent analyses of imputed data will include variance correction factors to account for additional variability introduced into parameter values as a result of the imputation process. Cost-effectiveness will be calculated as the mean cost difference between groups divided by the mean difference in outcomes (QALYs) to give the incremental cost-effectiveness ratio (ICER). We will also calculate net monetary benefits (NMBs). We will subject the results to extensive deterministic (one-, two- and multi-way) and probabilistic sensitivity analysis. For the latter, non-parametric methods for calculating confidence intervals around the ICERs and NMBs based on bootstrapped estimates of the mean cost and QALY differences will be used. These methods will appropriately account for the multiple imputation of the missing data. The bootstrap replications will also be used to construct cost-effectiveness acceptability curves, which will show the probability that each strategy is cost-effective at 6 months for different values willingness to pay for additional QALYs by the NHS.

Lifetime analysis

In the lifetime model cost-effectiveness will be calculated in terms of the incremental cost per QALY gained. A review of the NIHR HTA website (www.hta.ac.uk/project/htapubs.asp) and the NHS Economic Evaluation Database (NHS-EED, www.crd.york.ac.uk https://www.crd.york.ac.uk/CRDWeb/) (last search 15/05/2017) reveals there have been no previous analyses to evaluate lifetime cost-effectiveness of the study strategies. Given this paucity of evidence, we will develop a de novo cost-effectiveness model that will be populated based on available evidence, including the data collected during the trial. We will: [1] design a lifetime model to characterize health states of ICU survivors; [2] populate the model using data identified from the trial and published literature and routine sources; [3] relate outcomes from the trial to final outcomes, expressed in terms of QALYs; and [4] identify which parameters in the model are most uncertain and are important drivers of cost-effectiveness. The model is likely to use a similar structure to a previous economic evaluation of long-term cost-effectiveness for ICU patients in the UK. Survival analysis of the RCT data will provide the basis for extrapolating any within-trial differences in costs and QALYs. The model will use external data on long-term survival of ICU survivors, including from co-applicants expert in this area (Lone, Walsh). Specific details of the data to be used to populate the model will be determined following the development of the structure and the systematic searches of the literature to identify existing models. We will undertake deterministic (one-, two- and multi-way) and probabilistic sensitivity analysis, the latter assuming appropriate distributions and parameter values. We will combine data on incremental costs with epidemiological data on projected patient numbers and undertake a budget impact analysis to evaluate what the total cost impact of each strategy would be were it to be scaled up; budget impact will be calculated separately for ICU-related costs only, the within-trial period and using a lifetime time horizon, as each might be appropriate

for different decision-makers. We will also use the probabilistic sensitivity analyses combined with the epidemiological information on projected patient numbers to undertake a value of information analysis to evaluate the potential economic value of future research on this topic.

Walsh TS, *et al. BMJ Open* 2023; 13:e078645. doi: 10.1136/bmjopen-2023-078645
