# Supplementary material for: Alpha 2 agonists for sedation to produce better outcomes from critical illness (A2B Trial): protocol for a multicentre phase 3 pragmatic clinical and cost-effectiveness randomised trial in the UK
Source: BMJ Open. 2023 Dec 9;13(12):e078645. doi: 10.1136/bmjopen-2023-078645 (PMC10729141; doi:10.1136/bmjopen-2023-078645)
Supplement: Supplementary data [file bmjopen-2023-078645supp002.pdf]

Statistical Analysis Plan	A2B
Version No	2.0
Date Finalised	dd/mm/yyyy

An ICU Sedation Study

Alpha 2 agonists for sedation to produce better outcomes from critical illness (A2B Trial): A randomised, parallel-group, allocation concealed, controlled, open, phase 3 pragmatic clinical and cost- effectiveness trial with internal pilot

A2B Trial

Statistical Analysis Plan (Version as at 28th July 2023)

CONFIDENTIAL

**Version No**	2.0
**Date Finalised**	dd/mm/yyyy
**Author(s)**	Richard Parker (until unblinded on 2 August 2019)<br>Christopher Weir (from 2 August 2019)
**CI Name**	Professor Timothy Walsh
**CI Email address**	timothy.walsh@ed.ac.uk

**Funder**	NIHR Health Technology Assessment
**Funding Reference Number**	HTA 16/93/01
**Sponsor**	The University of Edinburgh & Lothian Health Board ACCORD
**EudraCT Number**	2018-001650-98
**ClinicalTrials.gov**	NCT03653832

Signatures	
**Trial Statistician: Prof Christopher Weir**	**Date:**
**Chief Investigator: Prof Timothy Walsh**	**Date:**

**Document Control**		
**Version No**	**Date**	**Summary of Revisions**
1.0	22/01/2021	Initial Creation
2.0	dd/mm/yyyy	Incorporated modified sample size calculation. Updated to reflect latest ECTU SAP template (V4.0, 25Mar2021).

Statistical Analysis Plan	A2B
Version No	2.0
Date Finalised	dd/mm/yyyy

Table of Contents

List of Abbreviations ... 3
1. Introduction ... 4
2. Statistical Methods section from the protocol ... 4
8.2 PROPOSED ANALYSES ... 4
8.2.1 Estimand ... 4
8.2.2 Statistical analysis ... 5
3. Overall Statistical Principles ... 7
3.1 Analysis populations ... 7
3.2 Outcomes ... 8
4. List of Analyses ... 9
4.1 Recruitment, retention and missing data ... 10
4.2 Baseline characteristics ... 10
4.3 Primary outcome (primary analysis) ... 11
4.4 Primary outcome (supplementary analyses) ... 12
4.5 Subgroup analyses ... 13
4.6 Secondary outcomes ... 13
4.6.1 Missing data handling: secondary outcomes ... 15
4.7 Safety ... 15
4.8 Concomitant medications ... 16
4.9 Intervention dose, fidelity and reach ... 16
4.10 Protocol deviations and violations ... 16
5. Validation and QC ... 16
6. Data sharing ... 17
7. References ... 17
Appendix 1 Sedation Quality Assessment Tool (SQAT) ... 19
Appendix 2 PRE-DELIRIC score derivation ... 20
Appendix 3 Data completeness and intervention adherence ... 22

Statistical Analysis Plan	A2B
Version No	2.0
Date Finalised	dd/mm/yyyy

List of Abbreviations

Abbreviation	Full name
AE	Adverse event
CAM-ICU	Confusion-Assessment Method for ICU
CI	Confidence interval
CONSORT	CONsolidated Standards Of Reporting Trials
CPAP	Continuous positive airway pressure
CRF	Case report form
EQ-5D-5L	EuroQol instrument with five levels of severity in each of five dimensions
EudraCT	European Clinical Trials Database
HADS	Hospital Anxiety and Depression Scale
HR	Hazard ratio
HTA	Health Technology Assessment
ICE-Q	Intensive Care Experience Questionnaire
ICU	Intensive care unit
ICH	International Council for Harmonisation of Technical Requirements for Pharmaceuticals for Human Use
IES-R	Impact of Events Scale – Revised
MV	Mechanical ventilation
NIHR	National Institute for Health Research
NIV	Non-invasive mechanical ventilation
OR	Odds ratio
RASS	Richmond Agitation and Sedation Scale
SD	Standard deviation
SOFA	Sequential Organ Failure Assessment
SQAT	Sedation Quality Assessment Tool
T-MoCA	Montreal Cognitive Assessment tool (telephone version)

Walsh TS, *et al. BMJ Open* 2023; 13:e078645. doi: 10.1136/bmjopen-2023-078645

Statistical Analysis Plan	A2B
Version No	2.0
Date Finalised	dd/mm/yyyy

1. Introduction

A2B is a randomised, parallel-group, allocation concealed, controlled, open, multi-centre, phase 3 pragmatic clinical and cost- effectiveness trial with internal pilot. Adult intensive care unit (ICU) patients expected to require at least 24 hours further mechanical ventilation (MV) will be randomised within 48 hours of starting MV. Patients with primary brain injury; post-cardiac arrest; status epilepticus; and peripheral nervous system disease will be excluded. 1437 patients will be randomised to receive sedation using dexmedetomidine or clonidine or 'usual care' sedation in a 1:1:1 ratio. To simplify the enrolment process randomisation will be stratified by site alone.

This statistical analysis plan is written with reference to protocol version 7, dated 25 April 2023. Its scope covers the end of trial analysis for A2B, with the exception of the health economic evaluation, the process evaluation (apart from quantitative descriptions of fidelity to the intervention) and the mechanistic sub-study of pro- and anti-inflammatory mediators which will all be documented separately.

2. Statistical Methods section from the protocol

8.2 PROPOSED ANALYSES

8.2.1 Estimand

Here we define the estimand for the primary analysis of the primary outcome in the trial, in line with the draft addendum to ICH E9 (Statistical Principles for Clinical Trials): ICH E9(R1), Defining the Appropriate Estimand for a Clinical Trial/Sensitivity Analyses.

Population *Adult ICU patients enrolled within 48h of MV starting in ICU and expected to require sedation with propofol and MV for at least 48h, at least 24h of which would be after randomisation. Long-term home ventilation, terminal illness, selected diagnoses, allergy to study medication, pregnancy and expected death within 24h are exclusion criteria.*

Variable *Time to successful extubation post-randomisation (hours).*

Population-level Summary *Cumulative incidence function of time to extubation; sub-distribution hazard ratio (HR)*

The following ***Intercurrent Events*** *have been identified which would prevent measurement of the primary outcome or change the interpretation of the measured primary outcome:*

1. *Death before the time point at which randomised treatment is due to start.*
2. *(a) Dexmedetomidine allocated in randomisation but not started*
   (b) Clonidine allocated in randomisation but not started
3. *Additional propofol being administered when cardiovascular side effects have limited the escalation of dexmedetomidine or clonidine.*
4. *Additional propofol being administered when non-cardiovascular side effects have limited the escalation of dexmedetomidine or clonidine.*
5. *Death before successful extubation.*

Walsh TS, *et al. BMJ Open* 2023; 13:e078645. doi: 10.1136/bmjopen-2023-078645

6. *Patient withdrawal from intervention and follow-up (situation where deferred consent is not granted is a subset of such events).*
7. *Transfer to another ICU before successful extubation.*
8. *Use of dexmedetomidine as main sedative in usual care group.*
9. *Use of clonidine as main sedative in usual care group.*
10. *Use of rescue medication [1]in the presence of agitation or delirium.*

Events 1, 2(a), 2(b), 8 and 9 are expected to be rare and no specific actions will be taken: analysis of these events will be by intention to treat, except for event 1 which will be handled in the same way as event 5.

Events 3 and 4 will be dealt with using an intention to treat approach. Non-cardiovascular side-effects will mostly be sedation-related and therefore will be further analysed as secondary outcomes.

Event 5 will be treated as a competing risk for the primary outcome, and will therefore be analysed using a hypothetical strategy.

Event 6 will also be handled using a hypothetical strategy, in which the time to extubation will be censored at the point of withdrawal and the withdrawals will be assumed to lead to missing at random (MAR) data on the primary outcome. Complete follow up should still be possible for most participants in whom event 7 occurs; if not, the hypothetical strategy used for event 6 will also be implemented.

Event 10 is analogous to events 8 and 9 but applies to all treatment arms and medications. An intention to treat approach will be used for this event.

Full details of the methods of dealing with the above intercurrent events will be incorporated in the statistical analysis plan.

8.2.2 Statistical analysis

For the primary analysis, a Fine and Gray proportional sub-distribution hazards regression analysis of time from randomisation to successful extubation will be performed (this method allows us to directly model the cumulative incidence of extubation after taking into account the competing risk of mortality) for each hypothesis test permitted under the analytic structure (Figure 1). Results will be expressed as sub-distribution HRs with corresponding 95% confidence intervals and p-values.

The following supplementary analyses will be performed to provide reassurance about the robustness of the main analysis of the primary outcome, for each between-arm comparison:

(i) *A Cox frailty proportional hazards regression model will be fitted to the primary outcome, with censoring for deaths or loss to follow-up in ICU while on MV. Although death in ICU may be considered a competing risk, this modelling approach allows us to estimate the instantaneous risk of experiencing a successful extubation event at time t given that the patient is still alive at time t (in the literature this is called the "cause-specific hazard" of extubation for patients who have not yet died). Site will be included in the model as a random effect.*

[1] Rescue medication is recorded as haloperidol, quetiapine, dexmedetomidine, midazolam, olanzapine, clonidine, lorazepam or other

(ii) *A Cox frailty regression analysis of time from randomisation to ICU mortality while on ventilation. Patients experiencing successful extubation events or loss to follow-up prior to mortality will be censored. This analysis will provide "cause-specific" HRs for patients on MV to support the primary analysis results. Site will be included in the model as a random effect.*

(iii) *A Cox frailty regression analysis of time to all-cause mortality, with censoring only for patients lost to follow-up during the six months follow-up period. This analysis will allow us to compare the risk of overall mortality across trial arms for all patients, regardless of whether or not patients are still on MV. Site will be included in the model as a random effect.*

(iv) *For each participant, the proportion of care periods will be recorded in which propofol treatment was maintained even though dexmedetomidine or clonidine had not been up-titrated to its maximum dose and had no dose-limiting side-effects. As an exploratory analysis, the main analysis of the primary outcome will be repeated using the adherence analysis set (section 8.2.3) rather than the full analysis set.*

For the secondary outcomes other than mortality, formal hypothesis testing will not be performed but point estimates and 95% confidence intervals for pairwise differences between randomised groups will be calculated. A trial analysis plan providing full details will be finalised prior to locking the trial data base.

The hierarchical hypothesis testing framework for analysis of the primary outcome, which controls the overall type I error to be at most 6.5% across the multiple analyses being performed, is also outlined in protocol Figure 1:

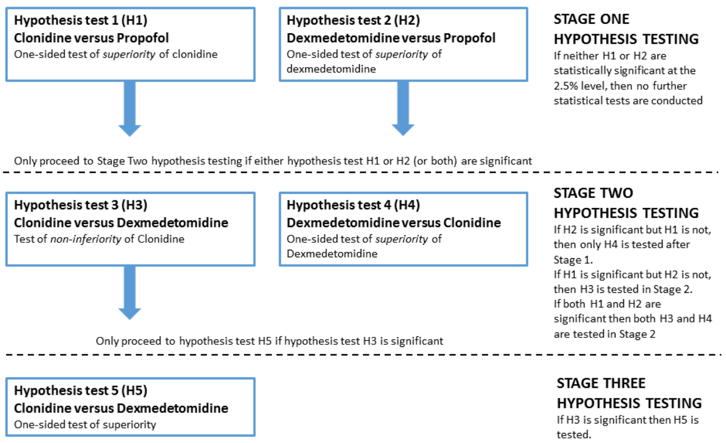

Figure 1: Analytic framework which efficiently tests the trial questions using a hierarchical analytic structure with serial gatekeeping to preserve study power. Hypothesis tests will be performed using a 2.5% one-sided significance level, with the exception of the non-inferiority test H3 which will use a 4% one-sided significance level.

3. Overall Statistical Principles

The Stage 1 hypothesis testing of the superiority of each of clonidine and dexmedetomidine versus propofol will be carried out at the one-sided 2.5% significance level. The Stage 2 hypothesis of non-inferiority of clonidine to dexmedetomidine will be performed with a one-sided 4% significance level. The Stage 2 hypothesis of superiority of dexmedetomidine to clonidine will have a one-sided 2.5% significance level. Finally, in Stage 3, there will be a possible test of superiority of clonidine versus dexmedetomidine at the one-sided 2.5% significance level. All hypothesis tests on the primary outcome are arranged in a hierarchical structure, with serial gatekeeping, to ensure overall control of the type 1 error to at most 6.5%.

Categorical variables will be summarised using frequencies and percentages; continuous variables will be summarised using the mean, standard deviation (SD), median, lower quartile, upper quartile, minimum and maximum values.

Analyses of outcomes will adjust for site as a random effect, since site is included as a stratification factor in the randomisation.

Generally speaking, missing data will be handled according to the principles outlined in the A2B estimand, described in protocol section 8.2.1. Participants randomised in error despite ineligibility, becoming ineligible before drug administered, or being withdrawn from the trial by family members prior to intervention, will be reported in the participant flow summary but will not be included in efficacy or safety analyses as no further data will be gathered on these participants.

Outliers will be identified by viewing boxplots of the outcome variables of interest. All analyses will include outliers as standard; where data are present which lie more than 4 standard deviations away from the mean, a sensitivity analysis will be performed removing these data values to determine the robustness of the findings in the analysis where outliers were included.

The planned analyses will be performed using the SAS statistical software, version 9.4 or later. Following the end of trial, defined as the date of the last follow-up of the final participant, the planned analyses will be performed once data querying has been completed and the locking of the trial database has been documented.

3.1 Analysis populations

Full analysis set
All participants randomised, analysed according to their allocated treatment group regardless of the treatment actually received.

Adherence analysis set
The **adherence analysis set** will be all randomised participants in the **full analysis set** who, in the dexmedetomidine group received any dexmedetomidine on the day of randomisation; in the clonidine

group received any clonidine on the day of randomisation; or in the usual care group received neither dexmedetomidine nor clonidine (except as rescue medication for agitation) on the day of randomisation.

3.2 Outcomes

Primary outcome

- Time to successful extubation post-randomisation (hours).

A successful first extubation from mechanical ventilation will be defined as follows:

a) From endotracheal extubation: time of first extubation that is followed by 48 hours of spontaneous breathing.

b) From tracheostomy: time of extubation will be defined as the start time of the first period during which a patient receives support not exceeding 5 cmH_2O CPAP with less or equal to pressure support ventilation of $5cmH_2O$ for a continuous period of 48 hours. Decannulation during this period will count as being free from mechanical ventilation.

c) From non-invasive mechanical ventilation (NIV): time of extubation will be the start time of the first period during which a patient receives support not exceeding 5 cmH_2O CPAP via mask/hood for a continuous period of 48 hours. NIV patients receiving any pressure supported breaths will not be considered to be spontaneously breathing unassisted.

NB: The use of high flow nasal oxygen will not be counted as mechanical ventilation, so a patient on high nasal flow oxygen alone will be considered to be spontaneously breathing unassisted.

Secondary outcomes

Secondary outcomes are listed in priority order. Specifically, mortality forms a component of the primary outcome time to successful extubation. Outcomes listed from Length of ICU Stay to Patient Experience of ICU Care are outcomes specified in the NIHR HTA briefing document for this commissioned funding call. The remaining outcomes are listed in order of priority according to guidance from patient and public involvement representatives.

S1 Mortality
ICU; hospital; 30 days; 90 days; 180 days post-randomisation

S2 Length of ICU stay (days from randomisation to ICU discharge)

S3 Sedation quality, measured by Richmond Agitation and Sedation Scale (RASS)
- Measured four-hourly during mechanical ventilation until primary outcome recorded, summarised as lowest and highest day shift and night shift RASS scores over time

S4 Sedation quality, measured during mechanical ventilation until primary outcome recorded by Sedation Quality Assessment Tool (SQAT- Appendix 1)
Four sedation quality states:
1. Overall optimum sedation (no agitation;no unnecessary deep sedation;no pain behaviour)
2. Agitation
3. Unnecessary deep sedation (RASS -4/-5 without clinical indication)
4. Pain (presence of pain behaviour based on limb movement and ventilation compliance)

S5 Time to first optimum sedation

- Hours from randomisation to first RASS score of -2 or greater
- Days from randomisation to first day with SQAT optimum sedation

S6 Delirium prior to successful extubation, assessed by Confusion-Assessment Method for ICU (CAM-ICU)
- Occurrence prior to successful extubation (binary outcome)
- Days with delirium or coma prior to successful extubation (continuous outcome)

S7 One or more pre-defined cardiac adverse events
(of those recorded daily: severe bradycardia; cardiac arrhythmias; cardiac arrest)

S8 Health-related Quality of Life, measured by recall prior to hospital admission, and at 30, 90 and 180 days after randomisation using the EuroQol EQ-5D-5L instrument

S9 Patient Ability to Communicate Pain and Ability to Cooperate with Care
Binary assessments for each 12 hours nursing shift:
- Was patient able to communicate pain?
- Was patient able to cooperate with care?

S10 Patient experience of ICU care, measured at 90 days after randomisation using the Intensive Care Experience Questionnaire (ICE-Q)
Provides numeric score in four domains:

1. Awareness of Surroundings (9 items; score range 9-45)
2. Frightening Experiences (6 items; score range 6-35)
3. Recall of Experiences (5 items; score range 5-25)
4. Satisfaction with Care (4 items; score range 4-20)

S11 Relative/partner/friend (PerLR) assessment of comfort and communication, measured daily during mechanical ventilation
Binary assessment for each question:

1. Does the patient appear awake to the visitor?
2. Does the patient seem comfortable to the visitor?
3. Does the visitor feel they can communicate with the patient?

S12 Anxiety and depression, measured at 180 days post randomisation using the Hospital Anxiety and Depression Scale (HADS) questionnaire

S13 Post-traumatic stress, measured at 180 days post randomisation using the Impact of Events Scale-revised (IES-R)

S14 Cognitive function, measured at 180 days post randomisation using the Montreal Cognitive Assessment tool telephone version (T-MoCA)

Commented [CW1]: Postal version no longer mentioned in protocol

4. List of Analyses

This analysis plan describes the end of trial statistical analyses to be performed on A2B, excluding analysis of the mechanistic sub-study of putative pro- and anti-inflammatory mediators (protocol

section 11), the health economics analyses and the process evaluation components of the trial. However, quantitative assessment of fidelity from the process evaluation is included in the scope of this analysis plan.

4.1 Recruitment, retention and missing data

A CONSORT flow diagram will be constructed. For EudraCT reporting purposes, enrolment will also be summarised into age categories 18-64; 65-84; and 85+ years.

The number and percentage of patients who were later found to be ineligible for the trial even though they were randomised will be summarised by randomised group, as will the number of patients formally withdrawn and the reason for withdrawal (if available). The number and percentage of patients with missing primary outcome data will be reported by randomised treatment allocation. No formal statistical testing will be performed.

4.2 Baseline characteristics

The following baseline characteristics will be summarised by treatment group and overall. A further descriptive summary will assess any association between the Covid-19 pandemic and participant characteristics. The baseline characteristics summary will be further stratified by randomisations occurring up to and including 23 March 2020 and those occurring after 23 March 2020.

Age (years)
Age (by EudraCT reporting categories)
Gender
Pre-randomisation:
- Estimated weight (kg)
- RASS
- CAM-ICU (unless RASS -4 or -5, or is -3 but the assessor is unable to assess CAM-ICU status)

Functional comorbidity index (Groll et al, 2005) (total count; and 18 separate items)
Medical history:
- Portal hypertension
- Biopsy proven cirrhosis
- Hepatic encephalopathy
- Alcohol dependence
- Drug dependence

Type of admission (Trauma, Non-trauma medical, Non-trauma surgical; Planned, Unplanned)
Diagnosis at admission (Medical)
Diagnosis at admission (Surgical)
Pre-randomisation sedatives (Propofol, Midazolam, Fentanyl, Alfentanil, Morphine, Remifentanil, Dexmedetomidine, Clonidine, Haloperidol, Diazepam, Other (free text)) For each report frequency and summarise dose, in units specified on CRF.
SOFA score (excluding neurological SOFA) (Singer et al, 2016)
Pre-randomisation blood results:
- Haemoglobin g/L
- Lymphocytes x10^9/L
- Sodium mmol/L
- Urea mmol/L
- Albumin g/L
- White cell count x10^9/L
- APTT ratio

Potassium mmol/L
eGFR mL/min/1.73m^2
ALT U/L

Blood gases:

H^+
pH
PaO_2 kPa
$PaCO_2$ kPa
Standard bicarbonate mmol/L
Lactate mmol/L

PRE-DELIRIC delirium prediction score (van den Boogaard et al, 2012; Appendix 2) including components:

Apache II score
Infection/sepsis
Antibiotics given during first 24 hours in ICU
Sepsis
Septic shock
Coma RASS -4/-5 for at least 8 hours in first 24 hours in ICU
If yes, by use of medication / other reason / both medication and other
Total morphine dose in first 24 hours in ICU
None / 0.01-7.1mg / 7.2-18.6mg / 18.7-331.6mg
Any propofol, midazolam or lorazepam use in first 24 hours in ICU
Highest urea value in first 24 hours in ICU (mmol/L)
Metabolic acidosis

Proxy baseline EQ-5D

4.3 Primary outcome (primary analysis)

For the primary analysis, performed on the full analysis set, a Fine and Gray proportional sub-distribution hazards regression analysis (Fine and Gray, 1999) of time from randomisation to successful extubation will be performed (this method allows us to directly model the cumulative incidence of extubation after taking into account the competing risk of mortality, thus implementing the hypothetical strategy outlined in the estimand for intercurrent events 1 and 5) for each hypothesis test permitted under the hierarchical testing structure. Results will be expressed as the sub-distribution hazard ratio (HR) for each of dexmedetomidine and clonidine versus usual care, with corresponding 95% confidence intervals (CI) and p-values from the Fine-Gray model. The exception will be the non-inferiority analysis of clonidine versus dexmedetomidine (hypothesis H3 in protocol figure 1) for which a 96% one-sided non-inferiority CI will be presented. Site will be accounted for in the analysis by implementing the marginal model approach to the Fine and Gray method for clustered data (Zhou et al, 2012). If this aspect of model fitting proves problematic due to sites which have randomised a small number of participants (fewer than 5), we will consider pooling of data from such sites to address this issue.

Intercurrent events 2(a), 2(b), 8 and 9 are expected to be rare and will therefore be handled using the intention to treat approach in the primary analysis of the primary outcome. Events 3 and 4 (propofol use due to cardiovascular and non-cardiovascular side-effects respectively) will also be handled using the intention to treat approach due the pragmatic exploration of the effects of clonidine and dexmedetomidine in A2B. Withdrawals where the participant has not withdrawn permission to use data collected up to the point of withdrawal will have time to extubation censored at the time of withdrawal (intercurrent event 6, missing at random assumption, hypothetical strategy). In the rare

cases of transfer to another ICU before extubation (intercurrent event 7), follow-up will be continued to extubation where possible but if extubation time is missing it will be censored at the last time at which the extubation status is known (missing at random assumption, hypothetical strategy). Intercurrent event 10 will be handled using intention to treat, again reflecting the treatment policy pragmatic nature of A2B.

The cumulative incidence function (CIF) obtained from the Fine-Gray model for time to successful extubation will be plotted separately for each treatment group; the median time to successful extubation and its 95% CI will be reported by treatment group. As recommended in the CONSORT reporting guidance, the absolute risk difference (and its 95% CI) for each of dexmedetomidine and clonidine versus control will be reported at 7 days after randomisation (the median time on mechanical ventilation under 'usual care' in a real ICU dataset).

Following the strategy recommended by Poythress et al. (2020), the fit of the Fine-Gray model will be evaluated by plotting, by treatment group, the CIF for time to successful extubation from the Fine-Gray model against the nonparametric CIF. If substantial differences occur between the Fine-Gray and nonparametric CIF curves an alternative modelling strategy, such as cause-specific hazards, will be considered.

4.4 Primary outcome (supplementary analyses)

Supplementary analyses will provide reassurance about the robustness of the primary analysis, for each between-arm comparison:

(i) A mixed effects partially proportional hazards regression model will be fitted to the primary outcome of time from randomisation to successful extubation, with censoring for deaths or loss to follow-up in ICU while on MV. Although death in ICU may be considered a competing risk, censoring for deaths allows us to estimate the instantaneous risk of experiencing a successful extubation event at time t given that the patient is still alive at time t (the "cause-specific hazard" of extubation for patients who have not yet died). Site will be included in the model as a random effect, treatment group as a fixed effect. Results will be expressed as the HR for each of dexmedetomidine and clonidine versus usual care, with its corresponding 95% CI and p-value.

(ii) A mixed effects partially proportional hazards regression analysis of time from randomisation to ICU mortality while on MV. Patients experiencing successful extubation events or loss to follow-up prior to mortality will be censored. For patients on MV, this analysis will provide the mortality "cause-specific" HR (and 95% CI) for each of dexmedetomidine and clonidine versus usual care, to support the primary analysis results. Site will be included in the model as a random effect, treatment group as a fixed effect.

(iii) Overall mortality will be analysed using a mixed effects partially proportional hazards regression analysis, see Section 4.6 for details.

(iv) The primary analysis will be repeated, but using the adherence analysis set.

Furthermore, selected baseline characteristics of patients with missing primary outcome data due to withdrawal will be compared descriptively to those with patients who did not withdraw prior to extubation to evaluate the missing at random assumption present in the primary analysis of intercurrent event 6.

Similarly, selected baseline characteristics of patients transferred to another ICU who did not have time to extubation recorded will be compared to those transferred to another ICU who did have it

recorded, to assess the missing at random assumption being made in the primary analysis of intercurrent event 7.

Finally, further exploratory analysis will assess any association between the Covid-19 pandemic and the primary outcome. Summary descriptive statistics of time to successful extubation will be reported by treatment group and further stratified by the date of the UK lockdown: randomisations occurring up to and including 23 March 2020 versus those occurring after 23 March 2020.

4.5 Subgroup analyses

The primary analysis of the primary outcome will be repeated for the following subgroups specified in the protocol.

(1) Patients with and without sepsis at enrolment to A2B.
(2) Patients with lower or higher delirium risk, as defined by the PRE-DELIRIC delirium risk prediction score. (van den Boogaard et al, 2012) The groups with values above (or including) and below the median PRE-DELIRIC score observed in the trial population will be compared.
(3) Patients with and without organ dysfunction at randomisation. The group with SOFA score values above or equal to the median SOFA score (excluding neurological score) that is present at baseline will be compared with the group with SOFA score values below the median score at baseline.
(4) Age (<64 versus ≥64)

For each subgroup variable, a p-value will be calculated for its interaction with each of dexmedetomidine and clonidine versus usual care. Within each subgroup category, we will calculate the sub-distribution HR and 95% confidence interval for (a) dexmedetomidine versus usual care and (b) clonidine versus usual care and present these in a forest plot. These analyses will be considered exploratory.

For age, an additional exploratory analysis will fit an interaction term based on its continuous value rather than age categories. A cubic B-spline, fractional polynomial or simple quadratic term will be fitted to determine, via a likelihood ratio test, whether there is a significant non-linear relationship between age and the effects of each of dexmedetomidine and clonidine versus usual care.

For the age subgroup, given the findings of the SPICE trial of dexmedetomidine (Shehabi et al., 2019), the above subgroup analysis will also be applied to the mortality secondary outcome **S1**.

4.6 Secondary outcomes

Each secondary outcome will be summarised appropriately, by treatment group and overall. Where informative graphical summaries will also be created. The large number of secondary outcomes means that not all will be included in the mean trial publication text. Instead, **S5**, **S9** and **S11** will be reported in the accompanying supplementary material. Other secondary outcomes for which there is substantial missing data will also be considered for transfer to the supplementary material.

For the secondary outcomes other than **S1**, mortality, formal hypothesis testing will not be performed but point estimates and 95% confidence intervals for pairwise differences between randomised groups will be calculated. P-values will not be reported.

For secondary outcomes measured at more than one time point following ICU discharge, separate analyses will be performed for each measurement occasion. Secondary outcomes **S9**, **S10** and **S11** will be summarised descriptively (for **S10,** for each of the four domains separately) without any calculation of confidence intervals for differences between groups.

S1 Mortality. A mixed effects partially proportional hazards regression analysis will be used to analyse time to all-cause mortality, with censoring only for patients lost to follow-up during the six months follow-up period. This analysis will allow us to compare the risk of overall mortality, using the HR, 95% CI and p-value, for each of dexmedetomidine and clonidine versus usual care for all patients, regardless of whether or not patients are still on MV. Site will be included in the model as a random effect and treatment group as a fixed effect.

The time to event secondary outcomes **S2** and **S9** will be analysed using the same method as for the primary analysis of the primary outcome (Section 4.3), in order to take account of the potential competing risk of death. The supplementary analyses of Section 4.4 will also be applied for these outcomes. Time to event outcome **S5** will be summarised descriptively but will not be formally analysed.

Binary secondary outcomes (**S6** [delirium occurrence], **S4**, **S7**, **S9**, **S11**) will be analysed by a generalised linear mixed model with a logit link function. Site will be included as a random effect in the model and treatment group as a fixed effect. For outcomes **S4** and **S9** which are measured in multiple care periods, a random effect for participant (nested within site) will also be included. Optimal sedation for outcome **S4** will be reported descriptively as a proportion for each combination of study day and treatment group. It will not be analysed formally. Each of the **S4** SQAT components (freedom from agitation; freedom from pain; and freedom from unnecessary deep sedation) will be reported descriptively as for optimal sedation and in addition will be analysed using the generalised linear mixed model with logit link. Results will be expressed as the odds ratio (OR) for each of dexmedetomidine and clonidine versus usual care, with corresponding 95% CI.

Continuous secondary outcomes (**S3** [highest RASS score recorded daily, regardless of whether clinical need for deep sedation was recorded], **S8**, **S12**, **S13**, **S14**) will be analysed using a normal linear mixed model. Site will be included as a random effect in the model and treatment group as a fixed effect. Outcome **S3** is measured in each care period so a random effect for participant (nested within site) will also be included. For **S3** each of the day shift and night shift highest and lowest RASS will also be summarised graphically up to the occurrence of the successful extubation primary outcome. A proxy for outcome **S8** is measured at baseline and this will be included as a fixed effect in the model. The parameter to be estimated is the adjusted mean difference: dexmedetomidine minus usual care; and clonidine minus usual care. The corresponding 95% CI will also be reported. If the assumption of normality of residuals does not hold (as determined by normal probability plot), the outcome variable will be transformed to rectify this. In the event that the assumption cannot be satisfied, alternative analyses (for example involving categorising the outcome measure) will be conducted. A similar strategy will be applied when residuals versus fitted values demonstrate non-constant variance for an outcome.

The count variable **S6**, delirium or coma days prior to successful extubation, will be analysed using a generalised linear mixed model with a log link (Poisson regression). Number of days prior to successful extubation will be included as an offset term in the model. Site will be included as a random effect in

the model. The result for each of dexmedetomidine and clonidine versus usual care will be presented as a rate ratio (RR) and 95% confidence interval.

4.6.1 Missing data handling: secondary outcomes

We anticipate minimal rates of missing data for the secondary outcome **S1**, mortality. In cases of missing data, the survival time will be censored at the date last known alive. Missing data on time to event secondary outcomes **S2** and **S9** will be handled using a similar approach to that used for **S1**.

In other secondary outcomes, for which no formal hypothesis testing will be undertaken, the following strategies will be implemented where missing data rates are low (less than 10% overall, and with a no more than 5% difference in the rate across treatment groups). For continuous secondary outcomes a "missing at random" assumption will be applied automatically within the normal linear mixed model, while complete case analyses will be performed for outcomes which are counts or binary variables. In the event of the missing data rate being greater than 10% overall, or differing by more than 5% across treatment groups, multiple imputation strategies will be considered.

4.7 Safety

Safety data will be reported for the full analysis population, according to treatment allocated.

While death will be analysed as a secondary outcome (Section 4.6), only deaths considered related to participation in A2B will be recorded as serious adverse events. Sedation-related adverse events (including hypotension, hypertension, unplanned NG removal, unplanned central line removal, unplanned arterial line removal, unplanned peripheral line removal, unplanned drain removal, unplanned extubation, staff injury as a result of patient, patient injury and ileus) will be reported descriptively: number and percentage by treatment group and overall.

During the recruiting ICU stay (or up to and including study day 28, whichever is earlier) the number and percentage of patients experiencing each of: any adverse event (AE); non-serious adverse event (NSAE); serious adverse event (SAE) and suspected unexpected serious adverse reaction (SUSAR) will be reported, overall and split by trial arm. Tabulations will be split by events occurring pre- and post-randomisation. The numbers of events will also be reported.

The AE, NSAE, SAE and SUSAR tables will also be further categorised by the number and percentage of patients recording an event in each of the MedDRA system organ class categories, with a further sub-categorisation according to verbatim text or MedDRA preferred term as appropriate.

Data listings of all adverse events will be provided by treatment group, according to MedDRA system organ class, verbatim text, severity, seriousness, causality, expectedness and outcome.

Daily data on blood results (platelets, bilirubin, creatinine), respiratory function (FiO_2, PaO_2, SpO_2), blood pressure (lowest systolic BP recorded and corresponding diastolic BP) and urine output (>500mL/day, 200-500mL/day,<200mL/day) will be summarised and presented graphically by ICU study day and treatment group. No formal statistical inference will be performed on these measures. When estimating the mean and SD measures below the limit of quantification (LLQ) will be handled by treating these observations as censored but positive, calculating the likelihood conditional on them being greater than zero. This is strategy M4 from Senn et al., 2012.

4.8 Concomitant medications

The frequency and percentage (of all those in the full analysis set) of patients in whom rescue medications are administered to decrease sedation when the RASS score is -4/-5 will be reported, overall and by treatment arm.

4.9 Intervention dose, fidelity and reach

Dose

The frequency of RASS assessments recorded per shift will be summarised overall, by treatment group and by study site.

Fidelity

The degree of implementation of various components of the A2B interventions will be summarised using the algorithm outlined in Appendix 3. Reporting will cover completeness of day and night shift forms; responses to deep sedation query; completeness of RASS data; completeness of CAM-ICU data on day and night shifts; completeness of pain behaviour data; deep sedation guidance compliance; number and proportion of care periods for each participant in which each of propofol, dexmedetomidine and clonidine was administered will be summarised overall and by treatment group; and propofol, dexmedetomidine and clonidine administration by study day for participants remaining on mechanical ventilation.

For each treatment group, the proportion of participants receiving propofol treatment on each study day will be reported.

Further evaluation of fidelity will be reported in the qualitative process evaluation.

Reach

The number and percentage of eligible patients recruited will be reported overall and by study site. More extensive analysis of reach will be reported in the qualitative process evaluation.

4.10 Protocol deviations and violations

For events which are specific to a participant, the number and percentage of each of protocol deviations and violations will be presented, split by site, trial arm and overall.

Deviations and violations which cannot be attributed to an individual participant (for example, an issue with a process in a site) will be presented in a line listing.

5. Validation and QC

The following will be performed by a second statistician:

1. Separate programming and checking of the primary and supplementary analyses for the primary outcome (Sections 4.3 and 4.4).

2. Separate programming and re-analysis of the mortality secondary outcome and all other secondary outcome analyses for which there is at least one statistically significant pairwise comparison (one-sided p-value <0.025) in the first statistician's analysis. If there are more than 10 such secondary outcomes, then 5 of them will be randomly selected for re-analysis.

3. The end of trial statistical report will be read and checked for accuracy and consistency.

6. Data sharing

A file, or set of files, containing an anonymised version of the final analysis data set will be prepared, along with a data dictionary. These will be made available to the Chief Investigator at the end of the analysis phase.

7. References

Andersen PK, Geskus RB, de Witte T, et al. Competing risks in epidemiology: possibilities and pitfalls. International Journal of Epidemiology 2012;41(3):861-70. doi: 10.1093/ije/dyr213

Fine J and Gray R. A proportional hazards model for the subdistribution of a competing risk. Journal of the American Statistical Association 1999;94(446): 496-509. doi:10.2307/2670170

Groll DL, To T, Bombardier C, Wright JG. The development of a comorbidity index with physical function as the outcome. Journal of Clinical Epidemiology 2005;58:595-602.

Noordzij M, Leffondre K, van Stralen KJ, et al. When do we need competing risks methods for survival analysis in nephrology? Nephrology, dialysis, transplantation : official publication of the European Dialysis and Transplant Association - European Renal Association 2013;28(11):2670-7. doi: 10.1093/ndt/gft355

Poythress JC, Yu Lee M, Young J. Planning and analyzing clinical trials with competing risks: Recommendations for choosing appropriate statistical methodology. Pharmaceutical Statistics. 2020;19:4–21. doi:10.1002/pst.1966

Senn S, Holford N, Hockey H. The ghosts of departed quantities: approaches to dealing with observations below the limit of quantitation. Statist. Med. 2012, 31 4280–4295.

Shehabi Y, Howe BD, Bellomo R, Arabi YM, Bailey M, Bass FE, Bin Kadiman S, McArthur CJ, Murray L, Reade MC, Seppelt IM, Takala J, Wise MP and Webb SA, for the ANZICS Clinical Trials Group and the SPICE III Investigators.* Early Sedation with Dexmedetomidine in Critically Ill Patients. N Engl J Med 2019; 380:2506-17.

Singer M, Deutschman CS, Warren Seymour C, Shankar-Hari M, Annane D, Bauer M, Bellomo R, -Bernard GR, Chiche J-D, Coopersmith CM, Hotchkiss RS, Levy MM, Marshall JC, Martin GS, Opal SM, Rubenfeld GD, van der Poll T, Vincent J-L, Angus DC. The Third International Consensus Definitions for Sepsis and Septic Shock (Sepsis-3). JAMA 2016;315(8):801-810. doi:10.1001/jama.2016.0287

van den Boogaard M, Pickkers P, Slooter AJ, et al. Development and validation of PRE-DELIRIC (PREdiction of DELIRium in ICu patients) delirium prediction model for intensive care patients: observational multicentre study. *BMJ (Clinical research ed)* 2012;344:e420. doi: 10.1136/bmj.e420

Varadhan R, Weiss CO, Segal JB, et al. Evaluating health outcomes in the presence of competing risks: a review of statistical methods and clinical applications. Medical Care 2010;48(6 Suppl):S96-105. doi: 10.1097/MLR.0b013e3181d99107

Zhou B, Fine J, Latouche A, Labopin M. (2012). Competing risks regression for clustered data. Biostatistics 2012;13(3):371-383.

Appendix 1 Sedation Quality Assessment Tool (SQAT)

For a given ICU shift, the sedation quality states of SQAT will be derived as:

Agitation Highest RASS +3/+4 (Daily Data Collection CRF)

Unnecessary deep sedation Lowest RASS -4/-5 AND Was the bedside nurse asked by medical staff to keep this patient deeply sedated? = "No" (Daily Data Collection CRF)

Pain Presence of pain behaviour based on:

Limb movement (Response to moving the participant = "Difficult to move most of the time" OR "Actively resisting movement most of the time") OR

((Compliance with the ventilator = "Tolerating ventilation but coughing/gagging frequently" OR "Unable to control ventilation due to poor patient synchronisation despite different modes tested") AND Was the participant paralysed throughout the entire nursing shift? = "No")

(Daily Data Collection CRF)

Overall optimum sedation is present when there is no agitation; no unnecessary deep sedation; and no pain behaviour.

Statistical Analysis Plan	A2B
Version No	2.0
Date Finalised	dd/mm/yyyy

Appendix 2 PRE-DELIRIC score derivation

The PRE-DELIRIC score will be derived according to the formula in van den Boogaard et al, 2012:

Formula for PRE-DELIRIC model

Risk of delirium = 1/(1+exp−(−6.31

+ 0.04 × age

+ 0.06 × APACHE-II score

+ 0 for non-coma or 0.55 for drug induced coma or 2.70 for miscellaneous coma or 2.84 for combination coma

+ 0 for surgical patients or 0.31 for medical patients or 1.13 for trauma patients or 1.38 for neurology/neurosurgical patients

+ 1.05 for infection

+ 0.29 for metabolic acidosis

+ 0 for no morphine use or 0.41 for 0.01-7.1 mg/24 h morphine use or 0.13 for 7.2-18.6 mg/24 h morphine use or 0.51 for >18.6 mg/24 h morphine use

+ 1.39 for use of sedatives

+ 0.03 × urea concentration (mmol/L)

+ 0.40 for urgent admission))

The scoring system's intercept is expressed as −6.31; the other numbers represent the shrunken regression coefficients (weight) of each risk factor.

Age: Randomisation date minus date of birth (Pre-Randomisation CRF)

APACHE II score: (Baseline CRF)

Coma:

Non-coma	Coma status = "No coma" (Baseline CRF)
Drug induced coma	Coma status = "Coma" AND "With use of medication" (Baseline CRF)
Miscellaneous coma	Coma status = "Coma" AND "Other" (Baseline CRF)
Combination coma	Coma status = "Coma" AND "Combination" (Baseline CRF)

Surgical/Medical/Trauma/Neurology/Neurosurgery:

Surgical	Type of ICU admission = "Non-trauma" AND ("Surgical" NOT (Diagnosis at Admission – Surgical Admission = "Intracerebral haemorrhage" OR "Subdural/epidural haematoma" OR "Subarachnoid haemorrhage" OR "Laminectomy / other spinal cord injury" OR "Craniotomy for neoplasm" OR "Other neurologic diseases"))
Medical	Type of ICU admission = "Non-trauma" AND ("Medical" NOT (Diagnosis at Admission – Medical Admission = "Intracerebral haemorrhage" OR "Subarachnoid haemorrhage" OR "Stroke" OR "Neurologic infection" OR "Neurologic neoplasm" OR "Neuromuscular disease" OR "Seizure" OR "Other neurologic disease"))
Trauma	Type of ICU admission = "Trauma (without traumatic brain injury)"
Neurology/Neurosurgery	Type of ICU admission = "Non-trauma" AND

((Diagnosis at Admission – Surgical Admission = “Intracerebral haemorrhage” OR “Subdural/epidural haematoma” OR “Subarachnoid haemorrhage” OR “Laminectomy / other spinal cord injury” OR “Craniotomy for neoplasm” OR “Other neurologic diseases”) OR (Diagnosis at Admission – Medical Admission = “Intracerebral haemorrhage” OR “Subarachnoid haemorrhage” OR “Stroke” OR “Neurologic infection” OR “Neurologic neoplasm” OR “Neuromuscular disease” OR “Seizure” OR “Other neurologic disease”))
(Baseline CRF)

Infection:
Did the participant receive antibiotics for proven or suspected infection during their first 24 hours in ICU? = ”Yes” (Baseline CRF)

Metabolic acidosis:
pH < 7.35 (H+ > 44.7) with bicarbonate < 24 mmol/L in the first 24 hours in ICU? = “Yes” (Baseline CRF)

Morphine use:
Total administered morphine dose in first 24 hours in ICU =
“Morphine use: 0.01 – 7.1 mg” cumulative OR
“Morphine use: 7.2 – 18.6 mg cumulative” OR
“Morphine use: 18.7 – 331.6 mg cumulative”
(Baseline CRF)

Sedatives:
Any use of propofol, midazolam, lorazepam or combination in the first 24 hours in ICU? = “Yes” (Baseline CRF)

Urea concentration:
Please specify the highest serum urea value in the first 24 hours in ICU [mmol/L]
(Baseline CRF)

Urgent admission: Planned Admission = “Unplanned” (Baseline CRF)

Appendix 3 Data completeness and intervention adherence

Rule 1: Removing non-intervention period days
Remove days on which answer to 'InvasivelyVentilated_YesNoDesc' and 'NonInvVentilation_YesNoDesc' is NO
This will remove the majority of days on which the patient was no longer ventilated during the intervention period. There will be a small number of days on which the response could be NO but the patient is subsequently re-intubated and the primary outcome has not been reached. However, subsequent ventilated days will be included as the answer to this question should revert to YES. For the purpose of tracking data quality this small discrepancy will not be important.
Remaining data should be all days on which patients was receiving mechanical ventilation as defined in the protocol

Rule 2: completeness of day and night shift forms
After rule 1:
Count proportion of 'DSBedsideNurse_YesNoDesc' that response is YES
Count proportion of 'NSBedsideNurse_YesNoDesc' that response is YES
Report this as proportion of 'shift forms' completed by clinical staff during day shift and night shift and overall by site and overall trial

Rule 3: responses to deep sedation query
After rule 1:
Count proportion of 'DSDeepSedation_YesNoNotCollectedDesc' reported for each category
Count proportion of 'NSDeepSedation_YesNoNotCollectedDesc' reported for each category
Report this for day shift and night shift and for overall by site and overall trial

Rule 4: completeness of sedation RASS data
After rule 1:
Report completeness of:
'DSHighestRASS_RASSScoreDesc'
'DSLowestRASS_RASSScoreDesc'
'NSHighestRASS_RASSScoreDesc'
'NSLowestRASS_RASSScoreDesc'
To provide a measure of ability to report a highest and lowest recorded RASS score on each day report:
Proportion of days on which:
'DSHighestRASS_RASSScoreDesc' OR 'NSHighestRASS_RASSScoreDesc' OR BOTH have a RASS score reported
'DSLowestRASS_RASSScoreDesc' OR 'NSLowestRASS_RASSScoreDesc' OR BOTH have a RASS score recorded

Rule 5: completeness of CAM-ICU data
After rule 1:
Report the following:
Day shift
Proportion of day shifts on which 'DSCAMICUPositive_CAMICUPositiveDesc' is YES OR NO (this is a definite response)
Proportion of day shifts on which 'DSCAMICUPositive_CAMICUPositiveDesc' response is 'RASS -3 but clinical team unable to assess CAM-ICU'

Proportion of day shifts on which 'DSCAMICUPositive_CAMICUPositiveDesc' response is 'Not collected' AND responses to ['DSHighestRASS_RASSScoreDesc' AND 'DSLowestRASS_RASSScoreDesc'] are both [-3 or -4 or -5]
Proportion of day shifts on which 'DSCAMICUPositive_CAMICUPositiveDesc' response is 'Not collected' AND responses to ['DSHighestRASS_RASSScoreDesc' AND 'DSLowestRASS_RASSScoreDesc'] are both [-2 or -1 or 0 or +1 or +2 or +3 or +4]
Night shift
Proportion of night shifts on which 'NSCAMICUPositive_CAMICUPositiveDesc' is YES OR NO (this is a definite response)
Proportion of night shifts on which 'NSCAMICUPositive_CAMICUPositiveDesc' response is 'RASS -3 but clinical team unable to assess CAM-ICU'
Proportion of night shifts on which 'NSCAMICUPositive_CAMICUPositiveDesc' response is 'Not collected' AND responses to ['NSHighestRASS_RASSScoreDesc' AND 'NSLowestRASS_RASSScoreDesc'] are both [-3 or -4 or -5]
Proportion of night shifts on which 'NSCAMICUPositive_CAMICUPositiveDesc' response is 'Not collected' AND responses to ['NSHighestRASS_RASSScoreDesc' AND 'NSLowestRASS_RASSScoreDesc'] are both [-2 or -1 or 0 or +1 or +2 or +3 or +4]

Rule 6: completeness of pain behaviour data
After rule 1:
Proportion of day shifts on which 'DSCompliance_VentilatorComplianceDesc' response is 'not collected by bedside nurse'
Proportion of day shifts on which 'DSCompliance_VentilatorComplianceDesc' response is NULL (ie no data)
Proportion of day shifts on which 'DSCompliance_VentilatorComplianceDesc' response is 'not collected by bedside nurse'
Proportion of day shifts on which 'DSCompliance_VentilatorComplianceDesc' response is NULL (ie no data)
Proportion of night shifts on which 'NSCompliance_VentilatorComplianceDesc' response is 'not collected by bedside nurse'
Proportion of night shifts on which 'NSCompliance_VentilatorComplianceDesc' response is NULL (ie no data)
Proportion of night shifts on which 'NSCompliance_VentilatorComplianceDesc' response is 'not collected by bedside nurse'
Proportion of night shifts on which 'NSCompliance_VentilatorComplianceDesc' response is NULL (ie no data)

Rule 7: indicative sedation guidance compliance
Shifts during which deep sedation was NOT requested
After rule 1:
Select shifts where response to 'DSDeepSedation_YesNoNotCollectedDesc' AND 'NSDeepSedation_YesNoNotCollectedDesc' is NO
For these shifts:
Proportion of each RASS score response to 'DSHighestRASS_RASSScoreDesc' AND 'NSHighestRASS_RASSScoreDesc'
These cumulative data should indicate how common it is for a patient in whom deep sedation was NOT requested for the patient NOT to achieve a highest recorded RASS of -2 or greater during the intervention period.

Rule 8: Correct administration of drugs according to group
After rule 1:
Patients allocated to usual care group
Number/proportion of days on which propofol administered ‘Propofol_YesNoDesc’
Number/proportion of days on which dexmedetomidine administered ‘Dexmedetomidine_YesNoDesc’
Number/proportion of days on which clonidine administered ‘Clonidine_YesNoDesc’
Patients allocated to dexmedetomidine group
Number/proportion of days on which propofol administered ‘Propofol_YesNoDesc’
Number/proportion of days on which dexmedetomidine administered ‘Dexmedetomidine_YesNoDesc’
Number/proportion of days on which clonidine administered ‘Clonidine_YesNoDesc’
Patients allocated to clonidine group
Number/proportion of days on which propofol administered ‘Propofol_YesNoDesc’
Number/proportion of days on which dexmedetomidine administered ‘Dexmedetomidine_YesNoDesc’
Number/proportion of days on which clonidine administered ‘Clonidine_YesNoDesc’
This plot will give an overall indication of compliance without adjustment for the day of study.

Rule 9: correct administration according to group and day of study
Using Rule 8 data:
For each intervention group separately:
For study day 1, study day 2, study day 3 etc plot
Number/proportion of days on which propofol administered ‘Propofol_YesNoDesc’
Number/proportion of days on which dexmedetomidine administered ‘Dexmedetomidine_YesNoDesc’
Number/proportion of days on which clonidine administered ‘Clonidine_YesNoDesc’
This plot will provide an indication of compliance according to the day of intervention (for patients remaining on mechanical ventilation.
