# Supplementary material for: Alpha 2 agonists for sedation to produce better outcomes from critical illness (A2B Trial): protocol for a multicentre phase 3 pragmatic clinical and cost-effectiveness randomised trial in the UK
Source: BMJ Open. 2023 Dec 9;13(12):e078645. doi: 10.1136/bmjopen-2023-078645 (PMC10729141; doi:10.1136/bmjopen-2023-078645)
Supplement: Author's manuscript [file bmjopen-2023-078645.draft_revisions.pdf]

BMJ Open

BMJ Open is committed to open peer review. As part of this commitment we make the peer review history of every article we publish publicly available.

When an article is published we post the peer reviewers' comments and the authors' responses online. We also post the versions of the paper that were used during peer review. These are the versions that the peer review comments apply to.

The versions of the paper that follow are the versions that were submitted during the peer review process. They are not the versions of record or the final published versions. They should not be cited or distributed as the published version of this manuscript.

BMJ Open is an open access journal and the full, final, typeset and author-corrected version of record of the manuscript is available on our site with no access controls, subscription charges or pay-per-view fees (http://bmjopen.bmj.com).

If you have any questions on BMJ Open's open peer review process please email info.bmjopen@bmj.com

BMJ Open

Alpha 2 agonists for sedation to produce better outcomes from critical illness (A2B Trial): Rationale, study design and statistical analysis plan for a phase 3 pragmatic clinical and cost- effectiveness randomised trial

Journal:	*BMJ Open*
Manuscript ID	bmjopen-2023-078645
Article Type:	Protocol
Date Submitted by the Author:	09-Aug-2023

Complete List of Authors:	Walsh, Timothy; The University of Edinburgh Usher Institute of Population Health Sciences and Informatics<br>Aitken, Leanne M; City University; City University of London<br>McKenzie, Cathrine; University of Southampton<br>Boyd, Julia; The University of Edinburgh Usher Institute of Population Health Sciences and Informatics, Edinburgh Clinical Trials Unit<br>Macdonald, Alix; The University of Edinburgh Usher Institute of Population Health Sciences and Informatics<br>Giddings, Annabel; The University of Edinburgh Usher Institute of Population Health Sciences and Informatics<br>Hope, David; NHS Lothian<br>Norrie, John; Edinburgh Clinical Trials Unit, University of Edinburgh No. 9, Bioquarter, Usher Institute<br>Weir, Christopher; University of Edinburgh, Edinburgh Clinical Trials Unit, Usher Institute<br>Parker, Richard; University of Edinburgh, Edinburgh Clinical Trials Unit<br>Lone, Nazir; The University of Edinburgh Usher Institute of Population Health Sciences and Informatics<br>Emerson, Lydia; City University of London<br>Kydonaki, Kalliopi ; Edinburgh Napier University<br>Creagh-Brown, Benedict; Royal Surrey County Hospital NHS Foundation Trust; Royal Surrey County Hospital, Intensive Care Unit<br>Morris, Stephen; University of Cambridge, Primary Care Unit<br>McAuley, Daniel; Queen's University Belfast, Centre for Experimental Medicine<br>Dark, Paul; University of Manchester, Intensive Care Unit<br>Wise, Matt ; University Hospital of Wales, Dept. of adult critical care<br>Gordon, Anthony; Imperial College London, 1. Section of Anaesthetics, Pain Medicine and Intensive Care<br>Perkins, Gavin; University of Warwick, Clinical Trials Unit<br>Reade, Michael; University of Queensland<br>Blackwood, Bronagh; Queen's University Belfast, Wellcome-Wolfson Institute for Experimental Medicine<br>MacLullich, Alasdair; University of Edinburgh, Geriatric Medicine Unit<br>Glen, Robert; NHS Lothian<br>Page, Valerie; West Hertfordshire Hospitals NHS Trust, Intensive Care; Imperial College London Faculty of Medicine,

Keywords:	Clinical Trial, Adult intensive & critical care < INTENSIVE & CRITICAL CARE, Clinical trials < THERAPEUTICS

SCHOLARONE™ Manuscripts

Alpha 2 agonists for sedation to produce better outcomes from critical illness (A2B Trial): Rationale, study design and statistical analysis plan for a phase 3 pragmatic clinical and cost- effectiveness randomised trial

Timothy S Walsh, Dept of Anaesthesia, Critical Care and Pain Medicine, Usher Institute, University of Edinburgh, Edinburgh, EH16 4SA, UK

Leanne M Aitken, School of Health & Psychological Sciences, City, University of London, London EC1V 0HB, UK leanne.aitken.1@city.ac.uk

Cathrine A McKenzie, University of Southampton, School of Medicine, National Institute of Health, and Social Care Research (NIHR), Biomedical Research Centre, Perioperative and Critical Care Theme, SO17 1BJ, UK

Julia Boyd, Edinburgh Clinical Trials Unit, Usher Institute, University of Edinburgh, Edinburgh, EH16 4UX, UK

Alix Macdonald, Edinburgh Clinical Trials Unit, Usher Institute, University of Edinburgh, Edinburgh, EH16 4UX, UK

Annabel Giddings, Edinburgh Clinical Trials Unit, Usher Institute, University of Edinburgh, Edinburgh, EH16 4UX, UK

David Hope, Edinburgh Critical Care Research Group, NHS Lothian, Edinburgh, EH16 4SA, UK

John Norrie, Edinburgh Clinical Trials Unit, Usher Institute, University of Edinburgh, Edinburgh, EH16 4UX, UK

Christopher J Weir, Edinburgh Clinical Trials Unit, Usher Institute, University of Edinburgh, Edinburgh, EH16 4UX, UK

Richard A Parker, Edinburgh Clinical Trials Unit, Usher Institute, University of Edinburgh, Edinburgh, EH16 4UX, UK

Nazir Lone, Dept of Anaesthesia, Critical Care and Pain Medicine, Usher Institute, University of Edinburgh, Edinburgh, EH16 4SA, UK

Lydia Emerson, School of Health & Psychological Sciences, City, University of London, London EC1V 0HB, UK

Kalliopi Kydonaki, School of Health and Social Care, Edinburgh Napier University, 9 Sighthill Court, EH11 4BN, Edinburgh, UK. C.Kydonaki@napier.ac.uk/ National and Kapodistrian University of Athens, Nursing department, 123 Papadiamadopoulou st. Athens, Greece.

Ben Creagh-Brown, Intensive Care Unit, Royal Surrey NHS Foundation Trust, Guildford, GU2 7XX, UK; Faculty of Health and Medical Sciences, University of Surrey, Guildford, UK.

For peer review only

Stephen Morris, Dept Public Health and Primary Care, University of Cambridge, Cambridge CB1 8RN, UK

Daniel F McAuley, School of Medicine, Dentistry and Biomedical Sciences, Queens University Belfast, Belfast, UK

Paul Dark, Critical Care Medicine, Division of Immunology, Immunity to infection and Respiratory Medicine, University of Manchester, Manchester M15 6JA, UK

Matt P Wise, Adult Critical Care, University Hospital of Wales, Cardiff, CF14 4XW, UK

mattwise@doctors.org.uk

Anthony C Gordon, Division of Anaesthetics, Pain Medicine and Intensive Care, Imperial College London, London W2 1NY, UK

Gavin D Perkins, Warwick Medical School, University of Warwick, Coventry, CV4 7AL, UK

Michael C. Reade, Faculty of Medicine, University of Queensland. Herston, Brisbane, 4029, Australia. m.reade@uq.edu.au

Bronagh Blackwood, School of Medicine, Dentistry and Biomedical Sciences, Queens University Belfast, Belfast, UK

Alasdair MacLullich, Edinburgh Delirium Research Group, Ageing and Health, Usher Institute, University of Edinburgh, Edinburgh, EH16 4SA, UK

Robert Glen, Lay Representative

Valerie Page, Dept of Anaesthetics, West Herts Teaching Hospitals NHS Trust, Watford, WD18 0HB, UK

Corresponding author:

Professor Tim Walsh

Department of Anaesthesia, Critical Care & Pain Medicine

Centre for Population Health Sciences, Usher Institute

Room S8208, 2nd Floor

The Royal Infirmary of Edinburgh

51 Little France Crescent

Edinburgh BioQuarter

Edinburgh EH16 4SA

Phone: 0131 242 6395

e-mail: timothy.walsh@ed.ac.uk

For peer review only

Key Words

Critical Illness; sedation; clinical trial; alpha2-agonists; mechanical ventilation

Word Count: 4598

Figures: 1

Tables: 3

This manuscript has an electronic supplement

For peer review only

Abstract

Introduction

Almost all patients receiving mechanical ventilation (MV) in intensive care units (ICUs) require analgesia and sedation. The most widely used sedative drug is propofol, but there is uncertainty whether alpha2-agonists are superior. The A2B trial aims to determine whether clonidine or dexmedetomidine (or both) are clinically and cost-effective in MV ICU patients compared to usual care.

Methods and analysis

Adult ICU patients within 48 hours of starting MV, expected to require at least 24 hours further MV, are randomised in an open-label three arm trial to receive propofol (usual care) or clonidine or dexmedetomidine as primary sedative, plus analgesia according to local practice. Exclusions include patients with primary brain injury; post-cardiac arrest; other neurological conditions; or bradycardia. Unless clinically contra-indicated, sedation is titrated using weight-based dosing guidance to achieve a Richmond-Agitation-Sedation score of -2 or greater as early as considered safe by clinicians. The primary outcome is time to successful extubation. Secondary ICU outcomes include delirium and coma incidence/duration, sedation quality, predefined adverse events, mortality, and ICU length of stay. Post-ICU outcomes include mortality, anxiety and depression, post-traumatic stress, cognitive function, and health-related quality of life at 6-month follow-up. A process evaluation and health economic evaluation are embedded in the trial.

The analytic framework uses a hierarchical approach to maximise efficiency and control type I error. Stage 1 tests whether each alpha2-agonist is superior to propofol. If either/both interventions are superior, stage 2 and 3 testing explores which alpha2-agonist is more effective. To detect a mean difference of 2 days in MV duration, we aim to recruit 1437 patients (479 per group) in 40-50 UK ICUs.

Ethics and dissemination

The Scotland A REC approved the trial (18/SS/0085). We use a surrogate decision-maker or deferred consent model consistent with UK law. Dissemination will be via publications, presentations, and updated guidelines.

Trial registration

ClinicalTrials.gov NCT03653832

299 words

For peer review only

Trial Summary

'Strengths and limitations of this study'

- This is the largest randomised trial simultaneously comparing both clonidine and dexmedetomidine to propofol (usual care) in a pragmatic effectiveness design.
- The trial maximises efficiency by using a hierarchical approach to hypothesis testing that primarily establishes whether each alpha2-agonist is superior to propofol, but retains power to explore their relative effectiveness if this is demonstrated.
- In addition to the primary outcome, the trial will measure important patient-centred outcomes such as delirium, sedation quality, and also longer-term psychological well-being and health-related quality of life.
- The trial includes a process evaluation that will provide information to help understand the results.
- The trial includes a detailed health economic evaluation, which is relevant because ICU care is costly; in addition, there are differences in costs between the drugs which are changing over time.
- The trial has moderate power to detect potentially important differences in mortality, and heterogeneity of effects according to patient age and other factors.
- The COVID19 pandemic required a reduction in the planned sample size from 1650 to 1437 patients; the main effect on power is for the non-inferiority comparison of clonidine versus dexmedetomidine

For peer review only

Introduction

Around 20 million patients worldwide require intubation and mechanical ventilation (MV) in intensive care units (ICUs) each year.[1] Almost all require sedation and analgesia to relieve pain and anxiety, achieve comfort, and facilitate treatment. Guidelines recommend that patients are kept awake or lightly sedated whenever possible, and as early during ICU care as possible.[2-4] Sedative choice may influence the prevalence and duration of delirium, which is associated with adverse outcomes. However, it remains uncertain whether this relationship is causal, in part because delirium prevention and management strategies have been ineffective in most studies.

Research has shown an association between deep sedation and adverse short-term outcomes including prolonged MV and ICU stay, hospital acquired infections, and greater mortality, although this evidence has been inconsistent.[2 5 6] A concern regarding keeping patients more awake has been whether long-term psychological morbidity, such as post-traumatic stress, anxiety, and depression might be increased.[7-9] It is uncertain whether 'light sedation' strategies or the choice of sedative agent can modify this, either directly or by decreasing delirium.[8 10 11]

The most established drugs for patient sedation are the gamma-aminobutyric acid receptor (GABA) agonists, namely propofol or benzodiazepines. These are prescribed once adequate analgesia, usually with opioid drugs, has been established. Benzodiazepines are associated with greater delirium, and propofol is recommended for first line use in guidelines and is the first-line sedative in the UK. Alpha2 agonists are an alternative class of sedative that provide sedation by dose-dependent decrease in noradrenergic neuron activity in the brain stem via pre- and post-synaptic receptor-mediated effects.[12] Unlike GABAergic sedatives, alpha2 agonists have analgesic properties, which can reduce opioid requirements.[13] Two alpha2-agonists are in widespread use in ICUs in the United Kingdom:

Dexmedetomidine is a highly selective alpha2-agonist with a α2:α1 receptor selectivity ratio of 1620:1.[14] It was developed as a sedative agent and is licensed for intravenous ICU sedation. The drug is >90% protein bound. Unbound drug crosses the blood–brain barrier to exert central effects. Metabolism in the liver creates inactive metabolites which are excreted renally. Renal impairment does not significantly alter clinical effects. The terminal elimination half-life is around 2 hours.

Clonidine was the prototype alpha2-agonist, licensed for hypertension, but subsequently used therapeutically for a wide range of neuropsychiatric conditions, drug withdrawal syndromes, and in pain medicine.[15] The drug is available in multiple formulations (including oral, transdermal, and intravenous). Many clinical uses are unlicensed, including ICU sedation via any route. Clonidine has significantly lower α2-receptor selectivity than dexmedetomidine; α2:α1 selectivity is 220:1 (x8 less than dexmedetomidine). Clonidine is less protein bound than dexmedetomidine (20-40%), and around 65% is excreted unchanged in the urine. The elimination half-life is significantly longer and variable (typically 5-13 hours), and (unlike dexmedetomidine) is prolonged by renal failure (18-41 hours). Peak effects after a single dose occur after 10-60 minutes, but may last 3-7 hours.

For peer review only

A survey of UK ICUs when planning this trial found 58% of ICUs use dexmedetomidine, but in less than 10% of patients. More than 90% used clonidine, in up to 25% of patients, but administration route and protocols varied widely. Widespread practice variation was present. Although widely used in the UK, intravenous clonidine has limited international use and is not included in international guidelines[16]. Dexmedetomidine is licensed for ICU sedation and has been manufactured 'off patent' since 2019. Clonidine not licensed for ICU use, but is administered via both oral/enteral and intravenous routes, especially for the management of agitation and delirium.

Current evidence

The safety and effectiveness of clonidine for ICU sedation has not been studied in large randomised trials. A systematic review (SR) of studies in critical care included eight studies (643 patients).[17] There was important and relevant heterogeneity in multiple areas, including the population; routes of administration (6 intravenous and 2 oral); and dosage regimens. In 7 of 8 trials clonidine was used for adjunctive rather than stand-alone sedation. Meta-analysis suggested no effect on clinical outcomes but an association with hypotension (RR 3.11; 95% CI = 1.64 to 5.87).

Dexmedetomidine has been widely studied, and evidence summarised in a range of systematic reviews (SR) and meta-analyses. These have varied in terms of population definition (including SRs of all critically ill MV adults, or restricted to older patients or those with sepsis) and also the comparator (including 'usual care sedation' or propofol). The primary outcomes include mortality, duration of mechanical ventilation, and delirium. SRs prior to 2020 did not include data from the largest trial of dexmedetomidine (see below). The most recent SRs compared dexmedetomidine versus other sedative agents[18] or propofol[19] in critically ill MV adults in published trials to 2022. Dexmedetomidine was found to reduce delirium (moderate certainty), the duration of MV (low certainty), and ICU length of stay (low certainty)[18]. There was no effect on mortality at 30 days (moderate certainty). Dexmedetomidine increased the risk of bradycardia and hypotension. Authors commented on population heterogeneity, with different risk profiles for key clinical outcomes.

The SPICE III trial randomised 4000 patients to receive dexmedetomidine or usual care within 12 hours of ICU admission.[20] The primary outcome of mortality was no different between the groups. Patients in the dexmedetomidine group had more ventilator free days (VFDs) and more days free of coma or delirium during 28 days follow-up. The median duration of ventilation in the trial was 3-4 days, and overall dexmedetomidine patients gained one VFD and had one less day of coma/delirium during 28 days follow-up. There were 6 pre-defined sub-group analyses. There were no differences in mortality according to baseline illness severity, severity of oxygenation impairment, geographic region, admission type (operative/non-operative), or sepsis at enrolment. There was a difference in mortality for patients above and below the median patient age. Patients aged <63.7 years who received dexmedetomidine experienced more deaths (mean absolute risk difference 4.4% (95% CI 0.8% -7.9%)), and patients aged ≥63.7 years experienced fewer deaths (mean absolute risk difference -4.4% (95% CI -8.7% - -0.1%)). This finding was explored in a detailed *post hoc* analysis which confirmed the finding using a range of statistical approaches, but without an explanation for the effect.[21] A cluster analysis suggested that a beneficial effect on mortality

may be most marked in operative versus non-operative patients. Based on these data a caution around increased mortality risk in patients aged ≤65 years was issued in June 2022 by the European Medicine Agency (EMA)[22].

Pharmaco-economic considerations

There is a cost-difference between the three agents used in the A2B trial, but the cost of dexmedetomidine has decreased substantially since coming off-licence. Current estimates (August 2023) for a typical daily UK cost for sedating a 70kg adult receiving MV in the UK are: propofol £15 (€17); dexmedetomidine £22 (€25) and clonidine £8 (€9). Changes in cost, combined with potential effects on clinically important outcomes mean a health economic evaluation of alpha2-agonists is relevant.

Research Commission and funding

The A2B trial was funded as a UK National Institute of Health and Care Research (NIHR) Health Technology Assessment (HTA) Agency commissioned trial (16/93 ‘alpha-2 agonists for sedation in critical care’, 2017). The project brief specifically highlighted the widespread off-licence use of clonidine in the absence of safety and effectiveness evidence (funder reference HTA Project:16/93/01).

Trial Registration

The trial is registered on ClinicalTrials.gov (NCT03653832); EudraCT number is 2018-001650-98. This paper is based on protocol version 7.0 (date: 25/4/2023)

Methods and analysis:

The primary hypothesis is that sedation with alpha2-agonists will decrease the time to extubation in adult MV ICU patients compared with propofol (usual care).

Design

Randomised, parallel-group, allocation concealed, controlled, open-label, phase 3, pragmatic, clinical and cost-effectiveness trial with an internal pilot. After intubating and stabilising patients, we randomise patients (1: 1: 1) as early as possible to receive sedation-analgesia based on clonidine *or* dexmedetomidine *or* to continue propofol (usual care) plus opioid analgesia as required.

Patients and Public Involvement (PPI)

Former ICU patients and their relatives were consulted during the application to the NIHR Health Technology Assessment panel in addressing the importance of the research questions, and the design of the study, through participation in focus groups. A former ICU patient (RG) is a co-applicant on the grant and co-investigator on the trial. The PPI group were consulted when agreeing the primary and secondary outcomes, and played a key role in agreeing the long term outcome measures, the frequency of assessment, and the tools used to collect

For peer review only

them. RG is providing advice throughout the trial. In addition, the Trial Steering Group includes an independent lay member.

Primary Objective

To determine whether intravenous sedation with the alpha2-agonist agents, dexmedetomidine or clonidine, can decrease the time to successful extubation from MV among adult critically ill patients.

Secondary Objectives

Clinical and Person-centred objectives

During ICU stay we compare rates and duration of delirium or coma, time to optimum sedation, average sedation depth, the ability of patients to communicate with staff and relatives, the quality of sedation, and duration of ICU stay. We also compare safety based on pre-defined adverse events relevant to sedation and alpha2-agonist agents.

Following discharge from the ICU we compare patient outcomes for which sedation and ICU experience may be on the causal pathway, namely patients' memories of their ICU stay, psychological wellbeing, and cognitive function. We will follow up patients for 6 months for survival, health-related quality of life (HRQoL), and healthcare resource use.

Economic evaluation

We will include a detailed cost-effectiveness analysis from an NHS and personal social services perspective.

Process evaluation

The trial, by necessity, is a complex healthcare intervention trial evaluating different classes of sedative agents that involves multiple healthcare professionals, assessing and delivering multiple agents using a series of interrelated activities guided by bedside flowcharts, across multiple sites. Recognising this, and consistent with the MRC complex intervention framework[23], we include a process evaluation to explore the processes involved in intervention delivery, and identify factors and the mechanisms of their interaction likely impacting on trial outcomes.

Outcomes and Endpoints

Primary endpoint:

Time to successful extubation post-randomisation (hours). This is defined as:

a. For patients with an endotracheal tube: the time of the first extubation that is followed by 48 hours of spontaneous breathing without mechanical support

For peer review only

b. For patients with a tracheostomy: the start time of the patient's first period of 48 hours of spontaneous breathing, where spontaneous breathing is defined as receiving support not exceeding 5 cmH_2O Positive End Expiratory Pressure (PEEP) or Continuous Positive Airway Pressure (CPAP) with ≤ 5 cmH_2O pressure support above PEEP

c. For patients who are receiving non-invasive mechanical ventilation (NIV): the start time of the patient's first period of 48 hours of spontaneous breathing, defined as receiving support not exceeding 5 cmH_2O CPAP via mask/hood

Secondary outcomes

The A2B trial has a range of clinical and patient centred outcomes, which were discussed and approved following a Public and Patient Involvement exercise. These are shown in table 1.

Table 1: secondary outcomes, measurement tool or method, and timing.

Outcome	Measurement tool or method	Timing
**Mortality**	Medical records check	ICU, hospital, 30, 90 and 180 days post randomisation
**Length of ICU stay**<br>Number of days the participant is in ICU	Medical record	ICU discharge
**Sedation and analgesia quality**<br>Lowest and highest RASS score per day over time during intervention<br><br>Quality of sedation using SQAT states (daily basis); days with optimum sedation, agitation, or unnecessary deep sedation (RASS -4/-5).<br><br>Quality of analgesia using presence of pain behaviour (daily basis) based on limb response to movement and ventilation compliance	Richmond Agitation and Sedation Scale (RASS)<br><br>Sedation Quality (based on Sedation Quality Assessment Tool (SQAT).[24] Two components of the SQAT pain assessment will be used in this trial to measure sedation quality (limb relaxation and compliance with ventilation)<br>Defines four states for sedation quality:<br>1. Overall optimum sedation (no agitation; no unnecessary deep sedation; no pain behaviour)<br>2. Agitation<br>3. Unnecessary deep sedation (RASS -4/-5 without clinical indication)<br>4. Pain (presence of pain behaviour based on limb response to movement and ventilation compliance)	Four hourly during ICU stay until primary outcome is reached<br><br>Derived from daily sedation and analgesia quality data during intervention period in ICU until primary outcome is reached
**Time to first Optimum sedation** Hours<br>Hours from randomisation to first 'light' sedation (RASS score of -2 or greater)	RASS scores 4 hourly during ICU stay<br><br>SQAT status (daily during ICU stay)	Based on daily sedation and pain assessments during the intervention period

For peer review only

Days from randomisation to first day with optimum sedation (based on SQAT definition)		
**Delirium prior to successful extubation**<br>Occurrence prior to successful extubation (binary outcome)<br><br>Days with delirium (CAM-ICU positive) or coma (RASS score -4/-5) prior to successful extubation (continuous outcome)	Confusion Assessment Method for the ICU (CAM-ICU)[25]	Twice daily during ICU stay until primary outcome is reached
**Drug-related adverse events**<br>Number of patients experiencing a predefined adverse event and each defined adverse event<br><br>Number of days prior to successful extubation that any predefined adverse event occurred, and each defined adverse event occurred.	Severe bradycardia; cardiac arrhythmias; cardiac arrest (defined in protocol)	Daily during the intervention period
**Health-related Quality of Life**<br>HRQoL at 30, 90, and 180 days post randomisation	EuroQol tool (EQ-5D-5L)	Recalled HRQoL prior to hospital admission; prospective measurement 30, 90 and 180 days post randomisation
**Patients' Ability to Communicate Pain and Ability to Cooperate with Care**<br><br>Number of days on which pain could be communicated during intervention (binary score)<br><br>Number of days on which patient was able to cooperate with care (binary score)	Binary assessment for each 12 hours nursing shift requested from bedside nurse (based on overall assessment of period of care). Answer to the following questions:<br>1. Was your patient able to communicate pain?<br>2. Was your patient able to cooperate with care?	Twice daily until primary outcome is reached
**Patient experience of ICU care**<br>ICE-Q score at 90 days post-randomisation overall for each domain	Intensive Care Experience Questionnaire (ICE-Q)[26]<br>Provides numeric score in four domains:<br>1. Awareness of Surroundings<br>2. Frightening Experiences<br>3. Recall of Experiences<br>4. Satisfaction with Care	90 days post randomisation
**Relative/partner/friend *(PerLR)* assessment of comfort and communication**<br><br>Daily response to each of the three questions (binary outcome)	Relative/partner/friends response to the following questions (based on their opinion at time of assessment):<br>1. Does the patient appear awake to the visitor?<br>2. Does the patient seem comfortable to the visitor?<br>3. Does the visitor feel they can communicate with the patient?	Daily at a visit until primary outcome is reached

For peer review only

**Anxiety and depression**<br>HADS score at 180 days post-randomisation	Hospital Anxiety and Depression Scale (HADS) questionnaire	180 days post randomisation
**Post-traumatic stress**<br>Impact of Events Scale-revised (IES-R) score at 180 days post-randomisation	Impact of Events Scale-revised (IES-R)	180 days post randomisation
**Cognitive function**<br>TMoCA score at 180 days post-randomisation	Montreal Cognitive Assessment Tool (Telephone version) (TMoCA)	180 days post randomisation

Study population

The target population are critically ill patients requiring MV, recruited as early during ICU stay as possible, with an anticipated total requirement for MV of *at least* two days. Alpha2-agonists are not appropriate as single agents for intubation and early sedation for most acutely ill patients. Anaesthesia to undertake endotracheal intubation and establish initial ICU sedation-analgesia follows current usual care.

Inclusion and exclusion criteria

Inclusion and exclusion criteria are listed in table 2.

Table 2: inclusion and exclusion criteria for the A2B trial.

**Inclusion criteria**
1. Patient requiring MV in an ICU<br>2. Aged 18 or over<br>3. Within 48 hours of first episode of mechanical ventilation in ICU<br>4. Requiring sedation with propofol<br>5. Expected to require *a total* of 48 hours of MV or more in ICU<br>6. Expected to require a further 24 hours of MV or more *at the time of randomisation* in the opinion of the responsible clinician<br>Note: Criteria 5 and 6 are intended to ensure that all participants require at least 48 hours of MV in the ICU and that all patients receive at least 24 hours of the allocated intervention after randomisation.
**Exclusions**
1. Acute brain injury (traumatic brain injury; intracranial haemorrhage; ischaemic brain injury from stroke or hypoperfusion)[1]<br>2. Post-cardiac arrest (where there is clinical concern about hypoxic brain injury)[1]<br>3. Status epilepticus[1]<br>4. Continuous therapeutic neuromuscular paralysis at the time of screening or randomisation[1]<br>5. Guillain-Barre Syndrome[1]<br>6. Myasthenia gravis[1]<br>7. Home ventilation[1, 4]<br>8. Fulminant hepatic failure[2]<br>9. Patient not expected by responsible clinician to survive 24 hours

For peer review only

10. Decision to provide only palliative or end-of-life care
11. Pregnancy
12. Known allergy to one of the study drugs
13. Patient known to have experienced a period with heart rate <50 beats per minute for 60 minutes or longer since commencing mechanical ventilation in the ICU
14. Untreated second or third degree heart block[3]
15. Transferred from another Intensive Care Unit in which MV occurred for >6 hours
16. Prisoners
17. Enrolled on another Clinical Trial of an Investigational Medicinal Product
18. Previously enrolled on the A2B Trial

Note:
[1]For these conditions the neuromuscular condition will dominate the primary outcome unrelated to sedation practice
[2]Uncertain pharmacokinetics of α-2 agonist; potential for cerebral oedema mandating deep sedation
[3]Patients with treated heart block, for example with a pacemaker, are eligible for inclusion
[4]Home ventilation does not include patients receiving night-time CPAP and/or BIPAP therapy for the treatment of obstructive sleep apnoea syndrome.

Screening and consent

Participants are identified by clinical and research teams. Potential participants lack mental capacity. Appropriate approaches to consent according to UK law are used, approaching Personal and Professional legal representatives. The use of the 'emergency provision' can be used for deferred consent when a legal representative is not available within 2 hours of meeting eligibility criteria. In all cases, when patients regain capacity, they are approached for consent to continue in the trial (see supplementary material).

Randomisation

Randomisation is undertaken immediately after consent is obtained or when deferred consent is triggered by the research team, using a remote web-based randomisation system. Randomisation is in a 1:1:1 ratio to the three interventions using permuted blocks (randomly arranged sizes of 3, 6, 9, 12) stratified by centre. The allocation sequence was generated by a clinical trials unit programmer not involved in clinical management and is stored on a remote secure server concealed from all personnel involved in the trial.

Intervention Groups

Patients commence intravenous infusion of open-label study drug according to a weight-based dose regimen (see supplementary material) as early as possible post-randomisation, and within a maximum of two hours.

Bedside clinical staff transition patients to achieve sedation with the allocated alpha2-agonist agent as quickly as clinically feasible and safe, using bedside guidance algorithms (see supplementary material). Additional opioid is used for analgesia using clinical judgement. Once alpha2-agonist is established, additional propofol is only recommended when the

For peer review only

maximum alpha2-agonist dose is reached or because cardiovascular or other side-effects limit dose escalation.

Dexmedetomidine group

For dexmedetomidine, starting dose is 0.7micrograms/kg/hour titrated to a maximum dose 1.4micrograms/kg/hour as per manufacturer guidance. Lower starting doses are used at clinical discretion for patients with cardiovascular instability e.g. for patients on high doses of norepinephrine. No loading dose is administered.

Clonidine group

For clonidine, the regimen is designed to be equipotent with dexmedetomidine based on known pharmacokinetics and pharmacodynamics. The chosen regimen is similar to that currently used in many UK ICUs as part of routine 'off label' practice. The starting dose is 1.0micrograms/kg/hour titrated to a maximum dose of 2micrograms/kg/hour. Lower starting doses can be used at clinical discretion for patients with cardiovascular instability as for dexmedetomidine. No loading dose is administered.

Usual care group

Patients continue to receive intravenous propofol according to current usual care. The sedation targets, weaning, and sedation discontinuation procedures follow the same clinical targets as for the intervention groups.

The dosing guidance algorithms are included in the supplementary material.

Duration of intervention

The intervention period continues until: [1] The patient is successfully extubated according to the definition of the primary outcome; or [2] the patient dies during MV in the ICU; or [3] the patient is transferred to another non-participating ICU prior to achieving the primary outcome, or [4] 28 days of MV in ICU have been required following randomisation without achieving the primary outcome.

Timing of discontinuation of sedative agents is at the discretion of the clinical team. If the patient is re-intubated before achieving the primary outcome, they continue with group allocated treatment until the primary outcome is successfully achieved.

Management during the intervention period

The default sedation target is the most awake and comfortable state considered safe by clinical staff. For each 12 hours nursing shift, clinical staff document whether there is a clinical indication for deep sedation. If deep sedation is required, the allocated sedative agent is titrated to achieve this if feasible. In the absence of clinical requirement for deep sedation, the *least awake* target sedation state will be 'brief eye contact made in response to voice' (RASS score of -2).

RASS score is recorded every 4 hours. The bedside algorithms recommend changes to sedation drug (according to group allocation) based on responses to RASS scores (see supplementary material). Patients receive opioid infusions for analgesia as clinically indicated.

For peer review only

Patients who require additional sedation or treatment, for example for agitation, receive this according to local practice.

Patients receiving norepinephrine or other vasopressors at enrolment can be commenced on lower doses of alpha2-agonist. This is suggested when the dose of norepinephrine is more than 0.15 micrograms/kg/min. Patients who develop hypotension and/or bradycardia in any treatment group are managed according to local practices using fluids and/or vasopressors. Sedative drugs can be reduced or stopped based on clinical discretion. In the alpha2-agonist groups, if the patient's heart rate decreases to less than 50/minute, the alpha2-agonist is stopped until the heart rate increases to greater than 50/minute. Re-starting the allocated sedative regimen is encouraged once cardiovascular instability has improved.

Weaning from mechanical ventilation

All patients have regular assessments and attempts to wean and discontinue MV throughout treatment. The approach used in individual ICUs and patients should adhere to 'best practice' principles for weaning from MV.

Data Collection

Data collection throughout the study is shown in table 3. Study data are recorded into a case report form (CRF), and transcribed into the web-based electronic CRF within the Edinburgh Clinical Trials Unit (ECTU). Automated query identification and checking is managed and resolved by the trial management team. A trial monitoring strategy by the sponsor tracks data quality at sites and triggers any corrective actions.

Withdrawals

Participants or their relatives can withdraw at any time. The three options for ongoing data collection will be: withdraw from intervention only, but follow-up and all data collection continues; intervention and follow-up only, with collection of routine data allowed; or withdrawal from all aspects of the trial and follow-up. Wherever possible primary outcome data are recorded for any withdrawn patient.

For peer review only

Table 3: assessments and measurements undertaken during the trial

	Pre-Randomisation	Baseline Data	Daily ICU Data Collection [1]	ICU Discharge [1]	Hospital Discharge [1]	30 days[2]	90 days[2]	180 days[2]
Screening for eligibility and consent, demographics, CHI/hospital number, RASS, CAM-ICU, final eligibility check	X							
Baseline data collection - baseline data, FCI, APACHE II, SOFA, RASS, CAM-ICU, PRE-DELIRIC (collected at 24 hours), EQ-5D-5L (assessed by proxy).		X						
Sepsis substudy only - 2 blood samples for inflammatory markers<br>• Baseline sample (within 12 hours post randomisation)<br>• 60 hour sample (within 48-72 hours post randomisation)		X						
Daily data collection during ICU stay until primary outcome confirmed or day 28 – clinical team (4hrly - RASS score and pain assessment; 12hrly – CAM-ICU, SQAT, co-operation and communication assessment)			X					
Daily data collection during ICU stay until primary outcome confirmed or day 28 – research team (MV data collection, IMP and drug usage, SOFA score, adverse event data collection)			X					
Assessment of comfort and communication by informant until primary outcome confirmed or day 28			X					
Adverse Event data collection until ICU discharge			X					
ICU and hospital discharge data				X	X			
Mortality			X	X	X	X	X	X
Intensive Care Experience Questionnaire (ICE-Q)							X	
Hospital Anxiety and Depression Scale (HADS) questionnaire								X
Impact of Events Scale – revised (IES-R)								X
Montreal Cognitive Assessment Tool (Telephone version - TMoCA)								X
Euroqol tool (EQ-5D-5L)						X	X	X
Recalled Euroqol tool (EQ-5D-5L)						X		
Health economic questionnaire (including hospital resource use and return to employment)							X	X

[1]These data are collected from the routine health record, except for the EG-5D-5L which is collected from the patient's proxy

[2-]These data are collected by research staff. Site teams confirm patient status, and then the research team contacts the patient using a mixed strategy including postal and telephone contact to maximise completion

Design and Analysis Plan

Analytic framework

The hierarchical analytic framework was devised to address key clinical effectiveness questions in a staged manner, to enable an efficient trial design that controls overall "family-wise" Type 1 error rate. The trial will determine whether alpha2-agonists are superior to current practice but also, if superiority is found, *which* agent is more clinically effective. We propose three analytic stages, where progression to hypothesis testing in sequential stages is dependent on preceding results (see figure 1). A detailed justification and explanation of these stages is included in the statistical analysis plan (see supplementary material).

For peer review only

Figure 1: Hierarchical design and analytics framework used in the A2B trial.

Note: All hypothesis tests performed using a one-sided 2.5% significance level in the original design

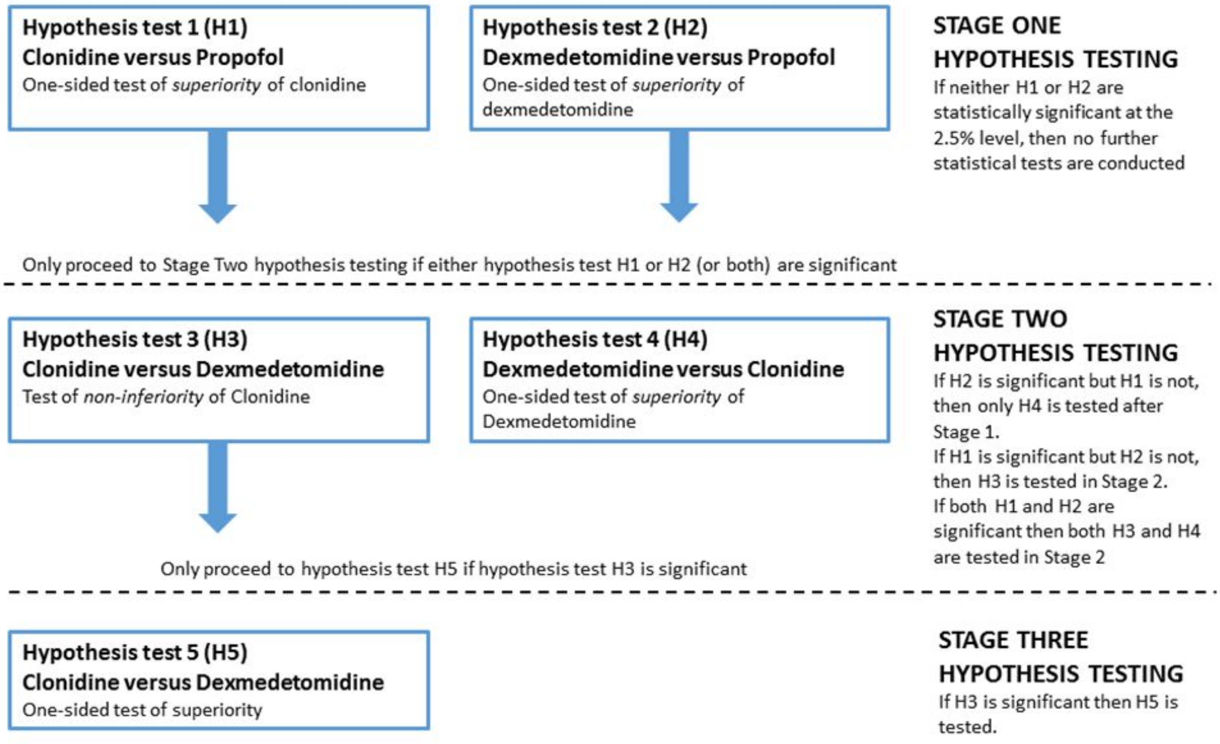

Further details regarding the original rationale for the study design and formation of the sample size calculations have been presented elsewhere[27].

Power and sample size during trial design

Based on clinical consensus, likely economic benefit, and the findings of systematic reviews, a minimum clinically important difference (MCID) of a mean difference in MV of 2 days was chosen for all superiority tests. For non-inferiority of clonidine versus dexmedetomidine, a non-inferiority margin of 1 day was chosen.

Sample size and power were modelled based on the analytic framework outlined in figure 1, which includes a hierarchical approach to hypothesis testing to control the "familywise" type I error to 5%. We used a large prospective data set from a sedation trial in 8 UK ICUs for modelling (N=708).[28] Based on this data set, we estimate that 53% of patients in the 'usual care' group will be extubated and around 14% will have died prior to extubation at 7 days.

Stage one: If either dexmedetomidine or clonidine are superior to usual care by an overall mean difference of 2 days in time to extubation, this translates to an estimated extubation rate of 63% in the dexmedetomidine or clonidine arm at 7 days. The death rate of 14% was

assumed to remain the same as for the usual care arm. Under these conditions, using nQuery version 8 software (log-rank test accounting for competing risks), a sample size of 550 per arm (1650 patients in total, 1328 extubation events across the three arms) has 99% power to detect hazard ratios of 1.37 indicating superiority of clonidine or dexmedetomidine over usual care, assuming a one-sided 2.5% significance level.

Stage two: These analyses are only undertaken if one or other or both of the Stage one tests are significant. For the non-inferiority test of clonidine relative to dexmedetomidine (test H3), the non-inferiority margin is a 1-day absolute mean difference in time to extubation. Based on the modelled dataset, a 1-day absolute mean difference translates into an estimated probability of 63% in the dexmedetomidine arm and 57% in the clonidine arm achieving the primary outcome at 7 days. This equates to an estimated non-inferiority margin on the hazard ratio scale of 0.83, assuming death rates in both arms are 14% at 7 days. Using this information in nQuery version 8 software (log-rank test accounting for competing risks), 550 patients per arm (1100 in total, 888 extubation events) provides 81% power to conclude non-inferiority of clonidine, using a one-sided 2.5% significance level. The power calculation for the superiority comparison of dexmedetomidine versus clonidine (test H4) is the same as that for Stage one. Simulation work was used to calculate the overall power of test H1 (clonidine superiority test versus propofol) *and* test H3 (clonidine non-inferiority test versus dexmedetomidine) being statistically significant using Fine and Gray proportional sub-distribution hazards regression analysis based on 2000 trials simulated from the real ICU dataset (mean 7 days on ventilation).[28] Assuming that dexmedetomidine and clonidine are both superior to usual care by an overall true mean difference of 2 days, and there is no difference between dexmedetomidine and clonidine, then a total sample size of 1650 (550 per group) provides 81% power of concluding non-inferiority of clonidine over dexmedetomidine (test H3) *and* concluding clonidine is superior to usual care (test H1) based on simulation, using a one-sided 2.5% significance level.

Stage three: The power calculation for the superiority comparison of clonidine versus dexmedetomidine (test H5), which will only be done if Stage one demonstrates superiority (tests H1 or H2) *and* clonidine is non-inferior to dexmedetomidine (test H3), is the same as that given in Stage 1.

Original sample size

We inflated sample size by 5% for loss to follow up for the primary outcome. The original trial sample size was therefore 1737 (579 patients per group).

Mortality

For the key outcome of mortality in ICU prior to extubation, a sample size of 550 per group provides 83% power to detect a difference in mortality of 7% (equivalent to a HR of approximately 1.5) using Cox regression assuming mortality in the usual care group is 23% and 16% in the clonidine/dexmedetomidine group, using a 2-sided 5% significance level.

For peer review only

Modifications to Sample Size due to impact of COVID19 pandemic

The COVID19 pandemic had a major impact on the trial progress and recruitment. In consultation with the funder, a modification to the original sample size was agreed in February 2023. The focus was on maintaining high power for the Stage one hypothesis testing, and included modelling the impact of a reduced sample size on the stage two test of non-inferiority of clonidine versus dexmedetomidine, plus the power for detecting an effect on mortality. Based on these investigations the sample size was reduced to 1437. This maintained 99% power for the Stage 1 comparisons of clonidine and dexmedetomidine versus propofol (H1 and H2), and also for the superiority comparison of dexmedetomidine versus clonidine if progression to Stage 2 testing occurs (H4). The main effect on power was for the non-inferiority comparison of clonidine versus dexmedetomidine (H3). For this comparison, in order to maintain 80% power when using the non-inferiority margin of 1 day, the significance level for test H3 was increased from 2.5% to 4%. This change to the hypothesis testing hierarchy meant that the upper limit on the familywise type I error rate increased from 5% to 6.5%. For the key secondary outcome of mortality, for the same 7% mortality difference, power decreased from 83% to 76%.

Pre-defined sub-group analyses

We plan four exploratory sub-group analyses, for patients with: [1] sepsis at enrolment; [2] higher delirium risk as defined by the PRE-DELIRIC delirium risk prediction score, using the version assessed at 24 hours post-admission[29]; [3] greater organ dysfunction, as measured by SOFA score, at randomisation (as this could differentially alter the safety profile of the three groups); and [4] age ≥64 years versus age <64 years (based on the relationship between age and mortality seen in the SPICE III trial)[20 21]

Statistical Analysis Plan (SAP)

An estimand was developed to deal how key intercurrent events will be dealt with in the analysis (see supplementary material). A detailed SAP has been finalised. The current version is included as an electronic supplement. The most up-to-date version can be found in the statistics section of the Trial Master File held in the ECTU.

Process Evaluation (PE)

A PE is included recognising that ICU sedation is a complex healthcare intervention that involves multiple healthcare professionals, assessing and delivering multiple agents using a series of interrelated activities, across multiple sites. The PE aims to: establish the extent to which the intervention is delivered as intended (fidelity, dose, and reach), over time and across different ICUs; ascertain how clinical staff understand and respond to the intervention, over time and across different ICUs; and, explore the importance of context (inter-ICU differences, changes over time) and determine factors (including organisational structure and processes) that affect intervention implementation and delivery. The detailed PE methods and analytic framework will be published separately.

For peer review only

Health economic evaluation

We will undertake a detailed analysis of the cost-effectiveness of dexmedetomidine, clonidine and usual care. We will estimate costs and cost-effectiveness for both the 'within-trial' period and over the expected lifetime of the patient. Costs will be assessed from the perspective of the NHS and personal social services (PSS). QALYs will be calculated based on the HRQoL and mortality data collected during the trial. Details of the health economic evaluation is included in the supplementary material.

Monitoring, Pharmacovigilance and Safety monitoring

Participants are monitored for adverse events (AEs) and serious adverse events (SAEs) until ICU discharge. Recording and reporting of AEs and SAEs will follow the Standard Operating Procedures of the trial sponsor (ACCORD). A trial monitoring plan designed by the study sponsor is in place, which includes study audits at study sites and within the trial management team and is carried out by independent sponsor QA personnel. All protocol amendments and their dissemination are managed according to sponsor SOPs compliant with UK Health Research Authority (HRA) guidance.

Ethics and dissemination

The trial is classified as a Clinical Trial of an Investigational Medicinal Product (CTIMP). The trial was reviewed and approved by the Scotland A REC (18/SS/0085), which for a CTIMP provides approval across the UK, and the Medicines and Healthcare products Regulatory Agency (MHRA). Each participating site undertakes local review and issues R&D approval according to UK HRA processes. As the trial involves incapacitated adults, all consent processes comply with the EU clinical trials regulations as written into UK law. Trial results will be disseminated through publications, conference presentations, and media engagement. Trial data will be uploaded to the EudraCT database (https://eudract.ema.europa.eu/).

Trial Management and Oversight

The trial is coordinated by a Project Management Group, including trial managers and coordinators, clinical investigators, and the statistics teams (see author contributions).

A Trial Steering Committee (TSC) is overseeing the conduct and progress of the trial, comprising an independent Chair, a PPI representative, and more than 70% independent clinical and methodology experts. All members sign a TSC charter.

An independent Data Monitoring Committee (DMC) is overseeing the safety of participants in the trial with an agreed DMC Charter to determine Terms of Reference. Given the caution around use in younger patients, the DMC is specifically monitoring safety and outcomes in younger versus older patient group throughout the trial.

The trial sponsor is the ACCORD joint research office of the University of Edinburgh and Lothian Health Board (https://www.accord.scot/). Indemnity for participants is provided through joint sponsorship by the University of Edinburgh and NHS Lothian.

For peer review only

All data are managed according to the General Data Protection Regulations (GDPR)

The funder and sponsor were not involved in design, but reviewed and approved the protocol and amendments. Neither have involvement in analysis, interpretation, or report writing. The sponsor is monitoring the trial.

Patient and Public Involvement

Patients were involved in the design of the trial and the production of trial materials. RG is a co-investigator and author. An independent patient representative sits on the TSC.

Current Status

The trial recruited its first patient in December 2018. Recruitment was severely affected by the COVID19 pandemic, with many sites closed for much of 2020-21. The trial re-opened in late 2020, but recruitment was affected by ICU pressures and research capacity during 2021-22. The funder requested a review of trial status and proposals to complete the trial in August 2022. The modelling work for a revised sample size, and considerations of plans to complete the trial recruitment, were concluded in October 2022. The final plan was approved by the funder and sponsor in February 2023, with a proposed recruitment end date of November 2023. Current protocol is version 7 (25th April 2023).

Author Contributions:

TSW, LMA, JN, CJW, RAP, NL, KK, B C-B, DFMcA, PD, MPW, ACG, GDP, MCR, BB, AMacL, RG, and VP designed the trial and led the funding application. All contributed to writing the detailed protocol. In addition JB, DH, AG, AMcD, and LE contributed to protocol development, implementation, monitoring, and amendments. The Process Evaluation was designed by LMA, LE, KK, BB, and TSW. The statistical design was led by RAP, JN, and CJW. The Health economic evaluation was designed by SM. TSW is Chief Investigator.

Funding statement:

This work is supported by the NIHR Health Technology Assessment Programme (HTA 16/93/01). The views expressed are those of the authors and not necessarily those of the NIHR or the Department of Health and Social Care. The NIHR Clinical Research Network (CRN) supports the trial.

Competing interests statement.

None of the authors report any relevant competing interests in relation to commercial companies or entities relevant to the A2B trial. No authors report any similar competing interests for spouses or children. Other than a clinical and academic interest in sedation management and its treatment, no authors declare any non-financial competing interests relevant to the A2B trial.

Data Access

Trial data will be held within the University of Edinburgh. Requests to access the full trial dataset will be considered on an individual request basis.

For peer review only

For peer review only

References

1. Adhikari NK, Fowler RA, Bhagwanjee S, et al. Critical care and the global burden of critical illness in adults. *Lancet (London, England)* 2010;376(9749):1339-46. doi: 10.1016/s0140-6736(10)60446-1 [published Online First: 2010/10/12]
2. Barr J, Fraser GL, Puntillo K, et al. Clinical practice guidelines for the management of pain, agitation, and delirium in adult patients in the intensive care unit. *Critical care medicine* 2013;41(1):263-306. doi: 10.1097/CCM.0b013e3182783b72 [published Online First: 2012/12/28]
3. Vincent JL, Shehabi Y, Walsh TS, et al. Comfort and patient-centred care without excessive sedation: the eCASH concept. *Intensive care medicine* 2016;42(6):962-71. doi: 10.1007/s00134-016-4297-4 [published Online First: 2016/04/15]
4. Reade MC, Finfer S. Sedation and delirium in intensive care. *The New England journal of medicine* 2014;370(16):1567. doi: 10.1056/NEJMc1402402 [published Online First: 2014/04/18]
5. Jackson DL, Proudfoot CW, Cann KF, et al. A systematic review of the impact of sedation practice in the ICU on resource use, costs and patient safety. *Critical care (London, England)* 2010;14(2):R59. doi: 10.1186/cc8956 [published Online First: 2010/04/13]
6. Aitken LM, Kydonaki K, Blackwood B, et al. Inconsistent relationship between depth of sedation and intensive care outcome: systematic review and meta-analysis. *Thorax* 2021;76(11):1089-98. doi: 10.1136/thoraxjnl-2020-216098 [published Online First: 2021/04/17]
7. Nikayin S, Rabiee A, Hashem MD, et al. Anxiety symptoms in survivors of critical illness: a systematic review and meta-analysis. *General hospital psychiatry* 2016;43:23-29. doi: 10.1016/j.genhosppsych.2016.08.005 [published Online First: 2016/11/01]
8. Parker AM, Sricharoenchai T, Raparla S, et al. Posttraumatic stress disorder in critical illness survivors: a metaanalysis. *Critical care medicine* 2015;43(5):1121-9. doi: 10.1097/ccm.0000000000000882 [published Online First: 2015/02/06]
9. Rabiee A, Nikayin S, Hashem MD, et al. Depressive Symptoms After Critical Illness: A Systematic Review and Meta-Analysis. *Critical care medicine* 2016;44(9):1744-53. doi: 10.1097/ccm.0000000000001811 [published Online First: 2016/05/07]
10. Wade D, Hardy R, Howell D, et al. Identifying clinical and acute psychological risk factors for PTSD after critical care: a systematic review. *Minerva anestesiologica* 2013;79(8):944-63. [published Online First: 2013/04/06]
11. Aitken LM, Castillo MI, Ullman A, et al. What is the relationship between elements of ICU treatment and memories after discharge in adult ICU survivors? *Australian critical care : official journal of the Confederation of Australian Critical Care Nurses* 2016;29(1):5-14; quiz 15. doi: 10.1016/j.aucc.2015.11.004 [published Online First: 2016/01/19]
12. Gertler R, Brown HC, Mitchell DH, et al. Dexmedetomidine: a novel sedative-analgesic agent. *Proceedings (Baylor University Medical Center)* 2001;14(1):13-21. [published Online First: 2005/12/22]
13. Nguyen V, Tiemann D, Park E, et al. Alpha-2 Agonists. *Anesthesiology clinics* 2017;35(2):233-45. doi: 10.1016/j.anclin.2017.01.009 [published Online First: 2017/05/21]
14. Li A, Yuen VM, Goulay-Dufay S, et al. Pharmacokinetics and pharmacodynamics of dexmedetomidine. *Drug development and industrial pharmacy* 2016;42(12):1917-27. doi: 10.1080/03639045.2016.1232727 [published Online First: 2016/09/07]
15. Jamadarkhana S, Gopal S. Clonidine in adults as a sedative agent in the intensive care unit. *Journal of anaesthesiology, clinical pharmacology* 2010;26(4):439-45. [published Online First: 2011/05/07]
16. Luz M, Brandão Barreto B, de Castro REV, et al. Practices in sedation, analgesia, mobilization, delirium, and sleep deprivation in adult intensive care units (SAMDS-ICU): an international survey before and during the COVID-19 pandemic. *Ann Intensive Care* 2022;12(1):9. doi: 10.1186/s13613-022-00985-y [published Online First: 20220204]
17. Wang JG, Belley-Cote E, Burry L, et al. Clonidine for sedation in the critically ill: a systematic review and meta-analysis. *Critical care (London, England)* 2017;21(1):75. doi: 10.1186/s13054-017-1610-8 [published Online First: 2017/03/24]
18. Lewis K, Alshamsi F, Carayannopoulos KL, et al. Dexmedetomidine vs other sedatives in critically ill mechanically ventilated adults: a systematic review and meta-analysis of randomized trials. *Intensive Care Med* 2022;48(7):811-40. doi: 10.1007/s00134-022-06712-2 [published Online First: 20220601]
19. Heybati K, Zhou F, Ali S, et al. Outcomes of dexmedetomidine versus propofol sedation in critically ill adults requiring mechanical ventilation: a systematic review and meta-analysis of randomised controlled

For peer review only

trials. *Br J Anaesth* 2022;129(4):515-26. doi: 10.1016/j.bja.2022.06.020 [published Online First: 20220810]

20. Shehabi Y, Howe BD, Bellomo R, et al. Early Sedation with Dexmedetomidine in Critically Ill Patients. *N Engl J Med* 2019;380(26):2506-17. doi: 10.1056/NEJMoa1904710 [published Online First: 2019/05/22]
21. Shehabi Y, Serpa Neto A, Howe BD, et al. Early sedation with dexmedetomidine in ventilated critically ill patients and heterogeneity of treatment effect in the SPICE III randomised controlled trial. *Intensive Care Med* 2021;47(4):455-66. doi: 10.1007/s00134-021-06356-8 [published Online First: 2021/03/10]
22. Agency EM. Dexmedetomidine: Increased risk of mortality in intensive care unit

(ICU) patients ≤65 years 2022 [Available from: https://www.ema.europa.eu/en/documents/dhpc/direct-healthcare-professional-communication-dhpc-dexmedetomidine-increased-risk-mortality-intensive_en.pdf accessed June 5th 2023.

23. Skivington K, Matthews L, Simpson SA, et al. A new framework for developing and evaluating complex interventions: update of Medical Research Council guidance. *Bmj* 2021;374:n2061. doi: 10.1136/bmj.n2061 [published Online First: 2021/10/02]
24. Walsh TS, Kydonaki K, Lee RJ, et al. Development of Process Control Methodology for Tracking the Quality and Safety of Pain, Agitation, and Sedation Management in Critical Care Units. *Critical care medicine* 2016;44(3):564-74. doi: 10.1097/ccm.0000000000001463 [published Online First: 2016/02/24]
25. Ely EW, Inouye SK, Bernard GR, et al. Delirium in mechanically ventilated patients: validity and reliability of the confusion assessment method for the intensive care unit (CAM-ICU). *Jama* 2001;286(21):2703-10. [published Online First: 2001/12/26]
26. Rattray J, Johnston M, Wildsmith JA. The intensive care experience: development of the ICE questionnaire. *Journal of advanced nursing* 2004;47(1):64-73. doi: 10.1111/j.1365-2648.2004.03066.x [published Online First: 2004/06/10]
27. Parker RA. Overcoming Obstacles to Deriving Sample Size Calculations: Experiences of a Biostatistician. *Sage Research Methods Cases: Medicine and Health* 2020 doi: https://doi.org/10.4135/9781529731699 [published Online First: March 23, 2020]
28. Walsh TS, Kydonaki K, Antonelli J, et al. Staff education, regular sedation and analgesia quality feedback, and a sedation monitoring technology for improving sedation and analgesia quality for critically ill, mechanically ventilated patients: a cluster randomised trial. *The Lancet Respiratory medicine* 2016;4(10):807-17. doi: 10.1016/s2213-2600(16)30178-3 [published Online First: 2016/07/31]
29. van den Boogaard M, Pickkers P, Slooter AJ, et al. Development and validation of PRE-DELIRIC (PREdiction of DELIRium in ICu patients) delirium prediction model for intensive care patients: observational multicentre study. *BMJ (Clinical research ed)* 2012;344:e420. doi: 10.1136/bmj.e420 [published Online First: 2012/02/11]

For peer review only

For peer review only

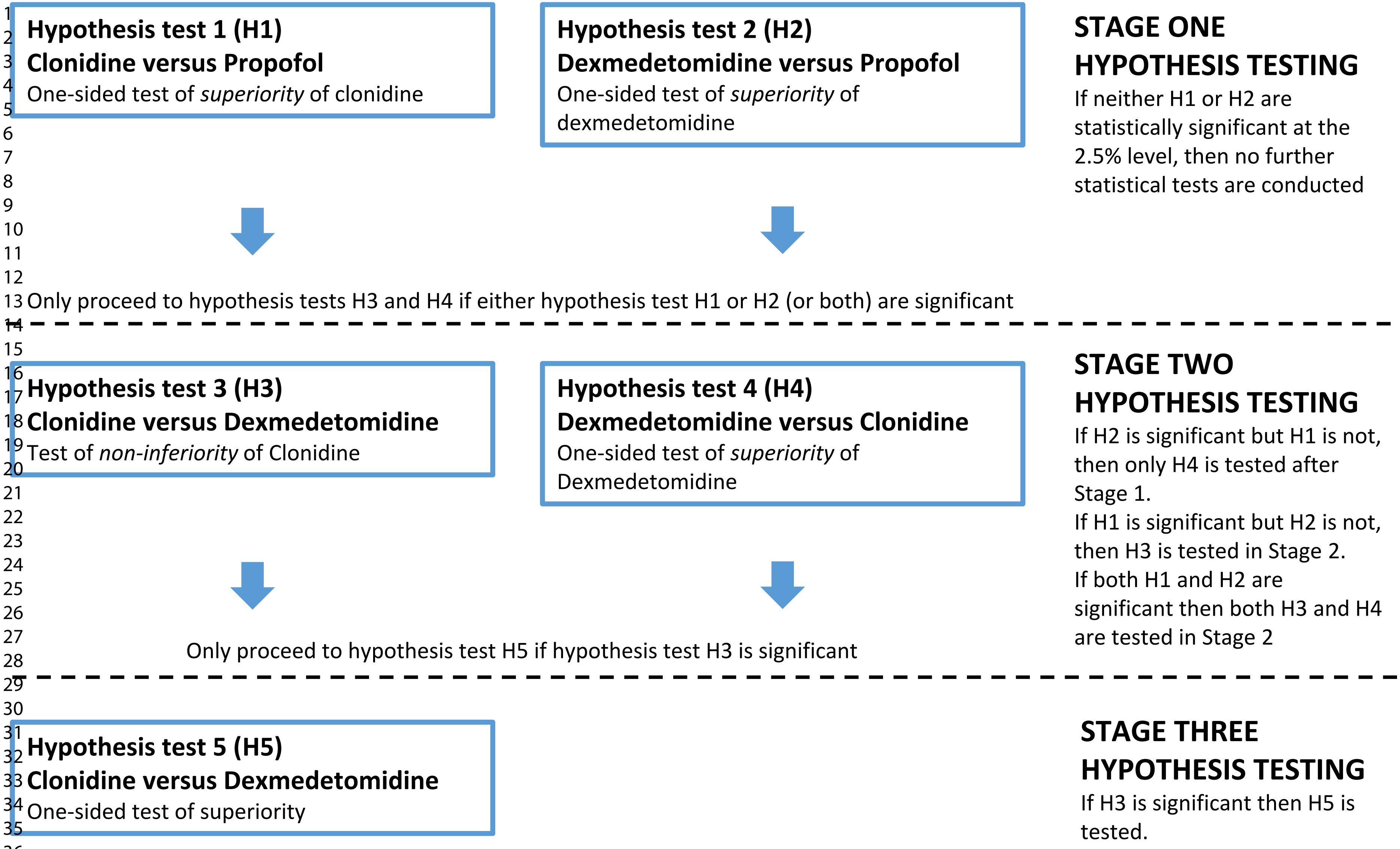
Hypothesis test 1 (H1)
Clonidine versus Propofol
One-sided test of superiority of clonidine
Hypothesis test 2 (H2)
Dexmedetomidine versus Propofol
One-sided test of superiority of dexmedetomidine
STAGE ONE HYPOTHESIS TESTING
If neither H1 or H2 are statistically significant at the 2.5% level, then no further statistical tests are conducted
Only proceed to hypothesis tests H3 and H4 if either hypothesis test H1 or H2 (or both) are significant
Hypothesis test 3 (H3)
Clonidine versus Dexmedetomidine
Test of non-inferiority of Clonidine
Hypothesis test 4 (H4)
Dexmedetomidine versus Clonidine
One-sided test of superiority of Dexmedetomidine
STAGE TWO HYPOTHESIS TESTING
If H2 is significant but H1 is not, then only H4 is tested after Stage 1.
If H1 is significant but H2 is not, then H3 is tested in Stage 2.
If both H1 and H2 are significant then both H3 and H4 are tested in Stage 2
Only proceed to hypothesis test H5 if hypothesis test H3 is significant
Hypothesis test 5 (H5)
Clonidine versus Dexmedetomidine
One-sided test of superiority
STAGE THREE HYPOTHESIS TESTING
If H3 is significant then H5 is tested.

Appendix

Contents

Appendix....1

Figure illustrating the consent process utilised for enrolling patients in the absence of patient capacity....2

Example of Consent Form – Personal Legal Representative Consent form....2

Example of consent form....3

Weight-based drug dosing algorithms used in the A2B trial....5

Clonidine Drug Regimen....5

Dexmedetomidine Drug regimen....6

Clinical management algorithms to guide dosing of intervention drugs in the A2B trial....7

CLONIDINE Flowchart....8

Dexmedetomidine Flowchart....10

Usual Care (propofol) flowchart....12

Trial Estimand (see also Statistical Analysis Plan)....14

Health Economic Evaluation....16

Overview....16

Within-trial analysis....16

Lifetime analysis....17

For peer review only

Figure illustrating the consent process utilised for enrolling patients in the absence of patient capacity

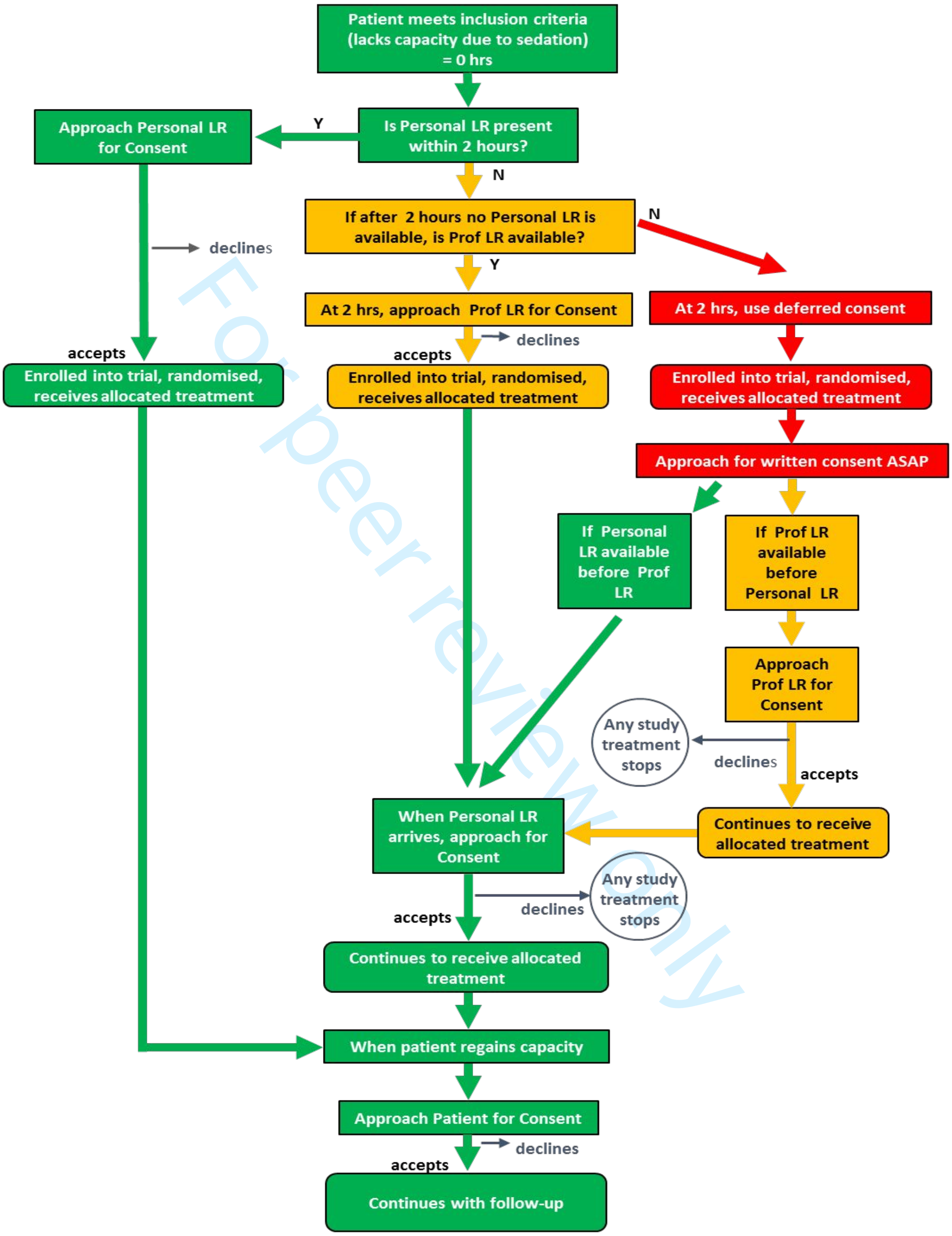

Personal LR = Personal Legal Representative Prof LR = Professional Legal Representative

Example of Consent Form – Personal Legal Representative Consent form

(Additional consent forms used for Professional Legal Representative Consent, and for Patient Consent to remain in trial (once regained capacity)

For peer review only - http://bmjopen.bmj.com/site/about/guidelines.xhtml

Example of consent form

Participant ID:		Centre ID	

CONSENT FORM
England, Wales, Northern Ireland
Guardian or Nearest Relative
(Personal Legal Representative – Pre randomisation)

ALPHA-2 AGONISTS FOR SEDATION TO PRODUCE BETTER OUTCOMES FROM CRITICAL ILLNESS ('A2B TRIAL')

Please **initial** box

1. I confirm that I have read and understand the Personal LR Pre-randomisation information sheet England/Wales/Northern Ireland (18MAY2023 V2.0) for the above study. I have had the opportunity to consider the information, ask questions and have had these questions answered satisfactorily. ☐
2. I understand that my relative's participation is voluntary and that I am free to withdraw my consent at any time without giving any reason and without my relative's medical care and/or legal rights being affected. ☐
3. I give permission for the research team to access my relative's medical records for the purposes of this research study ☐
4. I understand that relevant sections of my relative's medical notes and data collected during the study may be looked at by individuals from the Sponsor (University of Edinburgh and/or NHS Lothian), from the NHS organisation or other regulatory authorities where it is relevant to their taking part in this research. I give permission for these individuals to have access to my relative's data and/or medical records. ☐
5. I give permission for my relative's personal information (including name, address, date of birth, telephone number and consent form) to be passed to the University of Edinburgh and Edinburgh Clinical Trials Unit for administration of the study and follow-up purposes. ☐
6. I give permission for my relative's hospital number to be collected and passed to the University of Edinburgh and Edinburgh Clinical Trials Unit. ☐
7. I agree that the information held and maintained by NHS Digital and other central UK NHS bodies may be used to provide information about my relative's health status. ☐
8. I agree to my relative taking part in the substudy which would involve giving two 20ml blood samples which will be used to study inflammation in the blood and for genetic DNA analysis. Yes ☐ No ☐
9. I give permission for DNA analysis, including whole genome sequencing, to be conducted on my relative's samples Yes ☐ No ☐
10. I agree that information collected about my relative can be used to support other research in the future, and may be shared anonymously with other researchers. Yes ☐ No ☐
11. I agree that my relative's blood and DNA samples can be used to support other research in the future, and may be shared anonymously with other researchers. Yes ☐ No ☐
12. I agree to provide my opinion on my relative's level of comfort and my ability to communicate with them and I give my permission for this data to be used. ☐
13. I agree to my relative taking part in the above study ☐

For peer review only - http://bmjopen.bmj.com/site/about/guidelines.xhtml

Participant ID:			Centre ID

I understand that my relative's data will not be shared beyond those noted on the consent form and that access will be managed via a secure system.

Please **initial** box.

☐ I confirm that I am Personal Legal Representative for ______________________________

Relationship to participant ______________________________

____
Name of Person Giving Consent Date Time Signature

____
Name of person receiving consent Date Time Signature

For peer review only

Weight-based drug dosing algorithms used in the A2B trial

Clonidine Drug Regimen

Weight Based Infusion Rates in mls per hour for Clonidine 15micrograms per ml

	Patient's weight (actual) in kilograms											
Dose in micrograms per kilogram per hour	45	50	55	60	65	70	75	80	85	90	95	≥100
**0.1**	0.3	0.3	0.4	0.4	0.4	0.5	0.5	0.5	0.6	0.6	0.6	0.7
**0.2**	0.6	0.7	0.7	0.8	0.9	0.9	1.0	1.1	1.1	1.2	1.3	1.3
**0.3**	0.9	1.0	1.1	1.2	1.3	1.4	1.5	1.6	1.7	1.8	1.9	2.0
**0.4**	1.2	1.3	1.5	1.6	1.7	1.9	2.0	2.1	2.3	2.4	2.5	2.7
**0.5**	1.5	1.7	1.8	2.0	2.2	2.3	2.5	2.7	2.8	3.0	3.2	3.3
**0.6**	1.8	2.0	2.2	2.4	2.6	2.8	3.0	3.2	3.4	3.6	3.8	4.0
**0.7**	2.1	2.3	2.6	2.8	3.0	3.3	3.5	3.7	4.0	4.2	4.4	4.7
**0.8**	2.4	2.7	2.9	3.2	3.5	3.7	4.0	4.3	4.5	4.8	5.1	5.3
**0.9**	2.7	3.0	3.3	3.6	3.9	4.2	4.5	4.8	5.1	5.4	5.7	6.0
**1.0**	3.0	3.3	3.7	4.0	4.3	4.7	5.0	5.3	5.7	6.0	6.3	6.7
**1.1**	3.3	3.7	4.0	4.4	4.8	5.1	5.5	5.9	6.2	6.6	7.0	7.3
**1.2**	3.6	4.0	4.4	4.8	5.2	5.6	6.0	6.4	6.8	7.2	7.6	8.0
**1.3**	3.9	4.3	4.8	5.2	5.6	6.1	6.5	6.9	7.4	7.8	8.2	8.7
**1.4**	4.2	4.7	5.1	5.6	6.1	6.5	7.0	7.5	7.9	8.4	8.9	9.3
**1.5**	4.5	5.0	5.5	6.0	6.5	7.0	7.5	8.0	8.5	9.0	9.5	10.0
**1.6**	4.8	5.3	5.9	6.4	6.9	7.5	8.0	8.5	9.1	9.6	10.1	10.7
**1.7**	5.1	5.7	6.2	6.8	7.4	7.9	8.5	9.1	9.6	10.2	10.8	11.3
**1.8**	5.4	6.0	6.6	7.2	7.8	8.4	9.0	9.6	10.2	10.8	11.4	12.0
**1.9**	5.7	6.3	7.0	7.6	8.2	8.9	9.5	10.1	10.8	11.4	12.0	12.7
**2.0**	6.0	6.7	7.3	8.0	8.7	9.3	10.0	10.7	11.3	12.0	12.7	13.3

For patients who weigh less than 45kg, please contact the trial management team for specific advice prior to randomisation.

Patients who weigh greater than 100kg will be dosed using the regimen suggested for 100kg weight. Please contact the trial management team for advice if required prior to randomisation.

Dexmedetomidine Drug regimen

Weight Based Infusion Rates in mls per hour for Dexmedetomidine 8 micrograms per ml

Dose in micrograms per kilogram per hour	Patient's weight (actual) in kilograms											
	45	50	55	60	65	70	75	80	85	90	95	≥100
**0.1**	0.6	0.6	0.7	0.8	0.8	0.9	0.9	1.0	1.1	1.1	1.2	1.3
**0.2**	1.1	1.3	1.4	1.5	1.6	1.8	1.9	2.0	2.1	2.3	2.4	2.5
**0.3**	1.7	1.9	2.1	2.3	2.4	2.6	2.8	3.0	3.2	3.4	3.6	3.8
**0.4**	2.3	2.5	2.8	3.0	3.3	3.5	3.8	4.0	4.3	4.5	4.8	5.0
**0.5**	2.8	3.1	3.4	3.8	4.1	4.4	4.7	5.0	5.3	5.6	5.9	6.3
**0.6**	3.4	3.8	4.1	4.5	4.9	5.3	5.6	6.0	6.4	6.8	7.1	7.5
**0.7**	3.9	4.4	4.8	5.3	5.7	6.1	6.6	7.0	7.4	7.9	8.3	8.8
**0.8**	4.5	5.0	5.5	6.0	6.5	7.0	7.5	8.0	8.5	9.0	9.5	10.0
**0.9**	5.1	5.6	6.2	6.8	7.3	7.9	8.4	9.0	9.6	10.1	10.7	11.3
**1.0**	5.6	6.3	6.9	7.5	8.1	8.8	9.4	10.0	10.6	11.3	11.9	12.5
**1.1**	6.2	6.9	7.6	8.3	8.9	9.6	10.3	11.0	11.7	12.4	13.1	13.8
**1.2**	6.8	7.5	8.3	9.0	9.8	10.5	11.3	12.0	12.8	13.5	14.3	15.0
**1.3**	7.3	8.1	8.9	9.8	10.6	11.4	12.2	13.0	13.8	14.6	15.4	16.3
**1.4**	7.9	8.8	9.6	10.5	11.4	12.3	13.1	14.0	14.9	15.8	16.6	17.5

For patients who weigh less than 45kg, please contact the trial management team for specific advice prior to randomisation.

Patients who weigh greater than 100kg will be dosed using the regimen suggested for 100kg weight. Please contact the trial management team for advice if required prior to randomisation.

For peer review only - http://bmjopen.bmj.com/site/about/guidelines.xhtml

Clinical management algorithms to guide dosing of intervention drugs in the A2B trial

For peer review only

For peer review only - http://bmjopen.bmj.com/site/about/guidelines.xhtml

CLONIDINE Flowchart

An ICU Sedation Study

Clonidine Group Sedation Flowchart

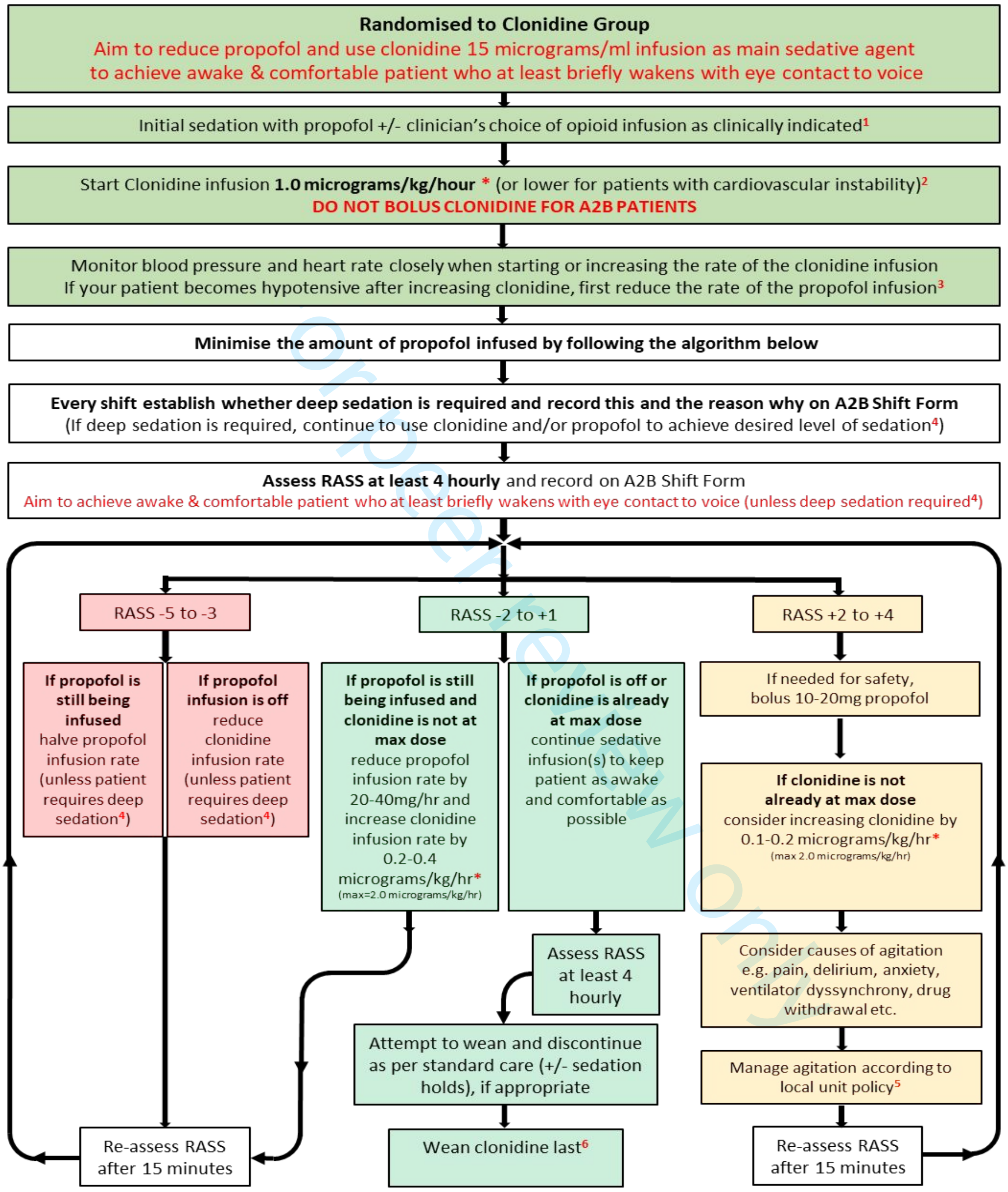

* See A2B Clonidine infusion table for mls/hr infusion rates for patient weight

[1] additional opioid boluses can be given as required

[2] if on more than 0.15 micrograms/kg/min noradrenaline to maintain target MAP, use lower starting dose initially

[3] PTO for advice on managing severe bradycardia or hypotension on the reverse of this page

[4] PTO for deep sedation advice on the reverse of this page

[5] dexmedetomidine should not be prescribed for the Clonidine group

[6] PTO for weaning advice on the reverse of this page

An ICU Sedation Study

For peer review only - http://bmjopen.bmj.com/site/about/guidelines.xhtml

Clonidine Group Sedation

An ICU Sedation Study

TARGET: most awake and comfortable state considered safe (the least awake target will be "briefly wakens with eye contact to voice")

- Primary sedative agent is **CLONIDINE** (diluted with 5% glucose or 0.9% NaCl solution to a concentration of **15 micrograms per ml**)
- **Aim to reduce/stop propofol infusion.**
- All patients should receive opioid infusions for analgesia as clinically indicated at the discretion of clinical teams.
- **NB Clonidine has analgesic properties, so it may be possible to reduce opioids if pain is well controlled.**

Drugs you should not give:

- **Dexmedetomidine should <u>not</u> be used as first line sedation during the intervention period.**

How to manage agitation (RASS +2 TO +4)

- Maintain patient/staff safety by bolusing propofol if needed.
- Once agitation is under control, try to identify and manage the cause, using local unit policy for the management of pain/delirium/anxiety/withdrawal etc.
- Anticipate and avoid agitation; use opioid analgesia in advance of uncomfortable or painful interventions or procedures.

What to do if my patient develops severe bradycardia (HR<50 beats per minute)

- **If your patient's heart rate decreases to less than 50 beats per minute, clonidine should be temporarily stopped.**
- NB Clonidine's effect on heart rate can take several hours to resolve, so stopping the clonidine infusion may not immediately resolve bradycardia.
- Seek advice from medical staff who can review and prescribe, glycopyrronium, atropine, dobutamine, adrenaline or other agents, as per your ICU policy.
- Once heart rate is maintained above 50 beats per minute, clonidine should be re-commenced at a dose appropriate to the sedation target, but caution should be used when the clinical target is deep sedation.

What to do if my patient becomes hypotensive

- Hypotension should be treated as per local unit policy. Continuous fluid infusions, fluid challenges and vasopressor infusions are all permitted for patients in the A2B Trial.
- If changes to sedation are required as a result of hypotension, propofol should be decreased before clonidine, unless clinically contraindicated (e.g. if patient requires propofol for neuromuscular blockade).
- Continue clonidine infusion unless causing haemodynamic compromise, such that target MAP cannot be maintained with 0.15 micrograms/kg/min of noradrenaline. If haemodynamically compromised, consider halving the rate of the clonidine infusion, then halving again, or stopping, as needed. Clonidine can be restarted/increased once the patient is more stable, at the discretion of medical staff.
- NB Clonidine's effect on heart rate can take several hours to resolve, so stopping the clonidine infusion may not immediately resolve hypotension.

What if my patient requires deep sedation (RASS -3 TO -5) e.g. for neuromuscular blockade (muscle relaxant)?

- If medical staff have asked to keep your patient deeply sedated, please record the reason deep sedation was requested on the A2B Shift Form.
- **Patients who require neuromuscular blockade after randomisation must receive adequate sedation as per standard care, at the discretion of the treating clinician, to prevent awareness during paralysis. Clonidine alone may not provide adequate sedation to prevent awareness.**
- It is suggested that clonidine is titrated up to the maximum tolerated dose, but if additional sedative drugs are needed to achieve target deep sedation this can be achieved with propofol or benzodiazepine.
- When deep sedation is no longer requested by the caring clinician, aim to use clonidine as main sedative agent as per flowchart over page. It is suggested that any propofol or benzodiazepine sedation is decreased and stopped prior to reducing the dose of clonidine.

What if my patient needs an operative procedure/CT scan/MRI etc.?

- Increase sedation for transfer if needed, using clonidine and/or propofol.
- If required, anaesthesia should be administered as per local perioperative guidelines.
- Continue clonidine infusion unless haemodynamically compromised, in which case consider halving infusion rate and halving again or stopping, as needed.
- Following the procedure, again aim to reduce propofol and use clonidine as main sedative agent as per flowchart over page.

Weaning and discontinuing Clonidine

- **Clonidine can be abruptly discontinued, but slower weaning should be used if withdrawal reactions occur (rebound hypertension or tachycardia, agitation, sweating etc.). However, this should not delay weaning from ventilation as clonidine is relatively free from respiratory depressive effects, so can be safely continued post-extubation.**

PTO for Clonidine Group Sedation Flowchart on reverse of this page

For peer review only - http://bmjopen.bmj.com/site/about/guidelines.xhtml

Dexmedetomidine Flowchart

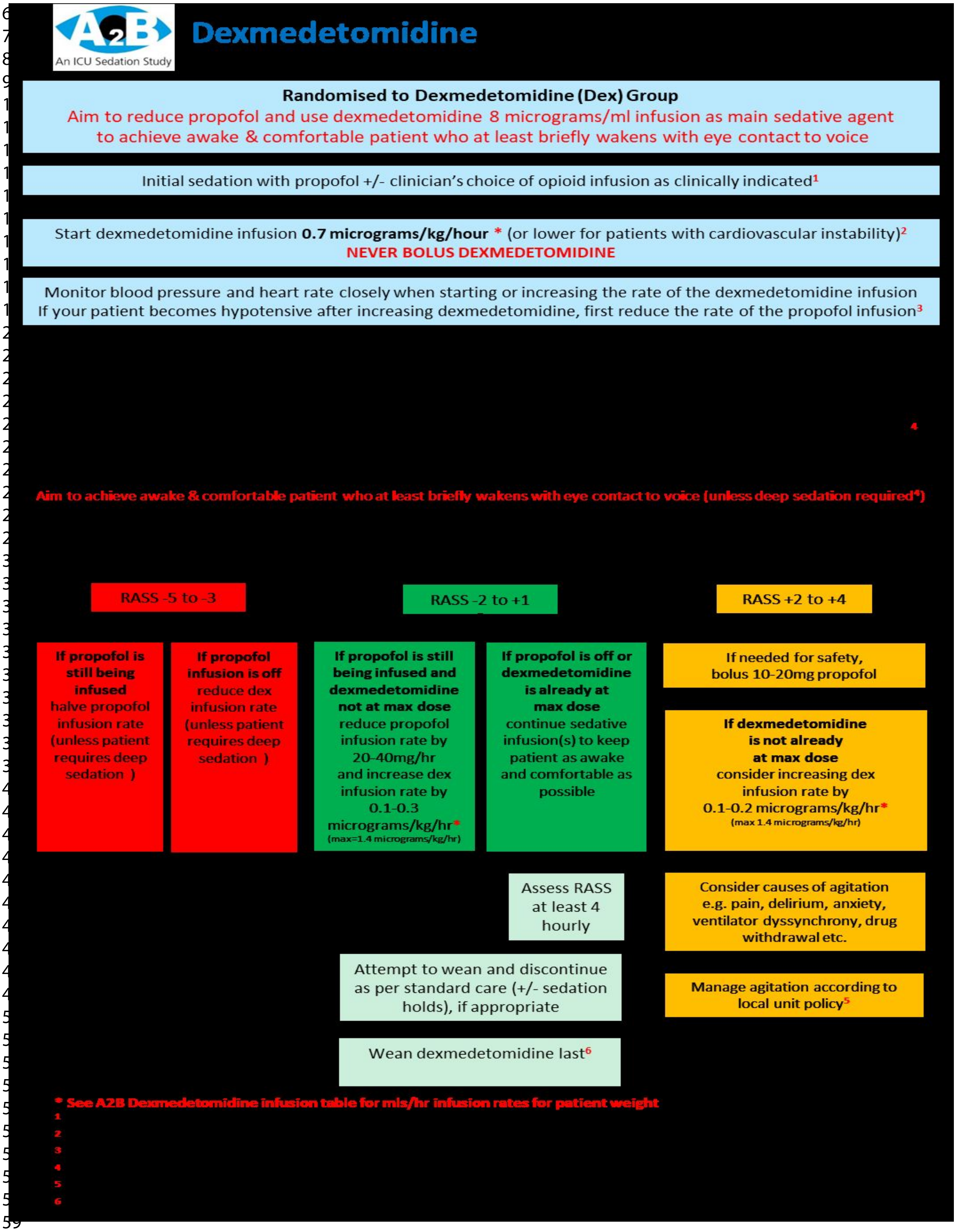

Dexmedetomidine (Dex) Group Sedation

An ICU Sedation Study

TARGET: most awake and comfortable state considered safe (the least awake target will be "briefly wakens with eye contact to voice")

- Primary sedative agent is **DEXMEDETOMIDINE** (diluted with 5% glucose or 0.9% NaCl solution to a concentration of **8 micrograms per ml**)
- **Aim to reduce/stop propofol infusion.**
- All patients should receive opioid infusions for analgesia as clinically indicated at the discretion of clinical teams.
- **NB Dexmedetomidine has analgesic properties, so it may be possible to reduce opioids if pain is well controlled.**

Drugs you should not give:

- **Clonidine should not be used as first line sedation during the intervention period.**

How to manage agitation (RASS +2 TO +4)

- Maintain patient/staff safety by bolusing propofol if needed.
- Once agitation is under control, try to identify and manage the cause, using local unit policy for the management of pain/delirium/anxiety/withdrawal etc.
- Anticipate and avoid agitation; use opioid analgesia in advance of uncomfortable or painful interventions or procedures.

What to do if my patient develops severe bradycardia (HR<50 beats per minute)

- **If your patient's heart rate decreases to less than 50 beats per minute, dexmedetomidine should be temporarily stopped.**
- NB Dexmedetomidine's effect on heart rate can take several hours to resolve, so stopping the dexmedetomidine infusion may not immediately resolve bradycardia.
- Seek advice from medical staff who can review and prescribe, glycopyrronium, atropine, dobutamine, adrenaline or other agents, as per your ICU policy.
- Once heart rate is maintained above 50 beats per minute, dexmedetomidine should be re-commenced at a dose appropriate to the sedation target, but caution should be used when the clinical target is deep sedation.

What to do if my patient becomes hypotensive

- Hypotension should be treated as per local unit policy. Continuous fluid infusions, fluid challenges and vasopressor infusions are all permitted for patients in the A2B Trial.
- If changes to sedation are required as a result of hypotension, propofol should be decreased before dexmedetomidine, unless clinically contraindicated (e.g. if patient requires propofol for neuromuscular blockade).
- Continue dexmedetomidine infusion unless causing haemodynamic compromise, such that target MAP cannot be maintained with 0.15 micrograms/kg/min of noradrenaline. If haemodynamically compromised, consider halving the rate of the dexmedetomidine infusion, then halving again, or stopping, as needed. Dexmedetomidine can be restarted/increased once the patient is more stable, at the discretion of medical staff.
- NB Dexmedetomidine's effect on heart rate can take several hours to resolve, so stopping the dexmedetomidine infusion may not immediately resolve hypotension.

What if my patient requires deep sedation (RASS -3 TO -5) e.g. for neuromuscular blockade (muscle relaxant)?

- If medical staff have asked to keep your patient deeply sedated, please record the reason deep sedation was requested on the A2B Shift Form.
- **Patients who require neuromuscular blockade after randomisation must receive adequate sedation as per standard care at the discretion of the treating clinician, to prevent awareness during paralysis. Dexmedetomidine alone may not provide adequate sedation to prevent awareness.**
- It is suggested that dexmedetomidine is titrated up to the maximum tolerated dose, but if additional sedative drugs are needed to achieve target deep sedation this can be achieved with propofol or benzodiazepine.
- When deep sedation is no longer requested by the caring clinician, aim to use dexmedetomidine as main sedative agent as per flowchart over page. It is suggested that any propofol or benzodiazepine sedation is decreased and stopped prior to reducing the dose of dexmedetomidine.

What if my patient needs an operative procedure/CT scan/MRI etc.?

- Increase sedation for transfer if needed, using dexmedetomidine and/or propofol.
- If required, anaesthesia should be administered as per local perioperative guidelines.
- Continue dexmedetomidine infusion unless haemodynamically compromised, in which case consider halving infusion rate and halving again or stopping, as needed.
- Following the procedure, again aim to reduce propofol and use dexmedetomidine as main sedative agent as per flowchart over page.

Weaning and discontinuing Dexmedetomidine

- **Dexmedetomidine can be abruptly discontinued, but slower weaning should be used if withdrawal reactions occur (rebound hypertension or tachycardia, agitation, sweating etc.). However, this should not delay weaning from ventilation as dexmedetomidine is relatively free from respiratory depressive effects, so can be safely continued post-extubation.**

PTO for Dexmedetomidine Group Sedation Flowchart on reverse of this page

For peer review only - http://bmjopen.bmj.com/site/about/guidelines.xhtml

Usual Care (propofol) flowchart

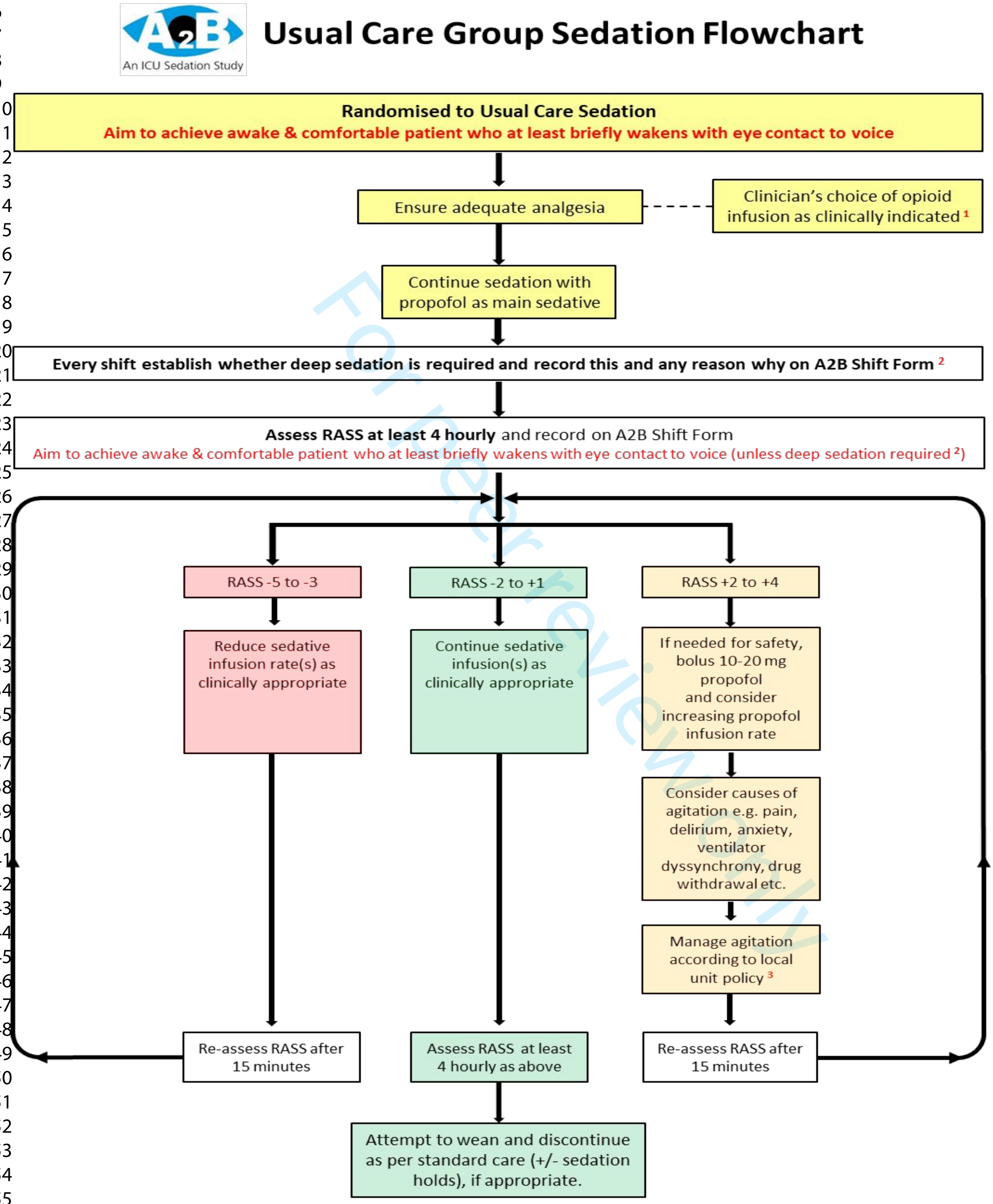

[1] additional opioid boluses can be given as required
[2] PTO for deep sedation advice on the reverse of this page
[3] See advice in "Drugs you should not give" on the reverse of this page before using Dexmedetomidine or Clonidine

For peer review only - http://bmjopen.bmj.com/site/about/guidelines.xhtml

Usual Care Group Sedation

TARGET: most awake and comfortable state considered safe (the least awake target will be "briefly wakens with eye contact to voice")

- Primary sedative agent is **PROPOFOL** (either **1% or 2%**).
- All patients should receive opioid infusions for analgesia as clinically indicated at the discretion of clinical teams

Drugs you should not give:

- **Neither clonidine nor dexmedetomidine should be used as first line sedation during the intervention period.**
- However, either clonidine or dexmedetomidine can be used at the discretion of the clinical teams for specific indications such as agitation, severe delirium or during difficult weaning.

How to manage agitation (RASS +2 TO +4)

- Maintain patient/staff safety by bolusing propofol if needed.
- Once agitation is under control, try to identify and manage the cause, using local unit policy for the management of pain/delirium/anxiety/withdrawal etc.
- Anticipate and avoid agitation; use opioid analgesia in advance of uncomfortable or painful interventions or procedures.

What to do if my patient develops severe bradycardia (HR<50/min)

- Seek advice from medical staff who can review and prescribe, glycopyrronium, atropine, dobutamine, adrenaline or other agents, as per your ICU policy.

What to do if my patient becomes hypotensive

- Hypotension should be treated as per local unit policy. Continuous fluid infusions, fluid challenges and vasopressor infusions are all permitted for patients in the A2B Trial.

What if my patient requires deep sedation (RASS -3 TO -5) e.g. for neuromuscular blockade (muscle relaxant)?

- If medical staff have asked to keep your patient deeply sedated, please record the reason deep sedation was requested on the A2B Shift Form.
- **Patients who require neuromuscular blockade after randomisation must receive adequate sedation as per standard care at the discretion of the treating clinician, to prevent awareness during paralysis.**
- If additional sedative drugs are needed to achieve target deep sedation this can be achieved with benzodiazepine.
- When deep sedation is no longer requested by the caring clinician, aim to use propofol as main sedative agent as per flowchart over page.

What if my patient needs an operative procedure?

- Increase sedation for transfer if needed.
- If required, anaesthesia should be administered as per local perioperative guidelines.
- Following the operative procedure, again aim to use propofol as main sedative agent as per flowchart over page.

What if my patient needs an operative procedure/CT scan/MRI etc.?

- Increase sedation for transfer to theatre if needed using propofol.
- If required, anaesthesia should be administered as per local perioperative guidelines.

PTO for Usual Care Group Sedation Flowchart on reverse of this page

For peer review only - http://bmjopen.bmj.com/site/about/guidelines.xhtml

For peer review only

Trial Estimand (see also Statistical Analysis Plan)

Here we define the estimand for the primary analysis of the primary outcome in the trial, in line with the draft addendum to ICH E9 (Statistical Principles for Clinical Trials): ICH E9(R1), Defining the Appropriate Estimand for a Clinical Trial/Sensitivity Analyses.

Population Adult ICU patients enrolled within 48h of MV starting in ICU and expected to require sedation with propofol and MV for at least 48h, at least 24h of which would be after randomisation. Long-term home ventilation, terminal illness, selected diagnoses, allergy to study medication, pregnancy and expected death within 24h are exclusion criteria.

Variable Time to successful extubation post-randomisation (hours).

Population-level Summary Cumulative incidence function of time to extubation; sub-distribution hazard ratio (HR)

The following Intercurrent Events have been identified which would prevent measurement of the primary outcome or change the interpretation of the measured primary outcome:

1. Death before the time point at which randomised treatment is due to start.

2. (a) Dexmedetomidine allocated in randomisation but not started

(b) Clonidine allocated in randomisation but not started

3. Additional propofol being administered when cardiovascular side effects have limited the escalation of dexmedetomidine or clonidine.

4. Additional propofol being administered when non-cardiovascular side effects have limited the escalation of dexmedetomidine or clonidine.

5. Death before successful extubation.

6. Patient withdrawal from intervention and follow-up (situation where deferred consent is not granted is a subset of such events).

7. Transfer to another ICU before successful extubation.

8. Use of dexmedetomidine as main sedative in usual care group.

9. Use of clonidine as main sedative in usual care group.

10. Use of rescue medication in the presence of agitation or delirium.

Events 1, 2(a), 2(b), 8 and 9 are expected to be rare and no specific actions will be taken: analysis of these events will be by intention to treat, except for event 1 which will be handled in the same way as event 5.

For peer review only - http://bmjopen.bmj.com/site/about/guidelines.xhtml

Events 3 and 4 will be dealt with using an intention to treat approach. Non-cardiovascular side-effects will mostly be sedation-related and therefore will be further analysed as secondary outcomes.

Event 5 will be treated as a competing risk for the primary outcome, and will therefore be analysed using a hypothetical strategy.

Event 6 will also be handled using a hypothetical strategy, in which the time to extubation will be censored at the point of withdrawal and the withdrawals will be assumed to lead to missing at random (MAR) data on the primary outcome. Complete follow up should still be possible for most participants in whom event 7 occurs; if not, the hypothetical strategy used for event 6 will also be implemented.

Event 10 is analogous to events 8 and 9 but applies to all treatment arms and medications. An intention to treat approach will be used for this event.

Full details of the methods of dealing with the above intercurrent events will be incorporated in the statistical analysis plan.

For peer review only

For peer review only - http://bmjopen.bmj.com/site/about/guidelines.xhtml

Health Economic Evaluation

Overview

The significant cost differences between dexmedetomidine and both usual care and clonidine make the health economic evaluation especially relevant. Of importance, the cost of dexmedetomidine has decreased substantially during the conduct of the trial, such that the cost-effectiveness of the interventions may be different in the context of pricing in the 'post trial' era. Additional drug costs associated with α2-agonists should be balanced against potential cost savings from reductions in ICU and hospital stay and altered outcomes. We will undertake a detailed analysis of the cost and cost-effectiveness of dexmedetomidine, clonidine and usual care. Our analysis will conform to accepted economic evaluation methods in the UK. The comparisons made in the economic evaluation will reflect those of the staged hypothesis tests for the primary outcome (figure 1). We will estimate costs and cost-effectiveness for the 'within-trial' period (6 months/short-run model) and also over the expected lifetime of the patient ('lifetime'/long-run model). Costs will be assessed from the perspective of the NHS and personal social services (PSS). For both the within-trial and lifetime analyses we will undertake cost-utility analyses, estimating incremental cost per quality-adjusted life year (QALY) gained.

Within-trial analysis

For the within-trial analysis we will calculate detailed cost information on index hospitalisation for every patient from ICU admission to hospital discharge and during 6 months follow-up. Patient-level resource use data will be collected on items including: sedative drugs; costs associated with managing adverse events (e.g., delirium); length of ICU stay and hospital wards; use of MV; and co-prescribed medications. Patient-level resource use data will be collected post-discharge up to 6 months using questionnaires at 3 and 6 month follow-up: hospital re-admissions; A&E and outpatient visits; GP and nurse visits in clinic and at home; medications; care home admissions; any other contacts with primary and social care (e.g., physiotherapist, occupational therapist, social worker, counsellor). Unit costs will be obtained from published sources and inflated where appropriate before being applied to the volume of resource use data.

QALYs will be calculated based on the HRQoL and mortality data collected during the trial. HRQoL will be measured using the EQ-5D-5L (www.euroqol.org), which we will collect at 30 days, 3 and 6 months (see table 1). As patients recruited to the trial will be critically ill, completion of the EQ-5D-5L at baseline will not be possible. Previous studies have assumed a common baseline value for all patients (e.g., zero). We will use this approach and two alternatives. First, we will estimate baseline utility scores for patients using proxy responses from a person who knows the patient. In this case we will use the proxy version of the EQ-5D-5L (by the patient's spouse, parent or (adult) child). The type of proxy respondent will be controlled for in subsequent analyses. Second, we will ask patients to retrospectively record their baseline EQ-5D-5L at the 30 day follow-up point. We will evaluate QALYs associated with each strategy using all three approaches; the base case approach will use the proxy responses. Patients who die will be assigned a utility value of zero at the date of death and all subsequent time periods. Patient-specific utility profiles will be constructed assuming a

For peer review only - http://bmjopen.bmj.com/site/about/guidelines.xhtml

straight line relation between each of the patients' EQ-5D-5L scores at each follow-up point. The QALYs experienced by each patient from baseline to 6 months will be calculated as the area underneath this profile.

Multiple imputation by chained equations will be used to deal with missing HRQoL and resource use values. Subsequent analyses of imputed data will include variance correction factors to account for additional variability introduced into parameter values as a result of the imputation process. Cost-effectiveness will be calculated as the mean cost difference between groups divided by the mean difference in outcomes (QALYs) to give the incremental cost-effectiveness ratio (ICER). We will also calculate net monetary benefits (NMBs). We will subject the results to extensive deterministic (one-, two- and multi-way) and probabilistic sensitivity analysis. For the latter, non-parametric methods for calculating confidence intervals around the ICERs and NMBs based on bootstrapped estimates of the mean cost and QALY differences will be used. These methods will appropriately account for the multiple imputation of the missing data. The bootstrap replications will also be used to construct cost-effectiveness acceptability curves, which will show the probability that each strategy is cost-effective at 6 months for different values willingness to pay for additional QALYs by the NHS.

Lifetime analysis

In the lifetime model cost-effectiveness will be calculated in terms of the incremental cost per QALY gained. A review of the NIHR HTA website (www.hta.ac.uk/project/htapubs.asp) and the NHS Economic Evaluation Database (NHS-EED, www.crd.york.ac.uk https://www.crd.york.ac.uk/CRDWeb/) (last search 15/05/2017) reveals there have been no previous analyses to evaluate lifetime cost-effectiveness of the study strategies. Given this paucity of evidence, we will develop a de novo cost-effectiveness model that will be populated based on available evidence, including the data collected during the trial. We will: [1] design a lifetime model to characterize health states of ICU survivors; [2] populate the model using data identified from the trial and published literature and routine sources; [3] relate outcomes from the trial to final outcomes, expressed in terms of QALYs; and [4] identify which parameters in the model are most uncertain and are important drivers of cost-effectiveness. The model is likely to use a similar structure to a previous economic evaluation of long-term cost-effectiveness for ICU patients in the UK. Survival analysis of the RCT data will provide the basis for extrapolating any within-trial differences in costs and QALYs. The model will use external data on long-term survival of ICU survivors, including from co-applicants expert in this area (Lone, Walsh). Specific details of the data to be used to populate the model will be determined following the development of the structure and the systematic searches of the literature to identify existing models. We will undertake deterministic (one-, two- and multi-way) and probabilistic sensitivity analysis, the latter assuming appropriate distributions and parameter values. We will combine data on incremental costs with epidemiological data on projected patient numbers and undertake a budget impact analysis to evaluate what the total cost impact of each strategy would be were it to be scaled up; budget impact will be calculated separately for ICU-related costs only, the within-trial period and using a lifetime time horizon, as each might be appropriate

For peer review only - http://bmjopen.bmj.com/site/about/guidelines.xhtml

for different decision-makers. We will also use the probabilistic sensitivity analyses combined with the epidemiological information on projected patient numbers to undertake a value of information analysis to evaluate the potential economic value of future research on this topic.

For peer review only

For peer review only - http://bmjopen.bmj.com/site/about/guidelines.xhtml

Statistical Analysis Plan	A2B
Version No	2.0
Date Finalised	dd/mm/yyyy

An ICU Sedation Study

Alpha 2 agonists for sedation to produce better outcomes from critical illness (A2B Trial): A randomised, parallel-group, allocation concealed, controlled, open, phase 3 pragmatic clinical and cost- effectiveness trial with internal pilot

A2B Trial

Statistical Analysis Plan (Version as at 28th July 2023)

CONFIDENTIAL

**Version No**	2.0
**Date Finalised**	dd/mm/yyyy
**Author(s)**	Richard Parker (until unblinded on 2 August 2019)<br>Christopher Weir (from 2 August 2019)
**CI Name**	Professor Timothy Walsh
**CI Email address**	timothy.walsh@ed.ac.uk

**Funder**	NIHR Health Technology Assessment
**Funding Reference Number**	HTA 16/93/01
**Sponsor**	The University of Edinburgh & Lothian Health Board ACCORD
**EudraCT Number**	2018-001650-98
**ClinicalTrials.gov**	NCT03653832

Signatures	
**Trial Statistician: Prof Christopher Weir**	**Date:**
**Chief Investigator: Prof Timothy Walsh**	**Date:**

Document Control

**Version No**	**Date**	**Summary of Revisions**
1.0	22/01/2021	Initial Creation
2.0	dd/mm/yyyy	Incorporated modified sample size calculation. Updated to reflect latest ECTU SAP template (V4.0, 25Mar2021).

For peer review only - http://bmjopen.bmj.com/site/about/guidelines.xhtml

Statistical Analysis Plan	A2B
Version No	2.0
Date Finalised	dd/mm/yyyy

Table of Contents

List of Abbreviations 4
1. Introduction 5
2. Statistical Methods section from the protocol 5
8.2 PROPOSED ANALYSES 5
8.2.1 Estimand 5
8.2.2 Statistical analysis....6
3. Overall Statistical Principles 8
3.1 Analysis populations 8
3.2 Outcomes....8
4. List of Analyses....10
4.1 Recruitment, retention and missing data 10
4.2 Baseline characteristics 11
4.3 Primary outcome (primary analysis)....12
4.4 Primary outcome (supplementary analyses) 13
4.5 Subgroup analyses 14
4.6 Secondary outcomes 14
4.6.1 Missing data handling: secondary outcomes....15
4.7 Safety 16
4.8 Concomitant medications....16
4.9 Intervention dose, fidelity and reach....16
4.10 Protocol deviations and violations 17
5. Validation and QC 17
6. Data sharing 18
7. References....18
Appendix 1 Sedation Quality Assessment Tool (SQAT) 20
Appendix 2 PRE-DELIRIC score derivation....21
Appendix 3 Data completeness and intervention adherence 23

For peer review only - http://bmjopen.bmj.com/site/about/guidelines.xhtml

List of Abbreviations

Abbreviation	Full name
AE	Adverse event
CAM-ICU	Confusion-Assessment Method for ICU
CI	Confidence interval
CONSORT	CONsolidated Standards Of Reporting Trials
CPAP	Continuous positive airway pressure
CRF	Case report form
EQ-5D-5L	EuroQol instrument with five levels of severity in each of five dimensions
EudraCT	European Clinical Trials Database
HADS	Hospital Anxiety and Depression Scale
HR	Hazard ratio
HTA	Health Technology Assessment
ICE-Q	Intensive Care Experience Questionnaire
ICU	Intensive care unit
ICH	International Council for Harmonisation of Technical Requirements for Pharmaceuticals for Human Use
IES-R	Impact of Events Scale – Revised
MV	Mechanical ventilation
NIHR	National Institute for Health Research
NIV	Non-invasive mechanical ventilation
OR	Odds ratio
RASS	Richmond Agitation and Sedation Scale
SD	Standard deviation
SOFA	Sequential Organ Failure Assessment
SQAT	Sedation Quality Assessment Tool
T-MoCA	Montreal Cognitive Assessment tool (telephone version)

For peer review only - http://bmjopen.bmj.com/site/about/guidelines.xhtml

Statistical Analysis Plan	A2B
Version No	2.0
Date Finalised	dd/mm/yyyy

1. Introduction

A2B is a randomised, parallel-group, allocation concealed, controlled, open, multi-centre, phase 3 pragmatic clinical and cost- effectiveness trial with internal pilot. Adult intensive care unit (ICU) patients expected to require at least 24 hours further mechanical ventilation (MV) will be randomised within 48 hours of starting MV. Patients with primary brain injury; post-cardiac arrest; status epilepticus; and peripheral nervous system disease will be excluded. 1437 patients will be randomised to receive sedation using dexmedetomidine or clonidine or 'usual care' sedation in a 1:1:1 ratio. To simplify the enrolment process randomisation will be stratified by site alone.

This statistical analysis plan is written with reference to protocol version 7, dated 25 April 2023. Its scope covers the end of trial analysis for A2B, with the exception of the health economic evaluation, the process evaluation (apart from quantitative descriptions of fidelity to the intervention) and the mechanistic sub-study of pro- and anti-inflammatory mediators which will all be documented separately.

2. Statistical Methods section from the protocol

8.2 PROPOSED ANALYSES

8.2.1 Estimand

Here we define the estimand for the primary analysis of the primary outcome in the trial, in line with the draft addendum to ICH E9 (Statistical Principles for Clinical Trials): ICH E9(R1), Defining the Appropriate Estimand for a Clinical Trial/Sensitivity Analyses.

Population Adult ICU patients enrolled within 48h of MV starting in ICU and expected to require sedation with propofol and MV for at least 48h, at least 24h of which would be after randomisation. Long-term home ventilation, terminal illness, selected diagnoses, allergy to study medication, pregnancy and expected death within 24h are exclusion criteria.

Variable Time to successful extubation post-randomisation (hours).

Population-level Summary Cumulative incidence function of time to extubation; sub-distribution hazard ratio (HR)

The following ***Intercurrent Events*** have been identified which would prevent measurement of the primary outcome or change the interpretation of the measured primary outcome:

1. Death before the time point at which randomised treatment is due to start.
2. (a) Dexmedetomidine allocated in randomisation but not started
   (b) Clonidine allocated in randomisation but not started
3. Additional propofol being administered when cardiovascular side effects have limited the escalation of dexmedetomidine or clonidine.
4. Additional propofol being administered when non-cardiovascular side effects have limited the escalation of dexmedetomidine or clonidine.
5. Death before successful extubation.

For peer review only - http://bmjopen.bmj.com/site/about/guidelines.xhtml

6. Patient withdrawal from intervention and follow-up (situation where deferred consent is not granted is a subset of such events).
7. Transfer to another ICU before successful extubation.
8. Use of dexmedetomidine as main sedative in usual care group.
9. Use of clonidine as main sedative in usual care group.
10. Use of rescue medication [1] in the presence of agitation or delirium.

Events 1, 2(a), 2(b), 8 and 9 are expected to be rare and no specific actions will be taken: analysis of these events will be by intention to treat, except for event 1 which will be handled in the same way as event 5.

Events 3 and 4 will be dealt with using an intention to treat approach. Non-cardiovascular side-effects will mostly be sedation-related and therefore will be further analysed as secondary outcomes.

Event 5 will be treated as a competing risk for the primary outcome, and will therefore be analysed using a hypothetical strategy.

Event 6 will also be handled using a hypothetical strategy, in which the time to extubation will be censored at the point of withdrawal and the withdrawals will be assumed to lead to missing at random (MAR) data on the primary outcome. Complete follow up should still be possible for most participants in whom event 7 occurs; if not, the hypothetical strategy used for event 6 will also be implemented.

Event 10 is analogous to events 8 and 9 but applies to all treatment arms and medications. An intention to treat approach will be used for this event.

Full details of the methods of dealing with the above intercurrent events will be incorporated in the statistical analysis plan.

8.2.2 Statistical analysis

For the primary analysis, a Fine and Gray proportional sub-distribution hazards regression analysis of time from randomisation to successful extubation will be performed (this method allows us to directly model the cumulative incidence of extubation after taking into account the competing risk of mortality) for each hypothesis test permitted under the analytic structure (Figure 1). Results will be expressed as sub-distribution HRs with corresponding 95% confidence intervals and p-values.

The following supplementary analyses will be performed to provide reassurance about the robustness of the main analysis of the primary outcome, for each between-arm comparison:

(i) A Cox frailty proportional hazards regression model will be fitted to the primary outcome, with censoring for deaths or loss to follow-up in ICU while on MV. Although death in ICU may be considered a competing risk, this modelling approach allows us to estimate the instantaneous risk of experiencing a successful extubation event at time t given that the patient is still alive at time t (in the literature this is called the "cause-specific hazard" of extubation for patients who have not yet died). Site will be included in the model as a random effect.

[1] Rescue medication is recorded as haloperidol, quetiapine, dexmedetomidine, midazolam, olanzapine, clonidine, lorazepam or other

For peer review only - http://bmjopen.bmj.com/site/about/guidelines.xhtml

(ii) A Cox frailty regression analysis of time from randomisation to ICU mortality while on ventilation. Patients experiencing successful extubation events or loss to follow-up prior to mortality will be censored. This analysis will provide "cause-specific" HRs for patients on MV to support the primary analysis results. Site will be included in the model as a random effect.

(iii) A Cox frailty regression analysis of time to all-cause mortality, with censoring only for patients lost to follow-up during the six months follow-up period. This analysis will allow us to compare the risk of overall mortality across trial arms for all patients, regardless of whether or not patients are still on MV. Site will be included in the model as a random effect.

(iv) For each participant, the proportion of care periods will be recorded in which propofol treatment was maintained even though dexmedetomidine or clonidine had not been up-titrated to its maximum dose and had no dose-limiting side-effects. As an exploratory analysis, the main analysis of the primary outcome will be repeated using the adherence analysis set (section 8.2.3) rather than the full analysis set.

For the secondary outcomes other than mortality, formal hypothesis testing will not be performed but point estimates and 95% confidence intervals for pairwise differences between randomised groups will be calculated. A trial analysis plan providing full details will be finalised prior to locking the trial data base.

The hierarchical hypothesis testing framework for analysis of the primary outcome, which controls the overall type I error to be at most 6.5% across the multiple analyses being performed, is also outlined in protocol Figure 1:

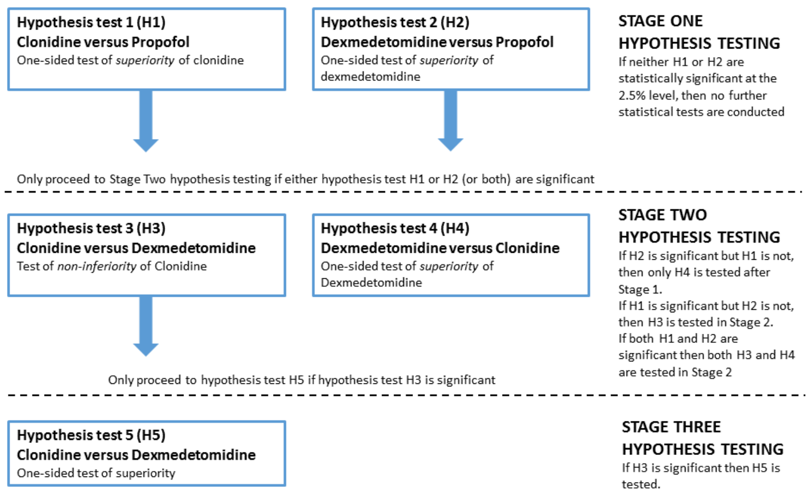

For peer review only - http://bmjopen.bmj.com/site/about/guidelines.xhtml

Statistical Analysis Plan	A2B
Version No	2.0
Date Finalised	dd/mm/yyyy

Figure 1: Analytic framework which efficiently tests the trial questions using a hierarchical analytic structure with serial gatekeeping to preserve study power. Hypothesis tests will be performed using a 2.5% one-sided significance level, with the exception of the non-inferiority test H3 which will use a 4% one-sided significance level.

3. Overall Statistical Principles

The Stage 1 hypothesis testing of the superiority of each of clonidine and dexmedetomidine versus propofol will be carried out at the one-sided 2.5% significance level. The Stage 2 hypothesis of non-inferiority of clonidine to dexmedetomidine will be performed with a one-sided 4% significance level. The Stage 2 hypothesis of superiority of dexmedetomidine to clonidine will have a one-sided 2.5% significance level. Finally, in Stage 3, there will be a possible test of superiority of clonidine versus dexmedetomidine at the one-sided 2.5% significance level. All hypothesis tests on the primary outcome are arranged in a hierarchical structure, with serial gatekeeping, to ensure overall control of the type 1 error to at most 6.5%.

Categorical variables will be summarised using frequencies and percentages; continuous variables will be summarised using the mean, standard deviation (SD), median, lower quartile, upper quartile, minimum and maximum values.

Analyses of outcomes will adjust for site as a random effect, since site is included as a stratification factor in the randomisation.

Generally speaking, missing data will be handled according to the principles outlined in the A2B estimand, described in protocol section 8.2.1. Participants randomised in error despite ineligibility, becoming ineligible before drug administered, or being withdrawn from the trial by family members prior to intervention, will be reported in the participant flow summary but will not be included in efficacy or safety analyses as no further data will be gathered on these participants.

Outliers will be identified by viewing boxplots of the outcome variables of interest. All analyses will include outliers as standard; where data are present which lie more than 4 standard deviations away from the mean, a sensitivity analysis will be performed removing these data values to determine the robustness of the findings in the analysis where outliers were included.

The planned analyses will be performed using the SAS statistical software, version 9.4 or later. Following the end of trial, defined as the date of the last follow-up of the final participant, the planned analyses will be performed once data querying has been completed and the locking of the trial database has been documented.

3.1 Analysis populations

Full analysis set

All participants randomised, analysed according to their allocated treatment group regardless of the treatment actually received.

Adherence analysis set

The **adherence analysis set** will be all randomised participants in the **full analysis set** who, in the dexmedetomidine group received any dexmedetomidine on the day of randomisation; in the clonidine

For peer review only - http://bmjopen.bmj.com/site/about/guidelines.xhtml

group received any clonidine on the day of randomisation; or in the usual care group received neither dexmedetomidine nor clonidine (except as rescue medication for agitation) on the day of randomisation.

3.2 Outcomes

Primary outcome

- Time to successful extubation post-randomisation (hours).

A successful first extubation from mechanical ventilation will be defined as follows:

a) From endotracheal extubation: time of first extubation that is followed by 48 hours of spontaneous breathing.

b) From tracheostomy: time of extubation will be defined as the start time of the first period during which a patient receives support not exceeding 5 cmH_2O CPAP with less or equal to pressure support ventilation of 5cmH_2O for a continuous period of 48 hours. Decannulation during this period will count as being free from mechanical ventilation.

c) From non-invasive mechanical ventilation (NIV): time of extubation will be the start time of the first period during which a patient receives support not exceeding 5 cmH_2O CPAP via mask/hood for a continuous period of 48 hours. NIV patients receiving any pressure supported breaths will not be considered to be spontaneously breathing unassisted.

NB: The use of high flow nasal oxygen will not be counted as mechanical ventilation, so a patient on high nasal flow oxygen alone will be considered to be spontaneously breathing unassisted.

Secondary outcomes

Secondary outcomes are listed in priority order. Specifically, mortality forms a component of the primary outcome time to successful extubation. Outcomes listed from Length of ICU Stay to Patient Experience of ICU Care are outcomes specified in the NIHR HTA briefing document for this commissioned funding call. The remaining outcomes are listed in order of priority according to guidance from patient and public involvement representatives.

S1 Mortality
ICU; hospital; 30 days; 90 days; 180 days post-randomisation

S2 Length of ICU stay (days from randomisation to ICU discharge)

S3 Sedation quality, measured by Richmond Agitation and Sedation Scale (RASS)

- Measured four-hourly during mechanical ventilation until primary outcome recorded, summarised as lowest and highest day shift and night shift RASS scores over time

S4 Sedation quality, measured during mechanical ventilation until primary outcome recorded by Sedation Quality Assessment Tool (SQAT- Appendix 1)
Four sedation quality states:

1. Overall optimum sedation (no agitation;no unnecessary deep sedation;no pain behaviour)
2. Agitation
3. Unnecessary deep sedation (RASS -4/-5 without clinical indication)
4. Pain (presence of pain behaviour based on limb movement and ventilation compliance)

S5 Time to first optimum sedation

For peer review only

- Hours from randomisation to first RASS score of -2 or greater
- Days from randomisation to first day with SQAT optimum sedation

S6 Delirium prior to successful extubation, assessed by Confusion-Assessment Method for ICU (CAM-ICU)
- Occurrence prior to successful extubation (binary outcome)
- Days with delirium or coma prior to successful extubation (continuous outcome)

S7 One or more pre-defined cardiac adverse events
(of those recorded daily: severe bradycardia; cardiac arrhythmias; cardiac arrest)

S8 Health-related Quality of Life, measured by recall prior to hospital admission, and at 30, 90 and 180 days after randomisation using the EuroQol EQ-5D-5L instrument

S9 Patient Ability to Communicate Pain and Ability to Cooperate with Care
Binary assessments for each 12 hours nursing shift:
- Was patient able to communicate pain?
- Was patient able to cooperate with care?

S10 Patient experience of ICU care, measured at 90 days after randomisation using the Intensive Care Experience Questionnaire (ICE-Q)
Provides numeric score in four domains:
1. Awareness of Surroundings (9 items; score range 9-45)
2. Frightening Experiences (6 items; score range 6-35)
3. Recall of Experiences (5 items; score range 5-25)
4. Satisfaction with Care (4 items; score range 4-20)

S11 Relative/partner/friend (PerLR) assessment of comfort and communication, measured daily during mechanical ventilation
Binary assessment for each question:
1. Does the patient appear awake to the visitor?
2. Does the patient seem comfortable to the visitor?
3. Does the visitor feel they can communicate with the patient?

S12 Anxiety and depression, measured at 180 days post randomisation using the Hospital Anxiety and Depression Scale (HADS) questionnaire

S13 Post-traumatic stress, measured at 180 days post randomisation using the Impact of Events Scale-revised (IES-R)

S14 Cognitive function, measured at 180 days post randomisation using the Montreal Cognitive Assessment tool telephone version (T-MoCA)

Commented [CW1]: Postal version no longer mentioned in protocol

4. List of Analyses

This analysis plan describes the end of trial statistical analyses to be performed on A2B, excluding analysis of the mechanistic sub-study of putative pro- and anti-inflammatory mediators (protocol

For peer review only - http://bmjopen.bmj.com/site/about/guidelines.xhtml

section 11), the health economics analyses and the process evaluation components of the trial. However, quantitative assessment of fidelity from the process evaluation is included in the scope of this analysis plan.

4.1 Recruitment, retention and missing data

A CONSORT flow diagram will be constructed. For EudraCT reporting purposes, enrolment will also be summarised into age categories 18-64; 65-84; and 85+ years.

The number and percentage of patients who were later found to be ineligible for the trial even though they were randomised will be summarised by randomised group, as will the number of patients formally withdrawn and the reason for withdrawal (if available). The number and percentage of patients with missing primary outcome data will be reported by randomised treatment allocation. No formal statistical testing will be performed.

4.2 Baseline characteristics

The following baseline characteristics will be summarised by treatment group and overall. A further descriptive summary will assess any association between the Covid-19 pandemic and participant characteristics. The baseline characteristics summary will be further stratified by randomisations occurring up to and including 23 March 2020 and those occurring after 23 March 2020.

Age (years)
Age (by EudraCT reporting categories)
Gender
Pre-randomisation:
- Estimated weight (kg)
- RASS
- CAM-ICU (unless RASS -4 or -5, or is -3 but the assessor is unable to assess CAM-ICU status)

Functional comorbidity index (Groll et al, 2005) (total count; and 18 separate items)
Medical history:
- Portal hypertension
- Biopsy proven cirrhosis
- Hepatic encephalopathy
- Alcohol dependence
- Drug dependence

Type of admission (Trauma, Non-trauma medical, Non-trauma surgical; Planned, Unplanned)
Diagnosis at admission (Medical)
Diagnosis at admission (Surgical)
Pre-randomisation sedatives (Propofol, Midazolam, Fentanyl, Alfentanil, Morphine, Remifentanil, Dexmedetomidine, Clonidine, Haloperidol, Diazepam, Other (free text)) For each report frequency and summarise dose, in units specified on CRF.
SOFA score (excluding neurological SOFA) (Singer et al, 2016)
Pre-randomisation blood results:
- Haemoglobin g/L
- Lymphocytes x10^9/L
- Sodium mmol/L
- Urea mmol/L
- Albumin g/L
- White cell count x10^9/L
- APTT ratio

For peer review only - http://bmjopen.bmj.com/site/about/guidelines.xhtml

Potassium mmol/L
eGFR mL/min/1.73m^2
ALT U/L

Blood gases:

H^+
pH
PaO_2 kPa
$PaCO_2$ kPa
Standard bicarbonate mmol/L
Lactate mmol/L

PRE-DELIRIC delirium prediction score (van den Boogaard et al, 2012; Appendix 2) including components:

Apache II score
Infection/sepsis
Antibiotics given during first 24 hours in ICU
Sepsis
Septic shock
Coma RASS -4/-5 for at least 8 hours in first 24 hours in ICU
If yes, by use of medication / other reason / both medication and other
Total morphine dose in first 24 hours in ICU
None / 0.01-7.1mg / 7.2-18.6mg / 18.7-331.6mg
Any propofol, midazolam or lorazepam use in first 24 hours in ICU
Highest urea value in first 24 hours in ICU (mmol/L)
Metabolic acidosis

Proxy baseline EQ-5D

4.3 Primary outcome (primary analysis)

For the primary analysis, performed on the full analysis set, a Fine and Gray proportional sub-distribution hazards regression analysis (Fine and Gray, 1999) of time from randomisation to successful extubation will be performed (this method allows us to directly model the cumulative incidence of extubation after taking into account the competing risk of mortality, thus implementing the hypothetical strategy outlined in the estimand for intercurrent events 1 and 5) for each hypothesis test permitted under the hierarchical testing structure. Results will be expressed as the sub-distribution hazard ratio (HR) for each of dexmedetomidine and clonidine versus usual care, with corresponding 95% confidence intervals (CI) and p-values from the Fine-Gray model. The exception will be the non-inferiority analysis of clonidine versus dexmedetomidine (hypothesis H3 in protocol figure 1) for which a 96% one-sided non-inferiority CI will be presented. Site will be accounted for in the analysis by implementing the marginal model approach to the Fine and Gray method for clustered data (Zhou et al, 2012). If this aspect of model fitting proves problematic due to sites which have randomised a small number of participants (fewer than 5), we will consider pooling of data from such sites to address this issue.

Intercurrent events 2(a), 2(b), 8 and 9 are expected to be rare and will therefore be handled using the intention to treat approach in the primary analysis of the primary outcome. Events 3 and 4 (propofol use due to cardiovascular and non-cardiovascular side-effects respectively) will also be handled using the intention to treat approach due the pragmatic exploration of the effects of clonidine and dexmedetomidine in A2B. Withdrawals where the participant has not withdrawn permission to use data collected up to the point of withdrawal will have time to extubation censored at the time of withdrawal (intercurrent event 6, missing at random assumption, hypothetical strategy). In the rare

For peer review only - http://bmjopen.bmj.com/site/about/guidelines.xhtml

cases of transfer to another ICU before extubation (intercurrent event 7), follow-up will be continued to extubation where possible but if extubation time is missing it will be censored at the last time at which the extubation status is known (missing at random assumption, hypothetical strategy). Intercurrent event 10 will be handled using intention to treat, again reflecting the treatment policy pragmatic nature of A2B.

The cumulative incidence function (CIF) obtained from the Fine-Gray model for time to successful extubation will be plotted separately for each treatment group; the median time to successful extubation and its 95% CI will be reported by treatment group. As recommended in the CONSORT reporting guidance, the absolute risk difference (and its 95% CI) for each of dexmedetomidine and clonidine versus control will be reported at 7 days after randomisation (the median time on mechanical ventilation under 'usual care' in a real ICU dataset).

Following the strategy recommended by Poythress et al. (2020), the fit of the Fine-Gray model will be evaluated by plotting, by treatment group, the CIF for time to successful extubation from the Fine-Gray model against the nonparametric CIF. If substantial differences occur between the Fine-Gray and nonparametric CIF curves an alternative modelling strategy, such as cause-specific hazards, will be considered.

4.4 Primary outcome (supplementary analyses)

Supplementary analyses will provide reassurance about the robustness of the primary analysis, for each between-arm comparison:

(i) A mixed effects partially proportional hazards regression model will be fitted to the primary outcome of time from randomisation to successful extubation, with censoring for deaths or loss to follow-up in ICU while on MV. Although death in ICU may be considered a competing risk, censoring for deaths allows us to estimate the instantaneous risk of experiencing a successful extubation event at time t given that the patient is still alive at time t (the "cause-specific hazard" of extubation for patients who have not yet died). Site will be included in the model as a random effect, treatment group as a fixed effect. Results will be expressed as the HR for each of dexmedetomidine and clonidine versus usual care, with its corresponding 95% CI and p-value.

(ii) A mixed effects partially proportional hazards regression analysis of time from randomisation to ICU mortality while on MV. Patients experiencing successful extubation events or loss to follow-up prior to mortality will be censored. For patients on MV, this analysis will provide the mortality "cause-specific" HR (and 95% CI) for each of dexmedetomidine and clonidine versus usual care, to support the primary analysis results. Site will be included in the model as a random effect, treatment group as a fixed effect.

(iii) Overall mortality will be analysed using a mixed effects partially proportional hazards regression analysis, see Section 4.6 for details.

(iv) The primary analysis will be repeated, but using the adherence analysis set.

Furthermore, selected baseline characteristics of patients with missing primary outcome data due to withdrawal will be compared descriptively to those with patients who did not withdraw prior to extubation to evaluate the missing at random assumption present in the primary analysis of intercurrent event 6.

Similarly, selected baseline characteristics of patients transferred to another ICU who did not have time to extubation recorded will be compared to those transferred to another ICU who did have it

For peer review only

recorded, to assess the missing at random assumption being made in the primary analysis of intercurrent event 7.

Finally, further exploratory analysis will assess any association between the Covid-19 pandemic and the primary outcome. Summary descriptive statistics of time to successful extubation will be reported by treatment group and further stratified by the date of the UK lockdown: randomisations occurring up to and including 23 March 2020 versus those occurring after 23 March 2020.

4.5 Subgroup analyses

The primary analysis of the primary outcome will be repeated for the following subgroups specified in the protocol.

(1) Patients with and without sepsis at enrolment to A2B.
(2) Patients with lower or higher delirium risk, as defined by the PRE-DELIRIC delirium risk prediction score. (van den Boogaard et al, 2012) The groups with values above (or including) and below the median PRE-DELIRIC score observed in the trial population will be compared.
(3) Patients with and without organ dysfunction at randomisation. The group with SOFA score values above or equal to the median SOFA score (excluding neurological score) that is present at baseline will be compared with the group with SOFA score values below the median score at baseline.
(4) Age (<64 versus ≥64)

For each subgroup variable, a p-value will be calculated for its interaction with each of dexmedetomidine and clonidine versus usual care. Within each subgroup category, we will calculate the sub-distribution HR and 95% confidence interval for (a) dexmedetomidine versus usual care and (b) clonidine versus usual care and present these in a forest plot. These analyses will be considered exploratory.

For age, an additional exploratory analysis will fit an interaction term based on its continuous value rather than age categories. A cubic B-spline, fractional polynomial or simple quadratic term will be fitted to determine, via a likelihood ratio test, whether there is a significant non-linear relationship between age and the effects of each of dexmedetomidine and clonidine versus usual care.

For the age subgroup, given the findings of the SPICE trial of dexmedetomidine (Shehabi et al., 2019), the above subgroup analysis will also be applied to the mortality secondary outcome **S1**.

4.6 Secondary outcomes

Each secondary outcome will be summarised appropriately, by treatment group and overall. Where informative graphical summaries will also be created. The large number of secondary outcomes means that not all will be included in the mean trial publication text. Instead, **S5**, **S9** and **S11** will be reported in the accompanying supplementary material. Other secondary outcomes for which there is substantial missing data will also be considered for transfer to the supplementary material.

For the secondary outcomes other than **S1**, mortality, formal hypothesis testing will not be performed but point estimates and 95% confidence intervals for pairwise differences between randomised groups will be calculated. P-values will not be reported.

For peer review only

For peer review only - http://bmjopen.bmj.com/site/about/guidelines.xhtml

Statistical Analysis Plan	A2B
Version No	2.0
Date Finalised	dd/mm/yyyy

For secondary outcomes measured at more than one time point following ICU discharge, separate analyses will be performed for each measurement occasion. Secondary outcomes **S9**, **S10** and **S11** will be summarised descriptively (for **S10,** for each of the four domains separately) without any calculation of confidence intervals for differences between groups.

S1 Mortality. A mixed effects partially proportional hazards regression analysis will be used to analyse time to all-cause mortality, with censoring only for patients lost to follow-up during the six months follow-up period. This analysis will allow us to compare the risk of overall mortality, using the HR, 95% CI and p-value, for each of dexmedetomidine and clonidine versus usual care for all patients, regardless of whether or not patients are still on MV. Site will be included in the model as a random effect and treatment group as a fixed effect.

The time to event secondary outcomes **S2** and **S9** will be analysed using the same method as for the primary analysis of the primary outcome (Section 4.3), in order to take account of the potential competing risk of death. The supplementary analyses of Section 4.4 will also be applied for these outcomes. Time to event outcome **S5** will be summarised descriptively but will not be formally analysed.

Binary secondary outcomes (**S6** [delirium occurrence], **S4**, **S7**, **S9**, **S11**) will be analysed by a generalised linear mixed model with a logit link function. Site will be included as a random effect in the model and treatment group as a fixed effect. For outcomes **S4** and **S9** which are measured in multiple care periods, a random effect for participant (nested within site) will also be included. Optimal sedation for outcome **S4** will be reported descriptively as a proportion for each combination of study day and treatment group. It will not be analysed formally. Each of the **S4** SQAT components (freedom from agitation; freedom from pain; and freedom from unnecessary deep sedation) will be reported descriptively as for optimal sedation and in addition will be analysed using the generalised linear mixed model with logit link. Results will be expressed as the odds ratio (OR) for each of dexmedetomidine and clonidine versus usual care, with corresponding 95% CI.

Continuous secondary outcomes (**S3** [highest RASS score recorded daily, regardless of whether clinical need for deep sedation was recorded], **S8**, **S12**, **S13**, **S14**) will be analysed using a normal linear mixed model. Site will be included as a random effect in the model and treatment group as a fixed effect. Outcome **S3** is measured in each care period so a random effect for participant (nested within site) will also be included. For **S3** each of the day shift and night shift highest and lowest RASS will also be summarised graphically up to the occurrence of the successful extubation primary outcome. A proxy for outcome **S8** is measured at baseline and this will be included as a fixed effect in the model. The parameter to be estimated is the adjusted mean difference: dexmedetomidine minus usual care; and clonidine minus usual care. The corresponding 95% CI will also be reported. If the assumption of normality of residuals does not hold (as determined by normal probability plot), the outcome variable will be transformed to rectify this. In the event that the assumption cannot be satisfied, alternative analyses (for example involving categorising the outcome measure) will be conducted. A similar strategy will be applied when residuals versus fitted values demonstrate non-constant variance for an outcome.

The count variable **S6**, delirium or coma days prior to successful extubation, will be analysed using a generalised linear mixed model with a log link (Poisson regression). Number of days prior to successful extubation will be included as an offset term in the model. Site will be included as a random effect in

For peer review only - http://bmjopen.bmj.com/site/about/guidelines.xhtml

the model. The result for each of dexmedetomidine and clonidine versus usual care will be presented as a rate ratio (RR) and 95% confidence interval.

4.6.1 Missing data handling: secondary outcomes

We anticipate minimal rates of missing data for the secondary outcome **S1**, mortality. In cases of missing data, the survival time will be censored at the date last known alive. Missing data on time to event secondary outcomes **S2** and **S9** will be handled using a similar approach to that used for **S1**.

In other secondary outcomes, for which no formal hypothesis testing will be undertaken, the following strategies will be implemented where missing data rates are low (less than 10% overall, and with a no more than 5% difference in the rate across treatment groups). For continuous secondary outcomes a "missing at random" assumption will be applied automatically within the normal linear mixed model, while complete case analyses will be performed for outcomes which are counts or binary variables. In the event of the missing data rate being greater than 10% overall, or differing by more than 5% across treatment groups, multiple imputation strategies will be considered.

4.7 Safety

Safety data will be reported for the full analysis population, according to treatment allocated.

While death will be analysed as a secondary outcome (Section 4.6), only deaths considered related to participation in A2B will be recorded as serious adverse events. Sedation-related adverse events (including hypotension, hypertension, unplanned NG removal, unplanned central line removal, unplanned arterial line removal, unplanned peripheral line removal, unplanned drain removal, unplanned extubation, staff injury as a result of patient, patient injury and ileus) will be reported descriptively: number and percentage by treatment group and overall.

During the recruiting ICU stay (or up to and including study day 28, whichever is earlier) the number and percentage of patients experiencing each of: any adverse event (AE); non-serious adverse event (NSAE); serious adverse event (SAE) and suspected unexpected serious adverse reaction (SUSAR) will be reported, overall and split by trial arm. Tabulations will be split by events occurring pre- and post-randomisation. The numbers of events will also be reported.

The AE, NSAE, SAE and SUSAR tables will also be further categorised by the number and percentage of patients recording an event in each of the MedDRA system organ class categories, with a further sub-categorisation according to verbatim text or MedDRA preferred term as appropriate.

Data listings of all adverse events will be provided by treatment group, according to MedDRA system organ class, verbatim text, severity, seriousness, causality, expectedness and outcome.

Daily data on blood results (platelets, bilirubin, creatinine), respiratory function (FiO_2, PaO_2, SpO_2), blood pressure (lowest systolic BP recorded and corresponding diastolic BP) and urine output (>500mL/day, 200-500mL/day,<200mL/day) will be summarised and presented graphically by ICU study day and treatment group. No formal statistical inference will be performed on these measures. When estimating the mean and SD measures below the limit of quantification (LLQ) will be handled by treating these observations as censored but positive, calculating the likelihood conditional on them being greater than zero. This is strategy M4 from Senn et al., 2012.

For peer review only - http://bmjopen.bmj.com/site/about/guidelines.xhtml

4.8 Concomitant medications

The frequency and percentage (of all those in the full analysis set) of patients in whom rescue medications are administered to decrease sedation when the RASS score is -4/-5 will be reported, overall and by treatment arm.

4.9 Intervention dose, fidelity and reach

Dose

The frequency of RASS assessments recorded per shift will be summarised overall, by treatment group and by study site.

Fidelity

The degree of implementation of various components of the A2B interventions will be summarised using the algorithm outlined in Appendix 3. Reporting will cover completeness of day and night shift forms; responses to deep sedation query; completeness of RASS data; completeness of CAM-ICU data on day and night shifts; completeness of pain behaviour data; deep sedation guidance compliance; number and proportion of care periods for each participant in which each of propofol, dexmedetomidine and clonidine was administered will be summarised overall and by treatment group; and propofol, dexmedetomidine and clonidine administration by study day for participants remaining on mechanical ventilation.

For each treatment group, the proportion of participants receiving propofol treatment on each study day will be reported.

Further evaluation of fidelity will be reported in the qualitative process evaluation.

Reach

The number and percentage of eligible patients recruited will be reported overall and by study site. More extensive analysis of reach will be reported in the qualitative process evaluation.

4.10 Protocol deviations and violations

For events which are specific to a participant, the number and percentage of each of protocol deviations and violations will be presented, split by site, trial arm and overall.

Deviations and violations which cannot be attributed to an individual participant (for example, an issue with a process in a site) will be presented in a line listing.

5. Validation and QC

The following will be performed by a second statistician:

1. Separate programming and checking of the primary and supplementary analyses for the primary outcome (Sections 4.3 and 4.4).

For peer review only - http://bmjopen.bmj.com/site/about/guidelines.xhtml

2. Separate programming and re-analysis of the mortality secondary outcome and all other secondary outcome analyses for which there is at least one statistically significant pairwise comparison (one-sided p-value <0.025) in the first statistician's analysis. If there are more than 10 such secondary outcomes, then 5 of them will be randomly selected for re-analysis.

3. The end of trial statistical report will be read and checked for accuracy and consistency.

6. Data sharing

A file, or set of files, containing an anonymised version of the final analysis data set will be prepared, along with a data dictionary. These will be made available to the Chief Investigator at the end of the analysis phase.

7. References

Andersen PK, Geskus RB, de Witte T, et al. Competing risks in epidemiology: possibilities and pitfalls. International Journal of Epidemiology 2012;41(3):861-70. doi: 10.1093/ije/dyr213

Fine J and Gray R. A proportional hazards model for the subdistribution of a competing risk. Journal of the American Statistical Association 1999;94(446): 496-509. doi:10.2307/2670170

Groll DL, To T, Bombardier C, Wright JG. The development of a comorbidity index with physical function as the outcome. Journal of Clinical Epidemiology 2005;58:595-602.

Noordzij M, Leffondre K, van Stralen KJ, et al. When do we need competing risks methods for survival analysis in nephrology? Nephrology, dialysis, transplantation : official publication of the European Dialysis and Transplant Association - European Renal Association 2013;28(11):2670-7. doi: 10.1093/ndt/gft355

Poythress JC, Yu Lee M, Young J. Planning and analyzing clinical trials with competing risks: Recommendations for choosing appropriate statistical methodology. Pharmaceutical Statistics. 2020;19:4–21. doi:10.1002/pst.1966

Senn S, Holford N, Hockey H. The ghosts of departed quantities: approaches to dealing with observations below the limit of quantitation. Statist. Med. 2012, 31 4280–4295.

Shehabi Y, Howe BD, Bellomo R, Arabi YM, Bailey M, Bass FE, Bin Kadiman S, McArthur CJ, Murray L, Reade MC, Seppelt IM, Takala J, Wise MP and Webb SA, for the ANZICS Clinical Trials Group and the SPICE III Investigators.* Early Sedation with Dexmedetomidine in Critically Ill Patients. N Engl J Med 2019; 380:2506-17.

Singer M, Deutschman CS, Warren Seymour C, Shankar-Hari M, Annane D, Bauer M, Bellomo R, -Bernard GR, Chiche J-D, Coopersmith CM, Hotchkiss RS, Levy MM, Marshall JC, Martin GS, Opal SM, Rubenfeld GD, van der Poll T, Vincent J-L, Angus DC. The Third International Consensus Definitions for Sepsis and Septic Shock (Sepsis-3). JAMA 2016;315(8):801-810. doi:10.1001/jama.2016.0287

For peer review only - http://bmjopen.bmj.com/site/about/guidelines.xhtml

Statistical Analysis Plan	A2B
Version No	2.0
Date Finalised	dd/mm/yyyy

van den Boogaard M, Pickkers P, Slooter AJ, et al. Development and validation of PRE-DELIRIC (PREdiction of DELIRium in ICu patients) delirium prediction model for intensive care patients: observational multicentre study. BMJ (Clinical research ed) 2012;344:e420. doi: 10.1136/bmj.e420

Varadhan R, Weiss CO, Segal JB, et al. Evaluating health outcomes in the presence of competing risks: a review of statistical methods and clinical applications. Medical Care 2010;48(6 Suppl):S96-105. doi: 10.1097/MLR.0b013e3181d99107

Zhou B, Fine J, Latouche A, Labopin M. (2012). Competing risks regression for clustered data. Biostatistics 2012;13(3):371-383.

For peer review only - http://bmjopen.bmj.com/site/about/guidelines.xhtml

Appendix 1 Sedation Quality Assessment Tool (SQAT)

For a given ICU shift, the sedation quality states of SQAT will be derived as:

Agitation Highest RASS +3/+4 (Daily Data Collection CRF)

Unnecessary deep sedation Lowest RASS -4/-5 AND Was the bedside nurse asked by medical staff to keep this patient deeply sedated? = "No" (Daily Data Collection CRF)

Pain Presence of pain behaviour based on:

Limb movement (Response to moving the participant = "Difficult to move most of the time" OR "Actively resisting movement most of the time") OR

((Compliance with the ventilator = "Tolerating ventilation but coughing/gagging frequently" OR "Unable to control ventilation due to poor patient synchronisation despite different modes tested") AND Was the participant paralysed throughout the entire nursing shift? = "No")

(Daily Data Collection CRF)

Overall optimum sedation is present when there is no agitation; no unnecessary deep sedation; and no pain behaviour.

For peer review only - http://bmjopen.bmj.com/site/about/guidelines.xhtml

Statistical Analysis Plan	A2B
Version No	2.0
Date Finalised	dd/mm/yyyy

Appendix 2 PRE-DELIRIC score derivation

The PRE-DELIRIC score will be derived according to the formula in van den Boogaard et al, 2012:

Formula for PRE-DELIRIC model

Risk of delirium = 1/(1+exp−(−6.31

+ 0.04 × age

+ 0.06 × APACHE-II score

+ 0 for non-coma or 0.55 for drug induced coma or 2.70 for miscellaneous coma or 2.84 for combination coma

+ 0 for surgical patients or 0.31 for medical patients or 1.13 for trauma patients or 1.38 for neurology/neurosurgical patients

+ 1.05 for infection

+ 0.29 for metabolic acidosis

+ 0 for no morphine use or 0.41 for 0.01-7.1 mg/24 h morphine use or 0.13 for 7.2-18.6 mg/24 h morphine use or 0.51 for >18.6 mg/24 h morphine use

+ 1.39 for use of sedatives

+ 0.03 × urea concentration (mmol/L)

+ 0.40 for urgent admission))

The scoring system's intercept is expressed as −6.31; the other numbers represent the shrunken regression coefficients (weight) of each risk factor.

Age: Randomisation date minus date of birth (Pre-Randomisation CRF)

APACHE II score: (Baseline CRF)

Coma:

Non-coma	Coma status = "No coma" (Baseline CRF)
Drug induced coma	Coma status = "Coma" AND "With use of medication" (Baseline CRF)
Miscellaneous coma	Coma status = "Coma" AND "Other" (Baseline CRF)
Combination coma	Coma status = "Coma" AND "Combination" (Baseline CRF)

Surgical/Medical/Trauma/Neurology/Neurosurgery:

Surgical	Type of ICU admission = "Non-trauma" AND ("Surgical" NOT (Diagnosis at Admission – Surgical Admission = "Intracerebral haemorrhage" OR "Subdural/epidural haematoma" OR "Subarachnoid haemorrhage" OR "Laminectomy / other spinal cord injury" OR "Craniotomy for neoplasm" OR "Other neurologic diseases"))
Medical	Type of ICU admission = "Non-trauma" AND ("Medical" NOT (Diagnosis at Admission – Medical Admission = "Intracerebral haemorrhage" OR "Subarachnoid haemorrhage" OR "Stroke" OR "Neurologic infection" OR "Neurologic neoplasm" OR "Neuromuscular disease" OR "Seizure" OR "Other neurologic disease"))
Trauma	Type of ICU admission = "Trauma (without traumatic brain injury)"
Neurology/Neurosurgery	Type of ICU admission = "Non-trauma" AND

For peer review only - http://bmjopen.bmj.com/site/about/guidelines.xhtml

Statistical Analysis Plan	A2B
Version No	2.0
Date Finalised	dd/mm/yyyy

((Diagnosis at Admission – Surgical Admission = “Intracerebral haemorrhage” OR “Subdural/epidural haematoma” OR “Subarachnoid haemorrhage” OR “Laminectomy / other spinal cord injury” OR “Craniotomy for neoplasm” OR “Other neurologic diseases”) OR
(Diagnosis at Admission – Medical Admission = “Intracerebral haemorrhage” OR “Subarachnoid haemorrhage” OR “Stroke” OR “Neurologic infection” OR “Neurologic neoplasm” OR “Neuromuscular disease” OR “Seizure” OR “Other neurologic disease”))
(Baseline CRF)

Infection:
Did the participant receive antibiotics for proven or suspected infection during their first 24 hours in ICU? = ”Yes” (Baseline CRF)

Metabolic acidosis:
pH < 7.35 (H+ > 44.7) with bicarbonate < 24 mmol/L in the first 24 hours in ICU? = “Yes” (Baseline CRF)

Morphine use:
Total administered morphine dose in first 24 hours in ICU =
“Morphine use: 0.01 – 7.1 mg” cumulative OR
“Morphine use: 7.2 – 18.6 mg cumulative” OR
“Morphine use: 18.7 – 331.6 mg cumulative”
(Baseline CRF)

Sedatives:
Any use of propofol, midazolam, lorazepam or combination in the first 24 hours in ICU? = “Yes” (Baseline CRF)

Urea concentration:
Please specify the highest serum urea value in the first 24 hours in ICU [mmol/L] (Baseline CRF)

Urgent admission: Planned Admission = “Unplanned” (Baseline CRF)

For peer review only - http://bmjopen.bmj.com/site/about/guidelines.xhtml

Appendix 3 Data completeness and intervention adherence

Rule 1: Removing non-intervention period days

Remove days on which answer to 'InvasivelyVentilated_YesNoDesc' and 'NonInvVentilation_YesNoDesc' is NO

This will remove the majority of days on which the patient was no longer ventilated during the intervention period. There will be a small number of days on which the response could be NO but the patient is subsequently re-intubated and the primary outcome has not been reached. However, subsequent ventilated days will be included as the answer to this question should revert to YES. For the purpose of tracking data quality this small discrepancy will not be important.

Remaining data should be all days on which patients was receiving mechanical ventilation as defined in the protocol

Rule 2: completeness of day and night shift forms

After rule 1:

Count proportion of 'DSBedsideNurse_YesNoDesc' that response is YES

Count proportion of 'NSBedsideNurse_YesNoDesc' that response is YES

Report this as proportion of 'shift forms' completed by clinical staff during day shift and night shift and overall by site and overall trial

Rule 3: responses to deep sedation query

After rule 1:

Count proportion of 'DSDeepSedation_YesNoNotCollectedDesc' reported for each category

Count proportion of 'NSDeepSedation_YesNoNotCollectedDesc' reported for each category

Report this for day shift and night shift and for overall by site and overall trial

Rule 4: completeness of sedation RASS data

After rule 1:

Report completeness of:

'DSHighestRASS_RASSScoreDesc'

'DSLowestRASS_RASSScoreDesc'

'NSHighestRASS_RASSScoreDesc'

'NSLowestRASS_RASSScoreDesc'

To provide a measure of ability to report a highest and lowest recorded RASS score on each day report:

Proportion of days on which:

'DSHighestRASS_RASSScoreDesc' OR 'NSHighestRASS_RASSScoreDesc' OR BOTH have a RASS score reported

'DSLowestRASS_RASSScoreDesc' OR 'NSLowestRASS_RASSScoreDesc' OR BOTH have a RASS score recorded

Rule 5: completeness of CAM-ICU data

After rule 1:

Report the following:

Day shift

Proportion of day shifts on which 'DSCAMICUPositive_CAMICUPositiveDesc' is YES OR NO (this is a definite response)

Proportion of day shifts on which 'DSCAMICUPositive_CAMICUPositiveDesc' response is 'RASS -3 but clinical team unable to assess CAM-ICU'

For peer review only - http://bmjopen.bmj.com/site/about/guidelines.xhtml

Proportion of day shifts on which 'DSCAMICUPositive_CAMICUPositiveDesc' response is 'Not collected' AND responses to ['DSHighestRASS_RASSScoreDesc' AND 'DSLowestRASS_RASSScoreDesc'] are both [-3 or -4 or -5]
Proportion of day shifts on which 'DSCAMICUPositive_CAMICUPositiveDesc' response is 'Not collected' AND responses to ['DSHighestRASS_RASSScoreDesc' AND 'DSLowestRASS_RASSScoreDesc'] are both [-2 or -1 or 0 or +1 or +2 or +3 or +4]
Night shift
Proportion of night shifts on which 'NSCAMICUPositive_CAMICUPositiveDesc' is YES OR NO (this is a definite response)
Proportion of night shifts on which 'NSCAMICUPositive_CAMICUPositiveDesc' response is 'RASS -3 but clinical team unable to assess CAM-ICU'
Proportion of night shifts on which 'NSCAMICUPositive_CAMICUPositiveDesc' response is 'Not collected' AND responses to ['NSHighestRASS_RASSScoreDesc' AND 'NSLowestRASS_RASSScoreDesc'] are both [-3 or -4 or -5]
Proportion of night shifts on which 'NSCAMICUPositive_CAMICUPositiveDesc' response is 'Not collected' AND responses to ['NSHighestRASS_RASSScoreDesc' AND 'NSLowestRASS_RASSScoreDesc'] are both [-2 or -1 or 0 or +1 or +2 or +3 or +4]

Rule 6: completeness of pain behaviour data
After rule 1:
Proportion of day shifts on which 'DSCompliance_VentilatorComplianceDesc' response is 'not collected by bedside nurse'
Proportion of day shifts on which 'DSCompliance_VentilatorComplianceDesc' response is NULL (ie no data)
Proportion of day shifts on which 'DSCompliance_VentilatorComplianceDesc' response is 'not collected by bedside nurse'
Proportion of day shifts on which 'DSCompliance_VentilatorComplianceDesc' response is NULL (ie no data)
Proportion of night shifts on which 'NSCompliance_VentilatorComplianceDesc' response is 'not collected by bedside nurse'
Proportion of night shifts on which 'NSCompliance_VentilatorComplianceDesc' response is NULL (ie no data)
Proportion of night shifts on which 'NSCompliance_VentilatorComplianceDesc' response is 'not collected by bedside nurse'
Proportion of night shifts on which 'NSCompliance_VentilatorComplianceDesc' response is NULL (ie no data)

Rule 7: indicative sedation guidance compliance
Shifts during which deep sedation was NOT requested
After rule 1:
Select shifts where response to 'DSDeepSedation_YesNoNotCollectedDesc' AND 'NSDeepSedation_YesNoNotCollectedDesc' is NO
For these shifts:
Proportion of each RASS score response to 'DSHighestRASS_RASSScoreDesc' AND 'NSHighestRASS_RASSScoreDesc'
These cumulative data should indicate how common it is for a patient in whom deep sedation was NOT requested for the patient NOT to achieve a highest recorded RASS of -2 or greater during the intervention period.

For peer review only - http://bmjopen.bmj.com/site/about/guidelines.xhtml

Rule 8: Correct administration of drugs according to group
After rule 1:
Patients allocated to usual care group
Number/proportion of days on which propofol administered 'Propofol_YesNoDesc'
Number/proportion of days on which dexmedetomidine administered 'Dexmedetomidine_YesNoDesc'
Number/proportion of days on which clonidine administered 'Clonidine_YesNoDesc'
Patients allocated to dexmedetomidine group
Number/proportion of days on which propofol administered 'Propofol_YesNoDesc'
Number/proportion of days on which dexmedetomidine administered 'Dexmedetomidine_YesNoDesc'
Number/proportion of days on which clonidine administered 'Clonidine_YesNoDesc'
Patients allocated to clonidine group
Number/proportion of days on which propofol administered 'Propofol_YesNoDesc'
Number/proportion of days on which dexmedetomidine administered 'Dexmedetomidine_YesNoDesc'
Number/proportion of days on which clonidine administered 'Clonidine_YesNoDesc'
This plot will give an overall indication of compliance without adjustment for the day of study.

Rule 9: correct administration according to group and day of study
Using Rule 8 data:
For each intervention group separately:
For study day 1, study day 2, study day 3 etc plot
Number/proportion of days on which propofol administered 'Propofol_YesNoDesc'
Number/proportion of days on which dexmedetomidine administered 'Dexmedetomidine_YesNoDesc'
Number/proportion of days on which clonidine administered 'Clonidine_YesNoDesc'
This plot will provide an indication of compliance according to the day of intervention (for patients remaining on mechanical ventilation.

For peer review only - http://bmjopen.bmj.com/site/about/guidelines.xhtml

STANDARD PROTOCOL ITEMS: RECOMMENDATIONS FOR INTERVENTIONAL TRIALS

SPIRIT 2013 Checklist: Recommended items to address in a clinical trial protocol and related documents*

Section/item	Item No	Description	Page
**Administrative information**			
Title	1	Descriptive title identifying the study design, population, interventions, and, if applicable, trial acronym	1
Trial registration	2a	Trial identifier and registry name. If not yet registered, name of intended registry	3
	2b	All items from the World Health Organization Trial Registration Data Set	?
Protocol version	3	Date and version identifier	7
Funding	4	Sources and types of financial, material, and other support	7, 19
Roles and responsibilities	5a	Names, affiliations, and roles of protocol contributors	1, 2
	5b	Name and contact information for the trial sponsor	18
	5c	Role of study sponsor and funders, if any, in study design; collection, management, analysis, and interpretation of data; writing of the report; and the decision to submit the report for publication, including whether they will have ultimate authority over any of these activities	18
	5d	Composition, roles, and responsibilities of the coordinating centre, steering committee, endpoint adjudication committee, data management team, and other individuals or groups overseeing the trial, if applicable (see Item 21a for data monitoring committee)	18
**Introduction**			

For peer review only

Background and rationale	6a	Description of research question and justification for undertaking the trial, including summary of relevant studies (published and unpublished) examining benefits and harms for each intervention	5-7
	6b	Explanation for choice of comparators	5-7, 12
Objectives	7	Specific objectives or hypotheses	7-8
Trial design	8	Description of trial design including type of trial (eg, parallel group, crossover, factorial, single group), allocation ratio, and framework (eg, superiority, equivalence, noninferiority, exploratory)	7, 14-15

Methods: Participants, interventions, and outcomes

Study setting	9	Description of study settings (eg, community clinic, academic hospital) and list of countries where data will be collected. Reference to where list of study sites can be obtained	10-11
Eligibility criteria	10	Inclusion and exclusion criteria for participants. If applicable, eligibility criteria for study centres and individuals who will perform the interventions (eg, surgeons, psychotherapists)	10-11
Intervention s	11a	Interventions for each group with sufficient detail to allow replication, including how and when they will be administered	11-13
	11b	Criteria for discontinuing or modifying allocated interventions for a given trial participant (eg, drug dose change in response to harms, participant request, or improving/worsening disease)	11-13
	11c	Strategies to improve adherence to intervention protocols, and any procedures for monitoring adherence (eg, drug tablet return, laboratory tests)	11-13, supplementary material
	11d	Relevant concomitant care and interventions that are permitted or prohibited during the trial	11-13
Outcomes	12	Primary, secondary, and other outcomes, including the specific measurement variable (eg, systolic blood pressure), analysis metric (eg, change from baseline, final value, time to event), method of aggregation (eg, median, proportion), and time point for each outcome. Explanation of the clinical relevance of chosen efficacy and harm outcomes is strongly recommended	8-10, table 1<br><br>Explanation/rationale 6-7

For peer review only - http://bmjopen.bmj.com/site/about/guidelines.xhtml

Participant timeline	13	Time schedule of enrolment, interventions (including any run-ins and washouts), assessments, and visits for participants. A schematic diagram is highly recommended (see Figure)	11-12, 14 Table 3
Sample size	14	Estimated number of participants needed to achieve study objectives and how it was determined, including clinical and statistical assumptions supporting any sample size calculations	15-17
Recruitment	15	Strategies for achieving adequate participant enrolment to reach target sample size	11, 17

Methods: Assignment of interventions (for controlled trials)

Allocation:

Sequence generation	16a	Method of generating the allocation sequence (eg, computer-generated random numbers), and list of any factors for stratification. To reduce predictability of a random sequence, details of any planned restriction (eg, blocking) should be provided in a separate document that is unavailable to those who enrol participants or assign interventions	11-12
Allocation concealment mechanism	16b	Mechanism of implementing the allocation sequence (eg, central telephone; sequentially numbered, opaque, sealed envelopes), describing any steps to conceal the sequence until interventions are assigned	11-12
Implementation	16c	Who will generate the allocation sequence, who will enrol participants, and who will assign participants to interventions	11-12
Blinding (masking)	17a	Who will be blinded after assignment to interventions (eg, trial participants, care providers, outcome assessors, data analysts), and how	N/A
	17b	If blinded, circumstances under which unblinding is permissible, and procedure for revealing a participant's allocated intervention during the trial	N/A

Methods: Data collection, management, and analysis

Data collection methods	18a	Plans for assessment and collection of outcome, baseline, and other trial data, including any related processes to promote data quality (eg, duplicate measurements, training of assessors) and a description of study instruments (eg, questionnaires, laboratory tests) along with their reliability and validity, if known. Reference to where data collection forms can be found, if not in the protocol	13, 14 table 3
	18b	Plans to promote participant retention and complete follow-up, including list of any outcome data to be collected for participants who discontinue or deviate from intervention protocols	13, table 3
Data management	19	Plans for data entry, coding, security, and storage, including any related processes to promote data quality (eg, double data entry; range checks for data values). Reference to where details of data management procedures can be found, if not in the protocol	13
Statistical methods	20a	Statistical methods for analysing primary and secondary outcomes. Reference to where other details of the statistical analysis plan can be found, if not in the protocol	14 (analytic framework) 14-17 statistical methods Statistical analysis plan (SAP)included as supplementary material
	20b	Methods for any additional analyses (eg, subgroup and adjusted analyses)	17
	20c	Definition of analysis population relating to protocol non-adherence (eg, as randomised analysis), and any statistical methods to handle missing data (eg, multiple imputation)	Estimand included in SAP

Methods: Monitoring

Data monitoring	21a	Composition of data monitoring committee (DMC); summary of its role and reporting structure; statement of whether it is independent from the sponsor and competing interests; and reference to where further details about its charter can be found, if not in the protocol. Alternatively, an explanation of why a DMC is not needed	18

	21b	Description of any interim analyses and stopping guidelines, including who will have access to these interim results and make the final decision to terminate the trial	Not Applicable
Harms	22	Plans for collecting, assessing, reporting, and managing solicited and spontaneously reported adverse events and other unintended effects of trial interventions or trial conduct	Pre-defined AEs collected in protocol table 3 AE/SAE reporting 18
Auditing	23	Frequency and procedures for auditing trial conduct, if any, and whether the process will be independent from investigators and the sponsor	Monitoring plan 18
**Ethics and dissemination**			
Research ethics approval	24	Plans for seeking research ethics committee/institutional review board (REC/IRB) approval	18
Protocol amendment s	25	Plans for communicating important protocol modifications (eg, changes to eligibility criteria, outcomes, analyses) to relevant parties (eg, investigators, REC/IRBs, trial participants, trial registries, journals, regulators)	18
Consent or assent	26a	Who will obtain informed consent or assent from potential trial participants or authorised surrogates, and how (see Item 32)	10
	26b	Additional consent provisions for collection and use of participant data and biological specimens in ancillary studies, if applicable	10
Confidentiali ty	27	How personal information about potential and enrolled participants will be collected, shared, and maintained in order to protect confidentiality before, during, and after the trial	18
Declaration of interests	28	Financial and other competing interests for principal investigators for the overall trial and each study site	19
Access to data	29	Statement of who will have access to the final trial dataset, and disclosure of contractual agreements that limit such access for investigators	19
Ancillary and post-trial care	30	Provisions, if any, for ancillary and post-trial care, and for compensation to those who suffer harm from trial participation	18

For peer review only

Dissemination policy	31a	Plans for investigators and sponsor to communicate trial results to participants, healthcare professionals, the public, and other relevant groups (eg, via publication, reporting in results databases, or other data sharing arrangements), including any publication restrictions	18
	31b	Authorship eligibility guidelines and any intended use of professional writers	Not applicable
	31c	Plans, if any, for granting public access to the full protocol, participant-level dataset, and statistical code	19
**Appendices**			
Informed consent materials	32	Model consent form and other related documentation given to participants and authorised surrogates	Supplementary material
Biological specimens	33	Plans for collection, laboratory evaluation, and storage of biological specimens for genetic or molecular analysis in the current trial and for future use in ancillary studies, if applicable	Not Applicable

*It is strongly recommended that this checklist be read in conjunction with the SPIRIT 2013 Explanation & Elaboration for important clarification on the items. Amendments to the protocol should be tracked and dated. The SPIRIT checklist is copyrighted by the SPIRIT Group under the Creative Commons "Attribution-NonCommercial-NoDerivs 3.0 Unported" license.

For peer review only

BMJ Open

Alpha 2 agonists for sedation to produce better outcomes from critical illness (A2B Trial): Protocol for a multicentre phase 3 pragmatic clinical and cost-effectiveness randomised trial in the United Kingdom

Journal:	*BMJ Open*
Manuscript ID	bmjopen-2023-078645.R1
Article Type:	Protocol
Date Submitted by the Author:	22-Oct-2023
Complete List of Authors:	Walsh, Timothy; The University of Edinburgh Usher Institute of Population Health Sciences and Informatics<br>Aitken, Leanne M; City University; City University of London<br>McKenzie, Cathrine; University of Southampton<br>Boyd, Julia; The University of Edinburgh Usher Institute of Population Health Sciences and Informatics, Edinburgh Clinical Trials Unit<br>Macdonald, Alix; The University of Edinburgh Usher Institute of Population Health Sciences and Informatics<br>Giddings, Annabel; The University of Edinburgh Usher Institute of Population Health Sciences and Informatics<br>Hope, David; NHS Lothian<br>Norrie, John; Edinburgh Clinical Trials Unit, University of Edinburgh No. 9, Bioquarter, Usher Institute<br>Weir, Christopher; University of Edinburgh, Edinburgh Clinical Trials Unit, Usher Institute<br>Parker, Richard; University of Edinburgh, Edinburgh Clinical Trials Unit<br>Lone, Nazir; The University of Edinburgh Usher Institute of Population Health Sciences and Informatics<br>Emerson, Lydia; City University of London<br>Kydonaki, Kalliopi ; Edinburgh Napier University<br>Creagh-Brown, Benedict; Royal Surrey County Hospital NHS Foundation Trust; Royal Surrey County Hospital, Intensive Care Unit<br>Morris, Stephen; University of Cambridge, Primary Care Unit<br>McAuley, Daniel; Queen's University Belfast, Centre for Experimental Medicine<br>Dark, Paul; University of Manchester, Intensive Care Unit<br>Wise, Matt ; University Hospital of Wales, Dept. of adult critical care<br>Gordon, Anthony; Imperial College London, 1. Section of Anaesthetics, Pain Medicine and Intensive Care<br>Perkins, Gavin; University of Warwick, Clinical Trials Unit<br>Reade, Michael; University of Queensland<br>Blackwood, Bronagh; Queen's University Belfast, Wellcome-Wolfson Institute for Experimental Medicine<br>MacLullich, Alasdair; University of Edinburgh, Geriatric Medicine Unit<br>Glen, Robert; NHS Lothian<br>Page, Valerie; West Hertfordshire Hospitals NHS Trust, Intensive Care; Imperial College London Faculty of Medicine,

**Primary Subject Heading**:	Intensive care
Secondary Subject Heading:	Health economics, Research methods
Keywords:	Clinical Trial, Adult intensive & critical care < INTENSIVE & CRITICAL CARE, Clinical trials < THERAPEUTICS

SCHOLARONE™ Manuscripts

Alpha 2 agonists for sedation to produce better outcomes from critical illness (A2B Trial): Protocol for a multicentre phase 3 pragmatic clinical and cost-effectiveness randomised trial in the United Kingdom

Timothy S Walsh, Dept of Anaesthesia, Critical Care and Pain Medicine, Usher Institute, University of Edinburgh, Edinburgh, EH16 4SA, UK

Leanne M Aitken, School of Health & Psychological Sciences, City, University of London, London EC1V 0HB, UK

Cathrine A McKenzie, University of Southampton, School of Medicine, National Institute of Health, and Social Care Research (NIHR), Biomedical Research Centre, Perioperative and Critical Care Theme, SO17 1BJ, UK

Julia Boyd, Edinburgh Clinical Trials Unit, Usher Institute, University of Edinburgh, Edinburgh, EH16 4UX, UK

Alix Macdonald, Edinburgh Clinical Trials Unit, Usher Institute, University of Edinburgh, Edinburgh, EH16 4UX, UK

Annabel Giddings, Edinburgh Clinical Trials Unit, Usher Institute, University of Edinburgh, Edinburgh, EH16 4UX, UK

David Hope, Edinburgh Critical Care Research Group, NHS Lothian, Edinburgh, EH16 4SA, UK

John Norrie, Edinburgh Clinical Trials Unit, Usher Institute, University of Edinburgh, Edinburgh, EH16 4UX, UK

Christopher J Weir, Edinburgh Clinical Trials Unit, Usher Institute, University of Edinburgh, Edinburgh, EH16 4UX, UK

Richard A Parker, Edinburgh Clinical Trials Unit, Usher Institute, University of Edinburgh, Edinburgh, EH16 4UX, UK

Nazir Lone, Dept of Anaesthesia, Critical Care and Pain Medicine, Usher Institute, University of Edinburgh, Edinburgh, EH16 4SA, UK

Lydia Emerson, School of Health & Psychological Sciences, City, University of London, London EC1V 0HB, UK

Kalliopi Kydonaki, School of Health and Social Care, Edinburgh Napier University, 9 Sighthill Court, EH11 4BN, Edinburgh, UK. National and Kapodistrian University of Athens, Nursing department, 123 Papadiamadopoulou st. Athens, Greece.

Ben Creagh-Brown, Intensive Care Unit, Royal Surrey NHS Foundation Trust, Guildford, GU2 7XX, UK; Faculty of Health and Medical Sciences, University of Surrey, Guildford, UK.

For peer review only - http://bmjopen.bmj.com/site/about/guidelines.xhtml

Stephen Morris, Dept Public Health and Primary Care, University of Cambridge, Cambridge CB1 8RN, UK

Daniel F McAuley, School of Medicine, Dentistry and Biomedical Sciences, Queens University Belfast, Belfast, UK

Paul Dark, Critical Care Medicine, Division of Immunology, Immunity to infection and Respiratory Medicine, University of Manchester, Manchester M15 6JA, UK

Matt P Wise, Adult Critical Care, University Hospital of Wales, Cardiff, CF14 4XW, UK

Anthony C Gordon, Division of Anaesthetics, Pain Medicine and Intensive Care, Imperial College London, London W2 1NY, UK

Gavin D Perkins, Warwick Medical School, University of Warwick, Coventry, CV4 7AL, UK

Michael C. Reade, Faculty of Medicine, University of Queensland. Herston, Brisbane, 4029, Australia.

Bronagh Blackwood, School of Medicine, Dentistry and Biomedical Sciences, Queens University Belfast, Belfast, UK

Alasdair MacLullich, Edinburgh Delirium Research Group, Ageing and Health, Usher Institute, University of Edinburgh, Edinburgh, EH16 4SA, UK

Robert Glen, Lay Representative

Valerie Page, Dept of Anaesthetics, West Herts Teaching Hospitals NHS Trust, Watford, WD18 0HB, UK

Corresponding author:

Professor Tim Walsh

Department of Anaesthesia, Critical Care & Pain Medicine

Centre for Population Health Sciences, Usher Institute

Room S8208, 2nd Floor

The Royal Infirmary of Edinburgh

51 Little France Crescent

Edinburgh BioQuarter

Edinburgh EH16 4SA

Phone: 0131 242 6395

e-mail: twalsh@staffmail.ed.ac.uk

For peer review only

Key Words

Critical Illness; sedation; clinical trial; alpha2-agonists; mechanical ventilation

Word Count: 4598

Figures: 1

Tables: 3

This manuscript has an electronic supplement

For peer review only

For peer review only - http://bmjopen.bmj.com/site/about/guidelines.xhtml

Abstract

Introduction

Almost all patients receiving mechanical ventilation (MV) in intensive care units (ICUs) require analgesia and sedation. The most widely used sedative drug is propofol, but there is uncertainty whether alpha2-agonists are superior. The A2B trial aims to determine whether clonidine or dexmedetomidine (or both) are clinically and cost-effective in MV ICU patients compared to usual care.

Methods and analysis

Adult ICU patients within 48 hours of starting MV, expected to require at least 24 hours further MV, are randomised in an open-label three arm trial to receive propofol (usual care) or clonidine or dexmedetomidine as primary sedative, plus analgesia according to local practice. Exclusions include patients with primary brain injury; post-cardiac arrest; other neurological conditions; or bradycardia. Unless clinically contra-indicated, sedation is titrated using weight-based dosing guidance to achieve a Richmond-Agitation-Sedation score of -2 or greater as early as considered safe by clinicians. The primary outcome is time to successful extubation. Secondary ICU outcomes include delirium and coma incidence/duration, sedation quality, predefined adverse events, mortality, and ICU length of stay. Post-ICU outcomes include mortality, anxiety and depression, post-traumatic stress, cognitive function, and health-related quality of life at 6-month follow-up. A process evaluation and health economic evaluation are embedded in the trial.

The analytic framework uses a hierarchical approach to maximise efficiency and control type I error. Stage 1 tests whether each alpha2-agonist is superior to propofol. If either/both interventions are superior, stage 2 and 3 testing explores which alpha2-agonist is more effective. To detect a mean difference of 2 days in MV duration, we aim to recruit 1437 patients (479 per group) in 40-50 UK ICUs.

Ethics and dissemination

The Scotland A REC approved the trial (18/SS/0085). We use a surrogate decision-maker or deferred consent model consistent with UK law. Dissemination will be via publications, presentations, and updated guidelines.

Trial registration

ClinicalTrials.gov NCT03653832

299 words

For peer review only - http://bmjopen.bmj.com/site/about/guidelines.xhtml

Trial Summary

'Strengths and limitations of this study'

- This is the largest randomised trial simultaneously comparing both clonidine and dexmedetomidine to propofol (usual care) in a pragmatic effectiveness design.
- The trial maximises efficiency by using a hierarchical approach to hypothesis testing that primarily establishes whether each alpha2-agonist is superior to propofol, but retains power to explore their relative effectiveness if this is demonstrated.
- The trial includes a process evaluation that will provide information to help understand the results.
- The trial includes a detailed health economic evaluation, which is relevant because ICU care is costly and there are differences in costs between the drugs which are changing over time.
- The trial only has moderate power to detect potentially important differences in mortality, and heterogeneity of effects according to patient age and other factors.

For peer review only

For peer review only - http://bmjopen.bmj.com/site/about/guidelines.xhtml

Introduction

Around 20 million patients worldwide require intubation and mechanical ventilation (MV) in intensive care units (ICUs) each year.(1) Almost all require sedation and analgesia to relieve pain and anxiety, achieve comfort, and facilitate treatment. Guidelines recommend that patients are kept awake or lightly sedated whenever possible, and as early during ICU care as possible.(2-4) Sedative choice may influence the prevalence and duration of delirium, which is associated with adverse outcomes. However, it remains uncertain whether this relationship is causal, in part because delirium prevention and management strategies have been ineffective in most studies.

Research has shown an association between deep sedation and adverse short-term outcomes including prolonged MV and ICU stay, hospital acquired infections, and greater mortality, although this evidence has been inconsistent.(2, 5, 6) A concern regarding keeping patients more awake has been whether long-term psychological morbidity, such as post-traumatic stress, anxiety, and depression might be increased.(7-9) It is uncertain whether 'light sedation' strategies or the choice of sedative agent can modify this, either directly or by decreasing delirium.(8, 10, 11)

The most established drugs for patient sedation are the gamma-aminobutyric acid receptor (GABA) agonists, namely propofol or benzodiazepines. These are prescribed once adequate analgesia, usually with opioid drugs, has been established. Benzodiazepines are associated with greater delirium, and propofol is recommended for first line use in guidelines and is the first-line sedative in the UK. Alpha2 agonists are an alternative class of sedative that provide sedation by dose-dependent decrease in noradrenergic neuron activity in the brain stem via pre- and post-synaptic receptor-mediated effects.(12) Unlike GABAergic sedatives, alpha2 agonists have analgesic properties, which can reduce opioid requirements.(13) Two alpha2-agonists are in widespread use in ICUs in the United Kingdom:

Dexmedetomidine is a highly selective alpha2-agonist with a α2:α1 receptor selectivity ratio of 1620:1.(14) It was developed as a sedative agent and is licensed for intravenous ICU sedation. The drug is >90% protein bound. Unbound drug crosses the blood–brain barrier to exert central effects. Metabolism in the liver creates inactive metabolites which are excreted renally. Renal impairment does not significantly alter clinical effects. The terminal elimination half-life is around 2 hours.

Clonidine was the prototype alpha2-agonist, licensed for hypertension, but subsequently used therapeutically for a wide range of neuropsychiatric conditions, drug withdrawal syndromes, and in pain medicine.(15) The drug is available in multiple formulations (including oral, transdermal, and intravenous). Many clinical uses are unlicensed, including ICU sedation via any route. Clonidine has significantly lower α2-receptor selectivity than dexmedetomidine; α2:α1 selectivity is 220:1 (x8 less than dexmedetomidine). Clonidine is less protein bound than dexmedetomidine (20-40%), and around 65% is excreted unchanged in the urine. The elimination half-life is significantly longer and variable (typically 5-13 hours), and (unlike dexmedetomidine) is prolonged by renal failure (18-41 hours). Peak effects after a single dose occur after 10-60 minutes, but may last 3-7 hours.

For peer review only - http://bmjopen.bmj.com/site/about/guidelines.xhtml

A survey of UK ICUs when planning this trial found 58% of ICUs use dexmedetomidine, but in less than 10% of patients. More than 90% used clonidine, in up to 25% of patients, but administration route and protocols varied widely. Widespread practice variation was present. Although widely used in the UK, intravenous clonidine has limited international use and is not included in international guidelines(16). Dexmedetomidine is licensed for ICU sedation and has been manufactured 'off patent' since 2019. Clonidine not licensed for ICU use, but is administered via both oral/enteral and intravenous routes, especially for the management of agitation and delirium.

Current evidence

The safety and effectiveness of clonidine for ICU sedation has not been studied in large randomised trials. A systematic review (SR) of studies in critical care included eight studies (643 patients).(17) There was important and relevant heterogeneity in multiple areas, including the population; routes of administration (6 intravenous and 2 oral); and dosage regimens. In 7 of 8 trials clonidine was used for adjunctive rather than stand-alone sedation. Meta-analysis suggested no effect on clinical outcomes but an association with hypotension (RR 3.11; 95% CI = 1.64 to 5.87).

Dexmedetomidine has been widely studied, and evidence summarised in a range of systematic reviews (SR) and meta-analyses. These have varied in terms of population definition (including SRs of all critically ill MV adults, or restricted to older patients or those with sepsis) and also the comparator (including 'usual care sedation' or propofol). The primary outcomes include mortality, duration of mechanical ventilation, and delirium. SRs prior to 2020 did not include data from the largest trial of dexmedetomidine (see below). The most recent SRs compared dexmedetomidine versus other sedative agents(18) or propofol(19) in critically ill MV adults in published trials to 2022. Dexmedetomidine was found to reduce delirium (moderate certainty), the duration of MV (low certainty), and ICU length of stay (low certainty)(18). There was no effect on mortality at 30 days (moderate certainty). Dexmedetomidine increased the risk of bradycardia and hypotension. Authors commented on population heterogeneity, with different risk profiles for key clinical outcomes.

The SPICE III trial randomised 4000 patients to receive dexmedetomidine or usual care within 12 hours of ICU admission.(20) The primary outcome of mortality was no different between the groups. Patients in the dexmedetomidine group had more ventilator free days (VFDs) and more days free of coma or delirium during 28 days follow-up. The median duration of ventilation in the trial was 3-4 days, and overall dexmedetomidine patients gained one VFD and had one less day of coma/delirium during 28 days follow-up. There were 6 pre-defined sub-group analyses. There were no differences in mortality according to baseline illness severity, severity of oxygenation impairment, geographic region, admission type (operative/non-operative), or sepsis at enrolment. There was a difference in mortality for patients above and below the median patient age. Patients aged <63.7 years who received dexmedetomidine experienced more deaths (mean absolute risk difference 4.4% (95% CI 0.8% -7.9%)), and patients aged ≥63.7 years experienced fewer deaths (mean absolute risk difference -4.4% (95% CI -8.7% - -0.1%)). This finding was explored in a detailed *post hoc* analysis which confirmed the finding using a range of statistical approaches, but without an explanation for the effect.(21) A cluster analysis suggested that a beneficial effect on

For peer review only - http://bmjopen.bmj.com/site/about/guidelines.xhtml

mortality may be most marked in operative versus non-operative patients. Based on these data a caution around increased mortality risk in patients aged ≤65 years was issued in June 2022 by the European Medicine Agency (EMA)(22).

Pharmaco-economic considerations

There is a cost-difference between the three agents used in the A2B trial, but the cost of dexmedetomidine has decreased substantially since coming off-licence. Current estimates (August 2023) for a typical daily UK cost for sedating a 70kg adult receiving MV in the UK are: propofol £15 (€17); dexmedetomidine £22 (€25) and clonidine £8 (€9). Changes in cost, combined with potential effects on clinically important outcomes mean a health economic evaluation of alpha2-agonists is relevant.

Research Commission and funding

The A2B trial was funded as a UK National Institute of Health and Care Research (NIHR) Health Technology Assessment (HTA) Agency commissioned trial (16/93 'alpha-2 agonists for sedation in critical care', 2017). The project brief specifically highlighted the widespread off-licence use of clonidine in the absence of safety and effectiveness evidence. The funder and grant reference number is: 16/93/01.

Trial Registration

The trial is registered on ClinicalTrials.gov (NCT03653832); EudraCT number is 2018-001650-98. This paper is based on protocol version 7.0 (date: 25/4/2023)

Methods and analysis:

The primary hypothesis is that sedation with alpha2-agonists will decrease the time to extubation in adult MV ICU patients compared with propofol (usual care).

Design

Randomised, parallel-group, allocation concealed, controlled, open-label, phase 3, pragmatic, clinical and cost-effectiveness trial with an internal pilot. After intubating and stabilising patients, we randomise patients (1: 1: 1) as early as possible to receive sedation-analgesia based on clonidine *or* dexmedetomidine *or* to continue propofol (usual care) plus opioid analgesia as required.

Patients and Public Involvement (PPI)

Former ICU patients and their relatives were consulted during the application to the NIHR Health Technology Assessment panel in addressing the importance of the research questions, and the design of the study, through participation in focus groups. A former ICU patient (RG) is a co-applicant on the grant and co-investigator on the trial. The PPI group were consulted when agreeing the primary and secondary outcomes, and played a key role in agreeing the long term outcome measures, the frequency of assessment, and the tools used to collect

For peer review only - http://bmjopen.bmj.com/site/about/guidelines.xhtml

them. RG is providing advice throughout the trial. In addition, the Trial Steering Group includes an independent lay member.

Primary Objective

To determine whether intravenous sedation with the alpha2-agonist agents, dexmedetomidine or clonidine, can decrease the time to successful extubation from MV among adult critically ill patients.

Secondary Objectives

Clinical and Person-centred objectives

During ICU stay we compare rates and duration of delirium or coma, time to optimum sedation, average sedation depth, the ability of patients to communicate with staff and relatives, the quality of sedation, and duration of ICU stay. We also compare safety based on pre-defined adverse events relevant to sedation and alpha2-agonist agents.

Following discharge from the ICU we compare patient outcomes for which sedation and ICU experience may be on the causal pathway, namely patients' memories of their ICU stay, psychological wellbeing, and cognitive function. We will follow up patients for 6 months for survival, health-related quality of life (HRQoL), and healthcare resource use.

Economic evaluation

We will include a detailed cost-effectiveness analysis from an NHS and personal social services perspective.

Process evaluation

The trial, by necessity, is a complex healthcare intervention trial evaluating different classes of sedative agents that involves multiple healthcare professionals, assessing and delivering multiple agents using a series of interrelated activities guided by bedside flowcharts, across multiple sites. Recognising this, and consistent with the MRC complex intervention framework(23), we include a process evaluation to explore the processes involved in intervention delivery, and identify factors and the mechanisms of their interaction likely impacting on trial outcomes.

Outcomes and Endpoints

Primary endpoint:

Time to successful extubation post-randomisation (hours). This is defined as:

a. For patients with an endotracheal tube: the time of the first extubation that is followed by 48 hours of spontaneous breathing without mechanical support

For peer review only - http://bmjopen.bmj.com/site/about/guidelines.xhtml

b. For patients with a tracheostomy: the start time of the patient's first period of 48 hours of spontaneous breathing, where spontaneous breathing is defined as receiving support not exceeding 5 cmH_2O Positive End Expiratory Pressure (PEEP) or Continuous Positive Airway Pressure (CPAP) with ≤ 5 cmH_2O pressure support above PEEP

c. For patients who are receiving non-invasive mechanical ventilation (NIV): the start time of the patient's first period of 48 hours of spontaneous breathing, defined as receiving support not exceeding 5 cmH_2O CPAP via mask/hood

Secondary outcomes

The A2B trial has a range of clinical and patient centred outcomes, which were discussed and approved following a Public and Patient Involvement exercise. These are shown in table 1.

Table 1: secondary outcomes, measurement tool or method, and timing.

**Outcome**	**Measurement tool or method**	**Timing**
**Mortality**	Medical records check	ICU, hospital, 30, 90 and 180 days post randomisation
**Length of ICU stay**<br>Number of days the participant is in ICU	Medical record	ICU discharge
**Sedation and analgesia quality**<br>Lowest and highest RASS score per day over time during intervention<br><br>Quality of sedation using SQAT states (daily basis); days with optimum sedation, agitation, or unnecessary deep sedation (RASS -4/-5).<br><br>Quality of analgesia using presence of pain behaviour (daily basis) based on limb response to movement and ventilation compliance	Richmond Agitation and Sedation Scale (RASS)<br><br>Sedation Quality (based on Sedation Quality Assessment Tool (SQAT).(24) Two components of the SQAT pain assessment will be used in this trial to measure sedation quality (limb relaxation and compliance with ventilation)<br>Defines four states for sedation quality:<br>1. Overall optimum sedation (no agitation; no unnecessary deep sedation; no pain behaviour)<br>2. Agitation<br>3. Unnecessary deep sedation (RASS -4/-5 without clinical indication)<br>4. Pain (presence of pain behaviour based on limb response to movement and ventilation compliance)	Four hourly during ICU stay until primary outcome is reached<br><br>Derived from daily sedation and analgesia quality data during intervention period in ICU until primary outcome is reached
**Time to first Optimum sedation** Hours<br>Hours from randomisation to first 'light' sedation (RASS score of -2 or greater)	RASS scores 4 hourly during ICU stay<br><br>SQAT status (daily during ICU stay)	Based on daily sedation and pain assessments during the intervention period

For peer review only - http://bmjopen.bmj.com/site/about/guidelines.xhtml

Days from randomisation to first day with optimum sedation (based on SQAT definition)		
**Delirium prior to successful extubation**<br>Occurrence prior to successful extubation (binary outcome)<br><br>Days with delirium (CAM-ICU positive) or coma (RASS score -4/-5) prior to successful extubation (continuous outcome)	Confusion Assessment Method for the ICU (CAM-ICU)(25)	Twice daily during ICU stay until primary outcome is reached
**Drug-related adverse events**<br>Number of patients experiencing a predefined adverse event and each defined adverse event<br><br>Number of days prior to successful extubation that any predefined adverse event occurred, and each defined adverse event occurred.	Severe bradycardia; cardiac arrhythmias; cardiac arrest (defined in protocol)	Daily during the intervention period
**Health-related Quality of Life**<br>HRQoL at 30, 90, and 180 days post randomisation	EuroQol tool (EQ-5D-5L)	Recalled HRQoL prior to hospital admission; prospective measurement 30, 90 and 180 days post randomisation
**Patients' Ability to Communicate Pain and Ability to Cooperate with Care**<br><br>Number of days on which pain could be communicated during intervention (binary score)<br><br>Number of days on which patient was able to cooperate with care (binary score)	Binary assessment for each 12 hours nursing shift requested from bedside nurse (based on overall assessment of period of care). Answer to the following questions:<br>1. Was your patient able to communicate pain?<br>2. Was your patient able to cooperate with care?	Twice daily until primary outcome is reached
**Patient experience of ICU care**<br>ICE-Q score at 90 days post-randomisation overall for each domain	Intensive Care Experience Questionnaire (ICE-Q)(26)<br>Provides numeric score in four domains:<br>1. Awareness of Surroundings<br>2. Frightening Experiences<br>3. Recall of Experiences<br>4. Satisfaction with Care	90 days post randomisation
**Relative/partner/friend *__(PerLR)__* assessment of comfort and communication**<br><br>Daily response to each of the three questions (binary outcome)	Relative/partner/friends response to the following questions (based on their opinion at time of assessment):<br>1. Does the patient appear awake to the visitor?<br>2. Does the patient seem comfortable to the visitor?<br>3. Does the visitor feel they can communicate with the patient?	Daily at a visit until primary outcome is reached

For peer review only - http://bmjopen.bmj.com/site/about/guidelines.xhtml

**Anxiety and depression**<br>HADS score at 180 days post-randomisation	Hospital Anxiety and Depression Scale (HADS) questionnaire	180 days post randomisation
**Post-traumatic stress**<br>Impact of Events Scale-revised (IES-R) score at 180 days post-randomisation	Impact of Events Scale-revised (IES-R)	180 days post randomisation
**Cognitive function**<br>TMoCA score at 180 days post-randomisation	Montreal Cognitive Assessment Tool (Telephone version) (TMoCA)	180 days post randomisation

Study population

The target population are critically ill patients requiring MV, recruited as early during ICU stay as possible, with an anticipated total requirement for MV of *at least* two days. Alpha2-agonists are not appropriate as single agents for intubation and early sedation for most acutely ill patients. Anaesthesia to undertake endotracheal intubation and establish initial ICU sedation-analgesia follows current usual care.

Inclusion and exclusion criteria

Inclusion and exclusion criteria are listed in table 2.

Table 2: inclusion and exclusion criteria for the A2B trial.

**Inclusion criteria**
1. Patient requiring MV in an ICU<br>2. Aged 18 or over<br>3. Within 48 hours of first episode of mechanical ventilation in ICU<br>4. Requiring sedation with propofol<br>5. Expected to require *a total* of 48 hours of MV or more in ICU<br>6. Expected to require a further 24 hours of MV or more *at the time of randomisation* in the opinion of the responsible clinician<br>Note: Criteria 5 and 6 are intended to ensure that all participants require at least 48 hours of MV in the ICU and that all patients receive at least 24 hours of the allocated intervention after randomisation.
**Exclusions**
1. Acute brain injury (traumatic brain injury; intracranial haemorrhage; ischaemic brain injury from stroke or hypoperfusion)[1]<br>2. Post-cardiac arrest (where there is clinical concern about hypoxic brain injury)[1]<br>3. Status epilepticus[1]<br>4. Continuous therapeutic neuromuscular paralysis at the time of screening or randomisation[1]<br>5. Guillain-Barre Syndrome[1]<br>6. Myasthenia gravis[1]<br>7. Home ventilation[1, 4]<br>8. Fulminant hepatic failure[2]<br>9. Patient not expected by responsible clinician to survive 24 hours

For peer review only

10. Decision to provide only palliative or end-of-life care
11. Pregnancy
12. Known allergy to one of the study drugs
13. Patient known to have experienced a period with heart rate <50 beats per minute for 60 minutes or longer since commencing mechanical ventilation in the ICU
14. Untreated second or third degree heart block[3]
15. Transferred from another Intensive Care Unit in which MV occurred for >6 hours
16. Prisoners
17. Enrolled on another Clinical Trial of an Investigational Medicinal Product
18. Previously enrolled on the A2B Trial

Note:
[1]For these conditions the neuromuscular condition will dominate the primary outcome unrelated to sedation practice
[2]Uncertain pharmacokinetics of α-2 agonist; potential for cerebral oedema mandating deep sedation
[3]Patients with treated heart block, for example with a pacemaker, are eligible for inclusion
[4]Home ventilation does not include patients receiving night-time CPAP and/or BIPAP therapy for the treatment of obstructive sleep apnoea syndrome.

For peer review only

Screening and consent

Participants are identified by clinical and research teams. Potential participants lack mental capacity. Appropriate approaches to consent according to UK law are used, approaching Personal and Professional legal representatives. The use of the 'emergency provision' can be used for deferred consent when a legal representative is not available within 2 hours of meeting eligibility criteria. In all cases, when patients regain capacity, they are approached for consent to continue in the trial (see supplementary file 1).

Randomisation

Randomisation is undertaken immediately after consent is obtained or when deferred consent is triggered by the research team, using a remote web-based randomisation system. Randomisation is in a 1:1:1 ratio to the three interventions using permuted blocks (randomly arranged sizes of 3, 6, 9, 12) stratified by centre. The allocation sequence was generated by a clinical trials unit programmer not involved in clinical management and is stored on a remote secure server concealed from all personnel involved in the trial.

Intervention Groups

Patients commence intravenous infusion of open-label study drug according to a weight-based dose regimen (see supplementary file 1) as early as possible post-randomisation, and within a maximum of two hours.

Bedside clinical staff transition patients to achieve sedation with the allocated alpha2-agonist agent as quickly as clinically feasible and safe, using bedside guidance algorithms (see supplementary file 1). Additional opioid is used for analgesia using clinical judgement. Once alpha2-agonist is established, additional propofol is only recommended when the maximum

For peer review only - http://bmjopen.bmj.com/site/about/guidelines.xhtml

alpha2-agonist dose is reached or because cardiovascular or other side-effects limit dose escalation.

Dexmedetomidine group

For dexmedetomidine, starting dose is 0.7micrograms/kg/hour titrated to a maximum dose 1.4micrograms/kg/hour as per manufacturer guidance. Lower starting doses are used at clinical discretion for patients with cardiovascular instability e.g. for patients on high doses of norepinephrine. No loading dose is administered.

Clonidine group

For clonidine, the regimen is designed to be equipotent with dexmedetomidine based on known pharmacokinetics and pharmacodynamics. The chosen regimen is similar to that currently used in many UK ICUs as part of routine 'off label' practice. The starting dose is 1.0micrograms/kg/hour titrated to a maximum dose of 2micrograms/kg/hour. Lower starting doses can be used at clinical discretion for patients with cardiovascular instability as for dexmedetomidine. No loading dose is administered.

Usual care group

Patients continue to receive intravenous propofol according to current usual care. The sedation targets, weaning, and sedation discontinuation procedures follow the same clinical targets as for the intervention groups.

The dosing guidance algorithms are included in the supplementary material.

Duration of intervention

The intervention period continues until: [1] The patient is successfully extubated according to the definition of the primary outcome; or [2] the patient dies during MV in the ICU; or [3] the patient is transferred to another non-participating ICU prior to achieving the primary outcome, or [4] 28 days of MV in ICU have been required following randomisation without achieving the primary outcome.

Timing of discontinuation of sedative agents is at the discretion of the clinical team. If the patient is re-intubated before achieving the primary outcome, they continue with group allocated treatment until the primary outcome is successfully achieved.

Management during the intervention period

The default sedation target is the most awake and comfortable state considered safe by clinical staff. For each 12 hours nursing shift, clinical staff document whether there is a clinical indication for deep sedation, such as brain injury, seizures or a requirement for advanced mechanical ventilation modes. If deep sedation is required, the allocated sedative agent is titrated to achieve this if feasible. In the absence of clinical requirement for deep sedation, the *least awake* target sedation state will be 'brief eye contact made in response to voice' (RASS score of -2). The additional use of daily sedation breaks is at the discretion of the caring clinical teams.

For peer review only - http://bmjopen.bmj.com/site/about/guidelines.xhtml

Staff in participating ICUs receive training in the trial protocol prior to recruiting patients. RASS score is recorded every 4 hours. The bedside algorithms recommend changes to sedation drug (according to group allocation) based on responses to RASS scores (see supplementary file 1). Patients receive opioid infusions for analgesia as clinically indicated. Patients who require additional sedation or treatment, for example for agitation, receive this according to local practice.

Patients receiving norepinephrine or other vasopressors at enrolment can be commenced on lower doses of alpha2-agonist. This is suggested when the dose of norepinephrine is more than 0.15 micrograms/kg/min. Patients who develop hypotension and/or bradycardia in any treatment group are managed according to local practices using fluids and/or vasopressors. Sedative drugs can be reduced or stopped based on clinical discretion. In the alpha2-agonist groups, if the patient's heart rate decreases to less than 50/minute, the alpha2-agonist is stopped until the heart rate increases to greater than 50/minute. Re-starting the allocated sedative regimen is encouraged once cardiovascular instability has improved.

Weaning from mechanical ventilation

All patients have regular assessments and attempts to wean and discontinue MV throughout treatment. The approach used in individual ICUs and patients should adhere to 'best practice' principles for weaning from MV. The protocol does not control decisions about weaning sedation and mechanical ventilation tightly, given the pragmatic effectiveness design. Decisions and their timing are at the discretion of the responsible clinical team.

Data Collection

Data collection throughout the study is shown in table 3. Study data are recorded into a case report form (CRF), and transcribed into the web-based electronic CRF within the Edinburgh Clinical Trials Unit (ECTU). Automated query identification and checking is managed and resolved by the trial management team. A trial monitoring strategy by the sponsor tracks data quality at sites and triggers any corrective actions.

Withdrawals

Participants or their relatives can withdraw at any time. The three options for ongoing data collection will be: withdraw from intervention only, but follow-up and all data collection continues; intervention and follow-up only, with collection of routine data allowed; or withdrawal from all aspects of the trial and follow-up. Wherever possible primary outcome data are recorded for any withdrawn patient.

For peer review only - http://bmjopen.bmj.com/site/about/guidelines.xhtml

Table 3: assessments and measurements undertaken during the trial

	Pre-Randomisation	Baseline Data	Daily ICU Data Collection [1]	ICU Discharge [1]	Hospital Discharge [1]	30 days[2]	90 days[2]	180 days[2]
Screening for eligibility and consent, demographics, CHI/hospital number, RASS, CAM-ICU, final eligibility check	X							
Baseline data collection - baseline data, FCI, APACHE II, SOFA, RASS, CAM-ICU, PRE-DELIRIC (collected at 24 hours), EQ-5D-5L (assessed by proxy).		X						
Sepsis substudy only - 2 blood samples for inflammatory markers<br>• Baseline sample (within 12 hours post randomisation)<br>• 60 hour sample (within 48-72 hours post randomisation)		X						
Daily data collection during ICU stay until primary outcome confirmed or day 28 – clinical team (4hrly - RASS score and pain assessment; 12hrly – CAM-ICU, SQAT, co-operation and communication assessment)			X					
Daily data collection during ICU stay until primary outcome confirmed or day 28 – research team (MV data collection, IMP and drug usage, SOFA score, adverse event data collection)			X					
Assessment of comfort and communication by informant until primary outcome confirmed or day 28			X					
Adverse Event data collection until ICU discharge			X					
ICU and hospital discharge data				X	X			
Mortality			X	X	X	X	X	X
Intensive Care Experience Questionnaire (ICE-Q)							X	
Hospital Anxiety and Depression Scale (HADS) questionnaire								X
Impact of Events Scale – revised (IES-R)								X
Montreal Cognitive Assessment Tool (Telephone version - TMoCA)								X
Euroqol tool (EQ-5D-5L)						X	X	X
Recalled Euroqol tool (EQ-5D-5L)						X		
Health economic questionnaire (including hospital resource use and return to employment)							X	X

[1]These data are collected from the routine health record, except for the EG-5D-5L which is collected from the patient's proxy

[2-]These data are collected by research staff. Site teams confirm patient status, and then the research team contacts the patient using a mixed strategy including postal and telephone contact to maximise completion

Design and Analysis Plan

Analytic framework

The hierarchical analytic framework was devised to address key clinical effectiveness questions in a staged manner, to enable an efficient trial design that controls overall "family-wise" Type 1 error rate. The trial will determine whether alpha2-agonists are superior to current practice but also, if superiority is found, *which* agent is more clinically effective. We propose three analytic stages, where progression to hypothesis testing in sequential stages is dependent on preceding results (see figure 1). A detailed justification and explanation of these stages is included in the statistical analysis plan (see supplementary file 2).

For peer review only

For peer review only - http://bmjopen.bmj.com/site/about/guidelines.xhtml

Further details regarding the original rationale for the study design and formation of the sample size calculations have been presented elsewhere(27).

Power and sample size during trial design

Based on clinical consensus, likely economic benefit, and the findings of systematic reviews, a minimum clinically important difference (MCID) of a mean difference in MV of 2 days was chosen for all superiority tests. For non-inferiority of clonidine versus dexmedetomidine, a non-inferiority margin of 1 day was chosen.

Sample size and power were modelled based on the analytic framework outlined in figure 1, which includes a hierarchical approach to hypothesis testing to control the “familywise” type I error to 5%. We used a large prospective data set from a sedation trial in 8 UK ICUs for modelling (N=708).(28) Based on this data set, we estimate that 53% of patients in the ‘usual care’ group will be extubated and around 14% will have died prior to extubation at 7 days.

Stage one: If either dexmedetomidine or clonidine are superior to usual care by an overall mean difference of 2 days in time to extubation, this translates to an estimated extubation rate of 63% in the dexmedetomidine or clonidine arm at 7 days. The death rate of 14% was assumed to remain the same as for the usual care arm. Under these conditions, using nQuery version 8 software (log-rank test accounting for competing risks), a sample size of 550 per arm (1650 patients in total, 1328 extubation events across the three arms) has 99% power to detect hazard ratios of 1.37 indicating superiority of clonidine or dexmedetomidine over usual care, assuming a one-sided 2.5% significance level.

Stage two: These analyses are only undertaken if one or other or both of the Stage one tests are significant. For the non-inferiority test of clonidine relative to dexmedetomidine (test H3), the non-inferiority margin is a 1-day absolute mean difference in time to extubation. Based on the modelled dataset, a 1-day absolute mean difference translates into an estimated probability of 63% in the dexmedetomidine arm and 57% in the clonidine arm achieving the primary outcome at 7 days. This equates to an estimated non-inferiority margin on the hazard ratio scale of 0.83, assuming death rates in both arms are 14% at 7 days. Using this information in nQuery version 8 software (log-rank test accounting for competing risks), 550 patients per arm (1100 in total, 888 extubation events) provides 81% power to conclude non-inferiority of clonidine, using a one-sided 2.5% significance level. The power calculation for the superiority comparison of dexmedetomidine versus clonidine (test H4) is the same as that for Stage one. Simulation work was used to calculate the overall power of test H1 (clonidine superiority test versus propofol) *and* test H3 (clonidine non-inferiority test versus dexmedetomidine) being statistically significant using Fine and Gray proportional sub-distribution hazards regression analysis based on 2000 trials simulated from the real ICU dataset (mean 7 days on ventilation).(28) Assuming that dexmedetomidine and clonidine are both superior to usual care by an overall true mean difference of 2 days, and there is no difference between dexmedetomidine and clonidine, then a total sample size of 1650 (550 per group) provides 81% power of concluding non-inferiority of clonidine over

For peer review only - http://bmjopen.bmj.com/site/about/guidelines.xhtml

dexmedetomidine (test H3) *and* concluding clonidine is superior to usual care (test H1) based on simulation, using a one-sided 2.5% significance level.

Stage three: The power calculation for the superiority comparison of clonidine versus dexmedetomidine (test H5), which will only be done if Stage one demonstrates superiority (tests H1 or H2) *and* clonidine is non-inferior to dexmedetomidine (test H3), is the same as that given in Stage 1.

Original sample size

We inflated sample size by 5% for loss to follow up for the primary outcome. The original trial sample size was therefore 1737 (579 patients per group).

Mortality

For the key outcome of mortality in ICU prior to extubation, a sample size of 550 per group provides 83% power to detect a difference in mortality of 7% (equivalent to a HR of approximately 1.5) using Cox regression assuming mortality in the usual care group is 23% and 16% in the clonidine/dexmedetomidine group, using a 2-sided 5% significance level.

Modifications to Sample Size due to impact of COVID19 pandemic

The COVID19 pandemic had a major impact on the trial progress and recruitment. In consultation with the funder, a modification to the original sample size was agreed in February 2023. The focus was on maintaining high power for the Stage one hypothesis testing, and included modelling the impact of a reduced sample size on the stage two test of non-inferiority of clonidine versus dexmedetomidine, plus the power for detecting an effect on mortality. Based on these investigations the sample size was reduced to 1437. This maintained 99% power for the Stage 1 comparisons of clonidine and dexmedetomidine versus propofol (H1 and H2), and also for the superiority comparison of dexmedetomidine versus clonidine if progression to Stage 2 testing occurs (H4). The main effect on power was for the non-inferiority comparison of clonidine versus dexmedetomidine (H3). For this comparison, in order to maintain 80% power when using the non-inferiority margin of 1 day, the significance level for test H3 was increased from 2.5% to 4%. This change to the hypothesis testing hierarchy meant that the upper limit on the familywise type I error rate increased from 5% to 6.5%. For the key secondary outcome of mortality, for the same 7% mortality difference, power decreased from 83% to 76%.

Pre-defined sub-group analyses

We plan four exploratory sub-group analyses, for patients with: [1] sepsis at enrolment; [2] higher delirium risk as defined by the PRE-DELIRIC delirium risk prediction score, using the version assessed at 24 hours post-admission(29); [3] greater organ dysfunction, as measured by SOFA score, at randomisation (as this could differentially alter the safety profile of the three groups); and [4] age ≥64 years versus age <64 years (based on the relationship between age and mortality seen in the SPICE III trial)(20, 21)

For peer review only - http://bmjopen.bmj.com/site/about/guidelines.xhtml

Statistical Analysis Plan (SAP)

An estimand was developed to deal how key intercurrent events will be dealt with in the analysis (see supplementary files 1). A detailed SAP has been finalised. The current version is included as an electronic supplement (see supplementary file 2). The most up-to-date version can be found in the statistics section of the Trial Master File held in the ECTU.

Process Evaluation (PE)

A PE is included recognising that ICU sedation is a complex healthcare intervention that involves multiple healthcare professionals, assessing and delivering multiple agents using a series of interrelated activities, across multiple sites. The PE aims to: establish the extent to which the intervention is delivered as intended (fidelity, dose, and reach), over time and across different ICUs; ascertain how clinical staff understand and respond to the intervention, over time and across different ICUs; and, explore the importance of context (inter-ICU differences, changes over time) and determine factors (including organisational structure and processes) that affect intervention implementation and delivery. The detailed PE methods and analytic framework will be published separately.

Health economic evaluation

We will undertake a detailed analysis of the cost-effectiveness of dexmedetomidine, clonidine and usual care. We will estimate costs and cost-effectiveness for both the 'within-trial' period and over the expected lifetime of the patient. Costs will be assessed from the perspective of the NHS and personal social services (PSS). QALYs will be calculated based on the HRQoL and mortality data collected during the trial. Details of the health economic evaluation is included in the supplementary material.

Monitoring, Pharmacovigilance and Safety monitoring

Participants are monitored for adverse events (AEs) and serious adverse events (SAEs) until ICU discharge. Recording and reporting of AEs and SAEs will follow the Standard Operating Procedures of the trial sponsor (ACCORD). A trial monitoring plan designed by the study sponsor is in place, which includes study audits at study sites and within the trial management team and is carried out by independent sponsor QA personnel. All protocol amendments and their dissemination are managed according to sponsor SOPs compliant with UK Health Research Authority (HRA) guidance.

Ethics and dissemination

The trial is classified as a Clinical Trial of an Investigational Medicinal Product (CTIMP). The trial was reviewed and approved by the Scotland A REC (18/SS/0085), which for a CTIMP provides approval across the UK, and the Medicines and Healthcare products Regulatory Agency (MHRA). Each participating site undertakes local review and issues R&D approval according to UK HRA processes. As the trial involves incapacitated adults, all consent processes comply with the EU clinical trials regulations as written into UK law. Trial results will be disseminated through publications, conference presentations, and media engagement. Trial data will be uploaded to the EudraCT database (https://eudract.ema.europa.eu/).

For peer review only - http://bmjopen.bmj.com/site/about/guidelines.xhtml

Trial Management and Oversight

The trial is coordinated by a Project Management Group, including trial managers and coordinators, clinical investigators, and the statistics teams (see author contributions).

A Trial Steering Committee (TSC) is overseeing the conduct and progress of the trial, comprising an independent Chair, a PPI representative, and more than 70% independent clinical and methodology experts. All members sign a TSC charter.

An independent Data Monitoring Committee (DMC) is overseeing the safety of participants in the trial with an agreed DMC Charter to determine Terms of Reference. Given the caution around use in younger patients, the DMC is specifically monitoring safety and outcomes in younger versus older patient group throughout the trial.

The trial sponsor is the ACCORD joint research office of the University of Edinburgh and Lothian Health Board (https://www.accord.scot/). Indemnity for participants is provided through joint sponsorship by the University of Edinburgh and NHS Lothian.

All data are managed according to the General Data Protection Regulations (GDPR)

The funder and sponsor were not involved in design, but reviewed and approved the protocol and amendments. Neither have involvement in analysis, interpretation, or report writing. The sponsor is monitoring the trial.

Current Status

The trial recruited its first patient in December 2018. An internal feasibility pilot was successfully completed, and the funder approved progression to complete the full trial. Recruitment was severely affected by the COVID19 pandemic, with many sites closed for much of 2020-21. The trial re-opened in late 2020, but recruitment was affected by ICU pressures and research capacity during 2021-22. The funder requested a review of trial status and proposals to complete the trial in August 2022. The modelling work for a revised sample size, and considerations of plans to complete the trial recruitment, were concluded in October 2022. The final plan was approved by the funder and sponsor in February 2023, with a proposed recruitment end date of November 2023. Current protocol is version 7 (25th April 2023).

Author Contributions:

TSW, LMA, JN, CJW, RAP, NL, KK, B C-B, DFMcA, PD, MPW, ACG, GDP, MCR, BB, AMacL, RG, and VP designed the trial and led the funding application. All contributed to writing the detailed protocol. In addition JB, DH, AG, AMcD, and LE contributed to protocol development, implementation, monitoring, and amendments. The Process Evaluation was designed by LMA, LE, KK, BB, and TSW. The statistical design was led by RAP, JN, and CJW. The Health economic evaluation was designed by SM. TSW is Chief Investigator.

Funding statement:

This work is supported by the NIHR Health Technology Assessment Programme (HTA 16/93/01). The views expressed are those of the authors and not necessarily those of the

For peer review only - http://bmjopen.bmj.com/site/about/guidelines.xhtml

NIHR or the Department of Health and Social Care. The NIHR Clinical Research Network (CRN) supports the trial.

Competing interests statement.

None of the authors report any relevant competing interests in relation to commercial companies or entities relevant to the A2B trial. No authors report any similar competing interests for spouses or children. Other than a clinical and academic interest in sedation management and its treatment, no authors declare any non-financial competing interests relevant to the A2B trial.

Data Access

Trial data will be held within the University of Edinburgh. Requests to access the full trial dataset will be considered on an individual request basis.

For peer review only

For peer review only - http://bmjopen.bmj.com/site/about/guidelines.xhtml

Figure Legends

Figure 1: Hierarchical design and analytics framework used in the A2B trial. Note: All hypothesis tests performed using a one-sided 2.5% significance level in the original design

For peer review only

For peer review only - http://bmjopen.bmj.com/site/about/guidelines.xhtml

References

1. Adhikari NK, Fowler RA, Bhagwanjee S, Rubenfeld GD. Critical care and the global burden of critical illness in adults. Lancet (London, England). 2010;376(9749):1339-46.
2. Barr J, Fraser GL, Puntillo K, Ely EW, Gelinas C, Dasta JF, et al. Clinical practice guidelines for the management of pain, agitation, and delirium in adult patients in the intensive care unit. Critical care medicine. 2013;41(1):263-306.
3. Vincent JL, Shehabi Y, Walsh TS, Pandharipande PP, Ball JA, Spronk P, et al. Comfort and patient-centred care without excessive sedation: the eCASH concept. Intensive care medicine. 2016;42(6):962-71.
4. Reade MC, Finfer S. Sedation and delirium in intensive care. The New England journal of medicine. 2014;370(16):1567.
5. Jackson DL, Proudfoot CW, Cann KF, Walsh T. A systematic review of the impact of sedation practice in the ICU on resource use, costs and patient safety. Critical care (London, England). 2010;14(2):R59.
6. Aitken LM, Kydonaki K, Blackwood B, Trahair LG, Purssell E, Sekhon M, et al. Inconsistent relationship between depth of sedation and intensive care outcome: systematic review and meta-analysis. Thorax. 2021;76(11):1089-98.
7. Nikayin S, Rabiee A, Hashem MD, Huang M, Bienvenu OJ, Turnbull AE, et al. Anxiety symptoms in survivors of critical illness: a systematic review and meta-analysis. General hospital psychiatry. 2016;43:23-9.
8. Parker AM, Sricharoenchai T, Raparla S, Schneck KW, Bienvenu OJ, Needham DM. Posttraumatic stress disorder in critical illness survivors: a metaanalysis. Critical care medicine. 2015;43(5):1121-9.
9. Rabiee A, Nikayin S, Hashem MD, Huang M, Dinglas VD, Bienvenu OJ, et al. Depressive Symptoms After Critical Illness: A Systematic Review and Meta-Analysis. Critical care medicine. 2016;44(9):1744-53.
10. Wade D, Hardy R, Howell D, Mythen M. Identifying clinical and acute psychological risk factors for PTSD after critical care: a systematic review. Minerva anestesiologica. 2013;79(8):944-63.
11. Aitken LM, Castillo MI, Ullman A, Engstrom A, Cunningham K, Rattray J. What is the relationship between elements of ICU treatment and memories after discharge in adult ICU survivors? Australian critical care : official journal of the Confederation of Australian Critical Care Nurses. 2016;29(1):5-14; quiz 5.
12. Gertler R, Brown HC, Mitchell DH, Silvius EN. Dexmedetomidine: a novel sedative-analgesic agent. Proceedings (Baylor University Medical Center). 2001;14(1):13-21.
13. Nguyen V, Tiemann D, Park E, Salehi A. Alpha-2 Agonists. Anesthesiology clinics. 2017;35(2):233-45.
14. Li A, Yuen VM, Goulay-Dufay S, Kwok PC. Pharmacokinetics and pharmacodynamics of dexmedetomidine. Drug development and industrial pharmacy. 2016;42(12):1917-27.
15. Jamadarkhana S, Gopal S. Clonidine in adults as a sedative agent in the intensive care unit. Journal of anaesthesiology, clinical pharmacology. 2010;26(4):439-45.
16. Luz M, Brandão Barreto B, de Castro REV, Salluh J, Dal-Pizzol F, Araujo C, et al. Practices in sedation, analgesia, mobilization, delirium, and sleep deprivation in adult intensive care units (SAMDS-ICU): an international survey before and during the COVID-19 pandemic. Ann Intensive Care. 2022;12(1):9.
17. Wang JG, Belley-Cote E, Burry L, Duffett M, Karachi T, Perri D, et al. Clonidine for sedation in the critically ill: a systematic review and meta-analysis. Critical care (London, England). 2017;21(1):75.
18. Lewis K, Alshamsi F, Carayannopoulos KL, Granholm A, Piticaru J, Al Duhailib Z, et al. Dexmedetomidine vs other sedatives in critically ill mechanically ventilated adults: a systematic review and meta-analysis of randomized trials. Intensive Care Med. 2022;48(7):811-40.
19. Heybati K, Zhou F, Ali S, Deng J, Mohananey D, Villablanca P, et al. Outcomes of dexmedetomidine versus propofol sedation in critically ill adults requiring mechanical ventilation: a systematic review and meta-analysis of randomised controlled trials. Br J Anaesth. 2022;129(4):515-26.
20. Shehabi Y, Howe BD, Bellomo R, Arabi YM, Bailey M, Bass FE, et al. Early Sedation with Dexmedetomidine in Critically Ill Patients. N Engl J Med. 2019;380(26):2506-17.
21. Shehabi Y, Serpa Neto A, Howe BD, Bellomo R, Arabi YM, Bailey M, et al. Early sedation with dexmedetomidine in ventilated critically ill patients and heterogeneity of treatment effect in the SPICE III randomised controlled trial. Intensive Care Med. 2021;47(4):455-66.
22. Agency EM. Dexmedetomidine: Increased risk of mortality in intensive care unit

(ICU) patients ≤65 years 2022 [Available from: https://www.ema.europa.eu/en/documents/dhpc/direct-healthcare-professional-communication-dhpc-dexmedetomidine-increased-risk-mortality-intensive_en.pdf.
23. Skivington K, Matthews L, Simpson SA, Craig P, Baird J, Blazeby JM, et al. A new framework for developing and evaluating complex interventions: update of Medical Research Council guidance. Bmj. 2021;374:n2061.

For peer review only - http://bmjopen.bmj.com/site/about/guidelines.xhtml

24. Walsh TS, Kydonaki K, Lee RJ, Everingham K, Antonelli J, Harkness RT, et al. Development of Process Control Methodology for Tracking the Quality and Safety of Pain, Agitation, and Sedation Management in Critical Care Units. Critical care medicine. 2016;44(3):564-74.
25. Ely EW, Inouye SK, Bernard GR, Gordon S, Francis J, May L, et al. Delirium in mechanically ventilated patients: validity and reliability of the confusion assessment method for the intensive care unit (CAM-ICU). Jama. 2001;286(21):2703-10.
26. Rattray J, Johnston M, Wildsmith JA. The intensive care experience: development of the ICE questionnaire. Journal of advanced nursing. 2004;47(1):64-73.
27. Parker RA. Overcoming Obstacles to Deriving Sample Size Calculations: Experiences of a Biostatistician. Sage Research Methods Cases: Medicine and Health. 2020.
28. Walsh TS, Kydonaki K, Antonelli J, Stephen J, Lee RJ, Everingham K, et al. Staff education, regular sedation and analgesia quality feedback, and a sedation monitoring technology for improving sedation and analgesia quality for critically ill, mechanically ventilated patients: a cluster randomised trial. The Lancet Respiratory medicine. 2016;4(10):807-17.
29. van den Boogaard M, Pickkers P, Slooter AJ, Kuiper MA, Spronk PE, van der Voort PH, et al. Development and validation of PRE-DELIRIC (PREdiction of DELIRium in ICu patients) delirium prediction model for intensive care patients: observational multicentre study. BMJ (Clinical research ed). 2012;344:e420.

For peer review only

For peer review only - http://bmjopen.bmj.com/site/about/guidelines.xhtml

For peer review only

For peer review only - http://bmjopen.bmj.com/site/about/guidelines.xhtml

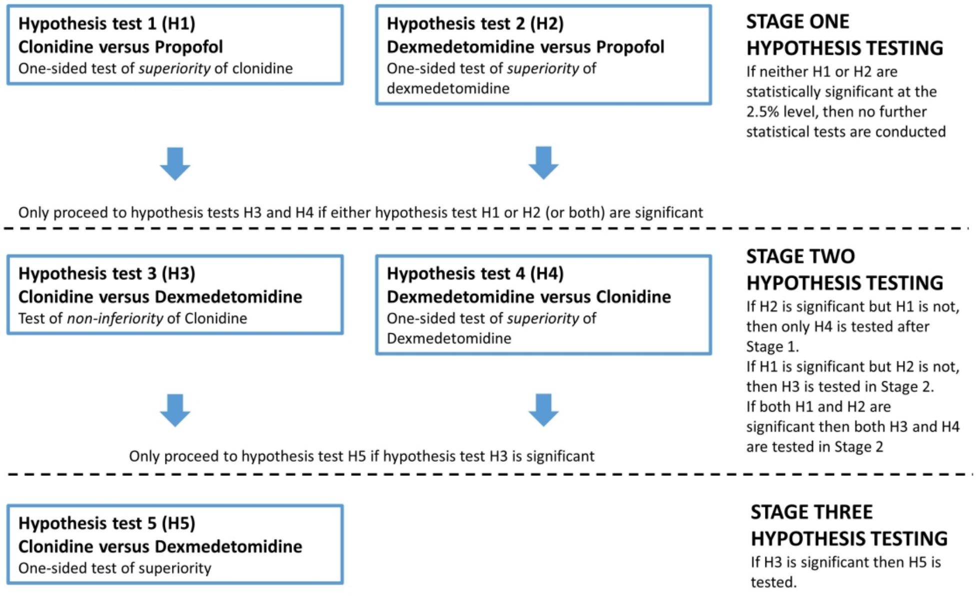

254x190mm (300 x 300 DPI)

Appendix

Contents

Appendix 1
Figure illustrating the consent process utilised for enrolling patients in the absence of patient capacity 2
Example of Consent Form – Personal Legal Representative Consent form 2
Example of consent form 3
Weight-based drug dosing algorithms used in the A2B trial 5
Clonidine Drug Regimen 5
Dexmedetomidine Drug regimen 6
Clinical management algorithms to guide dosing of intervention drugs in the A2B trial 7
CLONIDINE Flowchart 8
Dexmedetomidine Flowchart 10
Usual Care (propofol) flowchart 12
Trial Estimand (see also Statistical Analysis Plan) 14
Health Economic Evaluation 16
Overview 16
Within-trial analysis 16
Lifetime analysis 17

For peer review only

Figure illustrating the consent process utilised for enrolling patients in the absence of patient capacity

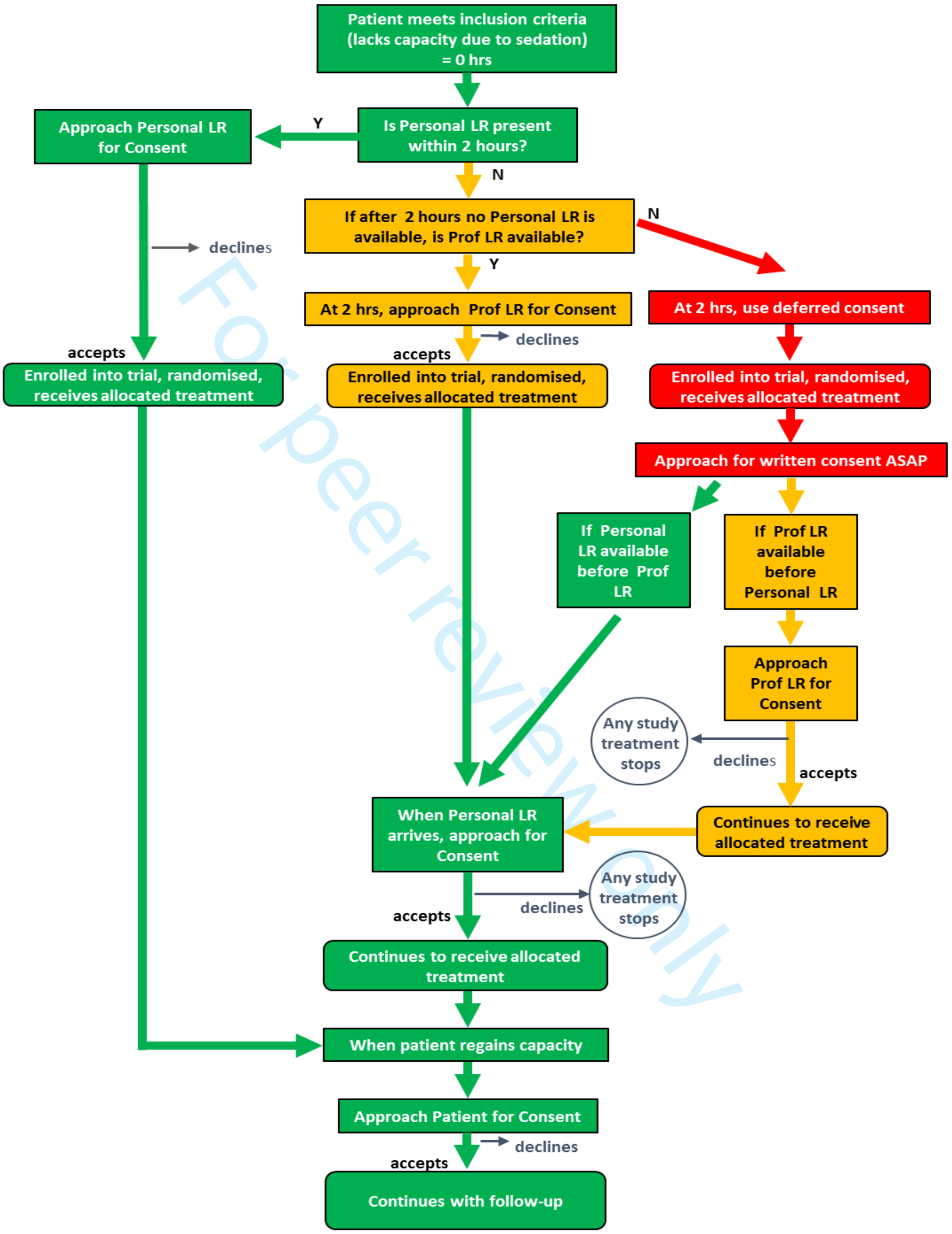

Personal LR = Personal Legal Representative Prof LR = Professional Legal Representative

Example of Consent Form – Personal Legal Representative Consent form

(Additional consent forms used for Professional Legal Representative Consent, and for Patient Consent to remain in trial (once regained capacity)

Example of consent form

Participant ID:		Centre ID	

CONSENT FORM
England, Wales, Northern Ireland
Guardian or Nearest Relative
(Personal Legal Representative – Pre randomisation)

ALPHA-2 AGONISTS FOR SEDATION TO PRODUCE BETTER OUTCOMES FROM CRITICAL ILLNESS (‘A2B TRIAL’)

Please **initial** box

1. I confirm that I have read and understand the Personal LR Pre-randomisation information sheet England/Wales/Northern Ireland (18MAY2023 V2.0) for the above study. I have had the opportunity to consider the information, ask questions and have had these questions answered satisfactorily. ☐
2. I understand that my relative’s participation is voluntary and that I am free to withdraw my consent at any time without giving any reason and without my relative’s medical care and/or legal rights being affected. ☐
3. I give permission for the research team to access my relative’s medical records for the purposes of this research study ☐
4. I understand that relevant sections of my relative’s medical notes and data collected during the study may be looked at by individuals from the Sponsor (University of Edinburgh and/or NHS Lothian), from the NHS organisation or other regulatory authorities where it is relevant to their taking part in this research. I give permission for these individuals to have access to my relative’s data and/or medical records. ☐
5. I give permission for my relative’s personal information (including name, address, date of birth, telephone number and consent form) to be passed to the University of Edinburgh and Edinburgh Clinical Trials Unit for administration of the study and follow-up purposes. ☐
6. I give permission for my relative’s hospital number to be collected and passed to the University of Edinburgh and Edinburgh Clinical Trials Unit. ☐
7. I agree that the information held and maintained by NHS Digital and other central UK NHS bodies may be used to provide information about my relative’s health status. ☐
8. I agree to my relative taking part in the substudy which would involve giving two 20ml blood samples which will be used to study inflammation in the blood and for genetic DNA analysis. Yes ☐ No ☐
9. I give permission for DNA analysis, including whole genome sequencing, to be conducted on my relative’s samples Yes ☐ No ☐
10. I agree that information collected about my relative can be used to support other research in the future, and may be shared anonymously with other researchers. Yes ☐ No ☐
11. I agree that my relative’s blood and DNA samples can be used to support other research in the future, and may be shared anonymously with other researchers. Yes ☐ No ☐
12. I agree to provide my opinion on my relative’s level of comfort and my ability to communicate with them and I give my permission for this data to be used. ☐
13. I agree to my relative taking part in the above study ☐

For peer review only - http://bmjopen.bmj.com/site/about/guidelines.xhtml

Participant ID:			Centre ID

I understand that my relative's data will not be shared beyond those noted on the consent form and that access will be managed via a secure system.

Please **initial** box.

☐ I confirm that I am Personal Legal Representative for ____

Relationship to participant ____

Name of Person Giving Consent	Date	Time	Signature
Name of person receiving consent	Date	Time	Signature

For peer review only

For peer review only - http://bmjopen.bmj.com/site/about/guidelines.xhtml

Weight-based drug dosing algorithms used in the A2B trial

Clonidine Drug Regimen

Weight Based Infusion Rates in mls per hour for Clonidine 15micrograms per ml

	Patient's weight (actual) in kilograms											
**Dose in micrograms per kilogram per hour**	**45**	**50**	**55**	**60**	**65**	**70**	**75**	**80**	**85**	**90**	**95**	**≥100**
**0.1**	0.3	0.3	0.4	0.4	0.4	0.5	0.5	0.5	0.6	0.6	0.6	0.7
**0.2**	0.6	0.7	0.7	0.8	0.9	0.9	1.0	1.1	1.1	1.2	1.3	1.3
**0.3**	0.9	1.0	1.1	1.2	1.3	1.4	1.5	1.6	1.7	1.8	1.9	2.0
**0.4**	1.2	1.3	1.5	1.6	1.7	1.9	2.0	2.1	2.3	2.4	2.5	2.7
**0.5**	1.5	1.7	1.8	2.0	2.2	2.3	2.5	2.7	2.8	3.0	3.2	3.3
**0.6**	1.8	2.0	2.2	2.4	2.6	2.8	3.0	3.2	3.4	3.6	3.8	4.0
**0.7**	2.1	2.3	2.6	2.8	3.0	3.3	3.5	3.7	4.0	4.2	4.4	4.7
**0.8**	2.4	2.7	2.9	3.2	3.5	3.7	4.0	4.3	4.5	4.8	5.1	5.3
**0.9**	2.7	3.0	3.3	3.6	3.9	4.2	4.5	4.8	5.1	5.4	5.7	6.0
**1.0**	3.0	3.3	3.7	4.0	4.3	4.7	5.0	5.3	5.7	6.0	6.3	6.7
**1.1**	3.3	3.7	4.0	4.4	4.8	5.1	5.5	5.9	6.2	6.6	7.0	7.3
**1.2**	3.6	4.0	4.4	4.8	5.2	5.6	6.0	6.4	6.8	7.2	7.6	8.0
**1.3**	3.9	4.3	4.8	5.2	5.6	6.1	6.5	6.9	7.4	7.8	8.2	8.7
**1.4**	4.2	4.7	5.1	5.6	6.1	6.5	7.0	7.5	7.9	8.4	8.9	9.3
**1.5**	4.5	5.0	5.5	6.0	6.5	7.0	7.5	8.0	8.5	9.0	9.5	10.0
**1.6**	4.8	5.3	5.9	6.4	6.9	7.5	8.0	8.5	9.1	9.6	10.1	10.7
**1.7**	5.1	5.7	6.2	6.8	7.4	7.9	8.5	9.1	9.6	10.2	10.8	11.3
**1.8**	5.4	6.0	6.6	7.2	7.8	8.4	9.0	9.6	10.2	10.8	11.4	12.0
**1.9**	5.7	6.3	7.0	7.6	8.2	8.9	9.5	10.1	10.8	11.4	12.0	12.7
**2.0**	6.0	6.7	7.3	8.0	8.7	9.3	10.0	10.7	11.3	12.0	12.7	13.3

For patients who weigh less than 45kg, please contact the trial management team for specific advice prior to randomisation.

For peer review only - http://bmjopen.bmj.com/site/about/guidelines.xhtml

Patients who weigh greater than 100kg will be dosed using the regimen suggested for 100kg weight. Please contact the trial management team for advice if required prior to randomisation.

Dexmedetomidine Drug regimen

Weight Based Infusion Rates in <u>mls per hour</u> for Dexmedetomidine 8 micrograms per ml

Dose in micrograms per kilogram per hour	Patient's weight (actual) in kilograms											
	45	50	55	60	65	70	75	80	85	90	95	≥100
**0.1**	0.6	0.6	0.7	0.8	0.8	0.9	0.9	1.0	1.1	1.1	1.2	1.3
**0.2**	1.1	1.3	1.4	1.5	1.6	1.8	1.9	2.0	2.1	2.3	2.4	2.5
**0.3**	1.7	1.9	2.1	2.3	2.4	2.6	2.8	3.0	3.2	3.4	3.6	3.8
**0.4**	2.3	2.5	2.8	3.0	3.3	3.5	3.8	4.0	4.3	4.5	4.8	5.0
**0.5**	2.8	3.1	3.4	3.8	4.1	4.4	4.7	5.0	5.3	5.6	5.9	6.3
**0.6**	3.4	3.8	4.1	4.5	4.9	5.3	5.6	6.0	6.4	6.8	7.1	7.5
**0.7**	3.9	4.4	4.8	5.3	5.7	6.1	6.6	7.0	7.4	7.9	8.3	8.8
**0.8**	4.5	5.0	5.5	6.0	6.5	7.0	7.5	8.0	8.5	9.0	9.5	10.0
**0.9**	5.1	5.6	6.2	6.8	7.3	7.9	8.4	9.0	9.6	10.1	10.7	11.3
**1.0**	5.6	6.3	6.9	7.5	8.1	8.8	9.4	10.0	10.6	11.3	11.9	12.5
**1.1**	6.2	6.9	7.6	8.3	8.9	9.6	10.3	11.0	11.7	12.4	13.1	13.8
**1.2**	6.8	7.5	8.3	9.0	9.8	10.5	11.3	12.0	12.8	13.5	14.3	15.0
**1.3**	7.3	8.1	8.9	9.8	10.6	11.4	12.2	13.0	13.8	14.6	15.4	16.3
**1.4**	7.9	8.8	9.6	10.5	11.4	12.3	13.1	14.0	14.9	15.8	16.6	17.5

For patients who weigh less than 45kg, please contact the trial management team for specific advice prior to randomisation.

Patients who weigh greater than 100kg will be dosed using the regimen suggested for 100kg weight. Please contact the trial management team for advice if required prior to randomisation.

For peer review only - http://bmjopen.bmj.com/site/about/guidelines.xhtml

Clinical management algorithms to guide dosing of intervention drugs in the A2B trial

For peer review only

For peer review only - http://bmjopen.bmj.com/site/about/guidelines.xhtml

CLONIDINE Flowchart

An ICU Sedation Study

Clonidine Group Sedation Flowchart

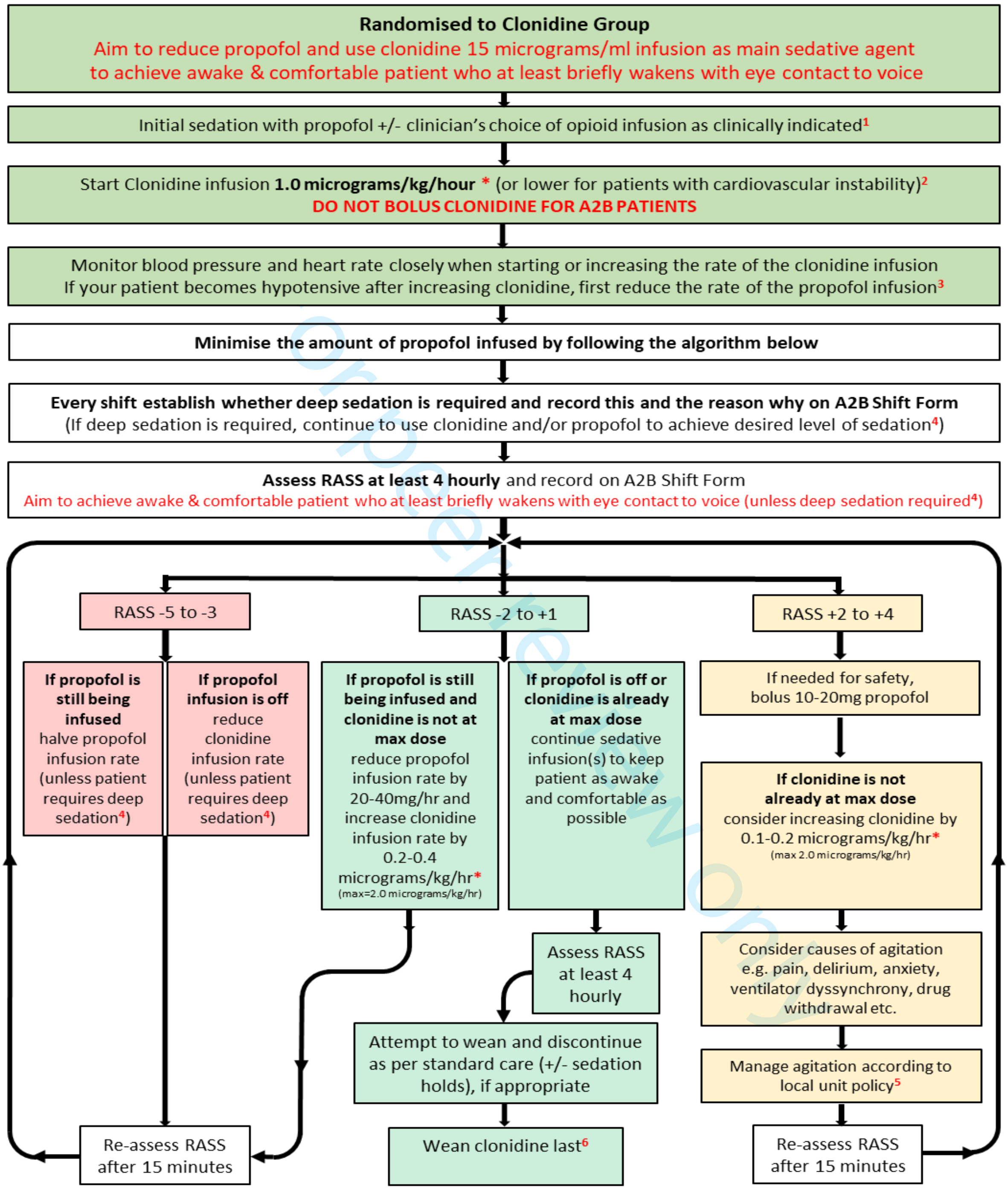

* **See A2B Clonidine infusion table for mls/hr infusion rates for patient weight**

[1] additional opioid boluses can be given as required

[2] if on more than 0.15 micrograms/kg/min noradrenaline to maintain target MAP, use lower starting dose initially

[3] PTO for advice on managing severe bradycardia or hypotension on the reverse of this page

[4] PTO for deep sedation advice on the reverse of this page

[5] dexmedetomidine should not be prescribed for the Clonidine group

[6] PTO for weaning advice on the reverse of this page

An ICU Sedation Study

For peer review only - http://bmjopen.bmj.com/site/about/guidelines.xhtml

Clonidine Group Sedation

A2B An ICU Sedation Study

TARGET: most awake and comfortable state considered safe (the least awake target will be "briefly wakens with eye contact to voice")

- Primary sedative agent is **CLONIDINE** (diluted with 5% glucose or 0.9% NaCl solution to a concentration of **15 micrograms per ml**)
- **Aim to reduce/stop propofol infusion.**
- All patients should receive opioid infusions for analgesia as clinically indicated at the discretion of clinical teams.
- **NB Clonidine has analgesic properties, so it may be possible to reduce opioids if pain is well controlled.**

Drugs you should not give:

- **Dexmedetomidine should <u>not</u> be used as first line sedation during the intervention period.**

How to manage agitation (RASS +2 TO +4)

- Maintain patient/staff safety by bolusing propofol if needed.
- Once agitation is under control, try to identify and manage the cause, using local unit policy for the management of pain/delirium/anxiety/withdrawal etc.
- Anticipate and avoid agitation; use opioid analgesia in advance of uncomfortable or painful interventions or procedures.

What to do if my patient develops severe bradycardia (HR<50 beats per minute)

- **If your patient's heart rate decreases to less than 50 beats per minute, clonidine should be temporarily stopped.**
- NB Clonidine's effect on heart rate can take several hours to resolve, so stopping the clonidine infusion may not immediately resolve bradycardia.
- Seek advice from medical staff who can review and prescribe, glycopyrronium, atropine, dobutamine, adrenaline or other agents, as per your ICU policy.
- Once heart rate is maintained above 50 beats per minute, clonidine should be re-commenced at a dose appropriate to the sedation target, but caution should be used when the clinical target is deep sedation.

What to do if my patient becomes hypotensive

- Hypotension should be treated as per local unit policy. Continuous fluid infusions, fluid challenges and vasopressor infusions are all permitted for patients in the A2B Trial.
- If changes to sedation are required as a result of hypotension, propofol should be decreased before clonidine, unless clinically contraindicated (e.g. if patient requires propofol for neuromuscular blockade).
- Continue clonidine infusion unless causing haemodynamic compromise, such that target MAP cannot be maintained with 0.15 micrograms/kg/min of noradrenaline. If haemodynamically compromised, consider halving the rate of the clonidine infusion, then halving again, or stopping, as needed. Clonidine can be restarted/increased once the patient is more stable, at the discretion of medical staff.
- NB Clonidine's effect on heart rate can take several hours to resolve, so stopping the clonidine infusion may not immediately resolve hypotension.

What if my patient requires deep sedation (RASS -3 TO -5) e.g. for neuromuscular blockade (muscle relaxant)?

- If medical staff have asked to keep your patient deeply sedated, please record the reason deep sedation was requested on the A2B Shift Form.
- **Patients who require neuromuscular blockade after randomisation must receive adequate sedation as per standard care, at the discretion of the treating clinician, to prevent awareness during paralysis. Clonidine alone may not provide adequate sedation to prevent awareness.**
- It is suggested that clonidine is titrated up to the maximum tolerated dose, but if additional sedative drugs are needed to achieve target deep sedation this can be achieved with propofol or benzodiazepine.
- When deep sedation is no longer requested by the caring clinician, aim to use clonidine as main sedative agent as per flowchart over page. It is suggested that any propofol or benzodiazepine sedation is decreased and stopped prior to reducing the dose of clonidine.

What if my patient needs an operative procedure/CT scan/MRI etc.?

- Increase sedation for transfer if needed, using clonidine and/or propofol.
- If required, anaesthesia should be administered as per local perioperative guidelines.
- Continue clonidine infusion unless haemodynamically compromised, in which case consider halving infusion rate and halving again or stopping, as needed.
- Following the procedure, again aim to reduce propofol and use clonidine as main sedative agent as per flowchart over page.

Weaning and discontinuing Clonidine

- **Clonidine can be abruptly discontinued, but slower weaning should be used if withdrawal reactions occur (rebound hypertension or tachycardia, agitation, sweating etc.). However, this should not delay weaning from ventilation as clonidine is relatively free from respiratory depressive effects, so can be safely continued post-extubation.**

PTO for Clonidine Group Sedation Flowchart on reverse of this page

For peer review only

Dexmedetomidine Flowchart

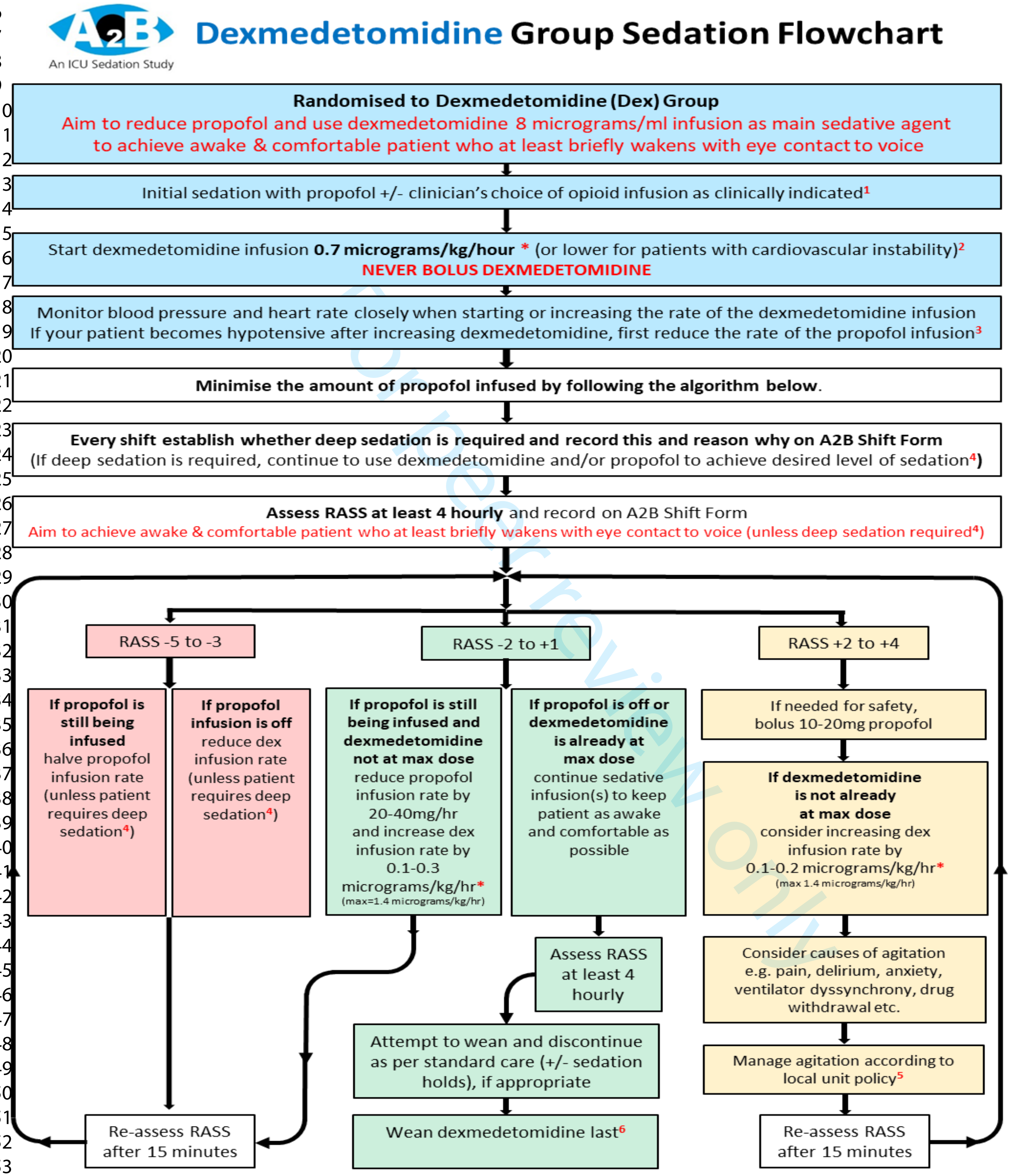

* See A2B Dexmedetomidine infusion table for mls/hr infusion rates for patient weight

[1] additional opioid boluses can be given as required

[2] if on more than 0.15 micrograms/kg/min noradrenaline to maintain target MAP, use lower starting dose initially

[3] PTO for advice on managing severe bradycardia or hypotension on the reverse of this page

[4] PTO for deep sedation advice on the reverse of this page

[5] clonidine should not be prescribed for the dexmedetomidine group

[6] PTO for weaning advice on the reverse of this page

For peer review only - http://bmjopen.bmj.com/site/about/guidelines.xhtml

Dexmedetomidine (Dex) Group Sedation

An ICU Sedation Study

TARGET: most awake and comfortable state considered safe (the least awake target will be "briefly wakens with eye contact to voice")

- Primary sedative agent is **DEXMEDETOMIDINE** (diluted with 5% glucose or 0.9% NaCl solution to a concentration of **8 micrograms per ml**)
- **Aim to reduce/stop propofol infusion.**
- All patients should receive opioid infusions for analgesia as clinically indicated at the discretion of clinical teams.
- **NB Dexmedetomidine has analgesic properties, so it may be possible to reduce opioids if pain is well controlled.**

Drugs you should not give:

- **Clonidine should <u>not</u> be used as first line sedation during the intervention period.**

How to manage agitation (RASS +2 TO +4)

- Maintain patient/staff safety by bolusing propofol if needed.
- Once agitation is under control, try to identify and manage the cause, using local unit policy for the management of pain/delirium/anxiety/withdrawal etc.
- Anticipate and avoid agitation; use opioid analgesia in advance of uncomfortable or painful interventions or procedures.

What to do if my patient develops severe bradycardia (HR<50 beats per minute)

- **If your patient's heart rate decreases to less than 50 beats per minute, dexmedetomidine should be temporarily stopped.**
- NB Dexmedetomidine's effect on heart rate can take several hours to resolve, so stopping the dexmedetomidine infusion may not immediately resolve bradycardia.
- Seek advice from medical staff who can review and prescribe, glycopyrronium, atropine, dobutamine, adrenaline or other agents, as per your ICU policy.
- Once heart rate is maintained above 50 beats per minute, dexmedetomidine should be re-commenced at a dose appropriate to the sedation target, but caution should be used when the clinical target is deep sedation.

What to do if my patient becomes hypotensive

- Hypotension should be treated as per local unit policy. Continuous fluid infusions, fluid challenges and vasopressor infusions are all permitted for patients in the A2B Trial.
- If changes to sedation are required as a result of hypotension, propofol should be decreased before dexmedetomidine, unless clinically contraindicated (e.g. if patient requires propofol for neuromuscular blockade).
- Continue dexmedetomidine infusion unless causing haemodynamic compromise, such that target MAP cannot be maintained with 0.15 micrograms/kg/min of noradrenaline. If haemodynamically compromised, consider halving the rate of the dexmedetomidine infusion, then halving again, or stopping, as needed. Dexmedetomidine can be restarted/increased once the patient is more stable, at the discretion of medical staff.
- NB Dexmedetomidine's effect on heart rate can take several hours to resolve, so stopping the dexmedetomidine infusion may not immediately resolve hypotension.

What if my patient requires deep sedation (RASS -3 TO -5) e.g. for neuromuscular blockade (muscle relaxant)?

- If medical staff have asked to keep your patient deeply sedated, please record the reason deep sedation was requested on the A2B Shift Form.
- **Patients who require neuromuscular blockade after randomisation must receive adequate sedation as per standard care at the discretion of the treating clinician, to prevent awareness during paralysis. Dexmedetomidine alone may not provide adequate sedation to prevent awareness.**
- It is suggested that dexmedetomidine is titrated up to the maximum tolerated dose, but if additional sedative drugs are needed to achieve target deep sedation this can be achieved with propofol or benzodiazepine.
- When deep sedation is no longer requested by the caring clinician, aim to use dexmedetomidine as main sedative agent as per flowchart over page. It is suggested that any propofol or benzodiazepine sedation is decreased and stopped prior to reducing the dose of dexmedetomidine.

What if my patient needs an operative procedure/CT scan/MRI etc.?

- Increase sedation for transfer if needed, using dexmedetomidine and/or propofol.
- If required, anaesthesia should be administered as per local perioperative guidelines.
- Continue dexmedetomidine infusion unless haemodynamically compromised, in which case consider halving infusion rate and halving again or stopping, as needed.
- Following the procedure, again aim to reduce propofol and use dexmedetomidine as main sedative agent as per flowchart over page.

Weaning and discontinuing Dexmedetomidine

- **Dexmedetomidine can be abruptly discontinued, but slower weaning should be used if withdrawal reactions occur (rebound hypertension or tachycardia, agitation, sweating etc.). However, this should not delay weaning from ventilation as dexmedetomidine is relatively free from respiratory depressive effects, so can be safely continued post-extubation.**

PTO for Dexmedetomidine Group Sedation Flowchart on reverse of this page

For peer review only - http://bmjopen.bmj.com/site/about/guidelines.xhtml

Usual Care (propofol) flowchart

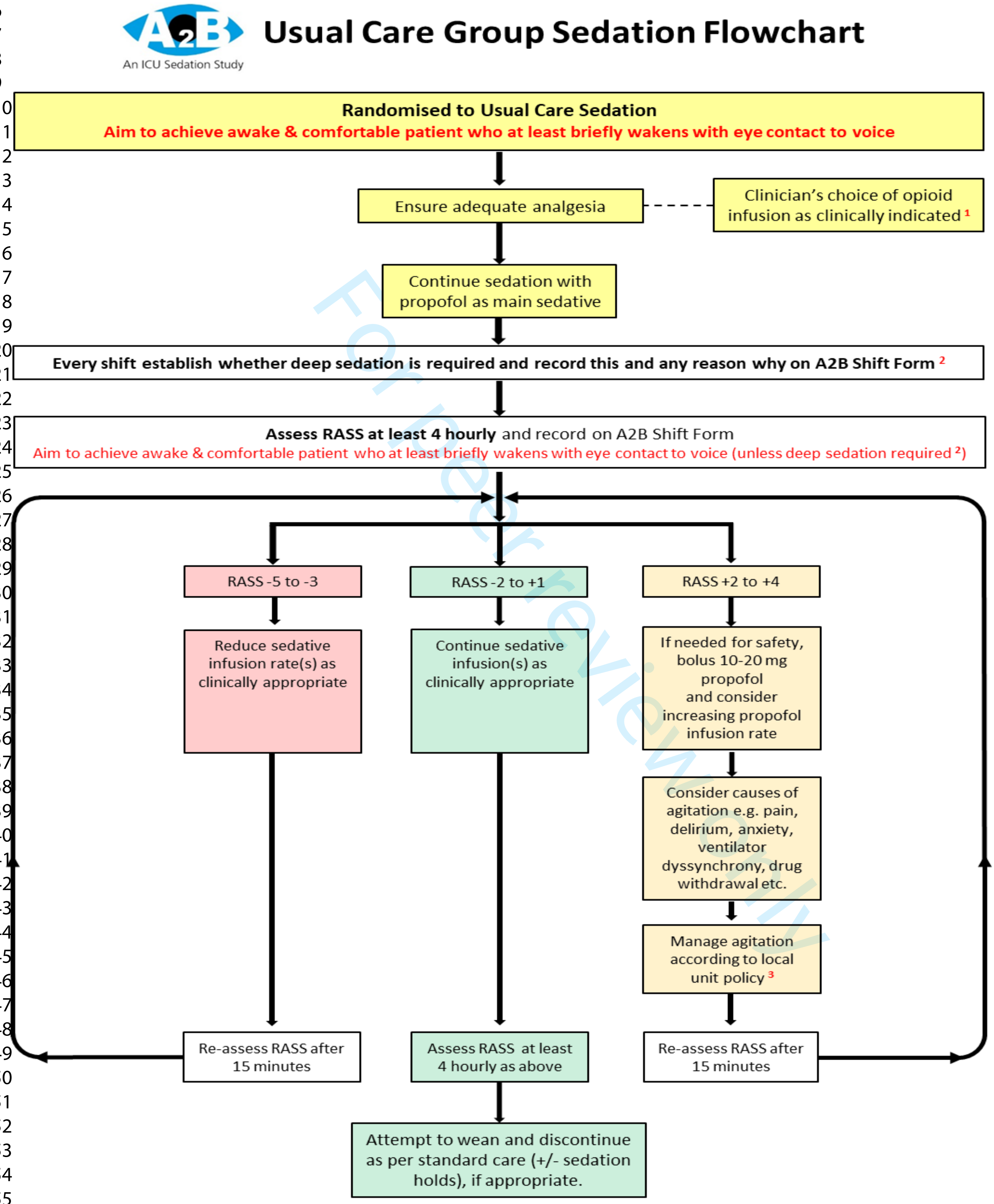

[1] additional opioid boluses can be given as required
[2] PTO for deep sedation advice on the reverse of this page
[3] See advice in "Drugs you should not give" on the reverse of this page before using Dexmedetomidine or Clonidine

For peer review only - http://bmjopen.bmj.com/site/about/guidelines.xhtml

Usual Care Group Sedation

TARGET: most awake and comfortable state considered safe (the least awake target will be "briefly wakens with eye contact to voice")

- Primary sedative agent is **PROPOFOL** (either **1% or 2%**).
- All patients should receive opioid infusions for analgesia as clinically indicated at the discretion of clinical teams

Drugs you should not give:

- **Neither clonidine nor dexmedetomidine should be used as first line sedation during the intervention period.**
- However, either clonidine or dexmedetomidine can be used at the discretion of the clinical teams for specific indications such as agitation, severe delirium or during difficult weaning.

How to manage agitation (RASS +2 TO +4)

- Maintain patient/staff safety by bolusing propofol if needed.
- Once agitation is under control, try to identify and manage the cause, using local unit policy for the management of pain/delirium/anxiety/withdrawal etc.
- Anticipate and avoid agitation; use opioid analgesia in advance of uncomfortable or painful interventions or procedures.

What to do if my patient develops severe bradycardia (HR<50/min)

- Seek advice from medical staff who can review and prescribe, glycopyrronium, atropine, dobutamine, adrenaline or other agents, as per your ICU policy.

What to do if my patient becomes hypotensive

- Hypotension should be treated as per local unit policy. Continuous fluid infusions, fluid challenges and vasopressor infusions are all permitted for patients in the A2B Trial.

What if my patient requires deep sedation (RASS -3 TO -5) e.g. for neuromuscular blockade (muscle relaxant)?

- If medical staff have asked to keep your patient deeply sedated, please record the reason deep sedation was requested on the A2B Shift Form.
- **Patients who require neuromuscular blockade after randomisation must receive adequate sedation as per standard care at the discretion of the treating clinician, to prevent awareness during paralysis.**
- If additional sedative drugs are needed to achieve target deep sedation this can be achieved with benzodiazepine.
- When deep sedation is no longer requested by the caring clinician, aim to use propofol as main sedative agent as per flowchart over page.

What if my patient needs an operative procedure?

- Increase sedation for transfer if needed.
- If required, anaesthesia should be administered as per local perioperative guidelines.
- Following the operative procedure, again aim to use propofol as main sedative agent as per flowchart over page.

What if my patient needs an operative procedure/CT scan/MRI etc.?

- Increase sedation for transfer to theatre if needed using propofol.
- If required, anaesthesia should be administered as per local perioperative guidelines.

PTO for Usual Care Group Sedation Flowchart on reverse of this page

For peer review only - http://bmjopen.bmj.com/site/about/guidelines.xhtml

Trial Estimand (see also Statistical Analysis Plan)

Here we define the estimand for the primary analysis of the primary outcome in the trial, in line with the draft addendum to ICH E9 (Statistical Principles for Clinical Trials): ICH E9(R1), Defining the Appropriate Estimand for a Clinical Trial/Sensitivity Analyses.

Population Adult ICU patients enrolled within 48h of MV starting in ICU and expected to require sedation with propofol and MV for at least 48h, at least 24h of which would be after randomisation. Long-term home ventilation, terminal illness, selected diagnoses, allergy to study medication, pregnancy and expected death within 24h are exclusion criteria.

Variable Time to successful extubation post-randomisation (hours).

Population-level Summary Cumulative incidence function of time to extubation; sub-distribution hazard ratio (HR)

The following Intercurrent Events have been identified which would prevent measurement of the primary outcome or change the interpretation of the measured primary outcome:

1. Death before the time point at which randomised treatment is due to start.

2. (a) Dexmedetomidine allocated in randomisation but not started

(b) Clonidine allocated in randomisation but not started

3. Additional propofol being administered when cardiovascular side effects have limited the escalation of dexmedetomidine or clonidine.

4. Additional propofol being administered when non-cardiovascular side effects have limited the escalation of dexmedetomidine or clonidine.

5. Death before successful extubation.

6. Patient withdrawal from intervention and follow-up (situation where deferred consent is not granted is a subset of such events).

7. Transfer to another ICU before successful extubation.

8. Use of dexmedetomidine as main sedative in usual care group.

9. Use of clonidine as main sedative in usual care group.

10. Use of rescue medication in the presence of agitation or delirium.

Events 1, 2(a), 2(b), 8 and 9 are expected to be rare and no specific actions will be taken: analysis of these events will be by intention to treat, except for event 1 which will be handled in the same way as event 5.

For peer review only - http://bmjopen.bmj.com/site/about/guidelines.xhtml

Events 3 and 4 will be dealt with using an intention to treat approach. Non-cardiovascular side-effects will mostly be sedation-related and therefore will be further analysed as secondary outcomes.

Event 5 will be treated as a competing risk for the primary outcome, and will therefore be analysed using a hypothetical strategy.

Event 6 will also be handled using a hypothetical strategy, in which the time to extubation will be censored at the point of withdrawal and the withdrawals will be assumed to lead to missing at random (MAR) data on the primary outcome. Complete follow up should still be possible for most participants in whom event 7 occurs; if not, the hypothetical strategy used for event 6 will also be implemented.

Event 10 is analogous to events 8 and 9 but applies to all treatment arms and medications. An intention to treat approach will be used for this event.

Full details of the methods of dealing with the above intercurrent events will be incorporated in the statistical analysis plan.

For peer review only - http://bmjopen.bmj.com/site/about/guidelines.xhtml

Health Economic Evaluation

Overview

The significant cost differences between dexmedetomidine and both usual care and clonidine make the health economic evaluation especially relevant. Of importance, the cost of dexmedetomidine has decreased substantially during the conduct of the trial, such that the cost-effectiveness of the interventions may be different in the context of pricing in the 'post trial' era. Additional drug costs associated with α2-agonists should be balanced against potential cost savings from reductions in ICU and hospital stay and altered outcomes. We will undertake a detailed analysis of the cost and cost-effectiveness of dexmedetomidine, clonidine and usual care. Our analysis will conform to accepted economic evaluation methods in the UK. The comparisons made in the economic evaluation will reflect those of the staged hypothesis tests for the primary outcome (figure 1). We will estimate costs and cost-effectiveness for the 'within-trial' period (6 months/short-run model) and also over the expected lifetime of the patient ('lifetime'/long-run model). Costs will be assessed from the perspective of the NHS and personal social services (PSS). For both the within-trial and lifetime analyses we will undertake cost-utility analyses, estimating incremental cost per quality-adjusted life year (QALY) gained.

Within-trial analysis

For the within-trial analysis we will calculate detailed cost information on index hospitalisation for every patient from ICU admission to hospital discharge and during 6 months follow-up. Patient-level resource use data will be collected on items including: sedative drugs; costs associated with managing adverse events (e.g., delirium); length of ICU stay and hospital wards; use of MV; and co-prescribed medications. Patient-level resource use data will be collected post-discharge up to 6 months using questionnaires at 3 and 6 month follow-up: hospital re-admissions; A&E and outpatient visits; GP and nurse visits in clinic and at home; medications; care home admissions; any other contacts with primary and social care (e.g., physiotherapist, occupational therapist, social worker, counsellor). Unit costs will be obtained from published sources and inflated where appropriate before being applied to the volume of resource use data.

QALYs will be calculated based on the HRQoL and mortality data collected during the trial. HRQoL will be measured using the EQ-5D-5L (www.euroqol.org), which we will collect at 30 days, 3 and 6 months (see table 1). As patients recruited to the trial will be critically ill, completion of the EQ-5D-5L at baseline will not be possible. Previous studies have assumed a common baseline value for all patients (e.g., zero). We will use this approach and two alternatives. First, we will estimate baseline utility scores for patients using proxy responses from a person who knows the patient. In this case we will use the proxy version of the EQ-5D-5L (by the patient's spouse, parent or (adult) child). The type of proxy respondent will be controlled for in subsequent analyses. Second, we will ask patients to retrospectively record their baseline EQ-5D-5L at the 30 day follow-up point. We will evaluate QALYs associated with each strategy using all three approaches; the base case approach will use the proxy responses. Patients who die will be assigned a utility value of zero at the date of death and all subsequent time periods. Patient-specific utility profiles will be constructed assuming a

For peer review only - http://bmjopen.bmj.com/site/about/guidelines.xhtml

straight line relation between each of the patients' EQ-5D-5L scores at each follow-up point. The QALYs experienced by each patient from baseline to 6 months will be calculated as the area underneath this profile.

Multiple imputation by chained equations will be used to deal with missing HRQoL and resource use values. Subsequent analyses of imputed data will include variance correction factors to account for additional variability introduced into parameter values as a result of the imputation process. Cost-effectiveness will be calculated as the mean cost difference between groups divided by the mean difference in outcomes (QALYs) to give the incremental cost-effectiveness ratio (ICER). We will also calculate net monetary benefits (NMBs). We will subject the results to extensive deterministic (one-, two- and multi-way) and probabilistic sensitivity analysis. For the latter, non-parametric methods for calculating confidence intervals around the ICERs and NMBs based on bootstrapped estimates of the mean cost and QALY differences will be used. These methods will appropriately account for the multiple imputation of the missing data. The bootstrap replications will also be used to construct cost-effectiveness acceptability curves, which will show the probability that each strategy is cost-effective at 6 months for different values willingness to pay for additional QALYs by the NHS.

Lifetime analysis

In the lifetime model cost-effectiveness will be calculated in terms of the incremental cost per QALY gained. A review of the NIHR HTA website (www.hta.ac.uk/project/htapubs.asp) and the NHS Economic Evaluation Database (NHS-EED, www.crd.york.ac.uk https://www.crd.york.ac.uk/CRDWeb/) (last search 15/05/2017) reveals there have been no previous analyses to evaluate lifetime cost-effectiveness of the study strategies. Given this paucity of evidence, we will develop a de novo cost-effectiveness model that will be populated based on available evidence, including the data collected during the trial. We will: [1] design a lifetime model to characterize health states of ICU survivors; [2] populate the model using data identified from the trial and published literature and routine sources; [3] relate outcomes from the trial to final outcomes, expressed in terms of QALYs; and [4] identify which parameters in the model are most uncertain and are important drivers of cost-effectiveness. The model is likely to use a similar structure to a previous economic evaluation of long-term cost-effectiveness for ICU patients in the UK. Survival analysis of the RCT data will provide the basis for extrapolating any within-trial differences in costs and QALYs. The model will use external data on long-term survival of ICU survivors, including from co-applicants expert in this area (Lone, Walsh). Specific details of the data to be used to populate the model will be determined following the development of the structure and the systematic searches of the literature to identify existing models. We will undertake deterministic (one-, two- and multi-way) and probabilistic sensitivity analysis, the latter assuming appropriate distributions and parameter values. We will combine data on incremental costs with epidemiological data on projected patient numbers and undertake a budget impact analysis to evaluate what the total cost impact of each strategy would be were it to be scaled up; budget impact will be calculated separately for ICU-related costs only, the within-trial period and using a lifetime time horizon, as each might be appropriate

For peer review only - http://bmjopen.bmj.com/site/about/guidelines.xhtml

for different decision-makers. We will also use the probabilistic sensitivity analyses combined with the epidemiological information on projected patient numbers to undertake a value of information analysis to evaluate the potential economic value of future research on this topic.

For peer review only

For peer review only - http://bmjopen.bmj.com/site/about/guidelines.xhtml

Statistical Analysis Plan	A2B
Version No	2.0
Date Finalised	dd/mm/yyyy

An ICU Sedation Study

Alpha 2 agonists for sedation to produce better outcomes from critical illness (A2B Trial): A randomised, parallel-group, allocation concealed, controlled, open, phase 3 pragmatic clinical and cost- effectiveness trial with internal pilot

A2B Trial

Statistical Analysis Plan (Version as at 28th July 2023)

CONFIDENTIAL

**Version No**	2.0
**Date Finalised**	dd/mm/yyyy
**Author(s)**	Richard Parker (until unblinded on 2 August 2019)<br>Christopher Weir (from 2 August 2019)
**CI Name**	Professor Timothy Walsh
**CI Email address**	timothy.walsh@ed.ac.uk

**Funder**	NIHR Health Technology Assessment
**Funding Reference Number**	HTA 16/93/01
**Sponsor**	The University of Edinburgh & Lothian Health Board ACCORD
**EudraCT Number**	2018-001650-98
**ClinicalTrials.gov**	NCT03653832

Signatures	
**Trial Statistician: Prof Christopher Weir**	**Date:**
**Chief Investigator: Prof Timothy Walsh**	**Date:**

Document Control

**Version No**	**Date**	**Summary of Revisions**
1.0	22/01/2021	Initial Creation
2.0	dd/mm/yyyy	Incorporated modified sample size calculation. Updated to reflect latest ECTU SAP template (V4.0, 25Mar2021).

For peer review only

For peer review only - http://bmjopen.bmj.com/site/about/guidelines.xhtml

Statistical Analysis Plan	A2B
Version No	2.0
Date Finalised	dd/mm/yyyy

Table of Contents

List of Abbreviations ... 3

1. Introduction ... 4

2. Statistical Methods section from the protocol ... 4

8.2 PROPOSED ANALYSES ... 4

8.2.1 Estimand ... 4

8.2.2 Statistical analysis ... 5

3. Overall Statistical Principles ... 7

3.1 Analysis populations ... 7

3.2 Outcomes ... 8

4. List of Analyses ... 9

4.1 Recruitment, retention and missing data ... 10

4.2 Baseline characteristics ... 10

4.3 Primary outcome (primary analysis) ... 11

4.4 Primary outcome (supplementary analyses) ... 12

4.5 Subgroup analyses ... 13

4.6 Secondary outcomes ... 13

4.6.1 Missing data handling: secondary outcomes ... 15

4.7 Safety ... 15

4.8 Concomitant medications ... 16

4.9 Intervention dose, fidelity and reach ... 16

4.10 Protocol deviations and violations ... 16

5. Validation and QC ... 16

6. Data sharing ... 17

7. References ... 17

Appendix 1 Sedation Quality Assessment Tool (SQAT) ... 19

Appendix 2 PRE-DELIRIC score derivation ... 20

Appendix 3 Data completeness and intervention adherence ... 22

For peer review only - http://bmjopen.bmj.com/site/about/guidelines.xhtml

Statistical Analysis Plan A2B
Version No 2.0
Date Finalised dd/mm/yyyy

List of Abbreviations

Abbreviation	Full name
AE	Adverse event
CAM-ICU	Confusion-Assessment Method for ICU
CI	Confidence interval
CONSORT	CONsolidated Standards Of Reporting Trials
CPAP	Continuous positive airway pressure
CRF	Case report form
EQ-5D-5L	EuroQol instrument with five levels of severity in each of five dimensions
EudraCT	European Clinical Trials Database
HADS	Hospital Anxiety and Depression Scale
HR	Hazard ratio
HTA	Health Technology Assessment
ICE-Q	Intensive Care Experience Questionnaire
ICU	Intensive care unit
ICH	International Council for Harmonisation of Technical Requirements for Pharmaceuticals for Human Use
IES-R	Impact of Events Scale – Revised
MV	Mechanical ventilation
NIHR	National Institute for Health Research
NIV	Non-invasive mechanical ventilation
OR	Odds ratio
RASS	Richmond Agitation and Sedation Scale
SD	Standard deviation
SOFA	Sequential Organ Failure Assessment
SQAT	Sedation Quality Assessment Tool
T-MoCA	Montreal Cognitive Assessment tool (telephone version)

For peer review only - http://bmjopen.bmj.com/site/about/guidelines.xhtml

Statistical Analysis Plan	A2B
Version No	2.0
Date Finalised	dd/mm/yyyy

1. Introduction

A2B is a randomised, parallel-group, allocation concealed, controlled, open, multi-centre, phase 3 pragmatic clinical and cost- effectiveness trial with internal pilot. Adult intensive care unit (ICU) patients expected to require at least 24 hours further mechanical ventilation (MV) will be randomised within 48 hours of starting MV. Patients with primary brain injury; post-cardiac arrest; status epilepticus; and peripheral nervous system disease will be excluded. 1437 patients will be randomised to receive sedation using dexmedetomidine or clonidine or 'usual care' sedation in a 1:1:1 ratio. To simplify the enrolment process randomisation will be stratified by site alone.

This statistical analysis plan is written with reference to protocol version 7, dated 25 April 2023. Its scope covers the end of trial analysis for A2B, with the exception of the health economic evaluation, the process evaluation (apart from quantitative descriptions of fidelity to the intervention) and the mechanistic sub-study of pro- and anti-inflammatory mediators which will all be documented separately.

2. Statistical Methods section from the protocol

8.2 PROPOSED ANALYSES

8.2.1 Estimand

Here we define the estimand for the primary analysis of the primary outcome in the trial, in line with the draft addendum to ICH E9 (Statistical Principles for Clinical Trials): ICH E9(R1), Defining the Appropriate Estimand for a Clinical Trial/Sensitivity Analyses.

Population *Adult ICU patients enrolled within 48h of MV starting in ICU and expected to require sedation with propofol and MV for at least 48h, at least 24h of which would be after randomisation. Long-term home ventilation, terminal illness, selected diagnoses, allergy to study medication, pregnancy and expected death within 24h are exclusion criteria.*

Variable *Time to successful extubation post-randomisation (hours).*

Population-level Summary *Cumulative incidence function of time to extubation; sub-distribution hazard ratio (HR)*

The following ***Intercurrent Events*** *have been identified which would prevent measurement of the primary outcome or change the interpretation of the measured primary outcome:*

1. *Death before the time point at which randomised treatment is due to start.*
2. *(a) Dexmedetomidine allocated in randomisation but not started*
   (b) Clonidine allocated in randomisation but not started
3. *Additional propofol being administered when cardiovascular side effects have limited the escalation of dexmedetomidine or clonidine.*
4. *Additional propofol being administered when non-cardiovascular side effects have limited the escalation of dexmedetomidine or clonidine.*
5. *Death before successful extubation.*

For peer review only - http://bmjopen.bmj.com/site/about/guidelines.xhtml

6. *Patient withdrawal from intervention and follow-up (situation where deferred consent is not granted is a subset of such events).*
7. *Transfer to another ICU before successful extubation.*
8. *Use of dexmedetomidine as main sedative in usual care group.*
9. *Use of clonidine as main sedative in usual care group.*
10. *Use of rescue medication [1] in the presence of agitation or delirium.*

Events 1, 2(a), 2(b), 8 and 9 are expected to be rare and no specific actions will be taken: analysis of these events will be by intention to treat, except for event 1 which will be handled in the same way as event 5.

Events 3 and 4 will be dealt with using an intention to treat approach. Non-cardiovascular side-effects will mostly be sedation-related and therefore will be further analysed as secondary outcomes.

Event 5 will be treated as a competing risk for the primary outcome, and will therefore be analysed using a hypothetical strategy.

Event 6 will also be handled using a hypothetical strategy, in which the time to extubation will be censored at the point of withdrawal and the withdrawals will be assumed to lead to missing at random (MAR) data on the primary outcome. Complete follow up should still be possible for most participants in whom event 7 occurs; if not, the hypothetical strategy used for event 6 will also be implemented.

Event 10 is analogous to events 8 and 9 but applies to all treatment arms and medications. An intention to treat approach will be used for this event.

Full details of the methods of dealing with the above intercurrent events will be incorporated in the statistical analysis plan.

8.2.2 Statistical analysis

For the primary analysis, a Fine and Gray proportional sub-distribution hazards regression analysis of time from randomisation to successful extubation will be performed (this method allows us to directly model the cumulative incidence of extubation after taking into account the competing risk of mortality) for each hypothesis test permitted under the analytic structure (Figure 1). Results will be expressed as sub-distribution HRs with corresponding 95% confidence intervals and p-values.

The following supplementary analyses will be performed to provide reassurance about the robustness of the main analysis of the primary outcome, for each between-arm comparison:

(i) A Cox frailty proportional hazards regression model will be fitted to the primary outcome, with censoring for deaths or loss to follow-up in ICU while on MV. Although death in ICU may be considered a competing risk, this modelling approach allows us to estimate the instantaneous risk of experiencing a successful extubation event at time t given that the patient is still alive at time t (in the literature this is called the "cause-specific hazard" of extubation for patients who have not yet died). Site will be included in the model as a random effect.

[1] Rescue medication is recorded as haloperidol, quetiapine, dexmedetomidine, midazolam, olanzapine, clonidine, lorazepam or other

For peer review only - http://bmjopen.bmj.com/site/about/guidelines.xhtml

Statistical Analysis Plan	A2B
Version No	2.0
Date Finalised	dd/mm/yyyy

(ii) *A Cox frailty regression analysis of time from randomisation to ICU mortality while on ventilation. Patients experiencing successful extubation events or loss to follow-up prior to mortality will be censored. This analysis will provide "cause-specific" HRs for patients on MV to support the primary analysis results. Site will be included in the model as a random effect.*

(iii) *A Cox frailty regression analysis of time to all-cause mortality, with censoring only for patients lost to follow-up during the six months follow-up period. This analysis will allow us to compare the risk of overall mortality across trial arms for all patients, regardless of whether or not patients are still on MV. Site will be included in the model as a random effect.*

(iv) *For each participant, the proportion of care periods will be recorded in which propofol treatment was maintained even though dexmedetomidine or clonidine had not been up-titrated to its maximum dose and had no dose-limiting side-effects. As an exploratory analysis, the main analysis of the primary outcome will be repeated using the adherence analysis set (section 8.2.3) rather than the full analysis set.*

For the secondary outcomes other than mortality, formal hypothesis testing will not be performed but point estimates and 95% confidence intervals for pairwise differences between randomised groups will be calculated. A trial analysis plan providing full details will be finalised prior to locking the trial data base.

The hierarchical hypothesis testing framework for analysis of the primary outcome, which controls the overall type I error to be at most 6.5% across the multiple analyses being performed, is also outlined in protocol Figure 1:

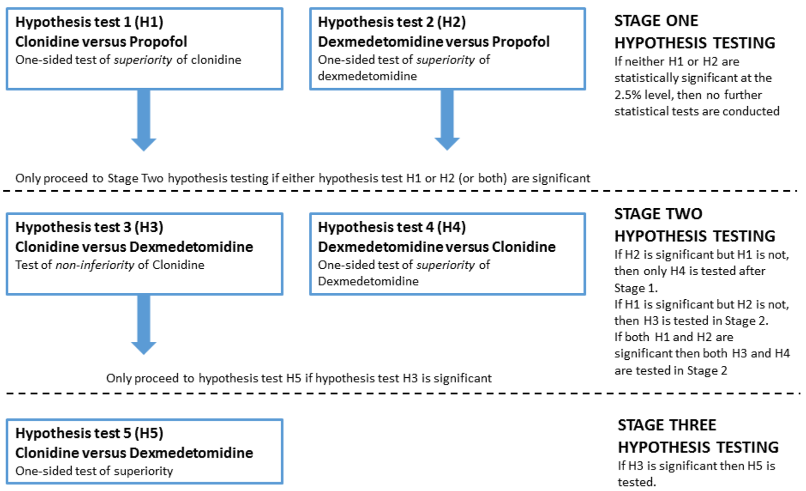

For peer review only - http://bmjopen.bmj.com/site/about/guidelines.xhtml

Figure 1: Analytic framework which efficiently tests the trial questions using a hierarchical analytic structure with serial gatekeeping to preserve study power. Hypothesis tests will be performed using a 2.5% one-sided significance level, with the exception of the non-inferiority test H3 which will use a 4% one-sided significance level.

3. Overall Statistical Principles

The Stage 1 hypothesis testing of the superiority of each of clonidine and dexmedetomidine versus propofol will be carried out at the one-sided 2.5% significance level. The Stage 2 hypothesis of non-inferiority of clonidine to dexmedetomidine will be performed with a one-sided 4% significance level. The Stage 2 hypothesis of superiority of dexmedetomidine to clonidine will have a one-sided 2.5% significance level. Finally, in Stage 3, there will be a possible test of superiority of clonidine versus dexmedetomidine at the one-sided 2.5% significance level. All hypothesis tests on the primary outcome are arranged in a hierarchical structure, with serial gatekeeping, to ensure overall control of the type 1 error to at most 6.5%.

Categorical variables will be summarised using frequencies and percentages; continuous variables will be summarised using the mean, standard deviation (SD), median, lower quartile, upper quartile, minimum and maximum values.

Analyses of outcomes will adjust for site as a random effect, since site is included as a stratification factor in the randomisation.

Generally speaking, missing data will be handled according to the principles outlined in the A2B estimand, described in protocol section 8.2.1. Participants randomised in error despite ineligibility, becoming ineligible before drug administered, or being withdrawn from the trial by family members prior to intervention, will be reported in the participant flow summary but will not be included in efficacy or safety analyses as no further data will be gathered on these participants.

Outliers will be identified by viewing boxplots of the outcome variables of interest. All analyses will include outliers as standard; where data are present which lie more than 4 standard deviations away from the mean, a sensitivity analysis will be performed removing these data values to determine the robustness of the findings in the analysis where outliers were included.

The planned analyses will be performed using the SAS statistical software, version 9.4 or later. Following the end of trial, defined as the date of the last follow-up of the final participant, the planned analyses will be performed once data querying has been completed and the locking of the trial database has been documented.

3.1 Analysis populations

Full analysis set
All participants randomised, analysed according to their allocated treatment group regardless of the treatment actually received.

Adherence analysis set
The **adherence analysis set** will be all randomised participants in the **full analysis set** who, in the dexmedetomidine group received any dexmedetomidine on the day of randomisation; in the clonidine

For peer review only - http://bmjopen.bmj.com/site/about/guidelines.xhtml

group received any clonidine on the day of randomisation; or in the usual care group received neither dexmedetomidine nor clonidine (except as rescue medication for agitation) on the day of randomisation.

3.2 Outcomes

Primary outcome

- Time to successful extubation post-randomisation (hours).

A successful first extubation from mechanical ventilation will be defined as follows:

a) From endotracheal extubation: time of first extubation that is followed by 48 hours of spontaneous breathing.

b) From tracheostomy: time of extubation will be defined as the start time of the first period during which a patient receives support not exceeding 5 cmH_2O CPAP with less or equal to pressure support ventilation of 5cmH_2O for a continuous period of 48 hours. Decannulation during this period will count as being free from mechanical ventilation.

c) From non-invasive mechanical ventilation (NIV): time of extubation will be the start time of the first period during which a patient receives support not exceeding 5 cmH_2O CPAP via mask/hood for a continuous period of 48 hours. NIV patients receiving any pressure supported breaths will not be considered to be spontaneously breathing unassisted.

NB: The use of high flow nasal oxygen will not be counted as mechanical ventilation, so a patient on high nasal flow oxygen alone will be considered to be spontaneously breathing unassisted.

Secondary outcomes

Secondary outcomes are listed in priority order. Specifically, mortality forms a component of the primary outcome time to successful extubation. Outcomes listed from Length of ICU Stay to Patient Experience of ICU Care are outcomes specified in the NIHR HTA briefing document for this commissioned funding call. The remaining outcomes are listed in order of priority according to guidance from patient and public involvement representatives.

S1 Mortality
ICU; hospital; 30 days; 90 days; 180 days post-randomisation

S2 Length of ICU stay (days from randomisation to ICU discharge)

S3 Sedation quality, measured by Richmond Agitation and Sedation Scale (RASS)
- Measured four-hourly during mechanical ventilation until primary outcome recorded, summarised as lowest and highest day shift and night shift RASS scores over time

S4 Sedation quality, measured during mechanical ventilation until primary outcome recorded by Sedation Quality Assessment Tool (SQAT- Appendix 1)
Four sedation quality states:
1. Overall optimum sedation (no agitation;no unnecessary deep sedation;no pain behaviour)
2. Agitation
3. Unnecessary deep sedation (RASS -4/-5 without clinical indication)
4. Pain (presence of pain behaviour based on limb movement and ventilation compliance)

S5 Time to first optimum sedation

For peer review only - http://bmjopen.bmj.com/site/about/guidelines.xhtml

- Hours from randomisation to first RASS score of -2 or greater
- Days from randomisation to first day with SQAT optimum sedation

S6 Delirium prior to successful extubation, assessed by Confusion-Assessment Method for ICU (CAM-ICU)
- Occurrence prior to successful extubation (binary outcome)
- Days with delirium or coma prior to successful extubation (continuous outcome)

S7 One or more pre-defined cardiac adverse events
(of those recorded daily: severe bradycardia; cardiac arrhythmias; cardiac arrest)

S8 Health-related Quality of Life, measured by recall prior to hospital admission, and at 30, 90 and 180 days after randomisation using the EuroQol EQ-5D-5L instrument

S9 Patient Ability to Communicate Pain and Ability to Cooperate with Care
Binary assessments for each 12 hours nursing shift:
- Was patient able to communicate pain?
- Was patient able to cooperate with care?

S10 Patient experience of ICU care, measured at 90 days after randomisation using the Intensive Care Experience Questionnaire (ICE-Q)
Provides numeric score in four domains:

1. Awareness of Surroundings (9 items; score range 9-45)
2. Frightening Experiences (6 items; score range 6-35)
3. Recall of Experiences (5 items; score range 5-25)
4. Satisfaction with Care (4 items; score range 4-20)

S11 Relative/partner/friend (PerLR) assessment of comfort and communication, measured daily during mechanical ventilation
Binary assessment for each question:

1. Does the patient appear awake to the visitor?
2. Does the patient seem comfortable to the visitor?
3. Does the visitor feel they can communicate with the patient?

S12 Anxiety and depression, measured at 180 days post randomisation using the Hospital Anxiety and Depression Scale (HADS) questionnaire

S13 Post-traumatic stress, measured at 180 days post randomisation using the Impact of Events Scale-revised (IES-R)

S14 Cognitive function, measured at 180 days post randomisation using the Montreal Cognitive Assessment tool telephone version (T-MoCA)

Commented [CW1]: Postal version no longer mentioned in protocol

4. List of Analyses

This analysis plan describes the end of trial statistical analyses to be performed on A2B, excluding analysis of the mechanistic sub-study of putative pro- and anti-inflammatory mediators (protocol

For peer review only - http://bmjopen.bmj.com/site/about/guidelines.xhtml

section 11), the health economics analyses and the process evaluation components of the trial. However, quantitative assessment of fidelity from the process evaluation is included in the scope of this analysis plan.

4.1 Recruitment, retention and missing data

A CONSORT flow diagram will be constructed. For EudraCT reporting purposes, enrolment will also be summarised into age categories 18-64; 65-84; and 85+ years.

The number and percentage of patients who were later found to be ineligible for the trial even though they were randomised will be summarised by randomised group, as will the number of patients formally withdrawn and the reason for withdrawal (if available). The number and percentage of patients with missing primary outcome data will be reported by randomised treatment allocation. No formal statistical testing will be performed.

4.2 Baseline characteristics

The following baseline characteristics will be summarised by treatment group and overall. A further descriptive summary will assess any association between the Covid-19 pandemic and participant characteristics. The baseline characteristics summary will be further stratified by randomisations occurring up to and including 23 March 2020 and those occurring after 23 March 2020.

Age (years)
Age (by EudraCT reporting categories)
Gender
Pre-randomisation:
- Estimated weight (kg)
- RASS
- CAM-ICU (unless RASS -4 or -5, or is -3 but the assessor is unable to assess CAM-ICU status)

Functional comorbidity index (Groll et al, 2005) (total count; and 18 separate items)
Medical history:
- Portal hypertension
- Biopsy proven cirrhosis
- Hepatic encephalopathy
- Alcohol dependence
- Drug dependence

Type of admission (Trauma, Non-trauma medical, Non-trauma surgical; Planned, Unplanned)
Diagnosis at admission (Medical)
Diagnosis at admission (Surgical)
Pre-randomisation sedatives (Propofol, Midazolam, Fentanyl, Alfentanil, Morphine, Remifentanil, Dexmedetomidine, Clonidine, Haloperidol, Diazepam, Other (free text)) For each report frequency and summarise dose, in units specified on CRF.
SOFA score (excluding neurological SOFA) (Singer et al, 2016)
Pre-randomisation blood results:
- Haemoglobin g/L
- Lymphocytes x10^9/L
- Sodium mmol/L
- Urea mmol/L
- Albumin g/L
- White cell count x10^9/L
- APTT ratio

For peer review only - http://bmjopen.bmj.com/site/about/guidelines.xhtml

Potassium mmol/L
eGFR mL/min/1.73m^2
ALT U/L

Blood gases:

H^+
pH
PaO_2 kPa
$PaCO_2$ kPa
Standard bicarbonate mmol/L
Lactate mmol/L

PRE-DELIRIC delirium prediction score (van den Boogaard et al, 2012; Appendix 2) including components:

Apache II score
Infection/sepsis
Antibiotics given during first 24 hours in ICU
Sepsis
Septic shock
Coma RASS -4/-5 for at least 8 hours in first 24 hours in ICU
If yes, by use of medication / other reason / both medication and other
Total morphine dose in first 24 hours in ICU
None / 0.01-7.1mg / 7.2-18.6mg / 18.7-331.6mg
Any propofol, midazolam or lorazepam use in first 24 hours in ICU
Highest urea value in first 24 hours in ICU (mmol/L)
Metabolic acidosis

Proxy baseline EQ-5D

4.3 Primary outcome (primary analysis)

For the primary analysis, performed on the full analysis set, a Fine and Gray proportional sub-distribution hazards regression analysis (Fine and Gray, 1999) of time from randomisation to successful extubation will be performed (this method allows us to directly model the cumulative incidence of extubation after taking into account the competing risk of mortality, thus implementing the hypothetical strategy outlined in the estimand for intercurrent events 1 and 5) for each hypothesis test permitted under the hierarchical testing structure. Results will be expressed as the sub-distribution hazard ratio (HR) for each of dexmedetomidine and clonidine versus usual care, with corresponding 95% confidence intervals (CI) and p-values from the Fine-Gray model. The exception will be the non-inferiority analysis of clonidine versus dexmedetomidine (hypothesis H3 in protocol figure 1) for which a 96% one-sided non-inferiority CI will be presented. Site will be accounted for in the analysis by implementing the marginal model approach to the Fine and Gray method for clustered data (Zhou et al, 2012). If this aspect of model fitting proves problematic due to sites which have randomised a small number of participants (fewer than 5), we will consider pooling of data from such sites to address this issue.

Intercurrent events 2(a), 2(b), 8 and 9 are expected to be rare and will therefore be handled using the intention to treat approach in the primary analysis of the primary outcome. Events 3 and 4 (propofol use due to cardiovascular and non-cardiovascular side-effects respectively) will also be handled using the intention to treat approach due the pragmatic exploration of the effects of clonidine and dexmedetomidine in A2B. Withdrawals where the participant has not withdrawn permission to use data collected up to the point of withdrawal will have time to extubation censored at the time of withdrawal (intercurrent event 6, missing at random assumption, hypothetical strategy). In the rare

For peer review only - http://bmjopen.bmj.com/site/about/guidelines.xhtml

cases of transfer to another ICU before extubation (intercurrent event 7), follow-up will be continued to extubation where possible but if extubation time is missing it will be censored at the last time at which the extubation status is known (missing at random assumption, hypothetical strategy). Intercurrent event 10 will be handled using intention to treat, again reflecting the treatment policy pragmatic nature of A2B.

The cumulative incidence function (CIF) obtained from the Fine-Gray model for time to successful extubation will be plotted separately for each treatment group; the median time to successful extubation and its 95% CI will be reported by treatment group. As recommended in the CONSORT reporting guidance, the absolute risk difference (and its 95% CI) for each of dexmedetomidine and clonidine versus control will be reported at 7 days after randomisation (the median time on mechanical ventilation under 'usual care' in a real ICU dataset).

Following the strategy recommended by Poythress et al. (2020), the fit of the Fine-Gray model will be evaluated by plotting, by treatment group, the CIF for time to successful extubation from the Fine-Gray model against the nonparametric CIF. If substantial differences occur between the Fine-Gray and nonparametric CIF curves an alternative modelling strategy, such as cause-specific hazards, will be considered.

4.4 Primary outcome (supplementary analyses)

Supplementary analyses will provide reassurance about the robustness of the primary analysis, for each between-arm comparison:

(i) A mixed effects partially proportional hazards regression model will be fitted to the primary outcome of time from randomisation to successful extubation, with censoring for deaths or loss to follow-up in ICU while on MV. Although death in ICU may be considered a competing risk, censoring for deaths allows us to estimate the instantaneous risk of experiencing a successful extubation event at time t given that the patient is still alive at time t (the "cause-specific hazard" of extubation for patients who have not yet died). Site will be included in the model as a random effect, treatment group as a fixed effect. Results will be expressed as the HR for each of dexmedetomidine and clonidine versus usual care, with its corresponding 95% CI and p-value.

(ii) A mixed effects partially proportional hazards regression analysis of time from randomisation to ICU mortality while on MV. Patients experiencing successful extubation events or loss to follow-up prior to mortality will be censored. For patients on MV, this analysis will provide the mortality "cause-specific" HR (and 95% CI) for each of dexmedetomidine and clonidine versus usual care, to support the primary analysis results. Site will be included in the model as a random effect, treatment group as a fixed effect.

(iii) Overall mortality will be analysed using a mixed effects partially proportional hazards regression analysis, see Section 4.6 for details.

(iv) The primary analysis will be repeated, but using the adherence analysis set.

Furthermore, selected baseline characteristics of patients with missing primary outcome data due to withdrawal will be compared descriptively to those with patients who did not withdraw prior to extubation to evaluate the missing at random assumption present in the primary analysis of intercurrent event 6.

Similarly, selected baseline characteristics of patients transferred to another ICU who did not have time to extubation recorded will be compared to those transferred to another ICU who did have it

For peer review only - http://bmjopen.bmj.com/site/about/guidelines.xhtml

recorded, to assess the missing at random assumption being made in the primary analysis of intercurrent event 7.

Finally, further exploratory analysis will assess any association between the Covid-19 pandemic and the primary outcome. Summary descriptive statistics of time to successful extubation will be reported by treatment group and further stratified by the date of the UK lockdown: randomisations occurring up to and including 23 March 2020 versus those occurring after 23 March 2020.

4.5 Subgroup analyses

The primary analysis of the primary outcome will be repeated for the following subgroups specified in the protocol.

(1) Patients with and without sepsis at enrolment to A2B.
(2) Patients with lower or higher delirium risk, as defined by the PRE-DELIRIC delirium risk prediction score. (van den Boogaard et al, 2012) The groups with values above (or including) and below the median PRE-DELIRIC score observed in the trial population will be compared.
(3) Patients with and without organ dysfunction at randomisation. The group with SOFA score values above or equal to the median SOFA score (excluding neurological score) that is present at baseline will be compared with the group with SOFA score values below the median score at baseline.
(4) Age (<64 versus ≥64)

For each subgroup variable, a p-value will be calculated for its interaction with each of dexmedetomidine and clonidine versus usual care. Within each subgroup category, we will calculate the sub-distribution HR and 95% confidence interval for (a) dexmedetomidine versus usual care and (b) clonidine versus usual care and present these in a forest plot. These analyses will be considered exploratory.

For age, an additional exploratory analysis will fit an interaction term based on its continuous value rather than age categories. A cubic B-spline, fractional polynomial or simple quadratic term will be fitted to determine, via a likelihood ratio test, whether there is a significant non-linear relationship between age and the effects of each of dexmedetomidine and clonidine versus usual care.

For the age subgroup, given the findings of the SPICE trial of dexmedetomidine (Shehabi et al., 2019), the above subgroup analysis will also be applied to the mortality secondary outcome **S1**.

4.6 Secondary outcomes

Each secondary outcome will be summarised appropriately, by treatment group and overall. Where informative graphical summaries will also be created. The large number of secondary outcomes means that not all will be included in the mean trial publication text. Instead, **S5**, **S9** and **S11** will be reported in the accompanying supplementary material. Other secondary outcomes for which there is substantial missing data will also be considered for transfer to the supplementary material.

For the secondary outcomes other than **S1**, mortality, formal hypothesis testing will not be performed but point estimates and 95% confidence intervals for pairwise differences between randomised groups will be calculated. P-values will not be reported.

For peer review only - http://bmjopen.bmj.com/site/about/guidelines.xhtml

Statistical Analysis Plan	A2B
Version No	2.0
Date Finalised	dd/mm/yyyy

For secondary outcomes measured at more than one time point following ICU discharge, separate analyses will be performed for each measurement occasion. Secondary outcomes **S9**, **S10** and **S11** will be summarised descriptively (for **S10,** for each of the four domains separately) without any calculation of confidence intervals for differences between groups.

S1 Mortality. A mixed effects partially proportional hazards regression analysis will be used to analyse time to all-cause mortality, with censoring only for patients lost to follow-up during the six months follow-up period. This analysis will allow us to compare the risk of overall mortality, using the HR, 95% CI and p-value, for each of dexmedetomidine and clonidine versus usual care for all patients, regardless of whether or not patients are still on MV. Site will be included in the model as a random effect and treatment group as a fixed effect.

The time to event secondary outcomes **S2** and **S9** will be analysed using the same method as for the primary analysis of the primary outcome (Section 4.3), in order to take account of the potential competing risk of death. The supplementary analyses of Section 4.4 will also be applied for these outcomes. Time to event outcome **S5** will be summarised descriptively but will not be formally analysed.

Binary secondary outcomes (**S6** [delirium occurrence], **S4**, **S7**, **S9**, **S11**) will be analysed by a generalised linear mixed model with a logit link function. Site will be included as a random effect in the model and treatment group as a fixed effect. For outcomes **S4** and **S9** which are measured in multiple care periods, a random effect for participant (nested within site) will also be included. Optimal sedation for outcome **S4** will be reported descriptively as a proportion for each combination of study day and treatment group. It will not be analysed formally. Each of the **S4** SQAT components (freedom from agitation; freedom from pain; and freedom from unnecessary deep sedation) will be reported descriptively as for optimal sedation and in addition will be analysed using the generalised linear mixed model with logit link. Results will be expressed as the odds ratio (OR) for each of dexmedetomidine and clonidine versus usual care, with corresponding 95% CI.

Continuous secondary outcomes (**S3** [highest RASS score recorded daily, regardless of whether clinical need for deep sedation was recorded], **S8**, **S12**, **S13**, **S14**) will be analysed using a normal linear mixed model. Site will be included as a random effect in the model and treatment group as a fixed effect. Outcome **S3** is measured in each care period so a random effect for participant (nested within site) will also be included. For **S3** each of the day shift and night shift highest and lowest RASS will also be summarised graphically up to the occurrence of the successful extubation primary outcome. A proxy for outcome **S8** is measured at baseline and this will be included as a fixed effect in the model. The parameter to be estimated is the adjusted mean difference: dexmedetomidine minus usual care; and clonidine minus usual care. The corresponding 95% CI will also be reported. If the assumption of normality of residuals does not hold (as determined by normal probability plot), the outcome variable will be transformed to rectify this. In the event that the assumption cannot be satisfied, alternative analyses (for example involving categorising the outcome measure) will be conducted. A similar strategy will be applied when residuals versus fitted values demonstrate non-constant variance for an outcome.

The count variable **S6**, delirium or coma days prior to successful extubation, will be analysed using a generalised linear mixed model with a log link (Poisson regression). Number of days prior to successful extubation will be included as an offset term in the model. Site will be included as a random effect in

For peer review only - http://bmjopen.bmj.com/site/about/guidelines.xhtml

the model. The result for each of dexmedetomidine and clonidine versus usual care will be presented as a rate ratio (RR) and 95% confidence interval.

4.6.1 Missing data handling: secondary outcomes

We anticipate minimal rates of missing data for the secondary outcome **S1**, mortality. In cases of missing data, the survival time will be censored at the date last known alive. Missing data on time to event secondary outcomes **S2** and **S9** will be handled using a similar approach to that used for **S1**.

In other secondary outcomes, for which no formal hypothesis testing will be undertaken, the following strategies will be implemented where missing data rates are low (less than 10% overall, and with a no more than 5% difference in the rate across treatment groups). For continuous secondary outcomes a "missing at random" assumption will be applied automatically within the normal linear mixed model, while complete case analyses will be performed for outcomes which are counts or binary variables. In the event of the missing data rate being greater than 10% overall, or differing by more than 5% across treatment groups, multiple imputation strategies will be considered.

4.7 Safety

Safety data will be reported for the full analysis population, according to treatment allocated.

While death will be analysed as a secondary outcome (Section 4.6), only deaths considered related to participation in A2B will be recorded as serious adverse events. Sedation-related adverse events (including hypotension, hypertension, unplanned NG removal, unplanned central line removal, unplanned arterial line removal, unplanned peripheral line removal, unplanned drain removal, unplanned extubation, staff injury as a result of patient, patient injury and ileus) will be reported descriptively: number and percentage by treatment group and overall.

During the recruiting ICU stay (or up to and including study day 28, whichever is earlier) the number and percentage of patients experiencing each of: any adverse event (AE); non-serious adverse event (NSAE); serious adverse event (SAE) and suspected unexpected serious adverse reaction (SUSAR) will be reported, overall and split by trial arm. Tabulations will be split by events occurring pre- and post-randomisation. The numbers of events will also be reported.

The AE, NSAE, SAE and SUSAR tables will also be further categorised by the number and percentage of patients recording an event in each of the MedDRA system organ class categories, with a further sub-categorisation according to verbatim text or MedDRA preferred term as appropriate.

Data listings of all adverse events will be provided by treatment group, according to MedDRA system organ class, verbatim text, severity, seriousness, causality, expectedness and outcome.

Daily data on blood results (platelets, bilirubin, creatinine), respiratory function (FiO_2, PaO_2, SpO_2), blood pressure (lowest systolic BP recorded and corresponding diastolic BP) and urine output (>500mL/day, 200-500mL/day,<200mL/day) will be summarised and presented graphically by ICU study day and treatment group. No formal statistical inference will be performed on these measures. When estimating the mean and SD measures below the limit of quantification (LLQ) will be handled by treating these observations as censored but positive, calculating the likelihood conditional on them being greater than zero. This is strategy M4 from Senn et al., 2012.

For peer review only - http://bmjopen.bmj.com/site/about/guidelines.xhtml

4.8 Concomitant medications

The frequency and percentage (of all those in the full analysis set) of patients in whom rescue medications are administered to decrease sedation when the RASS score is -4/-5 will be reported, overall and by treatment arm.

4.9 Intervention dose, fidelity and reach

Dose

The frequency of RASS assessments recorded per shift will be summarised overall, by treatment group and by study site.

Fidelity

The degree of implementation of various components of the A2B interventions will be summarised using the algorithm outlined in Appendix 3. Reporting will cover completeness of day and night shift forms; responses to deep sedation query; completeness of RASS data; completeness of CAM-ICU data on day and night shifts; completeness of pain behaviour data; deep sedation guidance compliance; number and proportion of care periods for each participant in which each of propofol, dexmedetomidine and clonidine was administered will be summarised overall and by treatment group; and propofol, dexmedetomidine and clonidine administration by study day for participants remaining on mechanical ventilation.

For each treatment group, the proportion of participants receiving propofol treatment on each study day will be reported.

Further evaluation of fidelity will be reported in the qualitative process evaluation.

Reach

The number and percentage of eligible patients recruited will be reported overall and by study site. More extensive analysis of reach will be reported in the qualitative process evaluation.

4.10 Protocol deviations and violations

For events which are specific to a participant, the number and percentage of each of protocol deviations and violations will be presented, split by site, trial arm and overall.

Deviations and violations which cannot be attributed to an individual participant (for example, an issue with a process in a site) will be presented in a line listing.

5. Validation and QC

The following will be performed by a second statistician:

1. Separate programming and checking of the primary and supplementary analyses for the primary outcome (Sections 4.3 and 4.4).

For peer review only - http://bmjopen.bmj.com/site/about/guidelines.xhtml

2. Separate programming and re-analysis of the mortality secondary outcome and all other secondary outcome analyses for which there is at least one statistically significant pairwise comparison (one-sided p-value <0.025) in the first statistician's analysis. If there are more than 10 such secondary outcomes, then 5 of them will be randomly selected for re-analysis.

3. The end of trial statistical report will be read and checked for accuracy and consistency.

6. Data sharing

A file, or set of files, containing an anonymised version of the final analysis data set will be prepared, along with a data dictionary. These will be made available to the Chief Investigator at the end of the analysis phase.

7. References

Andersen PK, Geskus RB, de Witte T, et al. Competing risks in epidemiology: possibilities and pitfalls. International Journal of Epidemiology 2012;41(3):861-70. doi: 10.1093/ije/dyr213

Fine J and Gray R. A proportional hazards model for the subdistribution of a competing risk. Journal of the American Statistical Association 1999;94(446): 496-509. doi:10.2307/2670170

Groll DL, To T, Bombardier C, Wright JG. The development of a comorbidity index with physical function as the outcome. Journal of Clinical Epidemiology 2005;58:595-602.

Noordzij M, Leffondre K, van Stralen KJ, et al. When do we need competing risks methods for survival analysis in nephrology? Nephrology, dialysis, transplantation : official publication of the European Dialysis and Transplant Association - European Renal Association 2013;28(11):2670-7. doi: 10.1093/ndt/gft355

Poythress JC, Yu Lee M, Young J. Planning and analyzing clinical trials with competing risks: Recommendations for choosing appropriate statistical methodology. Pharmaceutical Statistics. 2020;19:4–21. doi:10.1002/pst.1966

Senn S, Holford N, Hockey H. The ghosts of departed quantities: approaches to dealing with observations below the limit of quantitation. Statist. Med. 2012, 31 4280–4295.

Shehabi Y, Howe BD, Bellomo R, Arabi YM, Bailey M, Bass FE, Bin Kadiman S, McArthur CJ, Murray L, Reade MC, Seppelt IM, Takala J, Wise MP and Webb SA, for the ANZICS Clinical Trials Group and the SPICE III Investigators.* Early Sedation with Dexmedetomidine in Critically Ill Patients. N Engl J Med 2019; 380:2506-17.

Singer M, Deutschman CS, Warren Seymour C, Shankar-Hari M, Annane D, Bauer M, Bellomo R, -Bernard GR, Chiche J-D, Coopersmith CM, Hotchkiss RS, Levy MM, Marshall JC, Martin GS, Opal SM, Rubenfeld GD, van der Poll T, Vincent J-L, Angus DC. The Third International Consensus Definitions for Sepsis and Septic Shock (Sepsis-3). JAMA 2016;315(8):801-810. doi:10.1001/jama.2016.0287

For peer review only - http://bmjopen.bmj.com/site/about/guidelines.xhtml

van den Boogaard M, Pickkers P, Slooter AJ, et al. Development and validation of PRE-DELIRIC (PREdiction of DELIRium in ICu patients) delirium prediction model for intensive care patients: observational multicentre study. *BMJ (Clinical research ed)* 2012;344:e420. doi: 10.1136/bmj.e420

Varadhan R, Weiss CO, Segal JB, et al. Evaluating health outcomes in the presence of competing risks: a review of statistical methods and clinical applications. Medical Care 2010;48(6 Suppl):S96-105. doi: 10.1097/MLR.0b013e3181d99107

Zhou B, Fine J, Latouche A, Labopin M. (2012). Competing risks regression for clustered data. Biostatistics 2012;13(3):371-383.

For peer review only - http://bmjopen.bmj.com/site/about/guidelines.xhtml

Statistical Analysis Plan A2B
Version No 2.0
Date Finalised dd/mm/yyyy

Appendix 1 Sedation Quality Assessment Tool (SQAT)

For a given ICU shift, the sedation quality states of SQAT will be derived as:

Agitation Highest RASS +3/+4 (Daily Data Collection CRF)

Unnecessary deep sedation Lowest RASS -4/-5 AND Was the bedside nurse asked by medical staff to keep this patient deeply sedated? = “No” (Daily Data Collection CRF)

Pain Presence of pain behaviour based on:

Limb movement (Response to moving the participant = “Difficult to move most of the time” OR “Actively resisting movement most of the time”) OR
((Compliance with the ventilator = “Tolerating ventilation but coughing/gagging frequently” OR “Unable to control ventilation due to poor patient synchronisation despite different modes tested”) AND Was the participant paralysed throughout the entire nursing shift? = “No”)
(Daily Data Collection CRF)

Overall optimum sedation is present when there is no agitation; no unnecessary deep sedation; and no pain behaviour.

For peer review only - http://bmjopen.bmj.com/site/about/guidelines.xhtml

Statistical Analysis Plan	A2B
Version No	2.0
Date Finalised	dd/mm/yyyy

Appendix 2 PRE-DELIRIC score derivation

The PRE-DELIRIC score will be derived according to the formula in van den Boogaard et al, 2012:

Formula for PRE-DELIRIC model

Risk of delirium = 1/(1+exp−(−6.31
+ 0.04 × age
+ 0.06 × APACHE-II score
+ 0 for non-coma or 0.55 for drug induced coma or 2.70 for miscellaneous coma or 2.84 for combination coma
+ 0 for surgical patients or 0.31 for medical patients or 1.13 for trauma patients or 1.38 for neurology/neurosurgical patients
+ 1.05 for infection
+ 0.29 for metabolic acidosis
+ 0 for no morphine use or 0.41 for 0.01-7.1 mg/24 h morphine use or 0.13 for 7.2-18.6 mg/24 h morphine use or 0.51 for >18.6 mg/24 h morphine use
+ 1.39 for use of sedatives
+ 0.03 × urea concentration (mmol/L)
+ 0.40 for urgent admission))

The scoring system's intercept is expressed as −6.31; the other numbers represent the shrunken regression coefficients (weight) of each risk factor.

Age: Randomisation date minus date of birth (Pre-Randomisation CRF)

APACHE II score: (Baseline CRF)

Coma:

Non-coma	Coma status = "No coma" (Baseline CRF)
Drug induced coma	Coma status = "Coma" AND "With use of medication" (Baseline CRF)
Miscellaneous coma	Coma status = "Coma" AND "Other" (Baseline CRF)
Combination coma	Coma status = "Coma" AND "Combination" (Baseline CRF)

Surgical/Medical/Trauma/Neurology/Neurosurgery:

Surgical	Type of ICU admission = "Non-trauma" AND ("Surgical" NOT (Diagnosis at Admission – Surgical Admission = "Intracerebral haemorrhage" OR "Subdural/epidural haematoma" OR "Subarachnoid haemorrhage" OR "Laminectomy / other spinal cord injury" OR "Craniotomy for neoplasm" OR "Other neurologic diseases"))
Medical	Type of ICU admission = "Non-trauma" AND ("Medical" NOT (Diagnosis at Admission – Medical Admission = "Intracerebral haemorrhage" OR "Subarachnoid haemorrhage" OR "Stroke" OR "Neurologic infection" OR "Neurologic neoplasm" OR "Neuromuscular disease" OR "Seizure" OR "Other neurologic disease"))
Trauma	Type of ICU admission = "Trauma (without traumatic brain injury)"
Neurology/Neurosurgery	Type of ICU admission = "Non-trauma" AND

For peer review only - http://bmjopen.bmj.com/site/about/guidelines.xhtml

((Diagnosis at Admission – Surgical Admission = “Intracerebral haemorrhage” OR “Subdural/epidural haematoma” OR “Subarachnoid haemorrhage” OR “Laminectomy / other spinal cord injury” OR “Craniotomy for neoplasm” OR “Other neurologic diseases”) OR
(Diagnosis at Admission – Medical Admission = “Intracerebral haemorrhage” OR “Subarachnoid haemorrhage” OR “Stroke” OR “Neurologic infection” OR “Neurologic neoplasm” OR “Neuromuscular disease” OR “Seizure” OR “Other neurologic disease”))
(Baseline CRF)

Infection:
Did the participant receive antibiotics for proven or suspected infection during their first 24 hours in ICU? = ”Yes” (Baseline CRF)

Metabolic acidosis:
pH < 7.35 (H+ > 44.7) with bicarbonate < 24 mmol/L in the first 24 hours in ICU? = “Yes” (Baseline CRF)

Morphine use:
Total administered morphine dose in first 24 hours in ICU =
“Morphine use: 0.01 – 7.1 mg” cumulative OR
“Morphine use: 7.2 – 18.6 mg cumulative” OR
“Morphine use: 18.7 – 331.6 mg cumulative”
(Baseline CRF)

Sedatives:
Any use of propofol, midazolam, lorazepam or combination in the first 24 hours in ICU? = “Yes” (Baseline CRF)

Urea concentration:
Please specify the highest serum urea value in the first 24 hours in ICU [mmol/L]
(Baseline CRF)

Urgent admission: Planned Admission = “Unplanned” (Baseline CRF)

For peer review only - http://bmjopen.bmj.com/site/about/guidelines.xhtml

Appendix 3 Data completeness and intervention adherence

Rule 1: Removing non-intervention period days
Remove days on which answer to 'InvasivelyVentilated_YesNoDesc' and 'NonInvVentilation_YesNoDesc' is NO
This will remove the majority of days on which the patient was no longer ventilated during the intervention period. There will be a small number of days on which the response could be NO but the patient is subsequently re-intubated and the primary outcome has not been reached. However, subsequent ventilated days will be included as the answer to this question should revert to YES. For the purpose of tracking data quality this small discrepancy will not be important.
Remaining data should be all days on which patients was receiving mechanical ventilation as defined in the protocol

Rule 2: completeness of day and night shift forms
After rule 1:
Count proportion of 'DSBedsideNurse_YesNoDesc' that response is YES
Count proportion of 'NSBedsideNurse_YesNoDesc' that response is YES
Report this as proportion of 'shift forms' completed by clinical staff during day shift and night shift and overall by site and overall trial

Rule 3: responses to deep sedation query
After rule 1:
Count proportion of 'DSDeepSedation_YesNoNotCollectedDesc' reported for each category
Count proportion of 'NSDeepSedation_YesNoNotCollectedDesc' reported for each category
Report this for day shift and night shift and for overall by site and overall trial

Rule 4: completeness of sedation RASS data
After rule 1:
Report completeness of:
'DSHighestRASS_RASSScoreDesc'
'DSLowestRASS_RASSScoreDesc'
'NSHighestRASS_RASSScoreDesc'
'NSLowestRASS_RASSScoreDesc'
To provide a measure of ability to report a highest and lowest recorded RASS score on each day report:
Proportion of days on which:
'DSHighestRASS_RASSScoreDesc' OR 'NSHighestRASS_RASSScoreDesc' OR BOTH have a RASS score reported
'DSLowestRASS_RASSScoreDesc' OR 'NSLowestRASS_RASSScoreDesc' OR BOTH have a RASS score recorded

Rule 5: completeness of CAM-ICU data
After rule 1:
Report the following:
Day shift
Proportion of day shifts on which 'DSCAMICUPositive_CAMICUPositiveDesc' is YES OR NO (this is a definite response)
Proportion of day shifts on which 'DSCAMICUPositive_CAMICUPositiveDesc' response is 'RASS -3 but clinical team unable to assess CAM-ICU'

For peer review only - http://bmjopen.bmj.com/site/about/guidelines.xhtml

Proportion of day shifts on which 'DSCAMICUPositive_CAMICUPositiveDesc' response is 'Not collected' AND responses to ['DSHighestRASS_RASSScoreDesc' AND 'DSLowestRASS_RASSScoreDesc'] are both [-3 or -4 or -5]
Proportion of day shifts on which 'DSCAMICUPositive_CAMICUPositiveDesc' response is 'Not collected' AND responses to ['DSHighestRASS_RASSScoreDesc' AND 'DSLowestRASS_RASSScoreDesc'] are both [-2 or -1 or 0 or +1 or +2 or +3 or +4]
Night shift
Proportion of night shifts on which 'NSCAMICUPositive_CAMICUPositiveDesc' is YES OR NO (this is a definite response)
Proportion of night shifts on which 'NSCAMICUPositive_CAMICUPositiveDesc' response is 'RASS -3 but clinical team unable to assess CAM-ICU'
Proportion of night shifts on which 'NSCAMICUPositive_CAMICUPositiveDesc' response is 'Not collected' AND responses to ['NSHighestRASS_RASSScoreDesc' AND 'NSLowestRASS_RASSScoreDesc'] are both [-3 or -4 or -5]
Proportion of night shifts on which 'NSCAMICUPositive_CAMICUPositiveDesc' response is 'Not collected' AND responses to ['NSHighestRASS_RASSScoreDesc' AND 'NSLowestRASS_RASSScoreDesc'] are both [-2 or -1 or 0 or +1 or +2 or +3 or +4]

Rule 6: completeness of pain behaviour data
After rule 1:
Proportion of day shifts on which 'DSCompliance_VentilatorComplianceDesc' response is 'not collected by bedside nurse'
Proportion of day shifts on which 'DSCompliance_VentilatorComplianceDesc' response is NULL (ie no data)
Proportion of day shifts on which 'DSCompliance_VentilatorComplianceDesc' response is 'not collected by bedside nurse'
Proportion of day shifts on which 'DSCompliance_VentilatorComplianceDesc' response is NULL (ie no data)
Proportion of night shifts on which 'NSCompliance_VentilatorComplianceDesc' response is 'not collected by bedside nurse'
Proportion of night shifts on which 'NSCompliance_VentilatorComplianceDesc' response is NULL (ie no data)
Proportion of night shifts on which 'NSCompliance_VentilatorComplianceDesc' response is 'not collected by bedside nurse'
Proportion of night shifts on which 'NSCompliance_VentilatorComplianceDesc' response is NULL (ie no data)

Rule 7: indicative sedation guidance compliance
Shifts during which deep sedation was NOT requested
After rule 1:
Select shifts where response to 'DSDeepSedation_YesNoNotCollectedDesc' AND 'NSDeepSedation_YesNoNotCollectedDesc' is NO
For these shifts:
Proportion of each RASS score response to 'DSHighestRASS_RASSScoreDesc' AND 'NSHighestRASS_RASSScoreDesc'
These cumulative data should indicate how common it is for a patient in whom deep sedation was NOT requested for the patient NOT to achieve a highest recorded RASS of -2 or greater during the intervention period.

For peer review only - http://bmjopen.bmj.com/site/about/guidelines.xhtml

Rule 8: Correct administration of drugs according to group
After rule 1:
Patients allocated to usual care group
Number/proportion of days on which propofol administered 'Propofol_YesNoDesc'
Number/proportion of days on which dexmedetomidine administered 'Dexmedetomidine_YesNoDesc'
Number/proportion of days on which clonidine administered 'Clonidine_YesNoDesc'
Patients allocated to dexmedetomidine group
Number/proportion of days on which propofol administered 'Propofol_YesNoDesc'
Number/proportion of days on which dexmedetomidine administered 'Dexmedetomidine_YesNoDesc'
Number/proportion of days on which clonidine administered 'Clonidine_YesNoDesc'
Patients allocated to clonidine group
Number/proportion of days on which propofol administered 'Propofol_YesNoDesc'
Number/proportion of days on which dexmedetomidine administered 'Dexmedetomidine_YesNoDesc'
Number/proportion of days on which clonidine administered 'Clonidine_YesNoDesc'
This plot will give an overall indication of compliance without adjustment for the day of study.

Rule 9: correct administration according to group and day of study
Using Rule 8 data:
For each intervention group separately:
For study day 1, study day 2, study day 3 etc plot
Number/proportion of days on which propofol administered 'Propofol_YesNoDesc'
Number/proportion of days on which dexmedetomidine administered 'Dexmedetomidine_YesNoDesc'
Number/proportion of days on which clonidine administered 'Clonidine_YesNoDesc'
This plot will provide an indication of compliance according to the day of intervention (for patients remaining on mechanical ventilation.

For peer review only - http://bmjopen.bmj.com/site/about/guidelines.xhtml

SPIRIT 2013 Checklist: Recommended items to address in a clinical trial protocol and related documents*

Section/item	Item No	Description	Page
**Administrative information**			
Title	1	Descriptive title identifying the study design, population, interventions, and, if applicable, trial acronym	1
Trial registration	2a	Trial identifier and registry name. If not yet registered, name of intended registry	3
	2b	All items from the World Health Organization Trial Registration Data Set	?
Protocol version	3	Date and version identifier	7
Funding	4	Sources and types of financial, material, and other support	7, 19
Roles and responsibilities	5a	Names, affiliations, and roles of protocol contributors	1, 2
	5b	Name and contact information for the trial sponsor	18
	5c	Role of study sponsor and funders, if any, in study design; collection, management, analysis, and interpretation of data; writing of the report; and the decision to submit the report for publication, including whether they will have ultimate authority over any of these activities	18
	5d	Composition, roles, and responsibilities of the coordinating centre, steering committee, endpoint adjudication committee, data management team, and other individuals or groups overseeing the trial, if applicable (see Item 21a for data monitoring committee)	18
**Introduction**			

For peer review only

Background and rationale	6a	Description of research question and justification for undertaking the trial, including summary of relevant studies (published and unpublished) examining benefits and harms for each intervention	5-7
	6b	Explanation for choice of comparators	5-7, 12
Objectives	7	Specific objectives or hypotheses	7-8
Trial design	8	Description of trial design including type of trial (eg, parallel group, crossover, factorial, single group), allocation ratio, and framework (eg, superiority, equivalence, noninferiority, exploratory)	7, 14-15

Methods: Participants, interventions, and outcomes

Study setting	9	Description of study settings (eg, community clinic, academic hospital) and list of countries where data will be collected. Reference to where list of study sites can be obtained	10-11
Eligibility criteria	10	Inclusion and exclusion criteria for participants. If applicable, eligibility criteria for study centres and individuals who will perform the interventions (eg, surgeons, psychotherapists)	10-11
Intervention s	11a	Interventions for each group with sufficient detail to allow replication, including how and when they will be administered	11-13
	11b	Criteria for discontinuing or modifying allocated interventions for a given trial participant (eg, drug dose change in response to harms, participant request, or improving/worsening disease)	11-13
	11c	Strategies to improve adherence to intervention protocols, and any procedures for monitoring adherence (eg, drug tablet return, laboratory tests)	11-13, supplementary material
	11d	Relevant concomitant care and interventions that are permitted or prohibited during the trial	11-13
Outcomes	12	Primary, secondary, and other outcomes, including the specific measurement variable (eg, systolic blood pressure), analysis metric (eg, change from baseline, final value, time to event), method of aggregation (eg, median, proportion), and time point for each outcome. Explanation of the clinical relevance of chosen efficacy and harm outcomes is strongly recommended	8-10, table 1<br><br>Explanation/rationale 6-7

For peer review only - http://bmjopen.bmj.com/site/about/guidelines.xhtml

Participant timeline	13	Time schedule of enrolment, interventions (including any run-ins and washouts), assessments, and visits for participants. A schematic diagram is highly recommended (see Figure)	11-12, 14 Table 3
Sample size	14	Estimated number of participants needed to achieve study objectives and how it was determined, including clinical and statistical assumptions supporting any sample size calculations	15-17
Recruitment	15	Strategies for achieving adequate participant enrolment to reach target sample size	11, 17

Methods: Assignment of interventions (for controlled trials)

Allocation:

Sequence generation	16a	Method of generating the allocation sequence (eg, computer-generated random numbers), and list of any factors for stratification. To reduce predictability of a random sequence, details of any planned restriction (eg, blocking) should be provided in a separate document that is unavailable to those who enrol participants or assign interventions	11-12
Allocation concealment mechanism	16b	Mechanism of implementing the allocation sequence (eg, central telephone; sequentially numbered, opaque, sealed envelopes), describing any steps to conceal the sequence until interventions are assigned	11-12
Implementation	16c	Who will generate the allocation sequence, who will enrol participants, and who will assign participants to interventions	11-12
Blinding (masking)	17a	Who will be blinded after assignment to interventions (eg, trial participants, care providers, outcome assessors, data analysts), and how	N/A
	17b	If blinded, circumstances under which unblinding is permissible, and procedure for revealing a participant's allocated intervention during the trial	N/A

Methods: Data collection, management, and analysis

Data collection methods	18a	Plans for assessment and collection of outcome, baseline, and other trial data, including any related processes to promote data quality (eg, duplicate measurements, training of assessors) and a description of study instruments (eg, questionnaires, laboratory tests) along with their reliability and validity, if known. Reference to where data collection forms can be found, if not in the protocol	13, 14 table 3
	18b	Plans to promote participant retention and complete follow-up, including list of any outcome data to be collected for participants who discontinue or deviate from intervention protocols	13, table 3
Data manageme nt	19	Plans for data entry, coding, security, and storage, including any related processes to promote data quality (eg, double data entry; range checks for data values). Reference to where details of data management procedures can be found, if not in the protocol	13
Statistical methods	20a	Statistical methods for analysing primary and secondary outcomes. Reference to where other details of the statistical analysis plan can be found, if not in the protocol	14 (analytic framework) 14-17 statistical methods Statistical analysis plan (SAP)included as supplementary material
	20b	Methods for any additional analyses (eg, subgroup and adjusted analyses)	17
	20c	Definition of analysis population relating to protocol non-adherence (eg, as randomised analysis), and any statistical methods to handle missing data (eg, multiple imputation)	Estimand included in SAP

Methods: Monitoring

Data monitoring	21a	Composition of data monitoring committee (DMC); summary of its role and reporting structure; statement of whether it is independent from the sponsor and competing interests; and reference to where further details about its charter can be found, if not in the protocol. Alternatively, an explanation of why a DMC is not needed	18

	21b	Description of any interim analyses and stopping guidelines, including who will have access to these interim results and make the final decision to terminate the trial	Not Applicable
Harms	22	Plans for collecting, assessing, reporting, and managing solicited and spontaneously reported adverse events and other unintended effects of trial interventions or trial conduct	Pre-defined AEs collected in protocol table 3 AE/SAE reporting 18
Auditing	23	Frequency and procedures for auditing trial conduct, if any, and whether the process will be independent from investigators and the sponsor	Monitoring plan 18
**Ethics and dissemination**			
Research ethics approval	24	Plans for seeking research ethics committee/institutional review board (REC/IRB) approval	18
Protocol amendments	25	Plans for communicating important protocol modifications (eg, changes to eligibility criteria, outcomes, analyses) to relevant parties (eg, investigators, REC/IRBs, trial participants, trial registries, journals, regulators)	18
Consent or assent	26a	Who will obtain informed consent or assent from potential trial participants or authorised surrogates, and how (see Item 32)	10
	26b	Additional consent provisions for collection and use of participant data and biological specimens in ancillary studies, if applicable	10
Confidentiality	27	How personal information about potential and enrolled participants will be collected, shared, and maintained in order to protect confidentiality before, during, and after the trial	18
Declaration of interests	28	Financial and other competing interests for principal investigators for the overall trial and each study site	19
Access to data	29	Statement of who will have access to the final trial dataset, and disclosure of contractual agreements that limit such access for investigators	19
Ancillary and post-trial care	30	Provisions, if any, for ancillary and post-trial care, and for compensation to those who suffer harm from trial participation	18

For peer review only

Dissemination policy	31a	Plans for investigators and sponsor to communicate trial results to participants, healthcare professionals, the public, and other relevant groups (eg, via publication, reporting in results databases, or other data sharing arrangements), including any publication restrictions	18
	31b	Authorship eligibility guidelines and any intended use of professional writers	Not applicable
	31c	Plans, if any, for granting public access to the full protocol, participant-level dataset, and statistical code	19
**Appendices**			
Informed consent materials	32	Model consent form and other related documentation given to participants and authorised surrogates	Supplementary material
Biological specimens	33	Plans for collection, laboratory evaluation, and storage of biological specimens for genetic or molecular analysis in the current trial and for future use in ancillary studies, if applicable	Not Applicable

*It is strongly recommended that this checklist be read in conjunction with the SPIRIT 2013 Explanation & Elaboration for important clarification on the items. Amendments to the protocol should be tracked and dated. The SPIRIT checklist is copyrighted by the SPIRIT Group under the Creative Commons "Attribution-NonCommercial-NoDerivs 3.0 Unported" license.

For peer review only
